# Azolium‐Porphyrin Electrosynthesis

**DOI:** 10.1002/cssc.202401439

**Published:** 2024-10-22

**Authors:** Fatima Akhssas, Rongning Lin, Michal Trojan, Ludivine Poyac, Nesrine Amiri, Thibault Ertel, Sophie Fournier, Emmanuel Lerayer, Hélène Cattey, Sébastien Clément, Sébastien Richeter, Charles H. Devillers

**Affiliations:** ^1^ Institut de Chimie Moléculaire de l'Université de Bourgogne UMR6302 CNRS Univ. Bourgogne 9 avenue Alain Savary 21000 Dijon France; ^2^ Department of Organic Chemistry University of Chemistry and Technology, Prague Technická 5 Prague 6 166 28 Czech Republic; ^3^ ICGM Univ. Montpellier CNRS ENSCM 34293 Montpellier France

**Keywords:** Porphyrinoids, Azolium, Electrochemistry, Electrosynthesis, Azole nucleophiles, Anodic nucleophilic substitution

## Abstract

Electrochemical oxidation of Zn(II) 2,7,12,17‐tetra‐*tert*‐butylporphyrin in the presence of a series of azole derivatives (1‐methylimidazole, 1‐vinyl‐1*H*‐imidazole, 2‐(1*H*‐imidazol‐1‐yl)pyridine, 1‐methylbenzimidazole, 1‐methyl‐1*H*‐1,2,4‐triazole, and benzothiazole) affords the corresponding *meso*‐substituted azolium‐porphyrins in very mild conditions and good yields. It was found that these nucleophiles were strongly ligated to the zinc(II) azolium‐porphyrin complexes. Thus a demetalation/remetalation procedure was performed to recover the non‐azole‐coordinated zinc(II) complexes. X‐ray crystallographic structures of three azolium‐porphyrins were solved. Cyclic voltammetry analyses provided insight into the electron‐withdrawing effect of the azolium substituents.

## Graphical abstract

Azolium porphyrins have been synthesized for the first time by electrochemical oxidation of Zn(II) 2,7,12,18‐tetra‐tert‐butylporphyrin in the presence of the corresponding azole nucleophiles with high regioselectivity under very mild conditions. These cationic salts have been characterized by state‐of‐the‐art techniques such as NMR, UV‐Vis., MS, X‐ray crystallography and cyclic voltammetry.

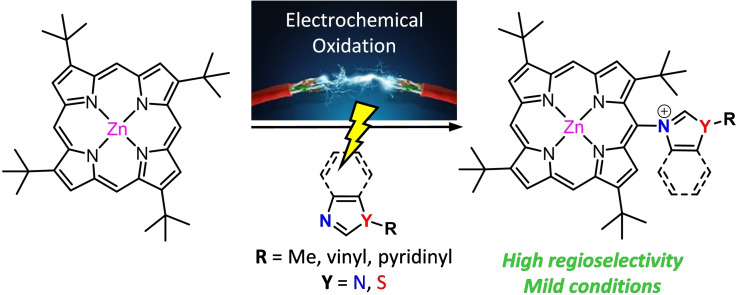

## Introduction

**Scheme 1:**
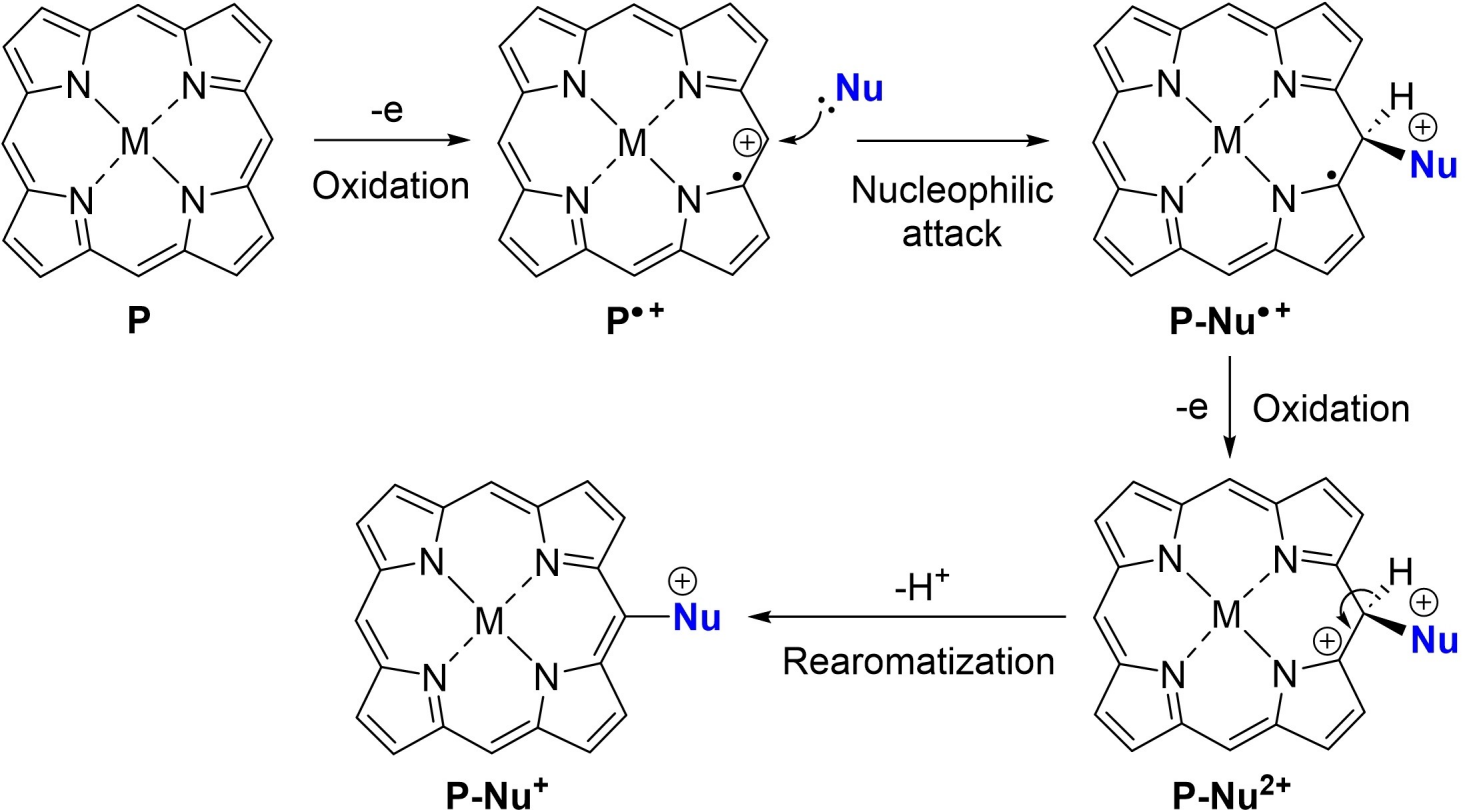
Suggested mechanism for the S_N_A reaction with a porphyrin and a nucleophile (Nu=pyridine,[^6^, ^7^, ^8^, ^9^, ^10^, ^11^] triphenylphosphine,[^9^, ^12^] or azoles (this work)).

The functionalization of porphyrins, the pigments of life, is an important area of research since these macrocycles are involved in numerous applications ranging from photovoltaic solar cells,^[1]^ electro‐ and photocatalysis[^2^, ^3^] to photodynamic therapy.^[4]^ Despite the considerable efforts that have been devoted to this task for several decades, more efficient and (regio)selective functionalization reactions of porphyrins are still being developed today. Most of these functionalizations are based on chemical transformations, *i. e*., involving commercial or non‐commercial chemical reagents. For more than a decade, our group has been interested in the electrochemical transformation of porphyrins,^[5]^ especially in the oxidative functionalization of the porphyrin ring. We have shown, like others,[^6^, ^7^, ^8^, ^9^, ^10^, ^11^] that the porphyrin core can be efficiently and (regio)selectively functionalized with different nucleophiles (pyridine,[^6^, ^7^, ^8^, ^9^, ^10^, ^11^] triphenylphosphine,[^9^, ^12^] nitrite,[^13^, ^14^] chloride…[^13^, ^15^]) by electrochemical oxidation. The reaction mechanism involves the electrochemical oxidation of the porphyrin ring affording its cation radical, the nucleophilic attack of the nucleophile on the porphyrin, the re‐oxidation of the resulting intermediate and finally the re‐aromatization *via* the loss of a proton. In general, this anodic nucleophilic substitution (S_N_A) reaction involves the abstraction of two electrons and the release of one proton (Scheme 1).

Recently, we have developed a similar but intramolecular strategy to extend the conjugation of pyri(mi)dinyl porphyrins *via* an oxidative C−N fusion reaction.[^16^, ^17^, ^18^] In 2014, Yoshida and co‐workers showed that the galvanostatic electrochemical oxidation of phenyl‐based or naphthalene compounds in the presence of *N*‐protected imidazoles led, after *in situ* deprotection, to the *N*‐aryl‐imidazole derivatives.^[19]^ In 2015, we developed a sustainable method for the synthesis of azolium‐pyrene derivatives under potentiostatic and galvanostatic conditions.^[20]^ Given the increasing interest in the development and applications of imidazole/imidazolium‐porphyrins,^[21]^ as well as the impressive resurgence of electrochemistry as a clean and sustainable way to transform and functionalize organic and organometallic derivatives,[^22^, ^23^, ^24^, ^25^] we wanted to explore this electrochemical route, as never done before for porphyrins. If we only consider the reactions that allows to functionalize the porphyrin ring with imidazole/imidazolium, the first example was described in 1976 by Fuhrhop and co‐workers. Thus, Mg(II) octaethylporphyrin, MgOEP, was functionalized in the *meso* position with an imidazole or imidazolium motifs by oxidation with benzoyl peroxide in the presence of imidazole. However, although the yield reported by the authors was 60 %, no experimental proof was provided. Three years later, Smith and co‐workers^[26]^ reported that the oxidation of porphyrin by Tl(III) in the presence of a large excess of imidazole provided imidazole‐porphyrin with a 7 % yield. In 2012 and then 2014, Ruppert, Weiss and co‐workers[^27^, ^28^] described the three‐step synthesis of *meso*‐azolylporphyrins. *Meso*‐bromination was carried out followed by an Ullmann coupling with an azole (imidazole, benzimidazole, 1,2,4‐triazole). Finally, the azole moiety was alkylated with *n*‐butyl iodide. In 2014, Devillers, Richeter and co‐workers^[29]^ reported another method of accessing *meso*‐imidazole‐porphyrin derivatives *via* an aromatic nucleophilic substitution reaction of a Ni(II) *meso*‐nitro‐porphyrin, method that was later improved.^[30]^ In 2007 and then 2010, Richeter and his collaborators reported a new multi‐step synthesis of fused imidazolium‐porphyrins starting with a nickel(II) 5,10,15,20‐tetra(*p*‐*tert*‐butylphenyl)porphyrin.[^31^, ^32^, ^33^]

**Scheme 2:**
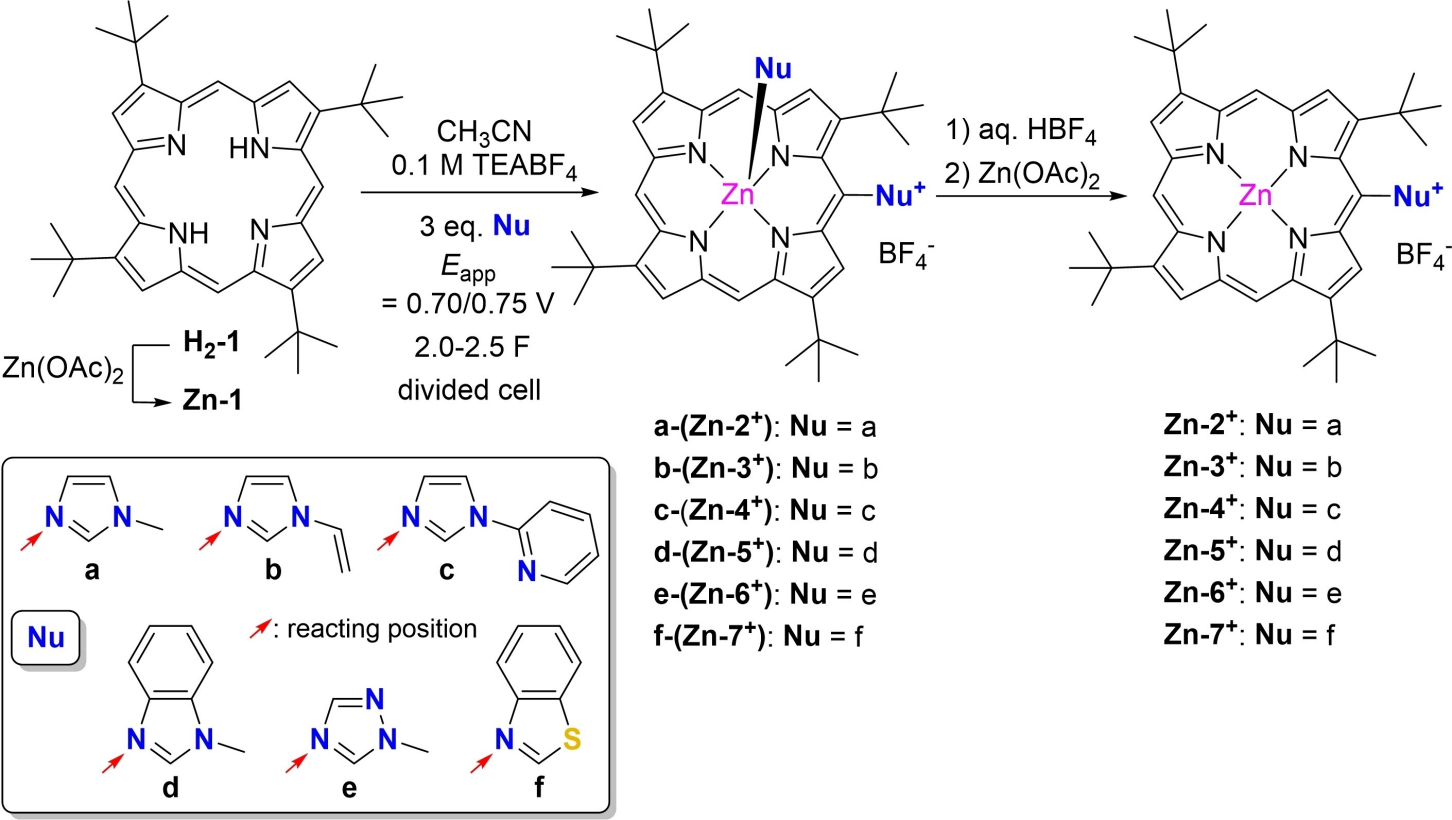
Electrochemical functionalization of **Zn‐1** with azole nucleophiles **a‐**‐**f**.

Although these different synthesis strategies provide azolium‐porphyrin salts in moderate to good yields, it is interesting to develop new, selective and straightforward methods, without using metal‐based reagents or catalysts and by reducing as much as possible the amount of chemical waste. In this context, this work describes the electrochemical synthesis of original azolium‐porphyrins starting with zinc(II) 2,7,12,17‐tetra‐*tert*‐butylporphyrin (**Zn‐1**) and the following nucleophiles: 1‐methylimidazole (**a**), 1‐vinyl‐1*H*‐imidazole (**b**), 2‐(1*H*‐imidazol‐1‐yl)pyridine (**c**), 1‐methyl‐benzimidazole (**d**), 1‐methyl‐1*H*‐1,2,4‐triazole (**e**), and benzothiazole (**f**) (Scheme 2).

## Results and Discussion

**Figure 1:**
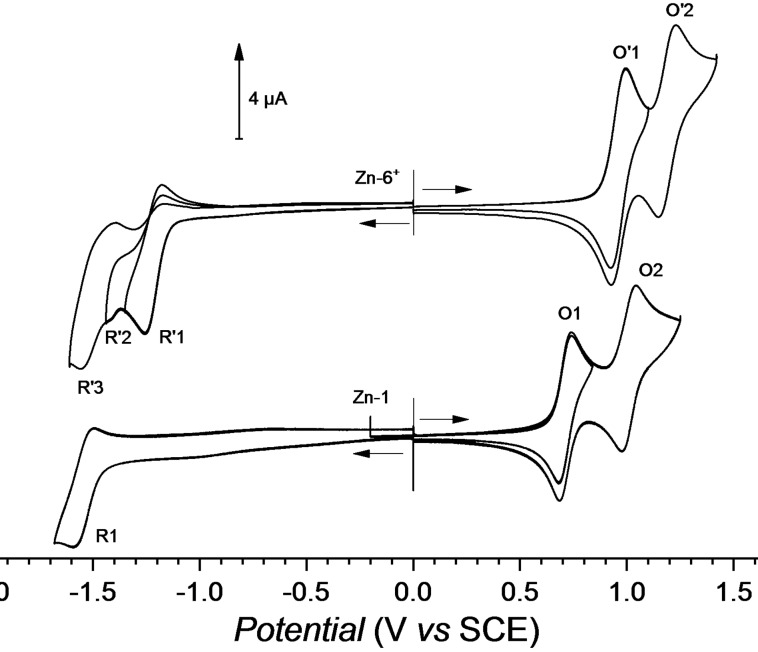
Cyclic voltammograms of **Zn‐1** and **Zn‐6^+^** in 0.1 M TEABF_4_/CH_3_CN, WE: Pt, *Ø*=2 mm, *c*=10^−3^ M, *ν*=100 mV s^−1^.

**Zn‐1** was chosen since it exhibits good solubility in organic solvents, has a relatively low oxidation potential (see below) and has never been functionalized by electrochemistry. The free base 2,7,12,17‐tetra‐*tert*‐butylporphyrin (**H_2_‐1**) was synthesized following the protocol patented by Nickel and Liebeskind^34,35^ and purified according to Whitlock.^36^ To improve the solubility of **H_2_‐1** and increase its electron density, the zinc(II) complex **Zn‐1** was prepared quantitatively.^17^ The ^1^H NMR spectrum of **Zn‐1** in DMSO‐d_6_ (Figure S62) exhibits two singlets at 10.48 (4H) and 9.35 ppm (4H), which correspond respectively to the protons of the *meso* and *β*‐pyrrolic positions and a singlet at 2.28 ppm attributed to the *tert*‐butyl protons (36 H). Given the good solubility of **Zn‐1** in acetonitrile, all the studies were performed in this solvent. Indeed, acetonitrile is one of the best solvents for oxidation reactions since it offers a very large positive potential range and does not suffer from chlorination side reaction that generally occurs in chlorinated solvents. The cyclic voltammogram (CV) of **Zn‐1** (Figure 1) shows two reversible monoelectronic oxidations (**Zn‐1**→**Zn‐1^⋅+^** →**Zn‐1^2+^**) at *E*_1/2_(O1)=0.71 V (Δ*E*_p_=60 mV) and *E*_1/2_ (O2)=1.01 V (Δ*E*_p_=60 mV, all the potential values of this paper are given *vs* the Saturated KCl Calomel Electrode, SCE, see also Table 1 for potential values of the porphyrins). In the negative potential range, only one reduction is observed and it corresponds to the pseudo‐reversible formation of the porphyrin anion radical (*E*_pc_(R1)=−1.59 V). When this porphyrin is electrolyzed at its first oxidation peak (*E*_app_=0.75 V), without nucleophile, the corresponding cation radical **Zn‐1^⋅+^** does not dimerize unlike other *meso*‐free porphyrins^37–41^ and is thus relatively stable at ambient temperature under inert atmosphere, at least at the electrolysis time scale (20 min., Figure S66). The cation radical **Zn‐1^⋅+^** can be fully reduced back to the initial **Zn‐1** (Figure S67). This behavior has already been observed for the sterically hindered zinc(II) 2,3,7,8,12,13,17,18‐octaethylporphyrin (**ZnOEP**).^42^ However, when **Zn‐1^⋅+^** is generated in the presence of nucleophiles, either the substitution product is obtained in good yield or, if the nucleophile is not sufficiently nucleophilic (we have tested the challenging benzoxazole and cafeine), no reaction occurs. After the addition of 3 equivalents of **a**–**f**, that corresponds to the optimized nucleophile amount used for the electrosynthesis, the first oxidation is still reversible indicating that the nucleophilic

**Table 1:** Potential values of the studied porphyrins *vs* the saturated KCl calomel reference electrode.

Porphyrin	Red2 (V)	Red1 (V)	Ox1 (V)	Ox2 (V)	HOMO‐LUMO gap (V)^[c]^
Zn‐1		−1.59^[a]^	0.71 (60)	1.01 (60)	2.30
Zn‐2^+^	−1.63^[a]^	−1.43^[a]^	0.89 (80)	1.19^[a]^	2.32
Zn‐3^+^	−1.37^[a]^	−1.29^[a]^	0.94 (70)	1.17 (70)	2.23
Zn‐4^+^	−1.47^[a]^	−1.35^[a]^	0.91 (70)	1.15 (90)	2.26
Zn‐5^+^	−1.62^[a]^	−1.36^[a]^	0.91 (70)	1.14 (80)	2.27
Zn‐6^+^	−1.56^[a]^	−1.26^[a]^	0.96 (80)	1.19 (80)	2.22
f‐(Zn‐7^+^)	−1.43^[a]^	−1.30^[a]^	0.61 (80)	0.89^[a]^	1.91

[a] *E*_pc_ are given for these irreversible or pseudo‐reversible systems. [b] *E*_1/2_=(*E*_pa_+*E*_pc_)/2 is given for these reversible systems, the values written into brackets corresponds to Δ*E*_p_=*E*_pa_−*E*_pc_ (in mV). [c] This experimental HOMO‐LUMO gap corresponds to the potential difference between Ox1 and Red1.

attack on **Zn‐1^⋅+^** is kinetically slow compared to the time scale of the cyclic voltammetry experiment. The significant steric hindrance of the *tert*‐butyl groups close to the *meso* position of **Zn‐1** accounts for this behavior. The electrolysis of **Zn‐1** was performed in the same conditions as those used for the electrochemical analysis, by applying a potential corresponding to the formation of **Zn‐1^⋅+^** (*E*_app_=0.70–0.76 V). When **Zn‐1** was consumed, the reaction was stopped. After elimination of the solvent and the supporting electrolyte, the crude solution was analyzed by ^1^H NMR spectroscopy. The ^1^H NMR spectrum supported the almost quantitative substitution of a single *meso* position of the macrocycle. The formation of mono‐substituted products was further confirmed by MALDI‐TOF mass spectrometry measured with the crude reaction mixture. The azolium‐porphyrins were then purified by column chromatography. The Zn(II) atom of the isolated compounds was systematically coordinated by the nucleophile used in the reaction (**a‐**(**Zn‐2^+^**), **b‐**(**Zn‐3^+^**), **c‐**(**Zn‐4^+^**), **d‐**(**Zn‐5^+^**), **e‐**(**Zn‐6^+^**) and **f‐**(**Zn‐7^+^**), Scheme 2). Despite several extraction and/or precipitation attempts to remove the coordinated ligand, it was not possible to isolate the pure uncoordinated products **Zn‐2^+^**, **Zn‐3^+^**, **Zn‐4^+^**, **Zn‐5^+^**, **Zn‐6^+^** and **Zn‐7^+^** (Scheme 2). For further applications in catalysis, it is important to remove these coordinated ligands. Thus, demetalation of the Zn(II) complexes was carried out using 10 equivalents of HBF_4_ (50 % in water), followed by water washes and precipitation. Then, Zn(II) remetalation was performed. As **f‐**(**Zn‐7^+^**) degraded during this acidic work‐up, this axially‐coordinated compound was isolated after water washes and precipitation. Experimental conditions and isolated yields for the electrosynthesis of azolium‐porphyrins are reported in Table 2.

**Table 2:** Main experimental conditions and isolated yields for the electrochemical synthesis of azolium‐porphyrins.^[a]^

Azolium‐porphyrin	Nucleophile (Nu)	eq. of Nu	Eapp (V)	Charge (F)	Yield (%)
Zn‐2^+^	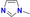	3	0.72	2.5	64
Zn‐3^+^	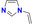	3	0.70	2.7	75
Zn‐4^+^	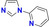	3	0.70	2.2	87
Zn‐5^+^	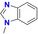	3	0.74	2.0	74
Zn‐6^+^	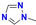	3	0.74	2.5	63
f‐(Zn‐7^+^)	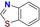	3	0.76	2.0	97^[b]^

[a] Three‐step yields (electrosynthesis, Zn demetalation, Zn(II) remetalation), see ESI for experimental details. [b] One step yield (electrosynthesis only).

**Figure 2:**
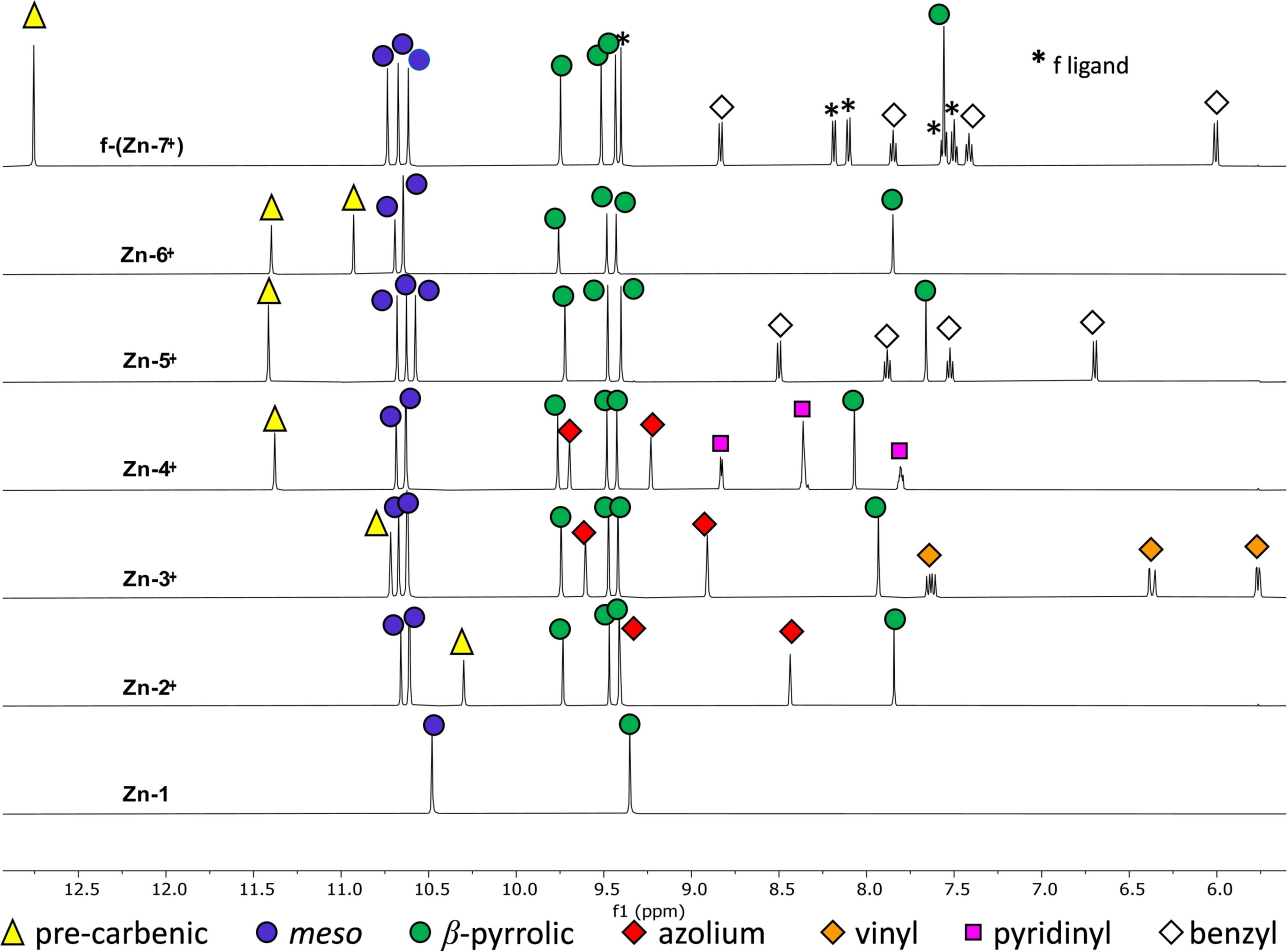
^1^H NMR spectra of **Zn‐2^+^**, **Zn‐3^+^**, **Zn‐4^+^**, **Zn‐5^+^**, and **f‐**(**Zn‐7^+^**) (DMSO‐d_6_, 500 MHz, 400 MHz for **Zn‐6^+^**, 298 K).

The ^1^H NMR spectra (Figure 2) of **Zn‐2^+^**, **Zn‐3^+^**, **Zn‐4^+^**, **Zn‐5^+^**, **Zn‐6^+^** and **f‐**(**Zn‐7^+^**) show very similar porphyrin signals.

**Figure 3:**
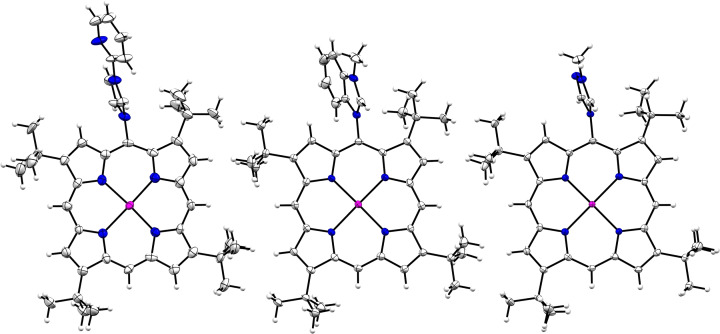
X‐ray crystallographic structure of **Zn‐4^+^**, **Zn‐5^+^** and **Zn‐6^+^**. BF_4_^−^ anion and solvents were omitted for clarity (see ESI for full characterization). Thermal ellipsoids are scaled to the 50 % probability level.

Only three singlets attributed to *meso* protons appear at low field, between 10.58 and 10.73 ppm confirming the functionalization of the porphyrin at the *meso* position. Three β‐pyrrolic protons are observed as singlets between 9.41 and 9.76 ppm while the β‐pyrrolic proton close to the *meso*‐functionalized position appears much more shielded between 7.55 and 8.07 ppm. This remarkable shielding is due to its location in the anisotropy cone of the azolium group which is orthogonal to the porphyrin plane, as confirmed by the X‐ray crystallographic structures of **Zn‐4^+^, Zn‐5^+^**, and **Zn‐6^+^** (see below). This shielding effect is also observed on the protons of the neighboring *tert*‐butyl group since it appears between 1.42 and 1.71 ppm while the three other *tert*‐butyl singlets are located between 2.04 and 2.29 ppm. Concerning the pre‐carbenic protons, the corresponding singlets are located between 12.75 and 10.30 ppm for the six porphyrins. In the particular case of the porphyrins **Zn‐2^+^**, **Zn‐5^+^** and **Zn‐6^+^**, the methyl protons are observed as a singlet between 4.29 and 4.68 ppm while the vinyl protons of **Zn‐3^+^** appear as doublet of doublets between 5.75 and 7.62 ppm. For the porphyrins **Zn‐2^+^**, **Zn‐3^+^** and **Zn‐4^+^**, the two proton signals attributed to the imidazolium fragment are located between 8.43 and 9.69 ppm. The benzene moiety of porphyrin **Zn‐5^+^** appears as two doublets and two triplets (superimposed doublet of doublet) between 6.70 and 8.50 ppm. Finally, the doublets and multiplets of the pyridine motif of porphyrin **Zn‐4^+^** appear between 7.79 and 8.82 ppm. The Soret bands of **Zn‐2^+^**−**Zn‐6^+^** appear in DMSO at 414–416 nm, *i. e*., a bathochromic shift of 4–6 nm as compared to **Zn‐1**. Furthermore, the Q bands are practically superimposable for **Zn‐2^+^**−**Zn‐6^+^** and are located at 543–544 and 581–582 nm, *i. e*., a bathochromic shift of 6–7 and 9–10 nm compared to **Zn‐1**. The HOMO‐LUMO band gap was also extracted from the lower energy Q band. It gives a similar trend than the one measured by electrochemistry: 2.16 eV was measured for **Zn‐1** while it slightly decreases to 2.12–2.13 eV for the azolium‐porphyrins. Single crystals analyzable by X‐ray diffraction were obtained for **Zn‐4^+^**, **Zn‐5^+^** and **Zn‐6^+^** (Figure 3). The crystallographic structures confirm the single functionalization at the *meso* position and the formation of the azolium cations is confirmed by the presence of the BF_4_^−^ anion.

The C−N bond between the *meso* carbon atom of the porphyrin and the nitrogen atom of the triazolium fragment ranges from 1.457–1.463 Å. The porphyrins **Zn‐2^+^**, **Zn‐3^+^**, **Zn‐4^+^**, **Zn‐5^+^**, **Zn‐6^+^** and **f‐**(**Zn‐7^+^**) were analyzed by cyclic voltammetry (CV) in 0.1 M acetonitrile TEABF_4_ (Figures S9, S18, S27, S36, S45 and S54, respectively and see Table 1 for potential values of the porphyrins). The voltammograms present two reversible oxidation systems (reversible formation of the cation and dication radical). The redox properties depend on the azolium substituent linked to the porphyrin. By comparing the value of the first oxidation peak of the **Zn‐1** precursor (*E*_pa_(O1)=0.74 V) to that of the substituted porphyrins, a shift towards more positive potentials is observed (Δ*E* between +180 and +250 mV), as expected for cationic electron‐withdrawing derivatives. The substituents can be classified in the following way, from the last electron‐withdrawing to the most electron‐withdrawing: methylimidazolium (Δ*E*=+180 mV)<methylbenzimidazolium (Δ*E*=+190 mV)<(2‐pyridinyl) imidazolium (Δ*E*=+200 mV)<vinylimidazolium (Δ*E*=+230 mV)<methyl‐1,2,4‐triazolium (Δ*E*=+250 mV). As **f‐**(**Zn‐7^+^**) is coordinated by one benzothiazole molecule, its redox properties cannot be compared to the other non‐coordinated azolium‐porphyrins **Zn‐2^+^**−**Zn‐6^+^**. In the negative potential range, porphyrins that are more difficult to oxidize are logically easier to reduce. For this reason, the porphyrin substituted with the triazolium moiety (**Zn‐6^+^**) exhibits reduction at a less negative potential than other azolium‐porphyrins (*E*_pc_(Red1)=−1.25 V). The poorly reversible cathodic peaks indicates that some chemical reaction(s) following the formation of the anion radical during the voltammetric analysis takes place. The potential difference between the first oxidation and the first reduction of the porphyrin (HOMO‐LUMO gap) is practically identical for all the porphyrins (from 2.22–2.32 eV) which confirms that these two redox systems are centered on the porphyrin ring.

## Conclusions

In conclusion, a series of azolium‐porphyrins was efficiently prepared by anodic nucleophilic substitution of **Zn‐1** with 1‐methylimidazole, 1‐vinyl‐1*H*‐imidazole, 2‐(1*H*‐imidazol‐1‐yl)pyridine, 1‐methylbenzimidazole, 1‐methyl‐1*H*‐1,2,4‐triazole, and benzothiazole, as never done for porphyrins. The electrolysis was carried out under mild conditions and led to satisfactory yields. The corresponding electrogenerated azolium‐porphyrin zinc(II) complexes are systematically coordinated by the initial imine‐based nucleophiles that are used in slight excess. Removal of the axial ligand was performed thanks to a demetalation/zinc(II) remetalation sequence. The target products were then obtained with high purity and good yields. Their possible applications in catalysis will be tested.

## Supporting Information Summary

Experimental details and experimental procedures for the preparation of porphyrins, NMR spectra, HRMS spectra, UV‐visible absorption spectra, cyclic voltammograms for all compounds. CCDC 2363632 (**Zn‐4^+^**), 2363633 (**Zn‐5^+^**) and 2363634 (**Zn‐6^+^**), contain the supplementary crystallographic data for this paper. These data can be obtained free of charge via www.ccdc.cam.ac.uk/data request/ cif, or by emailing data request@ccdc.cam.ac.uk, or by contacting The Cambridge Crystallographic Data Centre, 12 Union Road, Cambridge CB2 1EZ, UK; fax: +44 1223 336033. The authors have cited additional references within the Supporting Information.^36,43‐50^

## Conflict of Interests

The authors declare no conflict of interest.

1

## Supporting information

As a service to our authors and readers, this journal provides supporting information supplied by the authors. Such materials are peer reviewed and may be re‐organized for online delivery, but are not copy‐edited or typeset. Technical support issues arising from supporting information (other than missing files) should be addressed to the authors.

Supporting Information

## Data Availability

The data that support the findings of this study are available from the corresponding author upon reasonable request.
