# Supplementary material for: Azolium‐Porphyrin Electrosynthesis
Source: ChemSusChem. 2024 Oct 22;17(24):e202401439. doi: 10.1002/cssc.202401439 (PMC11660742; doi:10.1002/cssc.202401439)
Supplement: Supplementary file 1 — Supporting Information [file CSSC-17-e202401439-s001.pdf]

ChemSusChem

Supporting Information

Azolium-Porphyrin Electrosynthesis

Fatima Akhssas, Rongning Lin, Michal Trojan, Ludivine Poyac, Nesrine Amiri, Thibault Ertel, Sophie Fournier, Emmanuel Lerayer, Hélène Cattey, Sébastien Clément, Sébastien Richeter,* and Charles H. Devillers*

Azolium-Porphyrin Electrosynthesis

Fatima Akhssas,[a] Rongning Lin,[a] Michal Trojan,[a,b] Ludivine Poyac,[c] Nesrine Amiri,[a] Thibault Ertel,[a] Sophie Fournier,[a] Emmanuel Lerayer,[a] Hélène Cattey,[a] Sébastien Clément,[c] Sébastien Richeter,*[,c] and Charles H. Devillers*[,a]

[a] Institut de Chimie Moléculaire de l'Université de Bourgogne UMR6302, CNRS, Univ. Bourgogne, 9 avenue Alain Savary 21000 Dijon, France

[b] Department of Organic Chemistry, University of Chemistry and Technology, Prague, Technická 5, 166 28 Prague 6, Czech Republic.

[c] ICGM, Univ. Montpellier, CNRS, ENSCM, Montpellier 34293, France

* Sébastien Richeter – ICGM, Univ Montpellier, CNRS, ENSCM, Montpellier 34293, France, orcid.org/0000-0001-5284-0931 ; Email: sebastien.richeter@umontpellier.fr
* Charles H. Devillers – Institut de Chimie Moléculaire de l'Université de Bourgogne (ICMUB), UMR CNRS 6302, 9 avenue Alain Savary, 21078 Dijon cedex, Univ. Bourgogne, France; orcid.org/0000-0001-9078-7035; Email: charles.devillers@u-bourgogne.fr

Table of Content

General Comments 2
Instruments and methods 2
Electrochemistry 3
Voltammetric analyses 3
Bulk electrolyzes 4
Electrosynthesis of azolium-porphyrins 4
Electrosynthesis of Zn-2$^+$ 4
Characterization of Zn-2$^+$ 5
Electrosynthesis of Zn-3$^+$ 14
Characterization of Zn-3$^+$ 16
Electrosynthesis of Zn-4$^+$ 25
Characterization of Zn-4$^+$ 27
Electrosynthesis of Zn-5$^+$ 35
Characterization of Zn-5$^+$ 37
Electrosynthesis of Zn-6$^+$ 45
Characterization of Zn-6$^+$ 47

Electrosynthesis of f-($Zn-7^+$) ... 55
Characterization of f-($Zn-7^+$) ... 56
Synthesis and characterization of Zn-1 ... 64
Characterization of Zn-1 ... 65
Oxidation of Zn-1 followed by UV-visible absorption spectroelectrochemistry. ... 72
X-ray crystallographic data ... 74
$Zn-4^+$ ... 74
$Zn-5^+$ ... 95
$Zn-6^+$ ... 106
References ... 116

General Comments

Unless otherwise noted, all reactions were carried out with protection from air. CH_2Cl_2, MeCN and DMF were dried over alumina cartridges using a solvent purification system PureSolv PS-MD-5 model from Innovative Technology and kept under argon. All other solvents used for reactions were obtained from commercial suppliers and used as received. **Zn-1** was synthesized according to known procedures.[1–4] The TLC analyzes were carried out on Merck DC Kieselgel 60 F-254 aluminium sheets. The spots were directly visualized or through illumination with UV lamp (λ = 254/365 nm). Column chromatography purifications were performed manually on silica gel (SiO_2, 40-63 μm) from Sigma-Aldrich (technical grade).

Instruments and methods

^{1}H-, ^{13}C- spectra were recorded either on a Bruker Avance 300, on a Bruker Avance 500 or on a Bruker Avance 600 III HD spectrometer. Chemical shifts are expressed in parts per million (ppm) from the residual non-deuterated solvent signal.[5] *J* values are expressed in Hz. Spectra were calibrated to TMS on the basis of the relative chemical shift of the residual non-deuterated solvent signal as an internal standard. High-resolution mass spectra (HRMS) were recorded either on a Thermo LTQ Orbitrap XL apparatus equipped with an ESI source or on a Bruker UltraflexII LRF 2000 MALDI-TOF mass spectrometer (matrix: dithranol). UV-visible absorption spectra were recorded on a VARIAN Cary 50 UV-Visible spectrophotometer using quartz cells.

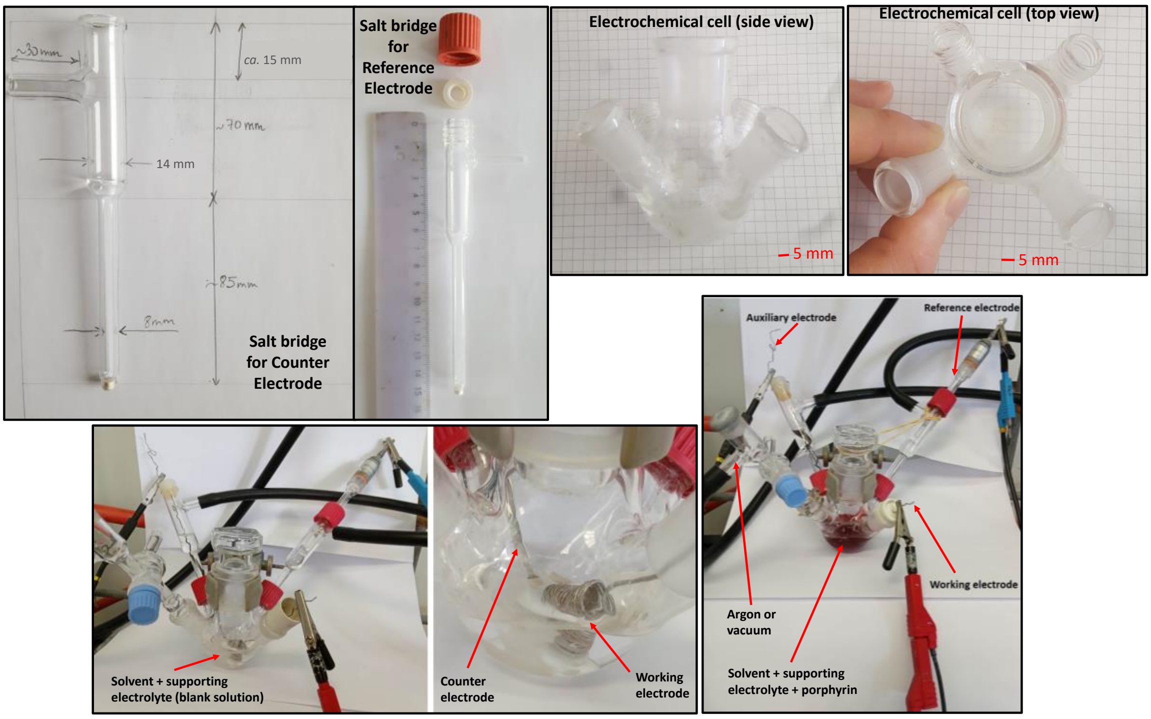

Figure S1. Electrochemical cell setup

Electrochemistry

All manipulations were performed using Schlenk techniques in an atmosphere of dry oxygen-free argon at room temperature (T = 20°C ± 3°C). The supporting electrolyte (tetraethylammonium tetrafluoroborate ($TEABF_4$) was degassed under vacuum before use and then dissolved in CH_3CN (unless otherwise noted) to a concentration of 0.1 mol L^{-1}. The following electrolyte volumes (unless otherwise noted) were used for voltammetric analyses and bulk electrolyses: cathodic compartment: V = 5 mL; reference electrode compartment: V = 5 mL.

Voltammetric analyses

Voltammetric analyses were carried out in a standard three-electrode cell, with Biologic SP-300 potentiostat, connected to an interfaced computer that employed EC-Lab (v. 11.43) software. The reference electrode was a saturated calomel electrode (SCE) separated from the analyzed solution by a sintered glass disk filled with the background solution. The auxiliary electrode was a platinum foil separated from the analyzed solution by a sintered glass disk filled with the background solution. For all voltammetric measurements, the working electrode was a platinum electrode ($Ø$ = 1.6 mm). Before each voltammetric analysis, the Pt electrode was polished with a diamond suspension. In these conditions, when operating in CH_3CN (0.1 M $TEABF_4$), the formal potential for the ferrocene (+/0) couple was +0.40 V *vs* SCE.

Bulk electrolyzes

Bulk electrolyzes were performed in CH_3CN (0.1 M $TEABF_4$) at controlled potential in a cell with three compartments separated with glass frits of medium porosity with a Biologic SP-300 potentiostat, connected to an interfaced computer that employed EC-Lab (v. 11.43) software. Two platinum wire spirals (l = 50 cm, Ø = 1 mm for each spiral, $S_{tot} \approx 2\times15 \approx 30$ cm^2) were used as working electrodes, a Pt plate (*ca*. 30 cm^2) or spiral (*ca*. 15 cm^2) was used as the counter electrode and a saturated calomel electrode was used as the reference electrode. Electrolyzes were followed by TLC analyses and CV analyses and were stopped when the starting porphyrins were consumed.

Electrosynthesis of azolium-porphyrins

Electrosynthesis of Zn-2$^+$

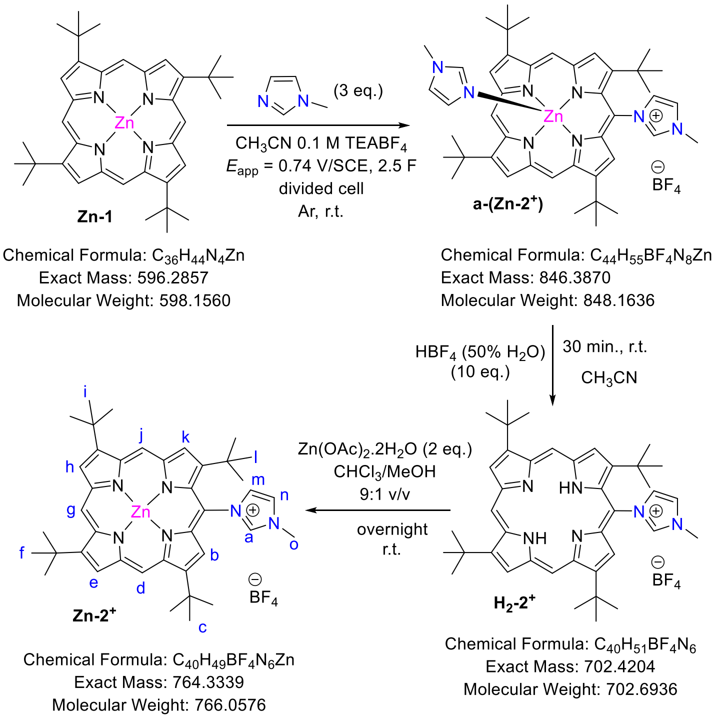

Zn-1 (200.00 mg, 3.34×10^{-1} mmol, 1 eq.) and methylimidazole (82 µL, 1.00 mmol, 3 eq.) were dissolved in CH_3CN (200 mL, 0.1 M $TEABF_4$). The electrolysis was carried out under an argon atmosphere under vigorous stirring at room temperature and at controlled potential (E_{app} = 0.74 V/SCE). Electrolysis was stopped after an uptake of 2.5 F *vs* **Zn-1** and the solvent was removed by rotary evaporation. The crude solid was dissolved in CH_2Cl_2 and washed three times (3×200 mL) with water to remove the supporting electrolyte. The product was purified by column chromatography (SiO_2, CH_2Cl_2 containing 5% CH_3OH). The fractions containing **a-(Zn-2$^+$)** were gathered and recrystallized in CH_2Cl_2/*n*-heptane. This porphyrin was dissolved in CH_3CN and HBF_4 (50% in H_2O, 423 µL, 3.37 mmol, 10.1 eq. *vs* **Zn-1**) to remove the zinc(II) metal (and the coordinated nucleophile). The mixture was stirred at room temperature for 30 min and the solvents were evaporated. The crude solid was dissolved in CH_2Cl_2 and washed three times (3×200 mL) with water, the solvent was then removed and the crude solid was dissolved in 20 mL of $CHCl_3/CH_3OH$ (9:1 v/v) and 2 eq. of $Zn(OAc)_2.2H_2O$ *vs* **Zn-1** (146.7 mg, 6.68×10^{-1} mmol)

were added. This solution was stirred overnight at room temperature, then the solvent was evaporated. The crude product was dissolved in CH_2Cl_2, then washed 3 times with H_2O. The product was recrystallized in CH_2Cl_2/*n*-heptane and dried at 110 °C for 6 h to give **Zn-2**$^+$ in 64% yield (m = 163.9 mg, 2.14×10^{-1} mmol).

^{1}H NMR ($(CD_3)_2SO$, 500 MHz, 298 K): δ (ppm): 10.66 (s, 1H, Hj), 10.61 (s, 1H, Hg), 10.60 (s, 1H, Hd), 10.30 (brs, 1H, Ha), 9.73 (s, 1H, Hk), 9.47 (s, 1H, Hh), 9.41 (s, 1H, He), 9.40 (brs, 1H, Hm), 8.43 (brs, 1H, Hn), 7.84 (s, 1H, Hb), 4.29 (s, 3H, Ho), 2.29 (s, 9H, Hi), 2.27 (s, 9H, Hf), 2.17 (s, 9H, Hc), 1.67 (s, 1H, Hl).

^{13}C{^{1}H} NMR ($(CD_3)_2SO$, 500 MHz, 298 K): 155.5, 155.3, 154.7, 152.1, 148.3, 148.1, 147.0, 146.9, 146.7, 145.9, 145.5, 144.9, 144.3, 135.4, 132.8, 130.4, 129.77, 124.0, 121.1, 107.6, 105.7, 105.5, 105.4, 36.4, 33.9, 33.8, 33.7, 33.7, 33.5, 33.5, 33.4, 33.2.

λ_{max} (DMSO)/ nm (log ε): 415 (5.51), 544 (4.15), 582 (4.15).

HRMS (ESI+): m/z cald for $C_{40}H_{49}N_6Zn^+$ $[M-BF_4^-]^+$ 677.3306, found 677.3300.

Characterization of Zn-2$^+$

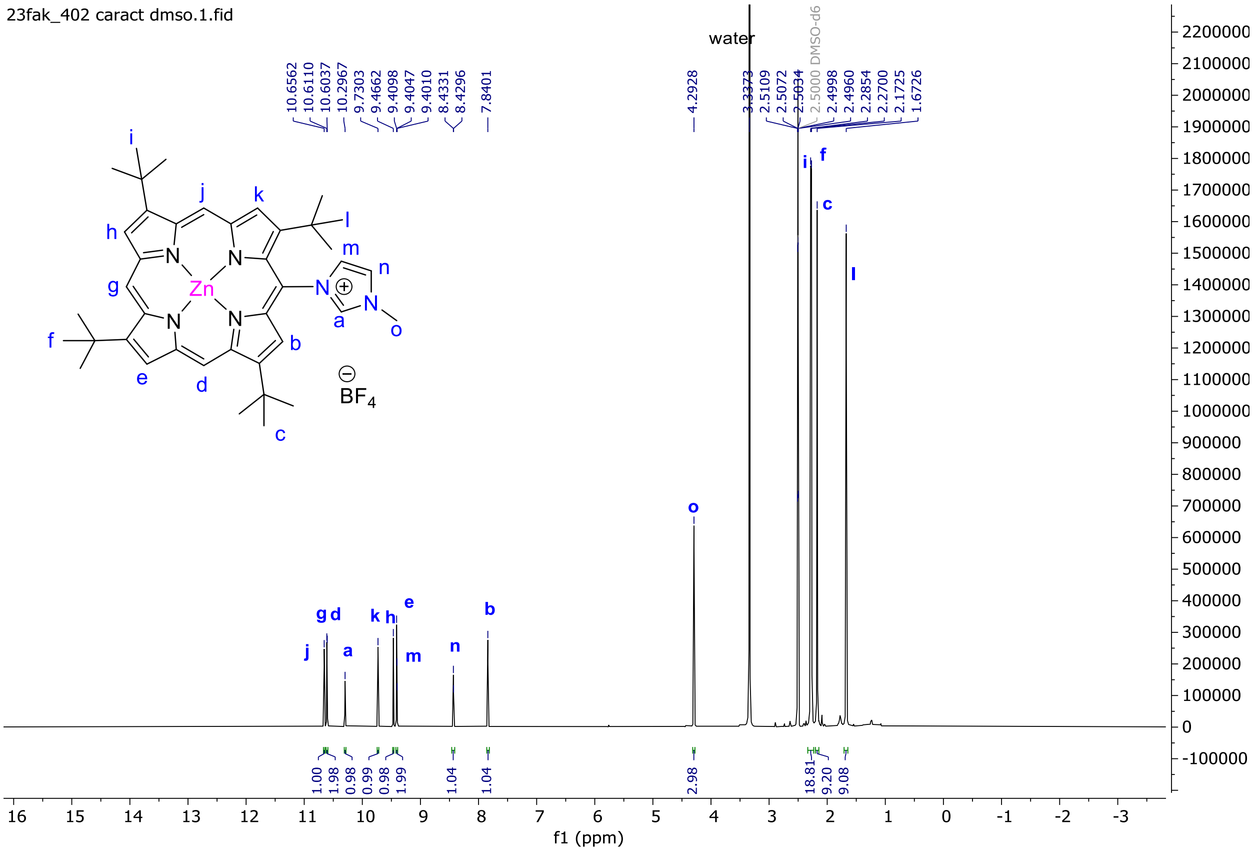

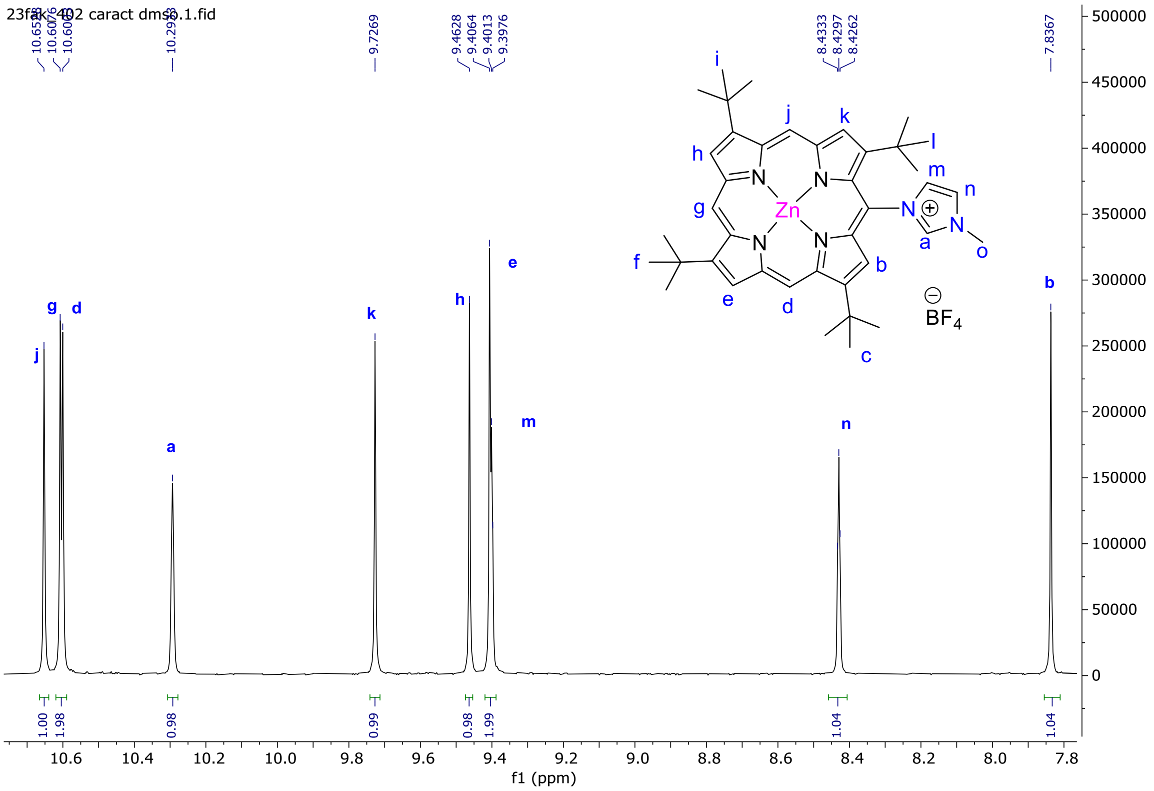

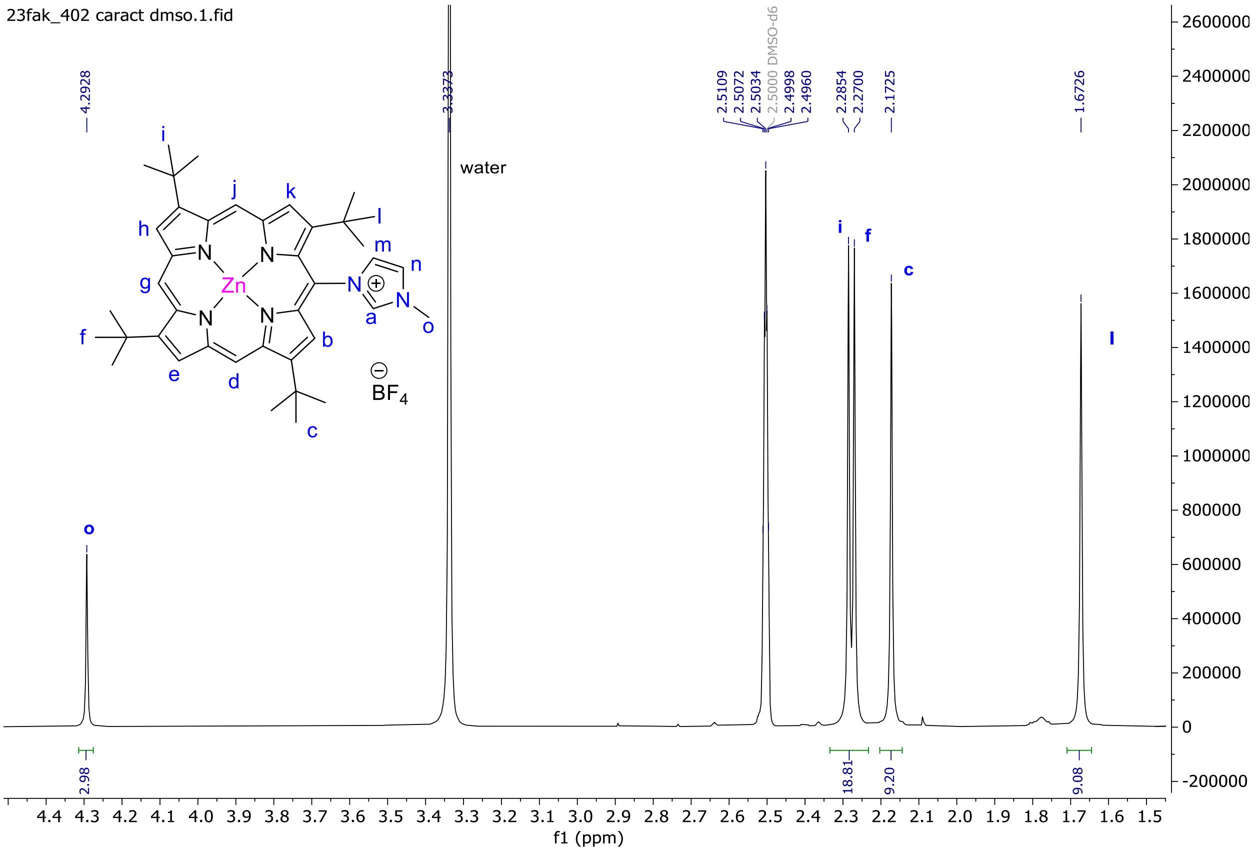

Figure S2. Full range (top) and partial (middle and bottom) ^{1}H NMR spectra of **Zn-2^{+}** in $(CD_3)_2SO$, 500 MHz, 298 K.

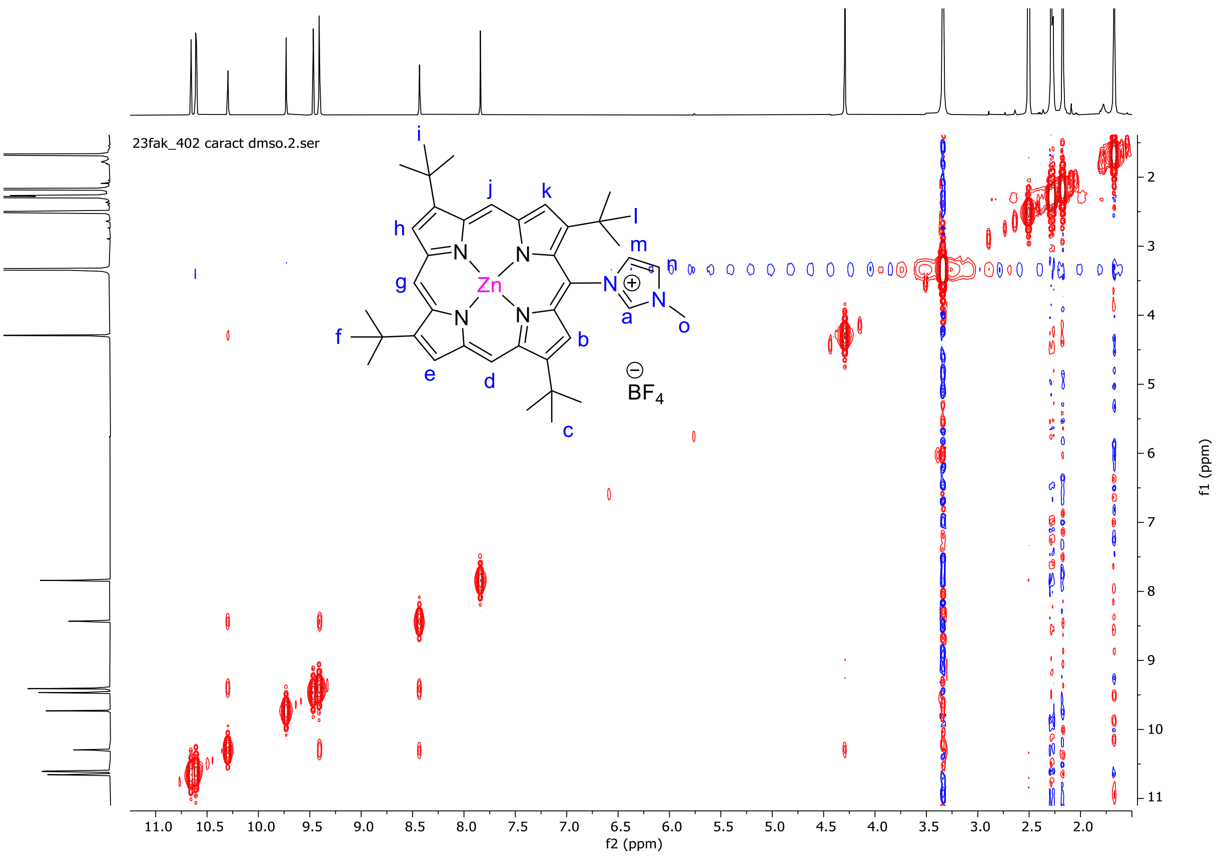

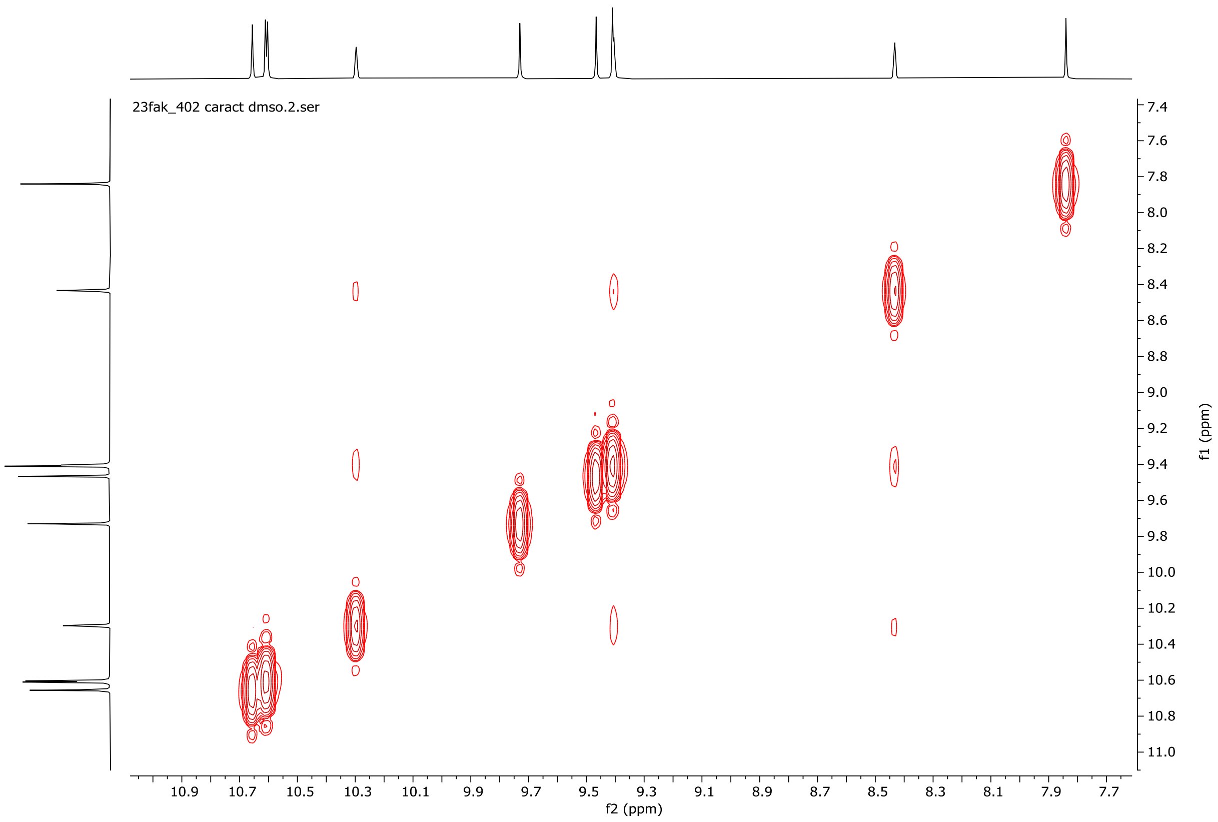

Figure S3. Full range (top) and partial (bottom) ^{1}H-^{1}H COSY spectra of **Zn-2**$^+$ in $(CD_3)_2SO$, 500 MHz, 298 K.

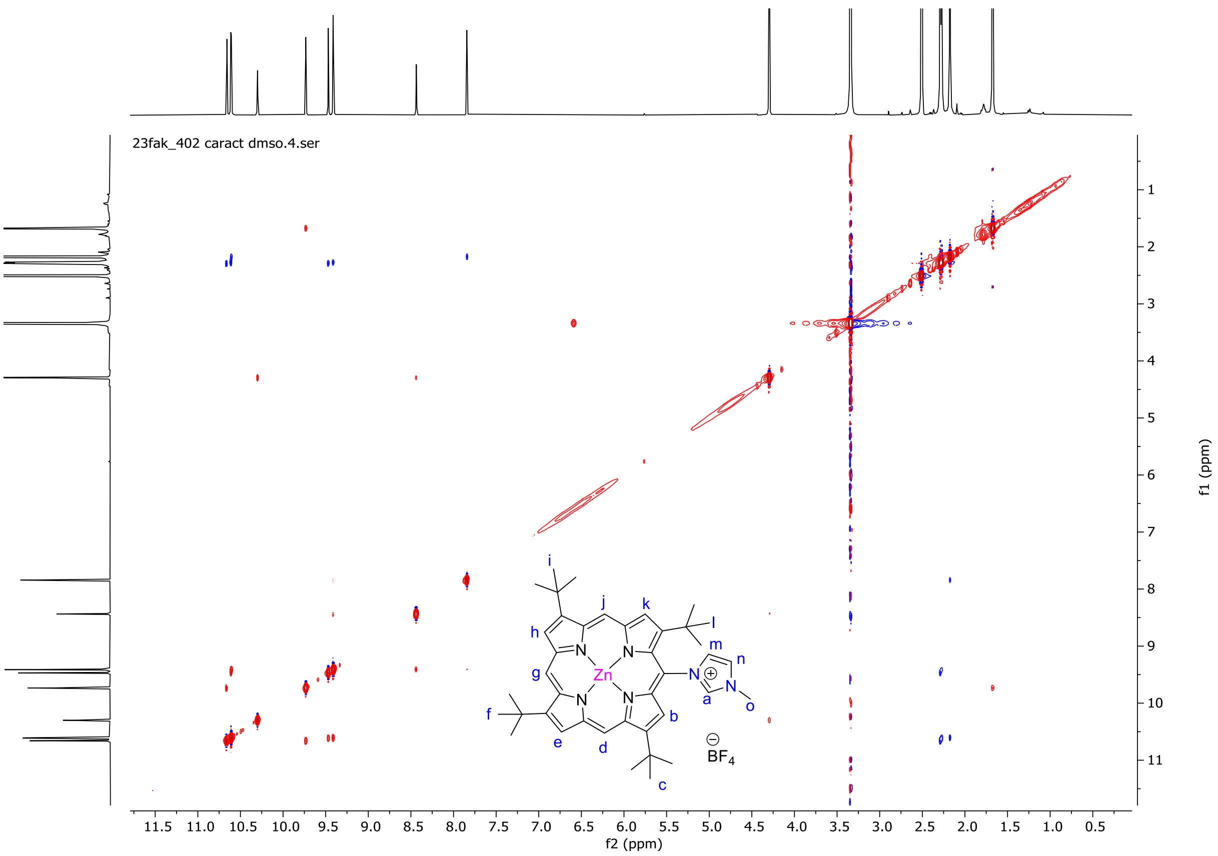

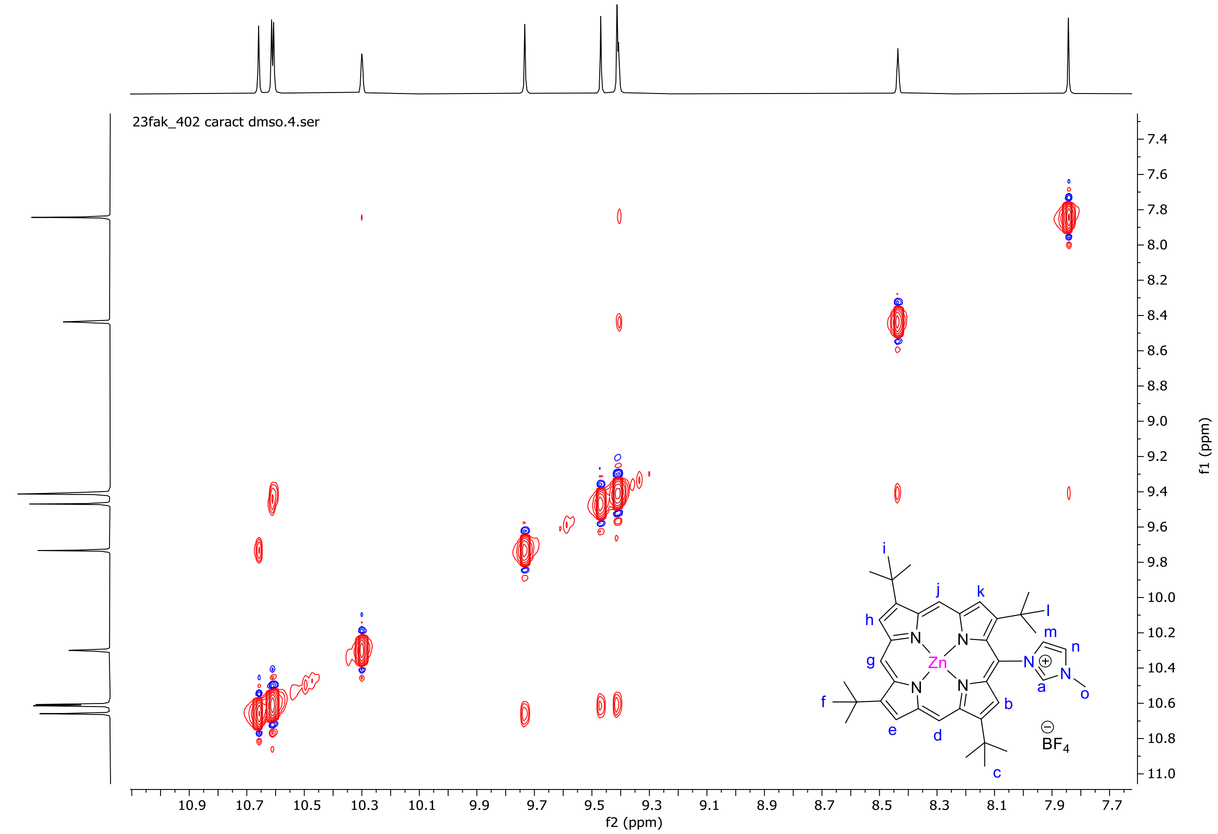

Figure S4. Full range (top) and partial (bottom) ^{1}H-^{1}H NOESY spectra of **Zn-2⁺** in $(CD_3)_2SO$, 500 MHz, 298 K.

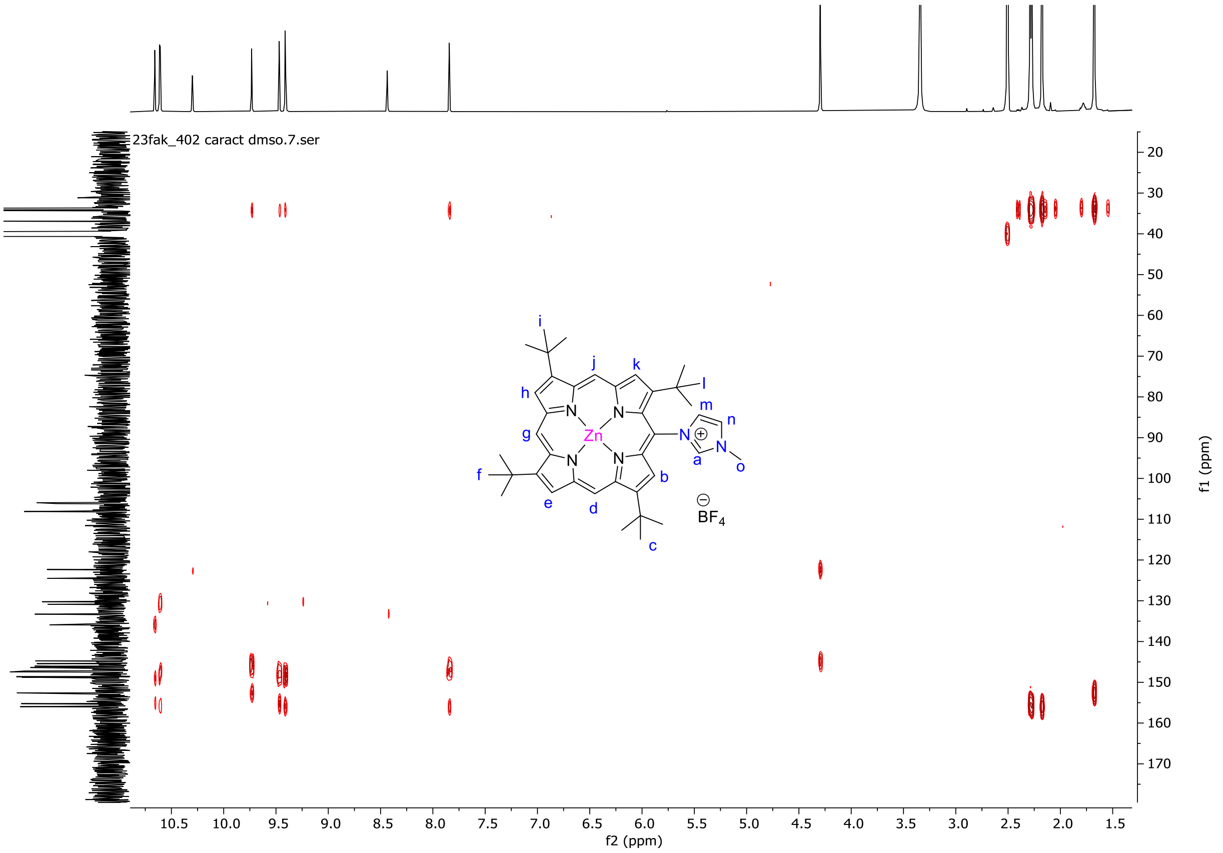

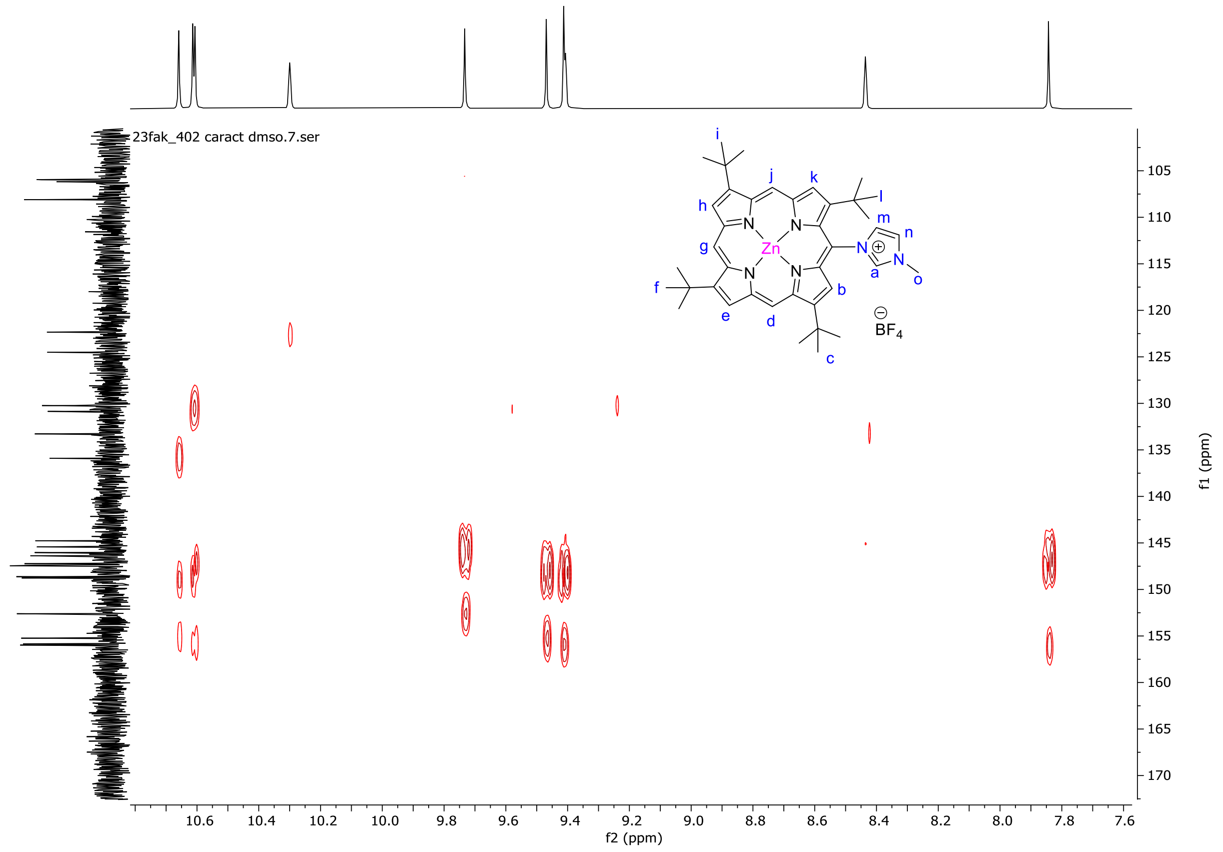

Figure S5. Full range (top) and partial (bottom) ^{1}H-^{13}C HMBC spectra of **Zn-2**$^{+}$ in $(CD_3)_2SO$, 500 MHz, 298 K.

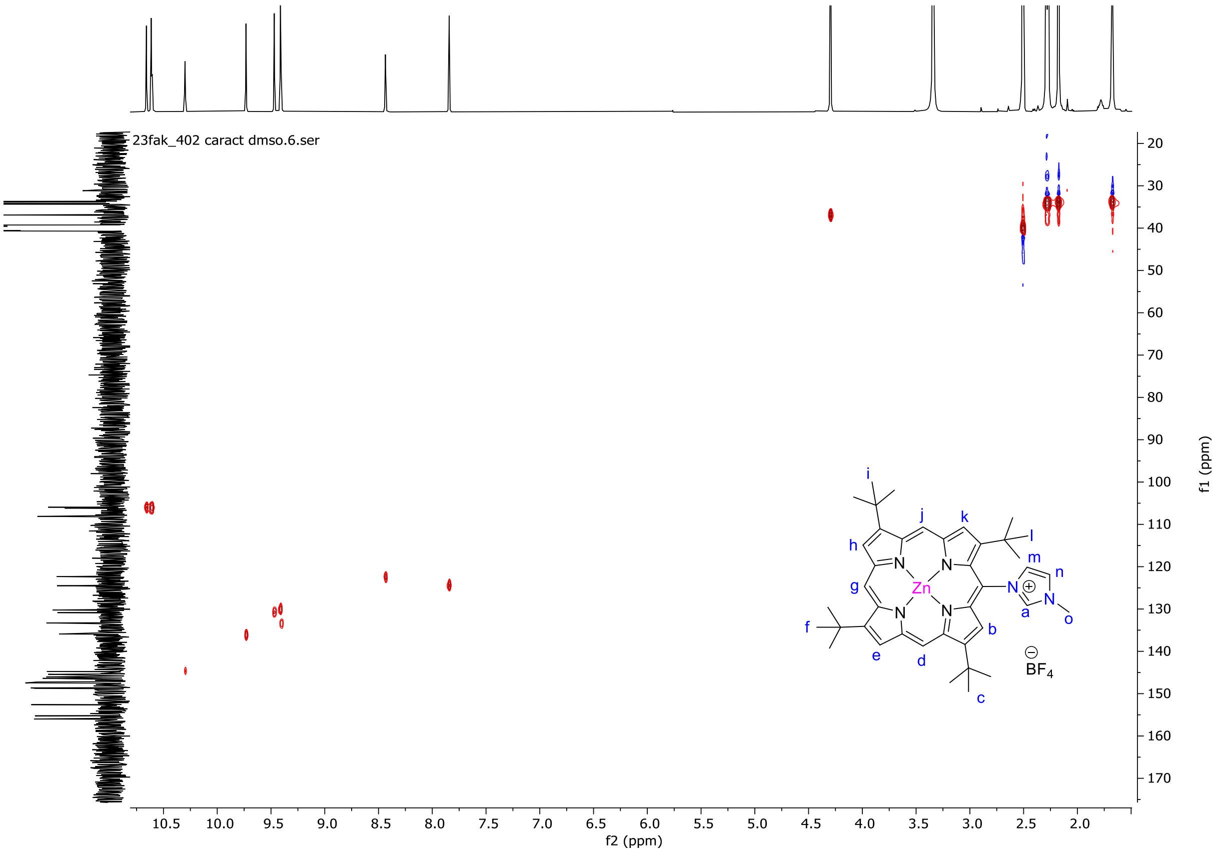

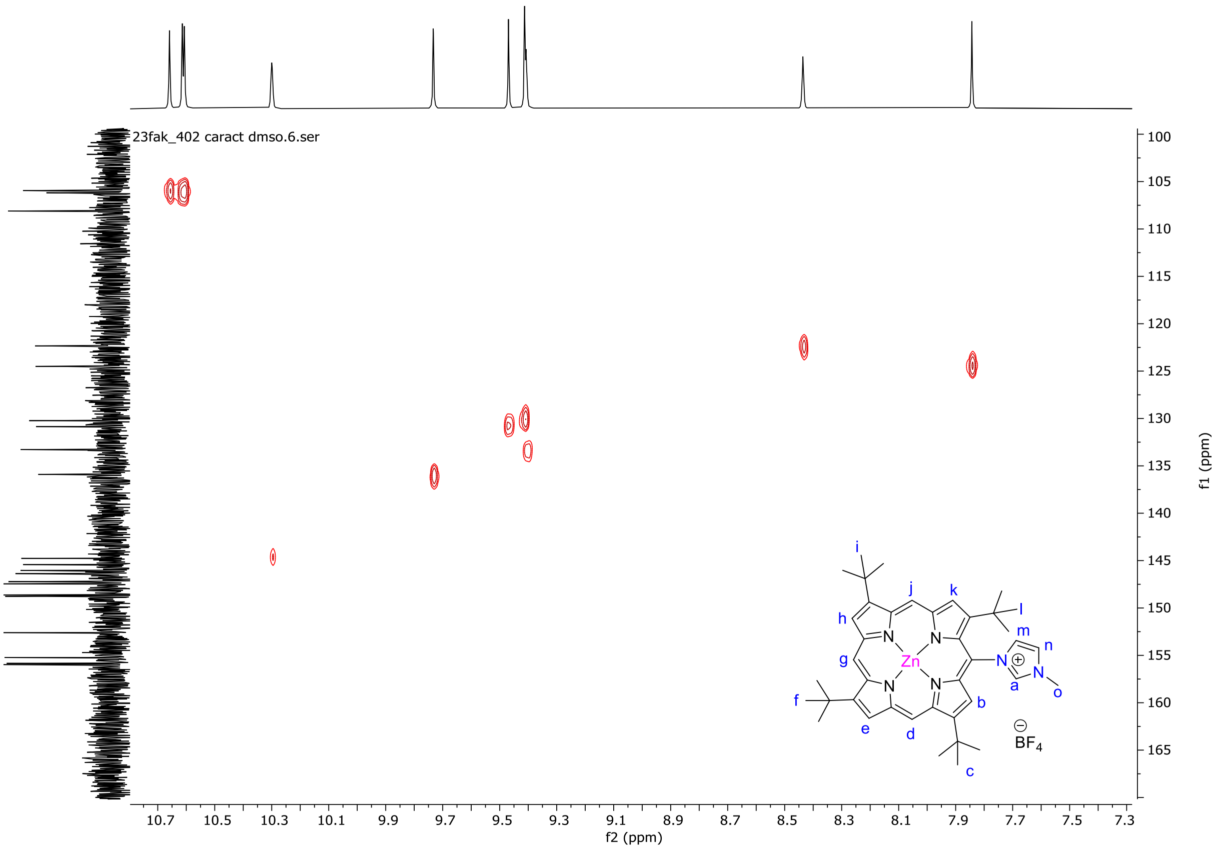

Figure S6. Full range (top) and partial (bottom) ^{1}H-^{13}C HSQC spectra of **Zn-2^{+}** in $(CD_3)_2SO$, 500 MHz, 298 K.

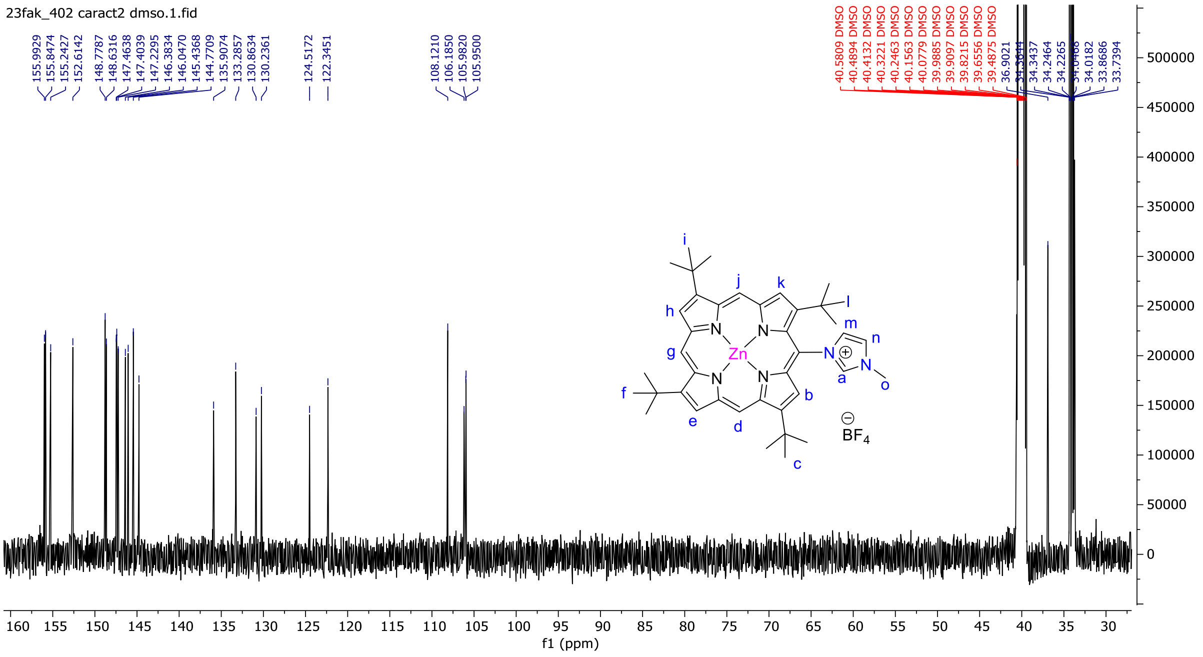

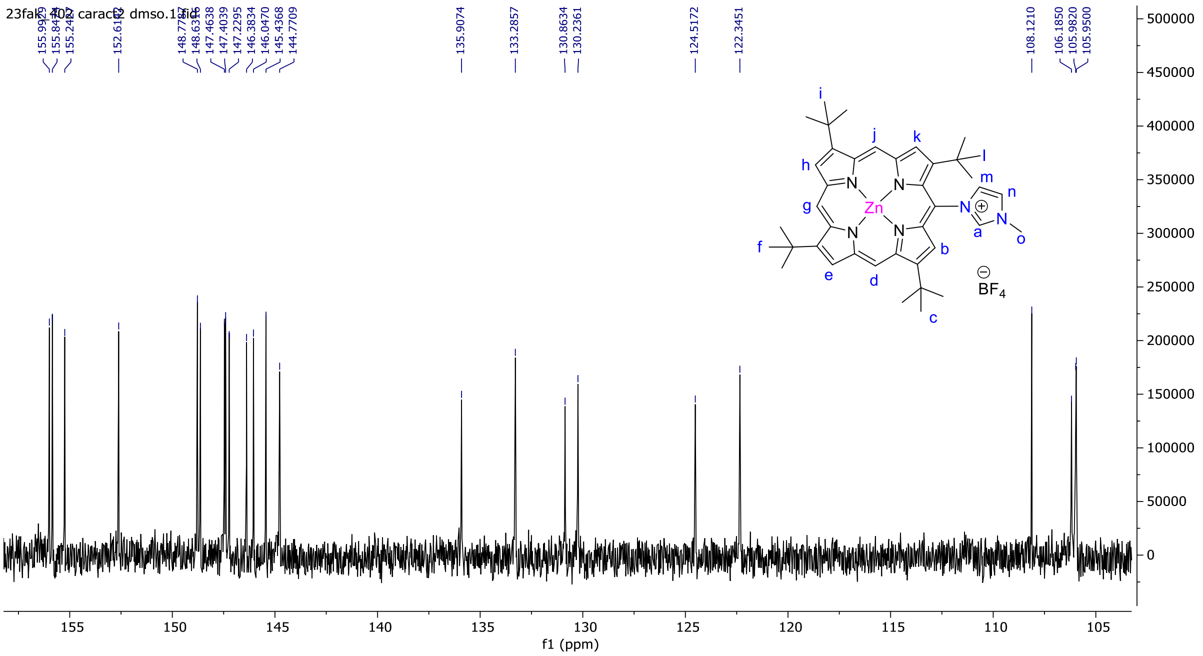

Figure S7. Full range (top) and partial (bottom) ^{13}C spectra of **Zn-2$^+$** in $(CD_3)_2SO$, 125 MHz, 298 K.

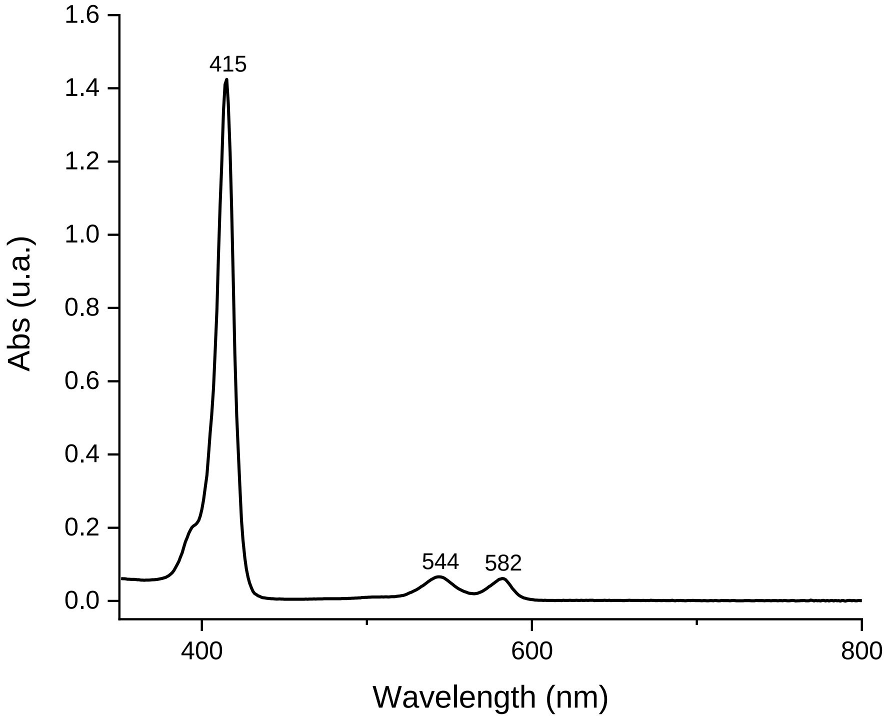

Figure S8. UV-Vis. absorption spectrum of **Zn-2**$^+$ in DMSO, room temperature.

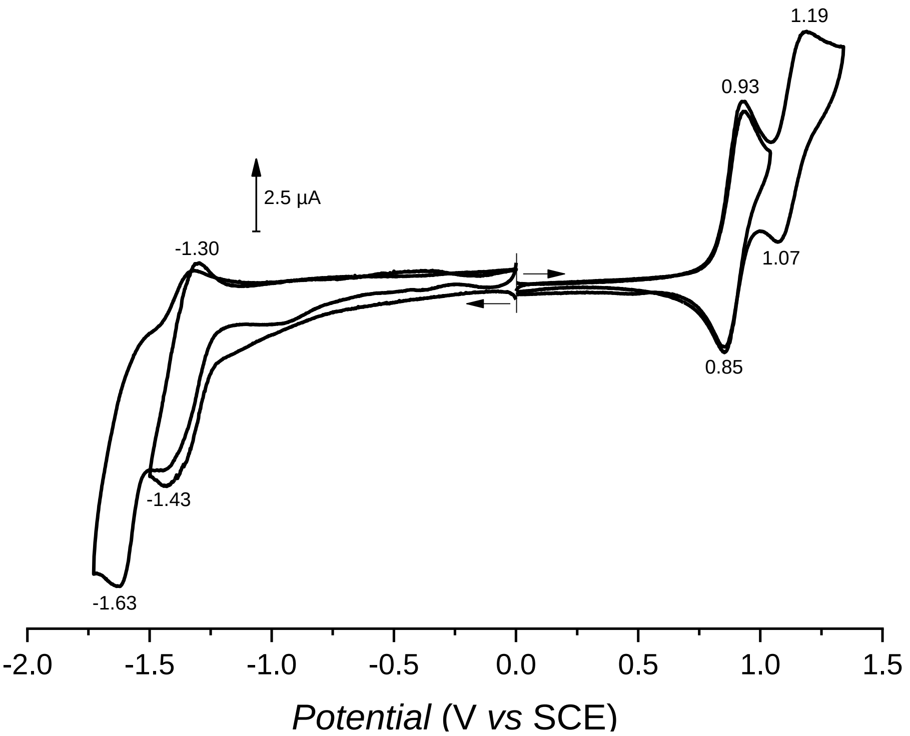

Figure S9. Cyclic voltammogram of compound **Zn-2**$^+$ (C = 10^{-3} M in CH_3CN 0.1 M $TEABF_4$, v = 100 $mV.s^{-1}$, WE: Pt, Ø = 1.6 mm, CE: Pt, RE: SCE, IUPAC convention).

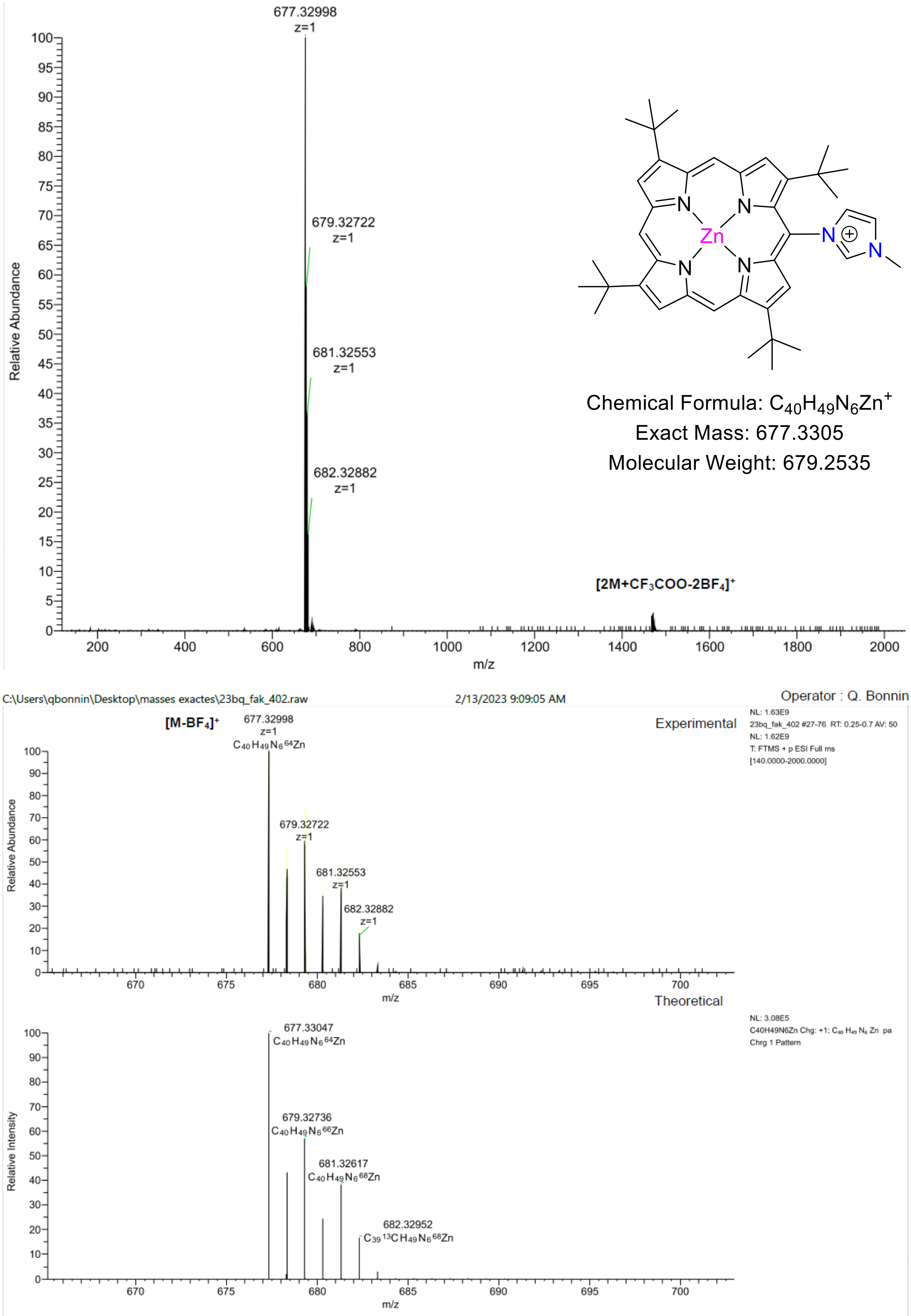

Display Formula	Peak Mass	Theo. mass	Delta [ppm]	RDB	Rank	MSMS Matched Fragments
$C_{40}H_{49}N_6{}^{64}Zn$	677.32998	677.33047	-0.72	19.50	1	(Collection)

Figure S10. High resolution ESI mass spectrum of **Zn-2**$^+$ and simulation of its isotopic pattern.

Electrosynthesis of Zn-3$^+$

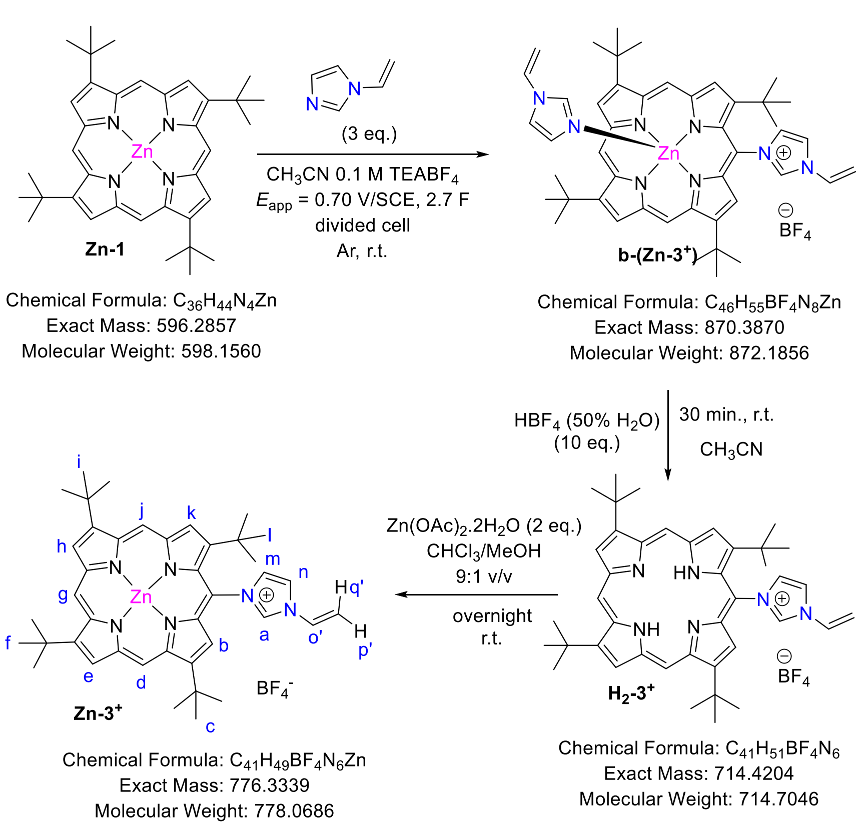

Zn-1 (30.0 mg, 5.01×10^{-2} mmol, 1 eq.), and 1-vinylimidazole (45 µL, 1.50×10^{-1} mmol, 3 eq.) were dissolved in CH_3CN (200 mL, 0.1 M $TEABF_4$). The electrolysis was carried out under an argon atmosphere under vigorous stirring at room temperature and at controlled potential (E_{app} = 0.70 V/SCE). Electrolysis was stopped after an uptake of 2.7 F *vs* **Zn-1** and the solvent was removed by rotary evaporation. The crude solid was dissolved in CH_2Cl_2 and washed three times (3×200 mL) with water to remove the supporting electrolyte. The product was purified by column chromatography (SiO_2, CH_2Cl_2 containing 3% CH_3OH). The fractions containing **b-(Zn-3$^+$)** were gathered and recrystallized in CH_2Cl_2/*n*-heptane. This porphyrin was then dissolved in CH_3CN and HBF_4 (50% in H_2O, 60 µL, 4.81 mmol, 10 eq. *vs* **b-(Zn-3$^+$)**) was added to remove the zinc(II) metal (and the coordinated nucleophile). The mixture was stirred at room temperature for 30 min. and the solvents were evaporated. The crude solid was dissolved in CH_2Cl_2 and washed three times (3×50 mL) with water. The solvent was then removed and the crude solid was dissolved in 4 mL of $CHCl_3/CH_3OH$ (9:1 v/v) and 2.0 eq. of $Zn(OAc)_2.2H_2O$ *vs* **Zn-1** (22.1 mg, 1.003×10^{-1} mmol) were added. This solution was stirred overnight at room temperature, then the solvent was evaporated. The crude product was dissolved in CH_2Cl_2, then washed 3 times with H_2O. The product was recrystallized in CH_2Cl_2/*n*-heptane and dried at 110 °C for 6 h to give **Zn-3$^+$** in 75% yield (29.0 mg, 3.79×10^{-2} mmol).

^{1}H NMR ($(CD_3)_2SO$, 500 MHz, 298 K): δ (ppm): 10.72 (s, 1H, Ha), 10.67 (s, 1H, Hj), 10.62 (s, 1H, Hg), 10.61 (s, 1H, Hd), 9.74 (s, 1H, Hk), 9.60 (brs, 1H, Hm), 9.47 (s, 1H, Hh), 9.41(s,

1H, He), 8.91 (brs, 1H, Hn), 7.93 (s, 1H, Hb), 7.62 (dd, $^3J_{H-H}$ = 15.0 Hz, $^3J_{H-H}$ = 8.7 Hz, 1H, Ho’), 6.36 (dd, $^3J_{H-H}$ = 15.0 Hz, $^2J_{H-H}$ = 2.4 Hz, 1H, Hq’), 5.76 (dd, $^3J_{H-H}$ = 8.7 Hz, $^2J_{H-H}$ = 2.4 Hz, 1H, Hp’), 2.28 (s, 9H, Hi), 2.27 (s, 9H, Hf), 2.17 (s, 9H, Hi), 1.67 (s, 9H, Hl).

^{13}C{^{1}H} NMR ($(CD_3)_2SO$, 125 MHz, 298 K): 155.7, 155.5, 154.8, 151.9, 148.3, 148.2, 147.1, 147.0, 146.8, 145.2, 145.2, 144.8, 142.4, 135.5, 133.3, 130.4, 129.8, 128.9, 124.0, 117.7, 110.9, 107.3, 105.8, 105.6, 33.92, 33.90, 33.8, 33.7, 33.6, 33.5, 33.5, 33.3, 33.2.

λ_{max} (DMSO)/ nm (log ε): 415 (5.41), 543 (4.09), 581 (4.07).

HRMS (ESI+): m/z cald for $C_{41}H_{49}BF_4N_6Zn^+$ [M-BF_4^-] 698.3339, found 689.3298.

Characterization of Zn-3⁺

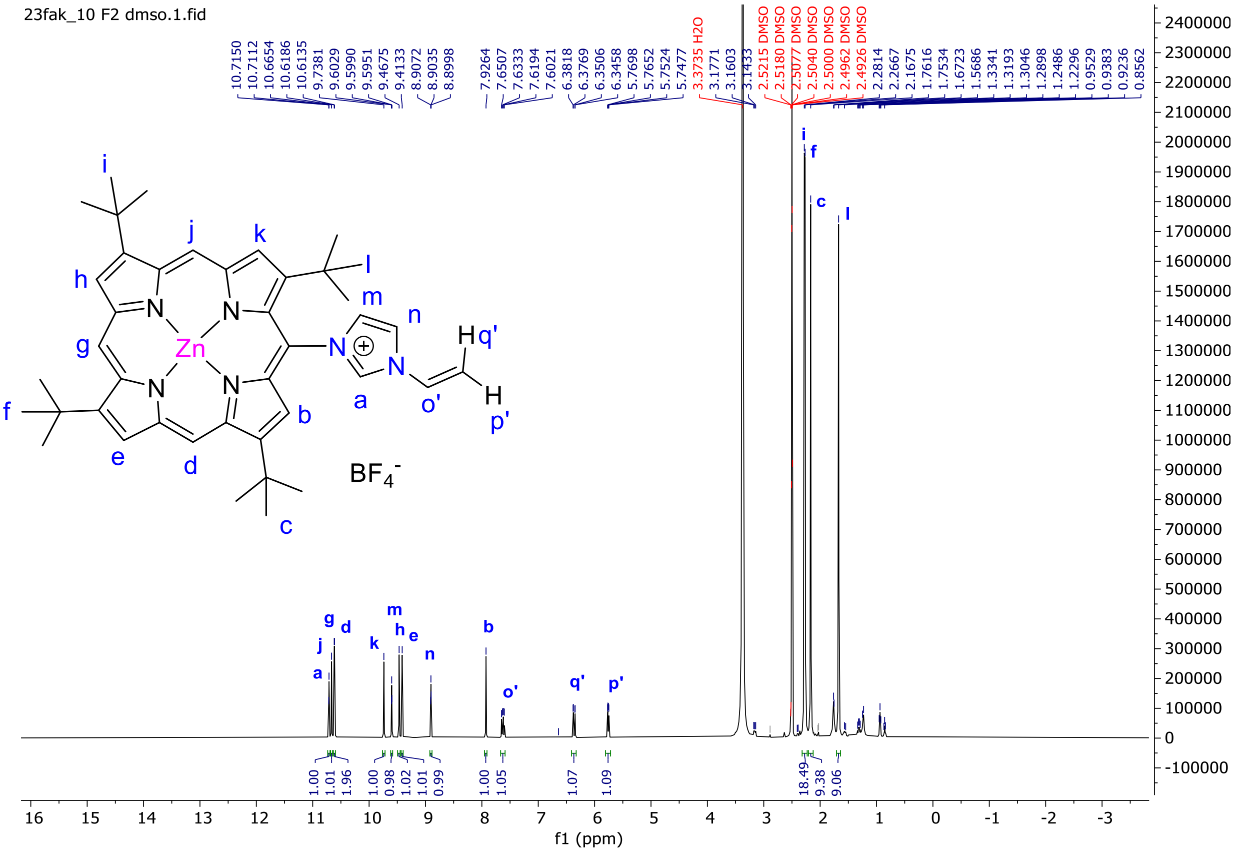

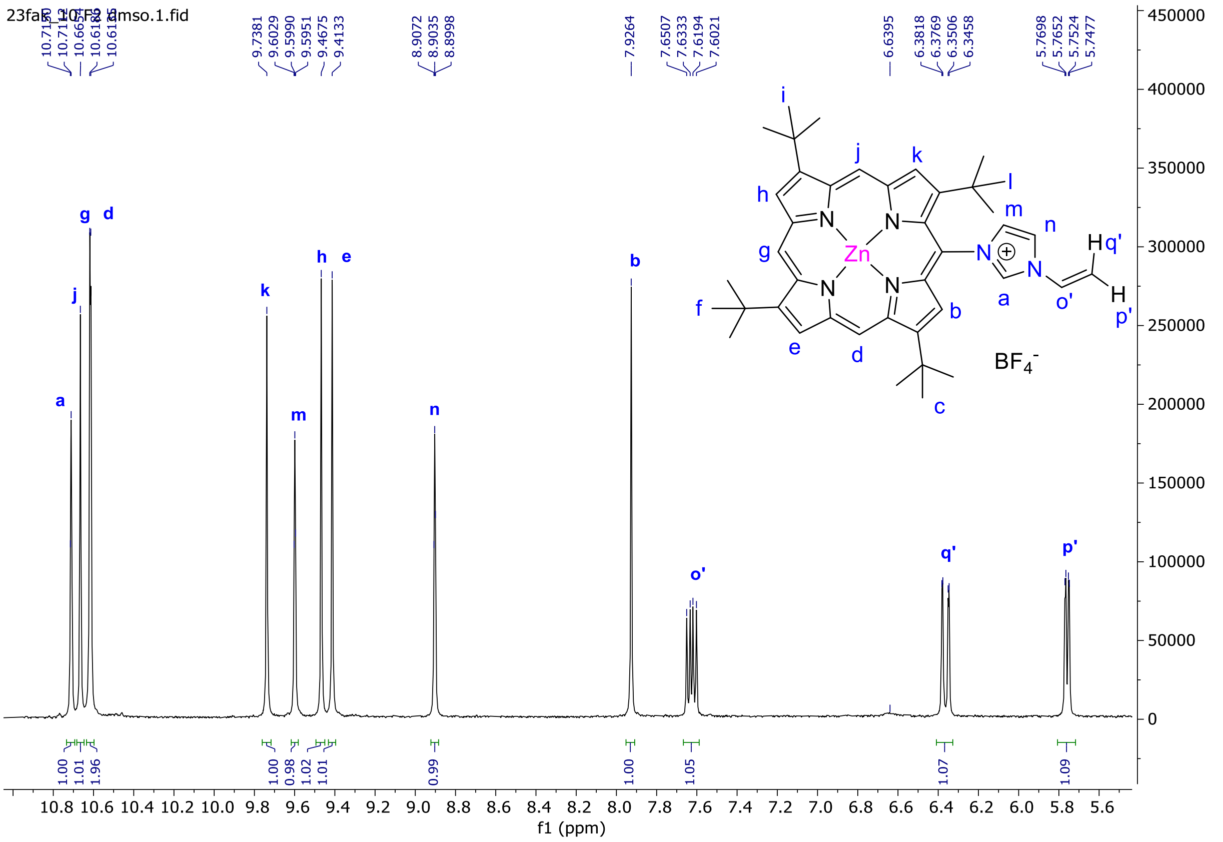

Figure S11. Full range (top) and partial (bottom) ^{1}H NMR spectra of **Zn-3⁺** in $(CD_3)_2SO$, 500 MHz, 298 K.

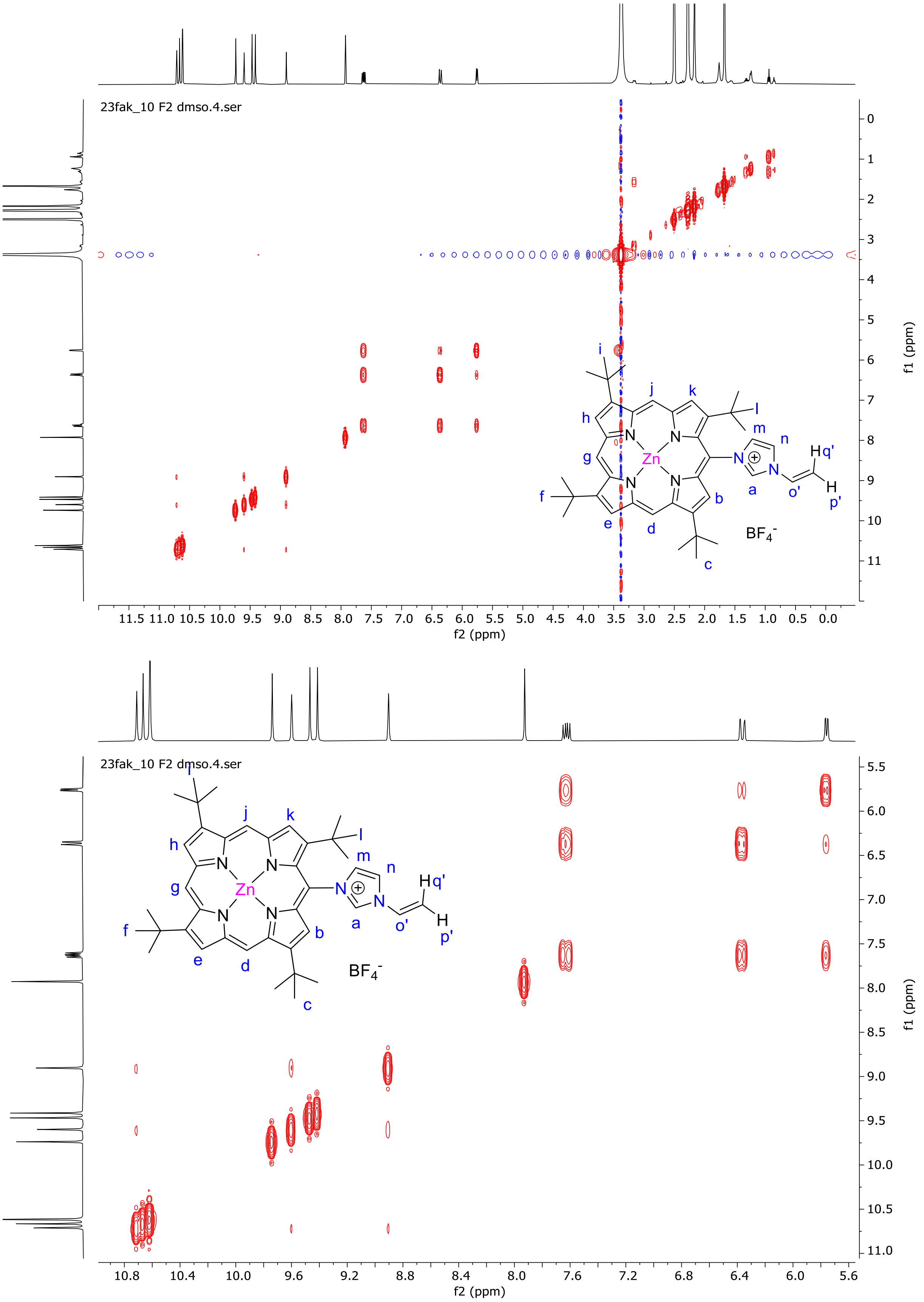

Figure S12. Full range (top) and partial (bottom) ^1H-^1H COSY spectra of **Zn-3$^+$** in $(CD_3)_2SO$, 500 MHz, 298 K.

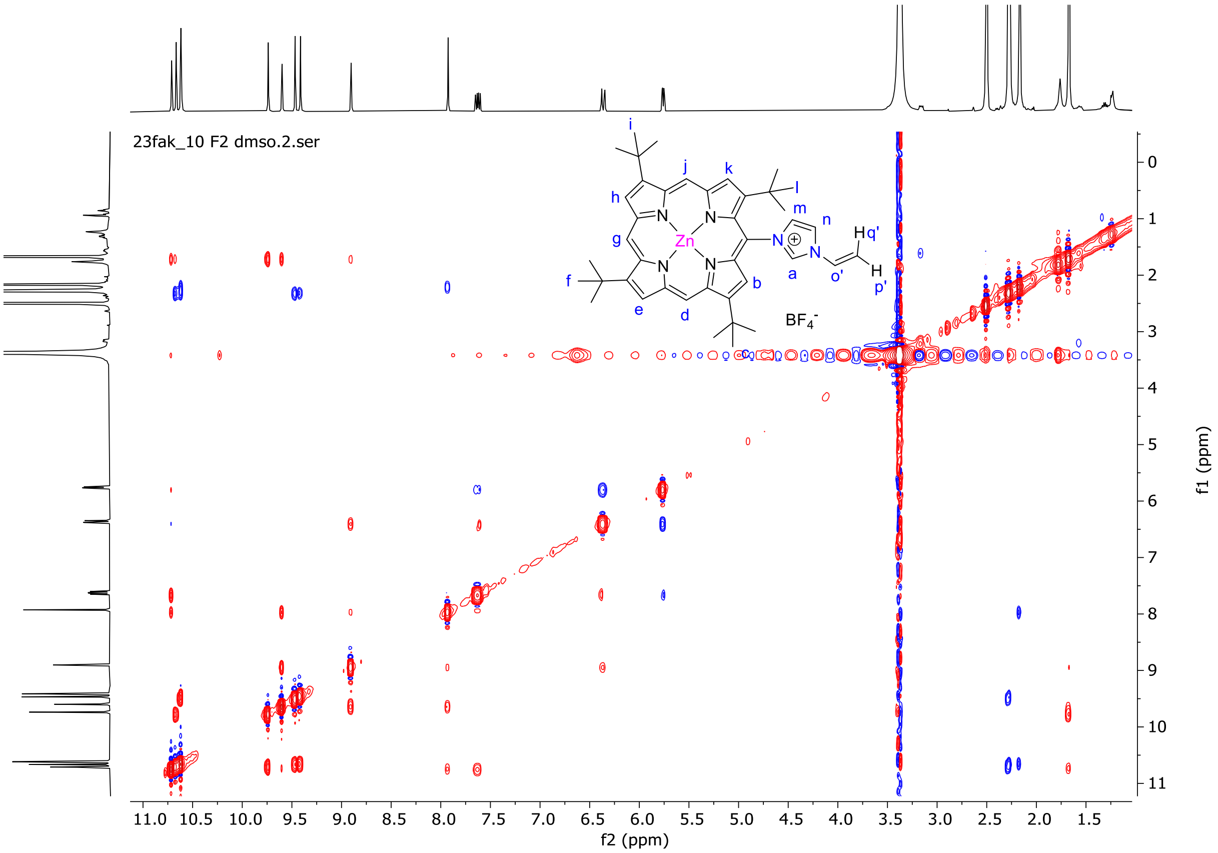

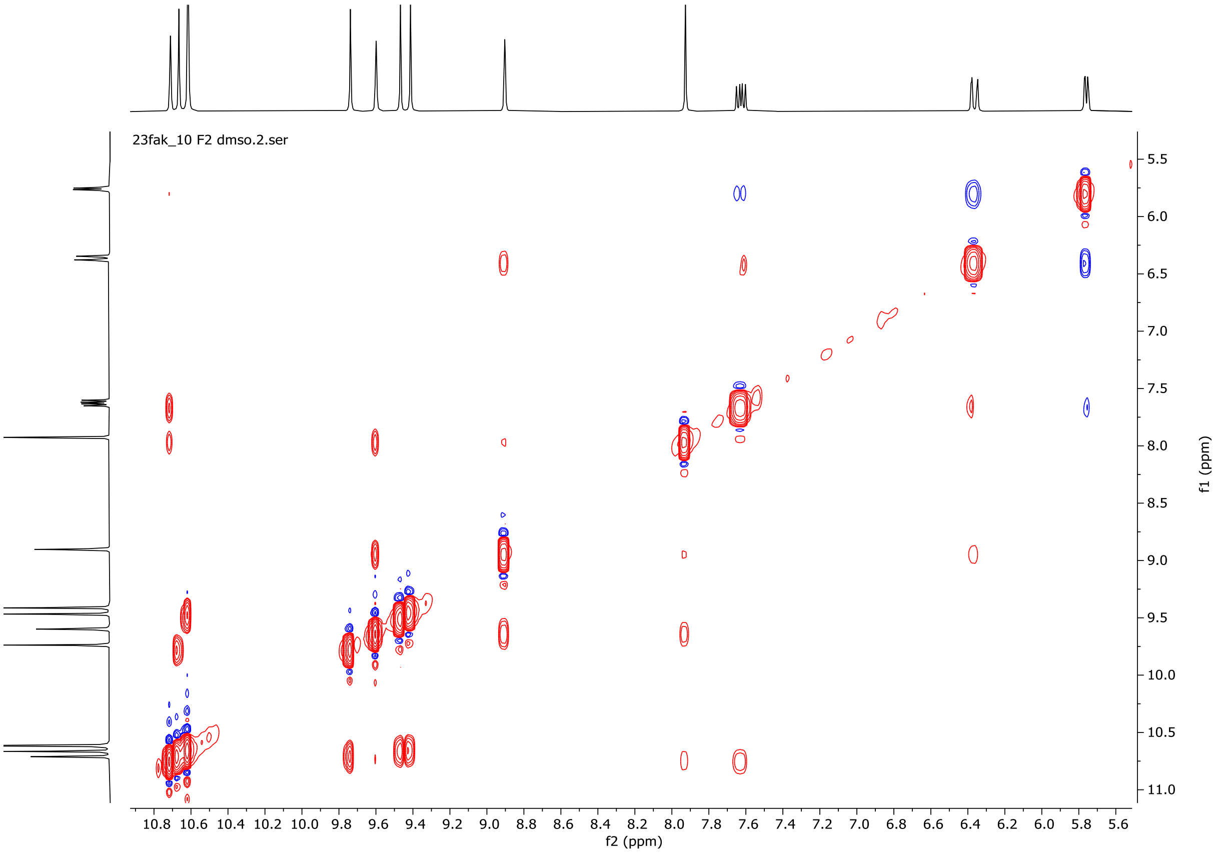

Figure S13. Full range (top) and partial (bottom) ^{1}H-^{1}H NOESY spectra of **Zn-3**$^+$ in $(CD_3)_2SO$, 500 MHz, 298 K

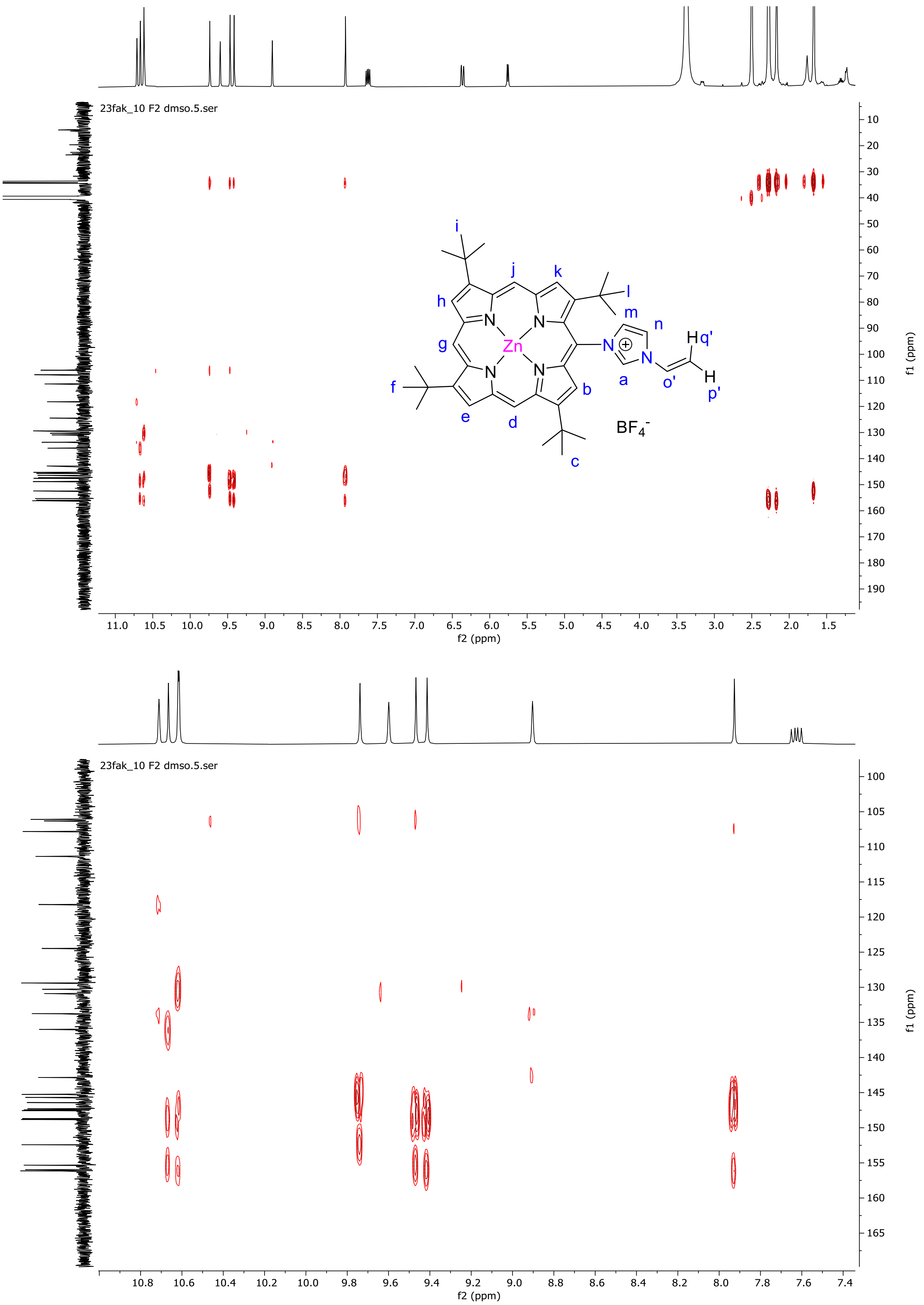

Figure S14. Full range (top) and partial (bottom) ^{1}H-^{13}C HMBC spectra of **Zn-3⁺** in $(CD_3)_2SO$, 500 MHz, 298 K

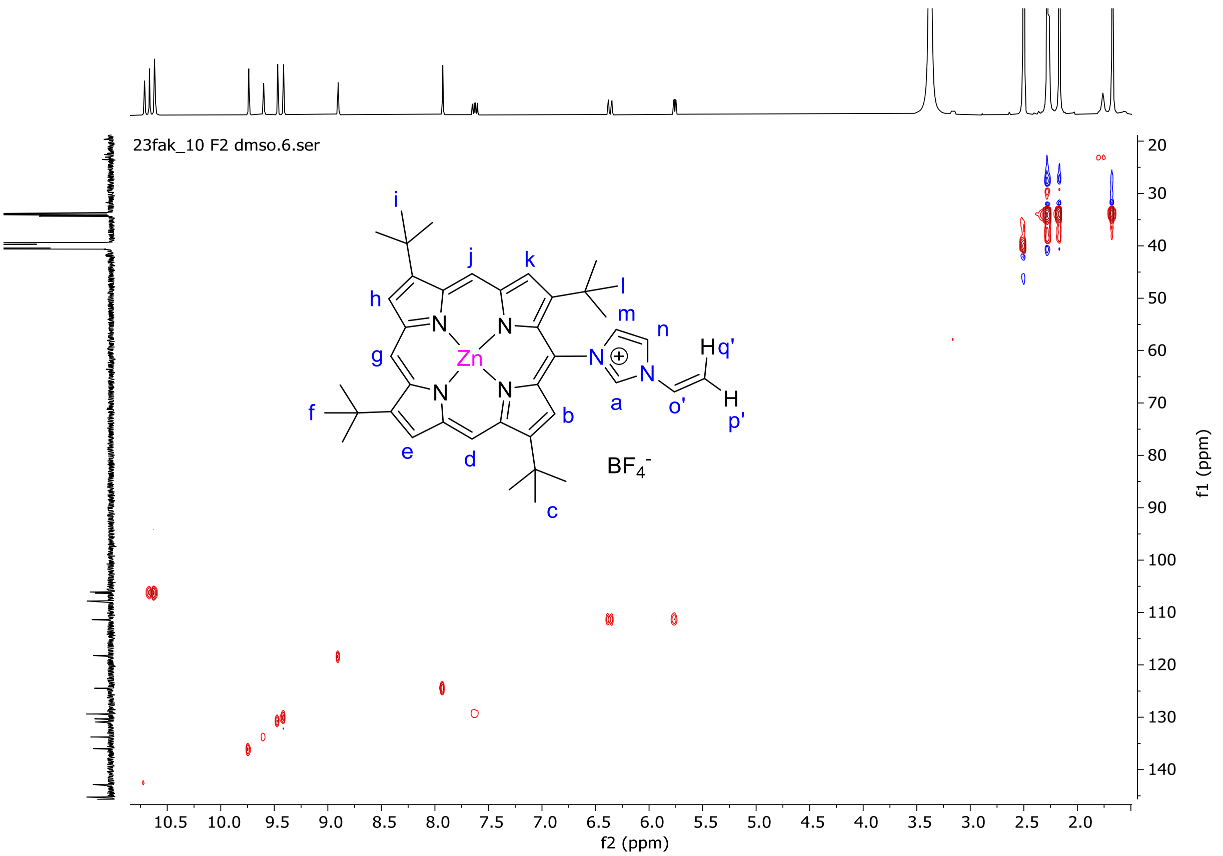

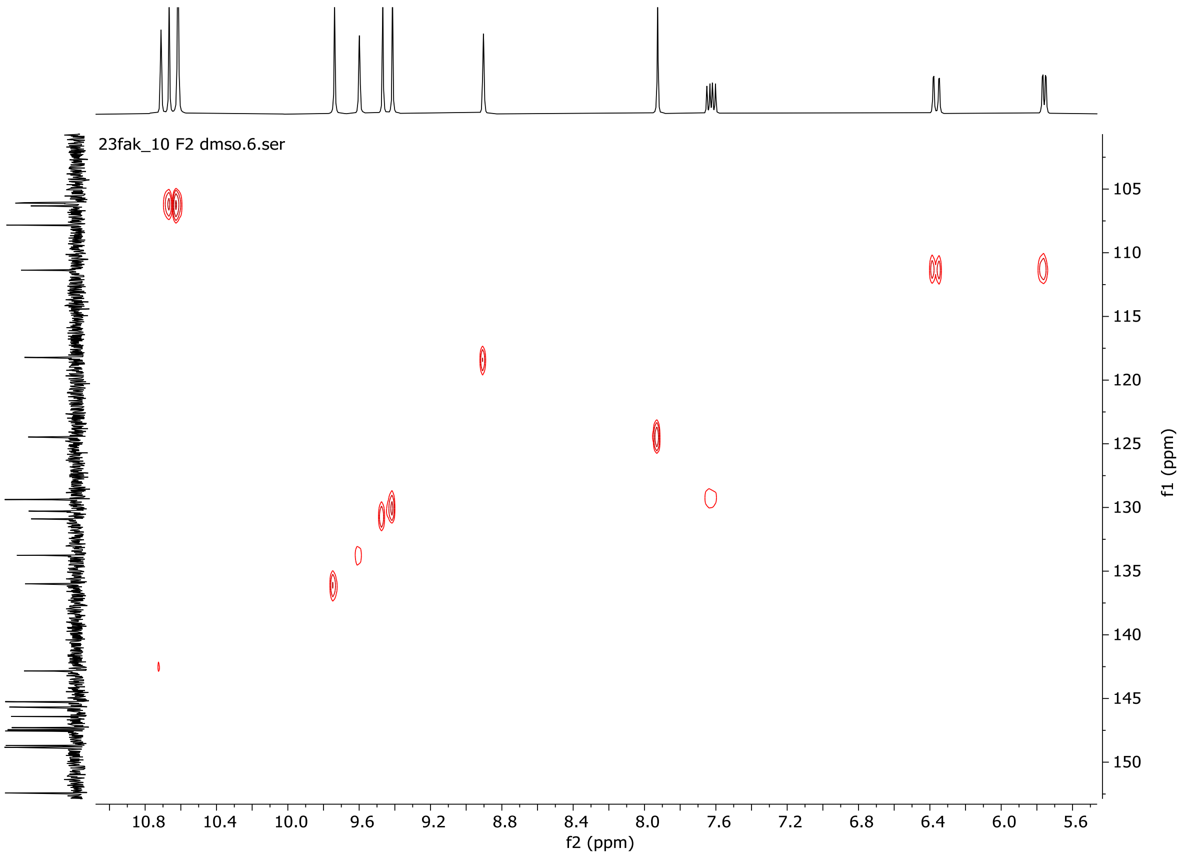

Figure S15. Full range (top) and partial (bottom) ^{1}H-^{13}C HSQC spectra of **Zn-3^{+}** in $(CD_3)_2SO$, 500 MHz, 298 K.

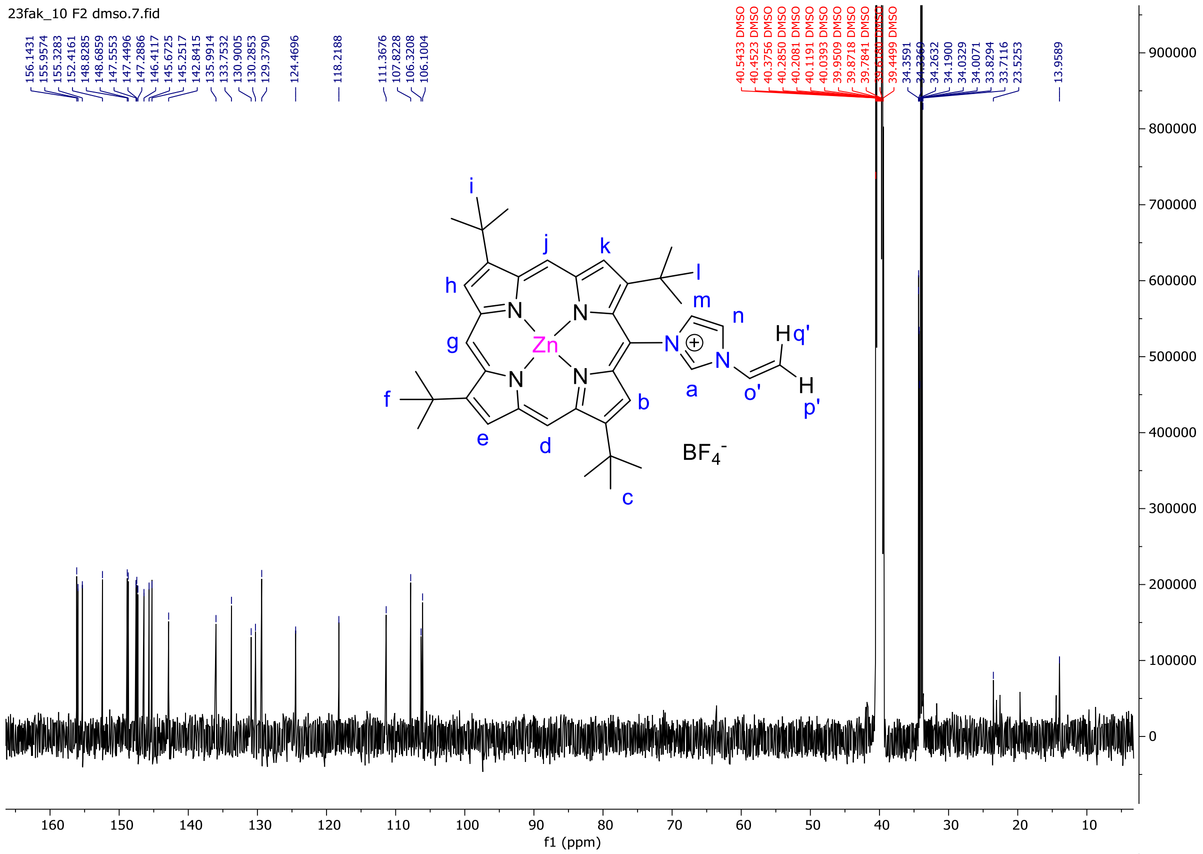
23fak_10 F2 dmso.7.fid
BF4-
f1 (ppm)

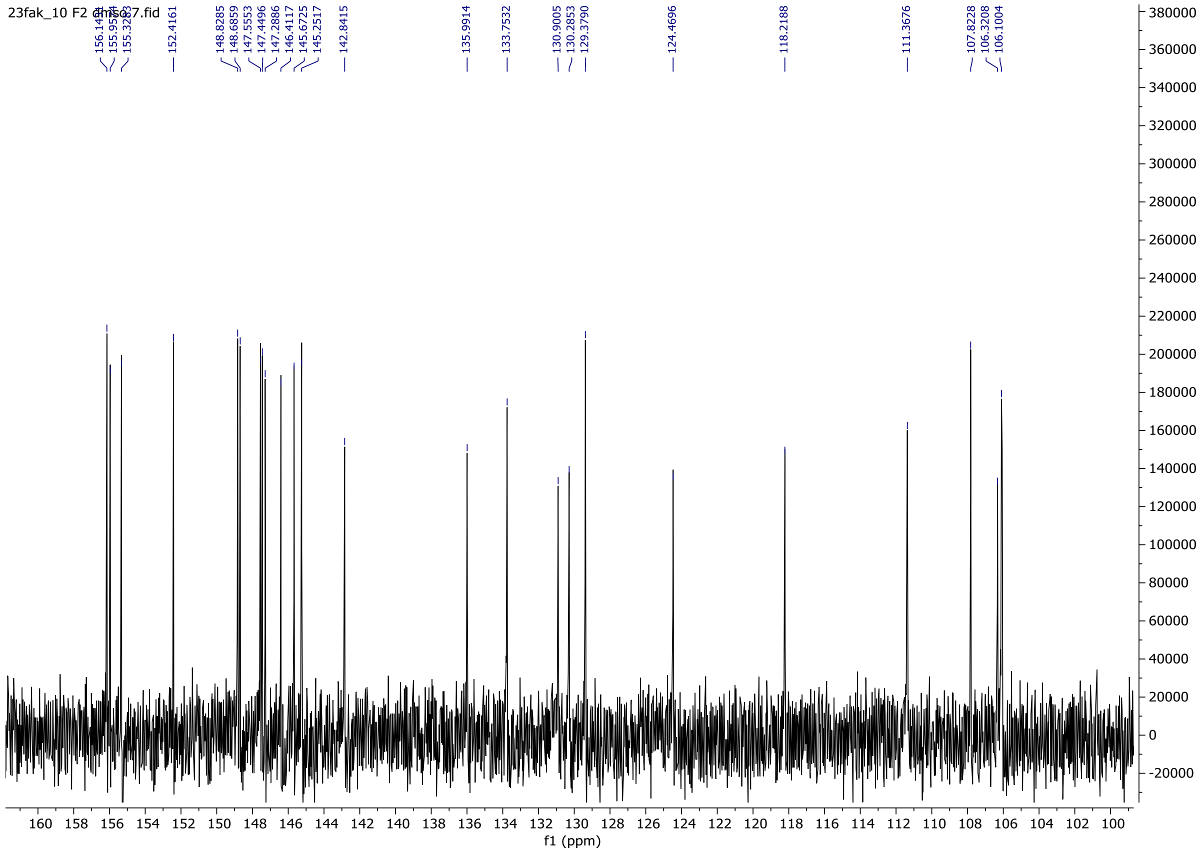
23fak_10 F2 dmso.7.fid
f1 (ppm)

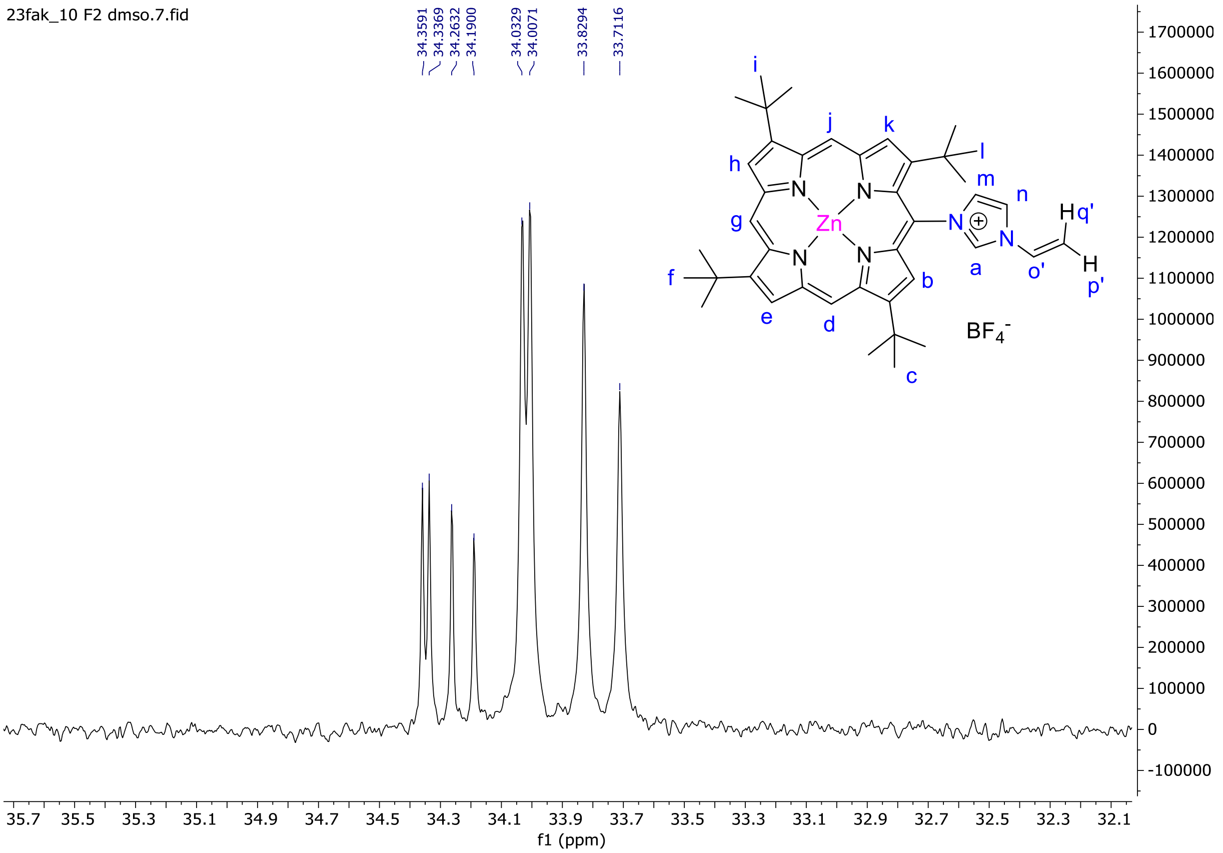

Figure S16. Full range (top) and partial (middle and bottom) ^{13}C spectra of **Zn-3**$^{+}$ in $(CD_3)_2SO$, 500 MHz, 298 K.

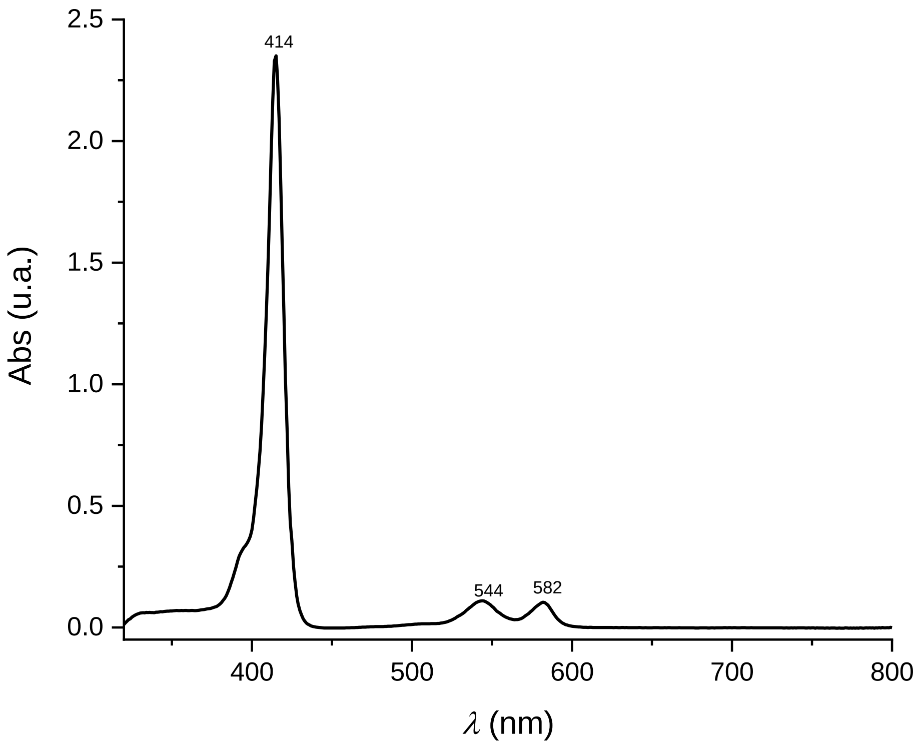

Figure S17. UV-Vis. absorption spectrum of **Zn-3**$^{+}$ in DMSO, room temperature.

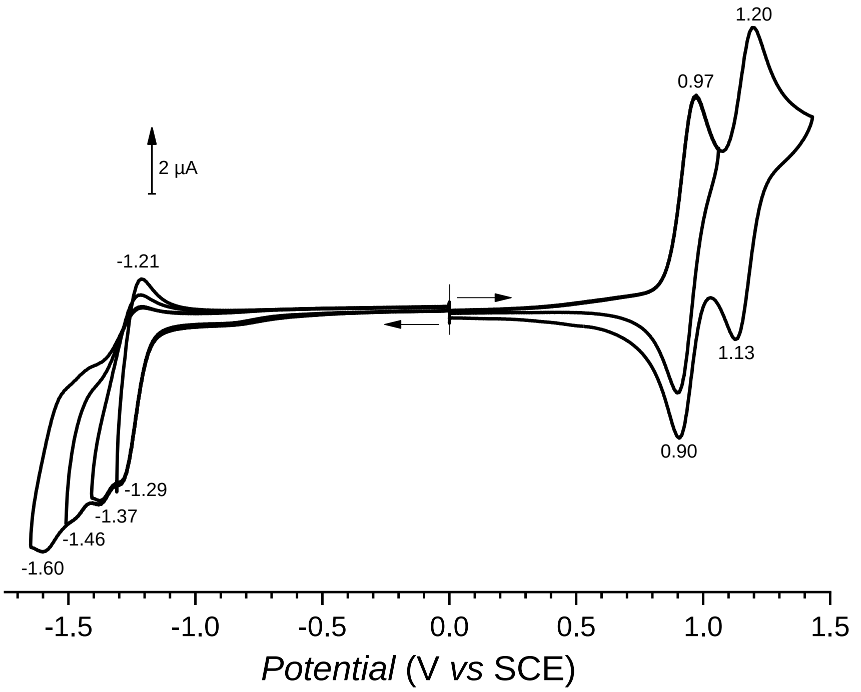

Figure S18. Cyclic voltammogram of compound **Zn-3**$^{+}$ (C = 10^{-3} M in CH_2CN 0.1 M $TEABF_4$, v = 100 mV.s^{-1}, WE: Pt, Ø = 1.6 mm, CE: Pt, RE: SCE, IUPAC convention).

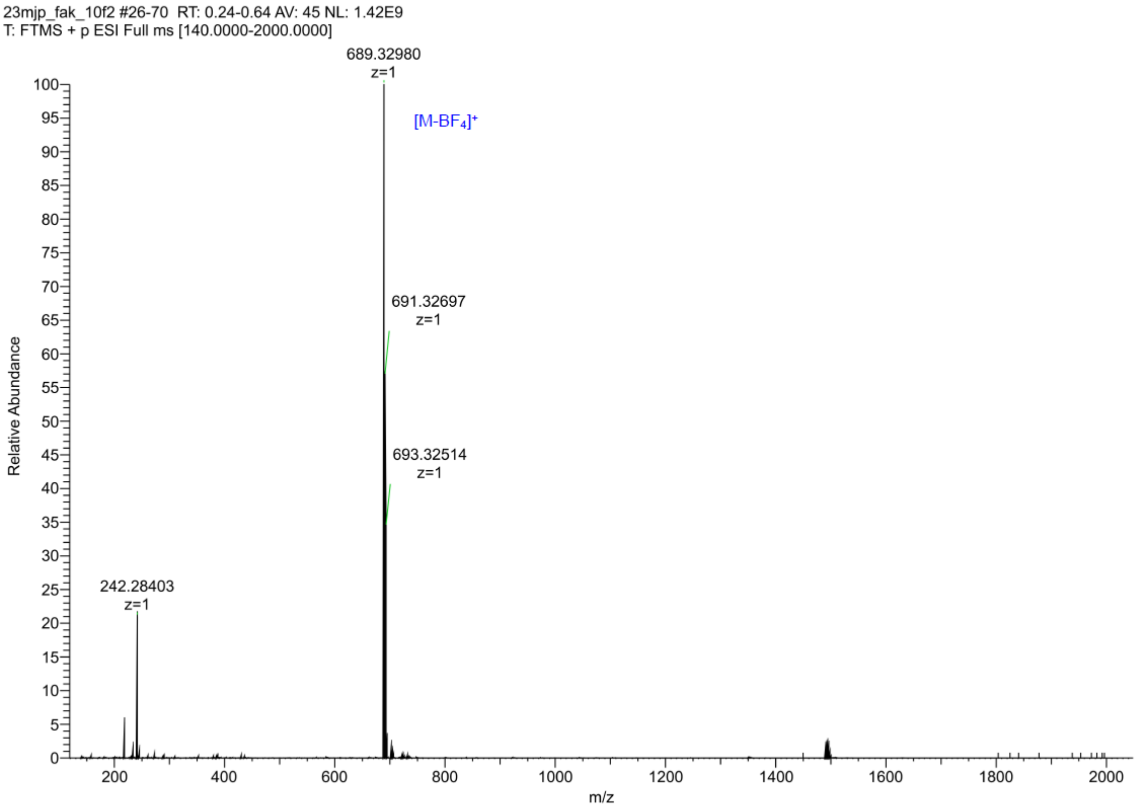

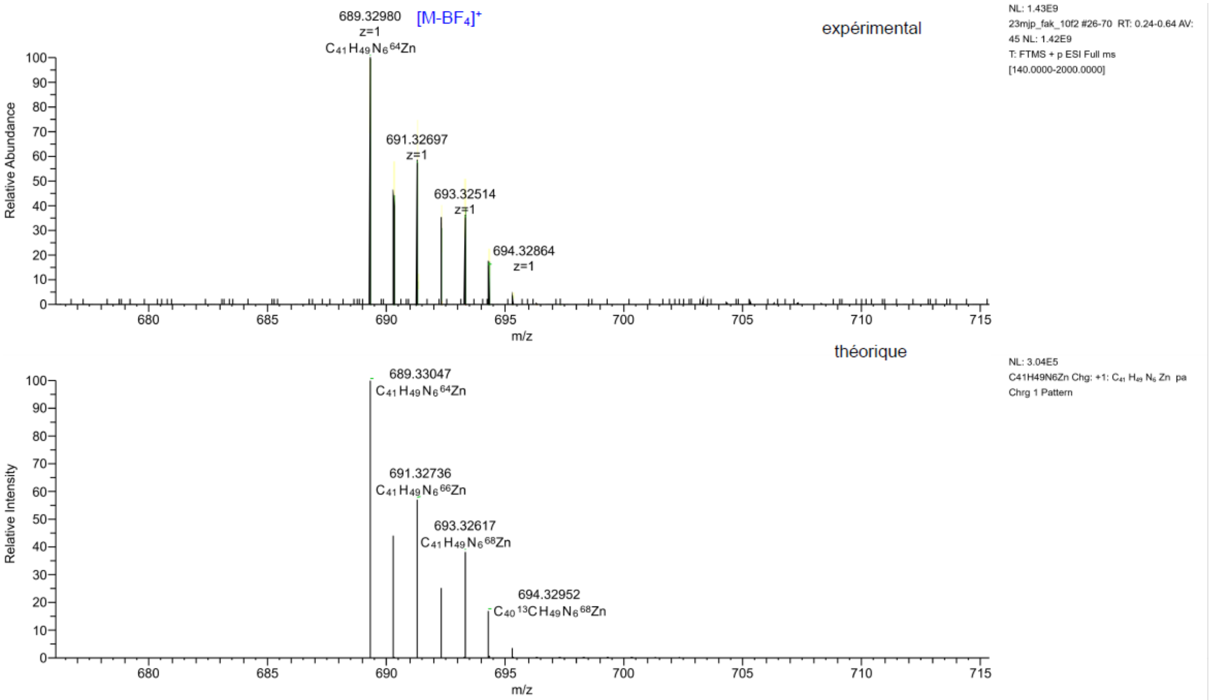

Peak Mass	Display Formula	RDB	Delta [ppm]	Theo. mass	Rank	Pattern Cov. [%]	MSMS Matched Fragm...
689.32980	$C_{41}H_{49}N_6{}^{64}Zn$	20.50	-0.97	689.33047	1	98.55	(Collection)

Figure S19. High resolution ESI mass spectrum of **Zn-3**+ and simulation of its isotopic pattern.

Electrosynthesis of Zn-4⁺

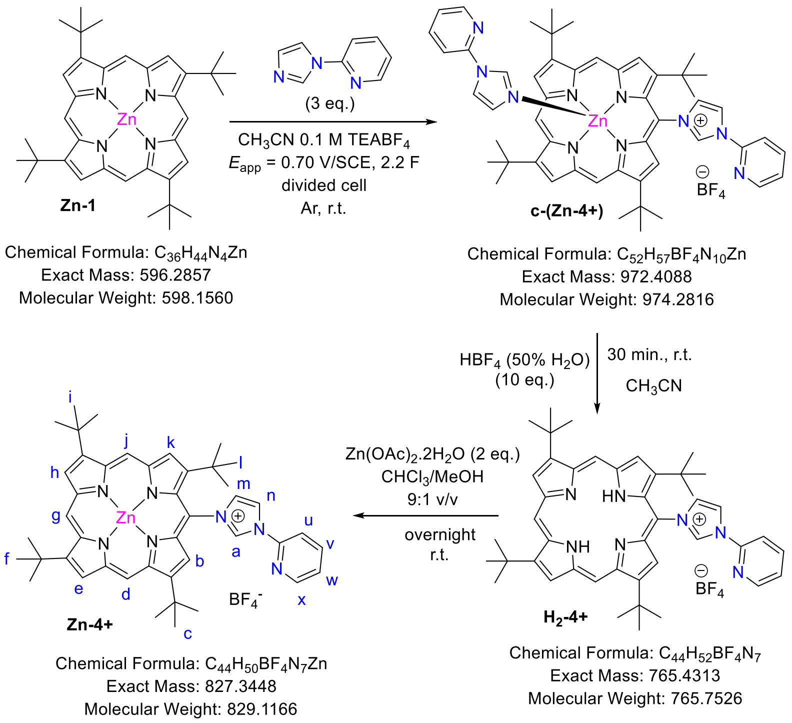

Zn-1 (200.2 mg, 3.35×10^{-1} mmol, 1 eq.) and 1-pyridinylimidazole (145.6 mg, 1.00 mmol, 3 eq.) were dissolved in CH_3CN (200 mL, 0.1 M $TEABF_4$). Electrolysis was carried out under an argon atmosphere under vigorous stirring at room temperature and at controlled potential (E_{app} = 0.70 V/SCE). Electrolysis was stopped after an uptake of 2.2 F *vs* **Zn-1** and the solvent was removed by rotary evaporation. The crude solid was dissolved in CH_2Cl_2 and washed three times (3×200 mL) with water to remove the supporting electrolyte. The product was purified by column chromatography (SiO_2, CH_2Cl_2 containing 5% CH_3OH). The fractions containing **c-(Zn-4⁺)** were gathered and recrystallized in CH_2Cl_2/*n*-heptane. This porphyrin was dissolved in CH_3CN and HBF_4 (50% in H_2O, 378 µL, 3.01 mmol, 9 eq. *vs* **Zn-1**) to remove the zinc(II) metal (and the coordinated nucleophile). This mixture was stirred at room temperature for 30 min and the solvents were evaporated. The crude solid was dissolved in CH_2Cl_2 and washed three times (3×200 mL) with water. The solvent was then removed and the crude solid was dissolved in 20 mL of $CHCl_3/CH_3OH$ (9:1 v/v) mixture and 2 eq. of $Zn(OAc)_2.2H_2O$) *vs* **Zn-1** (146.9 mg, 6.69×10^{-1} mmol) were added. The reaction was stirred overnight at room temperature, the solvent was evaporated, and the crude product was dissolved in CH_2Cl_2 and washed three times with H_2O. The product was recrystallized in CH_2Cl_2/*n*-heptane and dried at 110 °C for 6 h to give **Zn-4⁺** in 87% yield (242.0 mg, 2.91×10^{-1} mmol).

^{1}H NMR ($(CD_3)_2SO$, 500 MHz, 298 K): δ (ppm): 11.37 (s, 1H, Ha), 10.68 (s, 1H, Hj), 10.629 (s, 1H, Hg), 10.625 (s, 1H, Hd), 9.76 (s, 1H, Hk), 9.69 (br s, 1H, Hm), 9.48 (s, 1H, Hh), 9.42 (s, 1H, He), 9.22 (br s, 1H, Hn), 8.82 (d, $^3J_{H\text{-}H}$ = 4.8 Hz, 1H, Hx), 8.35 (m, 2H, Hu and Hv), 8.07 (s, 1H, Hb), 7.79 (m, 1H, Hw), 2.29 (s, 9H, Hi), 2.28 (s, 9H, Hf), 2.16 (s, 9H, Hc), 1.71 (s, 9H, Hl).

$^{13}C\{^{1}H\}$ NMR (($(CD_3)_2SO$, 125 MHz, 298 K): 156.0, 155.9, 155.3, 152.5, 150.1, 148.8, 148.6, 147.55, 147.51, 147.2, 147.0, 146.4, 145.7, 145.3, 142.5, 141.4, 136.0, 134.1, 130.9, 130.2, 126.4, 124.8, 118.2, 115.7, 108.2, 106.3, 106.0, 34.38, 34.36, 34.29, 34.23, 34.06, 34.03, 33.88, 33.86.

λ_{max} (DMSO)/ nm (log ε): 583 (4.14), 545 (4.17), 416 (5.49), 326 (430).

HRMS (ESI+): m/z cald for $C_{44}H_{50}N_7Zn^+$ [M-BF_4^-] 740.3414, found 740.3407.

Characterization of Zn-4⁺

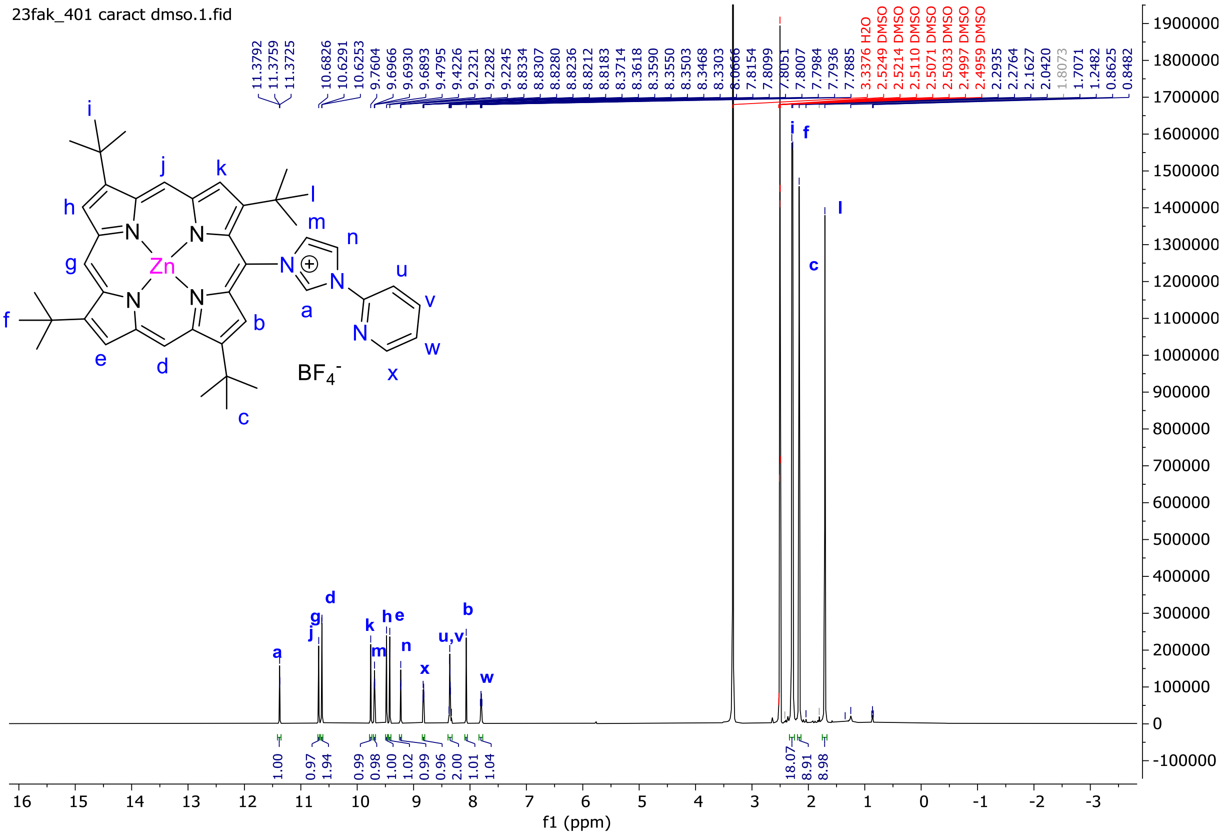

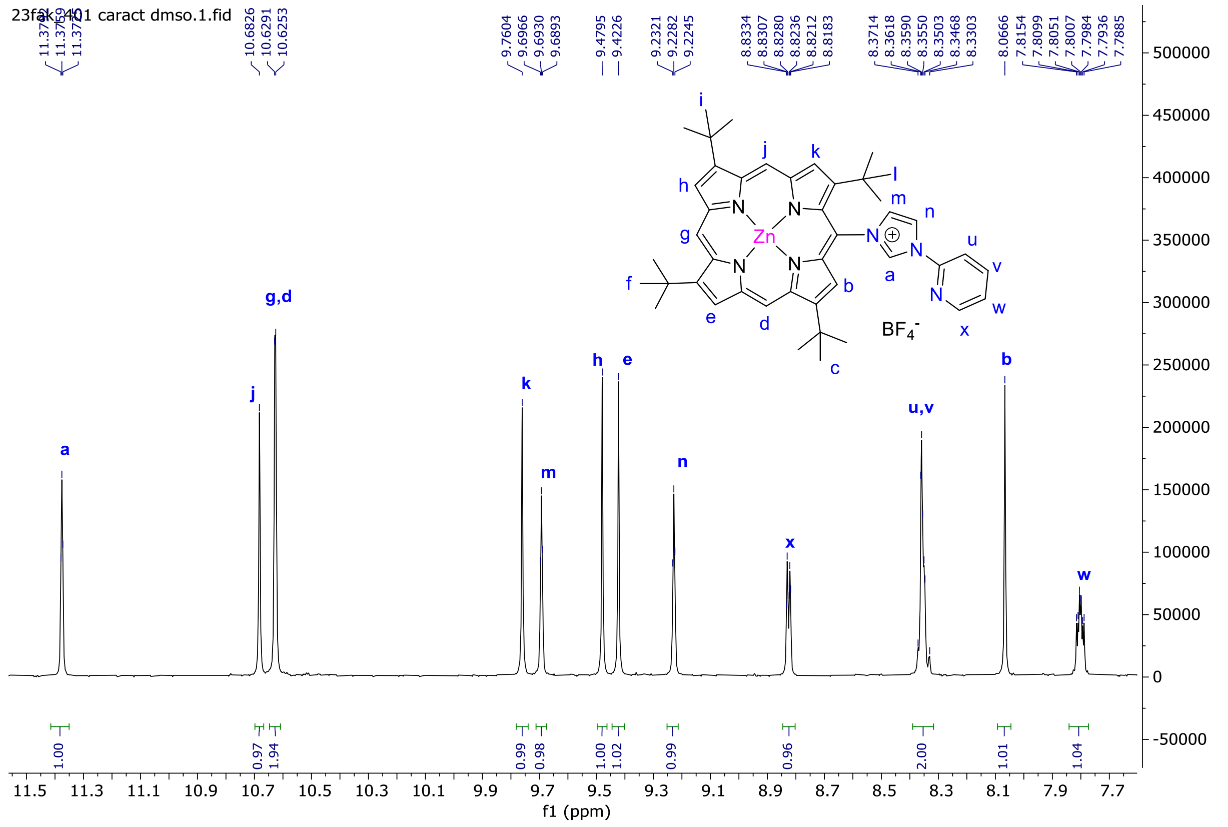

Figure S20. Full range (top) and partial (bottom) ^{1}H NMR spectra of **Zn-4⁺** in $(CD_3)_2SO$, 500 MHz, 298 K.

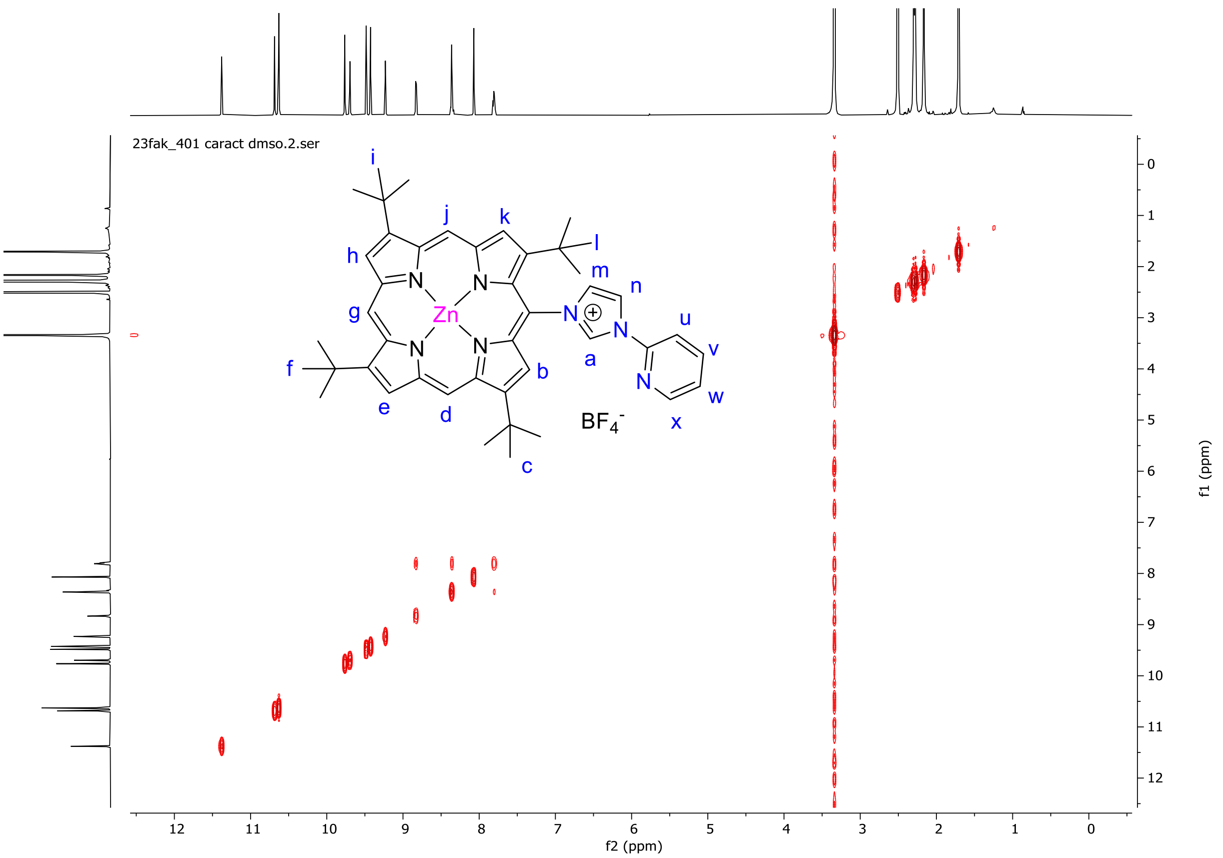

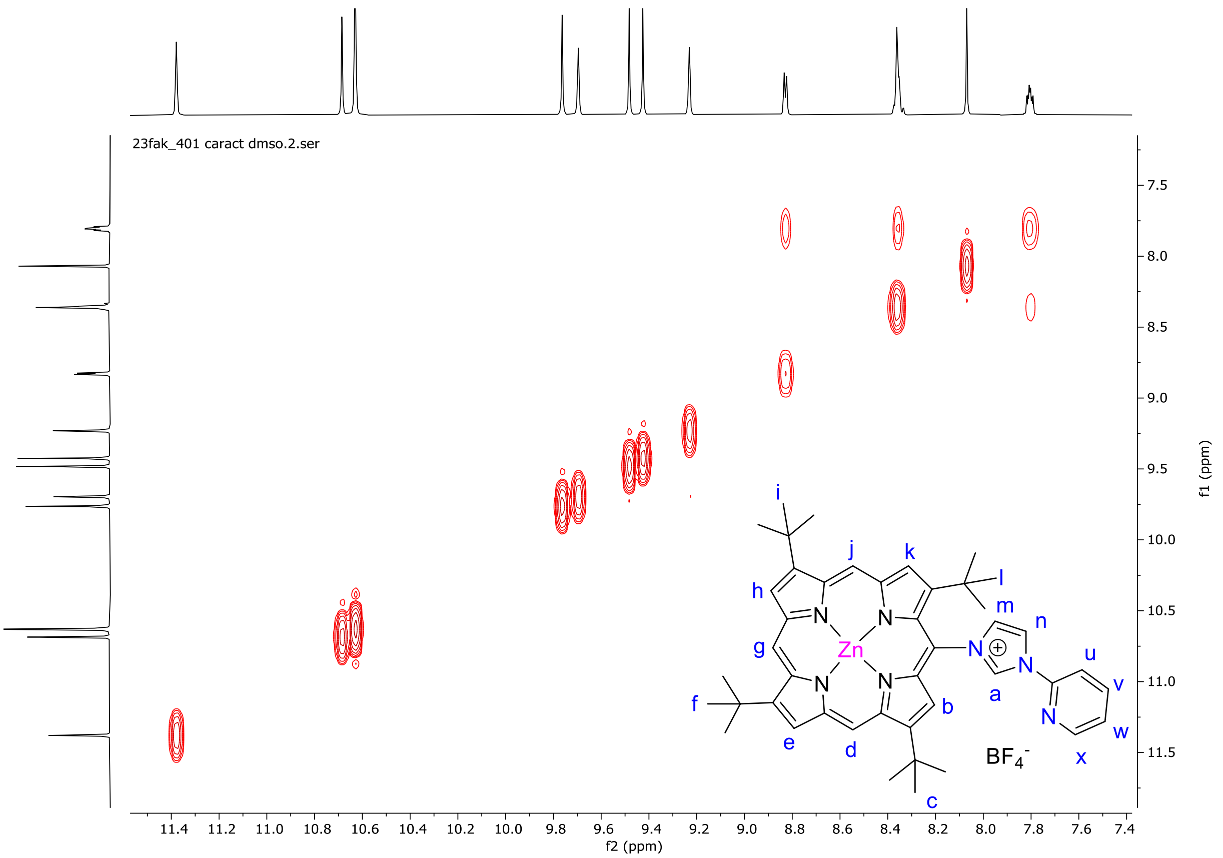

Figure S21. Full range (top) and partial (bottom) ^{1}H-^{1}H COSY spectra of **Zn-4**$^+$ in $(CD_3)_2SO$, 500 MHz, 298 K.

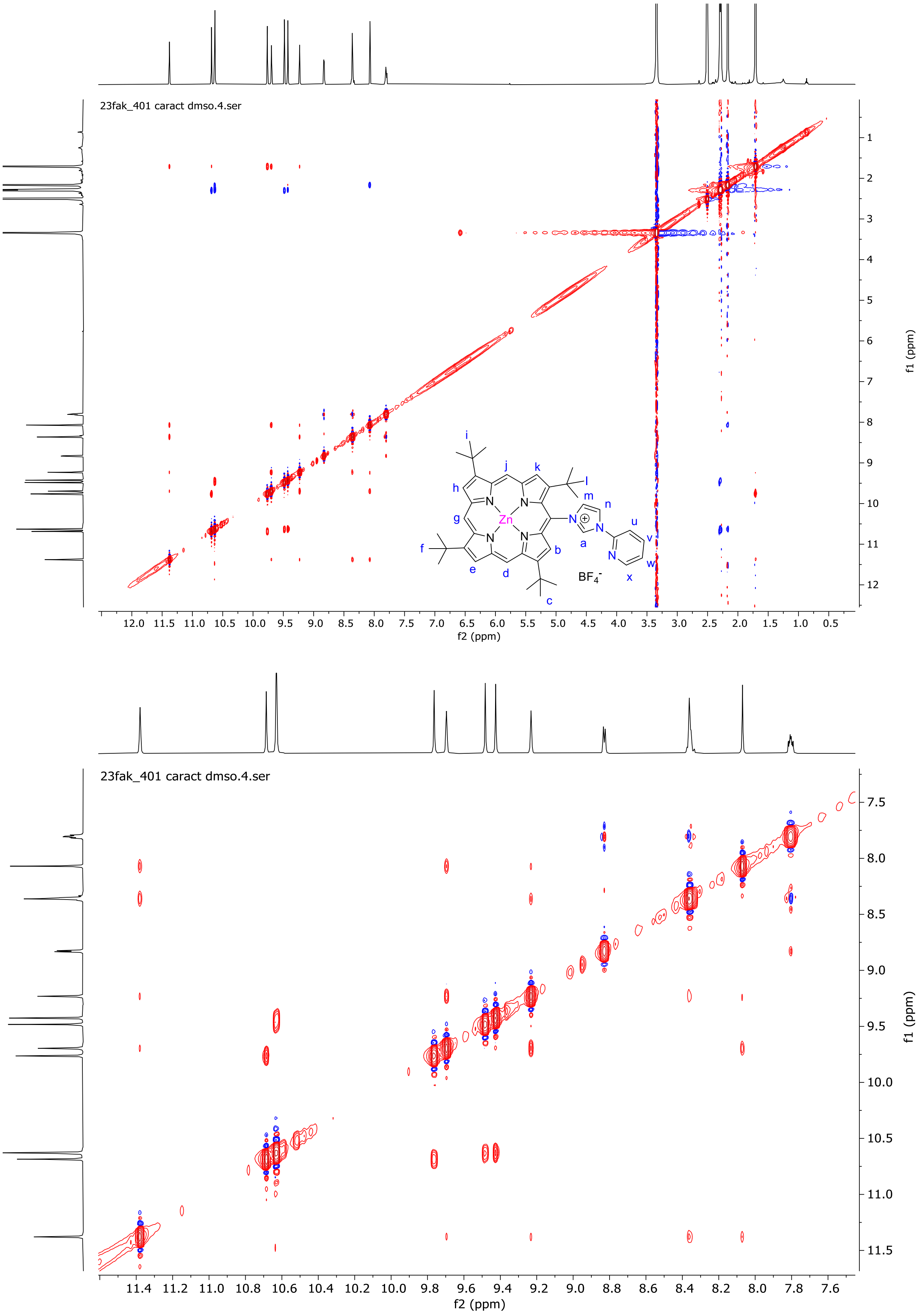

Figure S22. Full range (top) and partial (bottom) ^{1}H-^{1}H NOESY spectra of **Zn-4$^+$** in $(CD_3)_2SO$, 500 MHz, 298 K

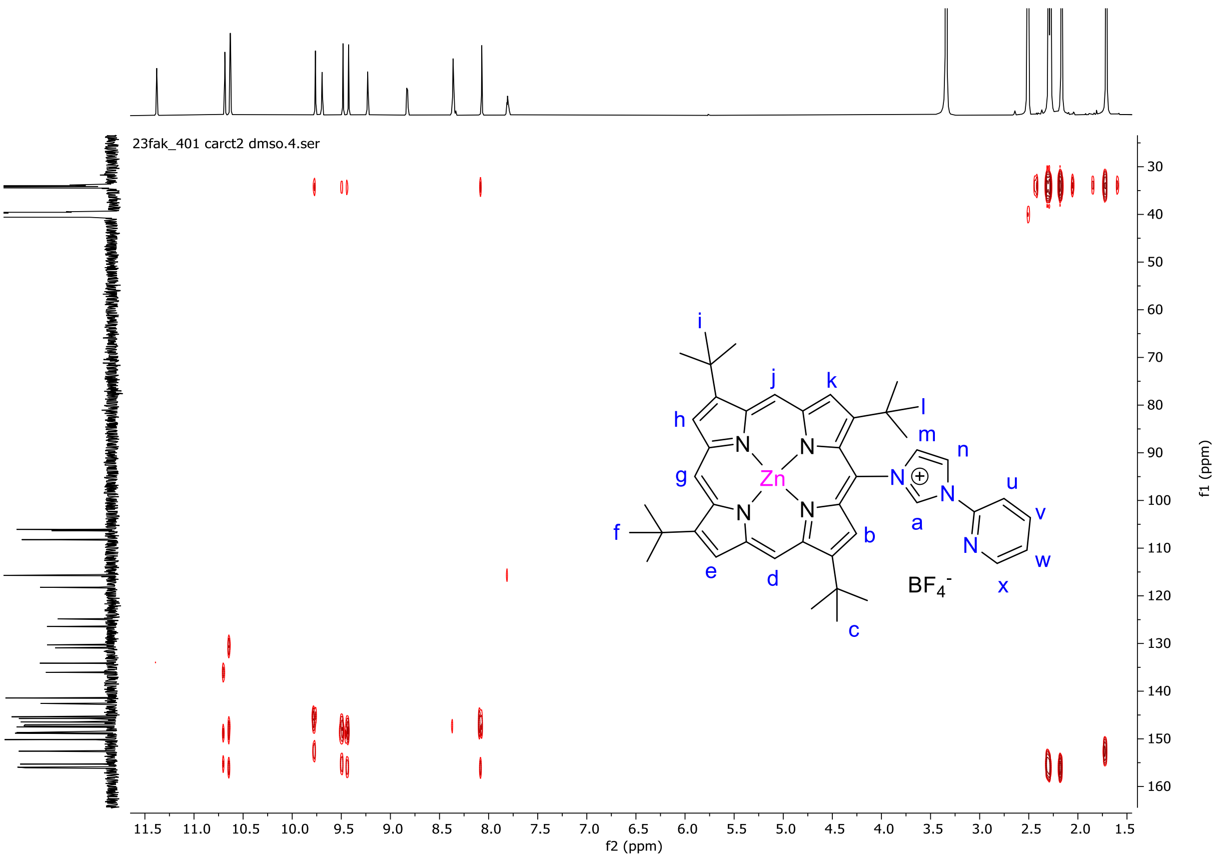

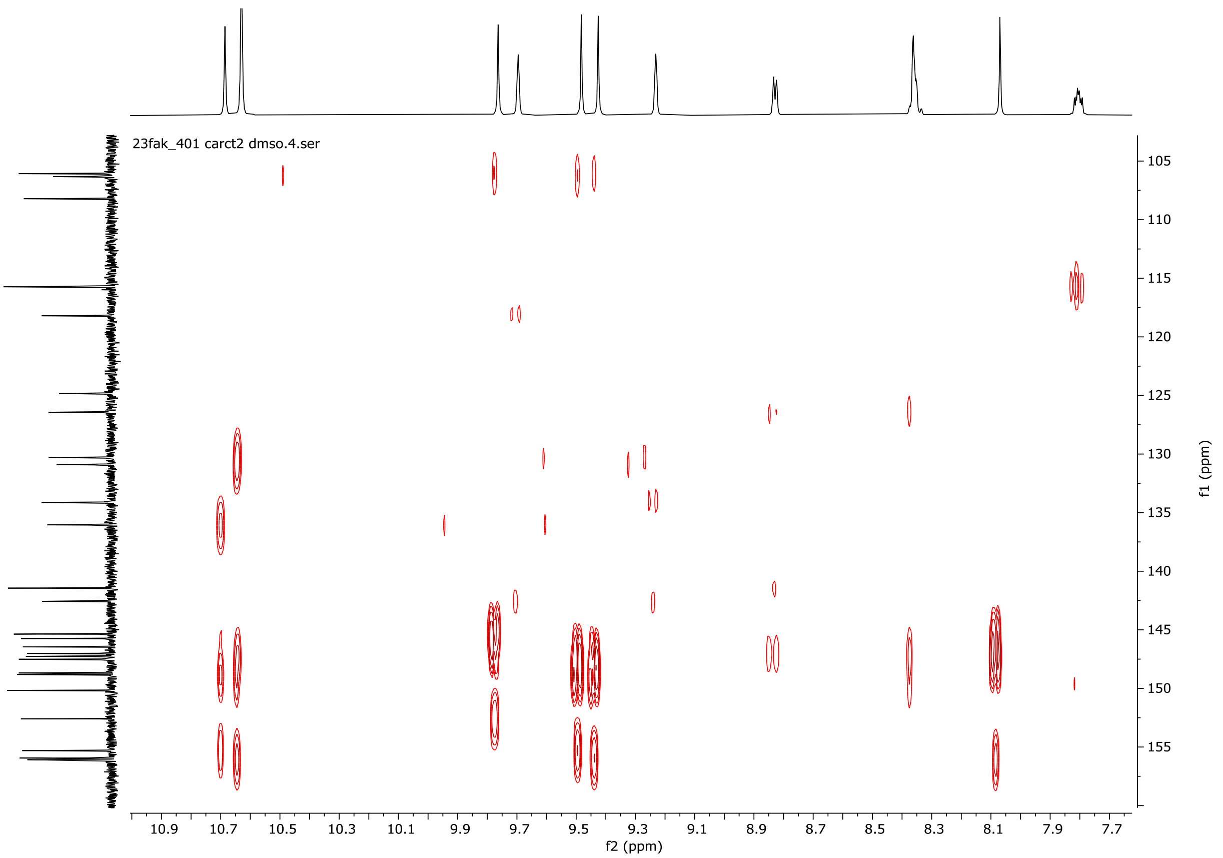

Figure S23. Full range (top) and partial (bottom) ^{1}H-^{13}C HMBC spectra of **Zn-4**$^{+}$ in $(CD_3)_2SO$, 500 MHz, 298 K

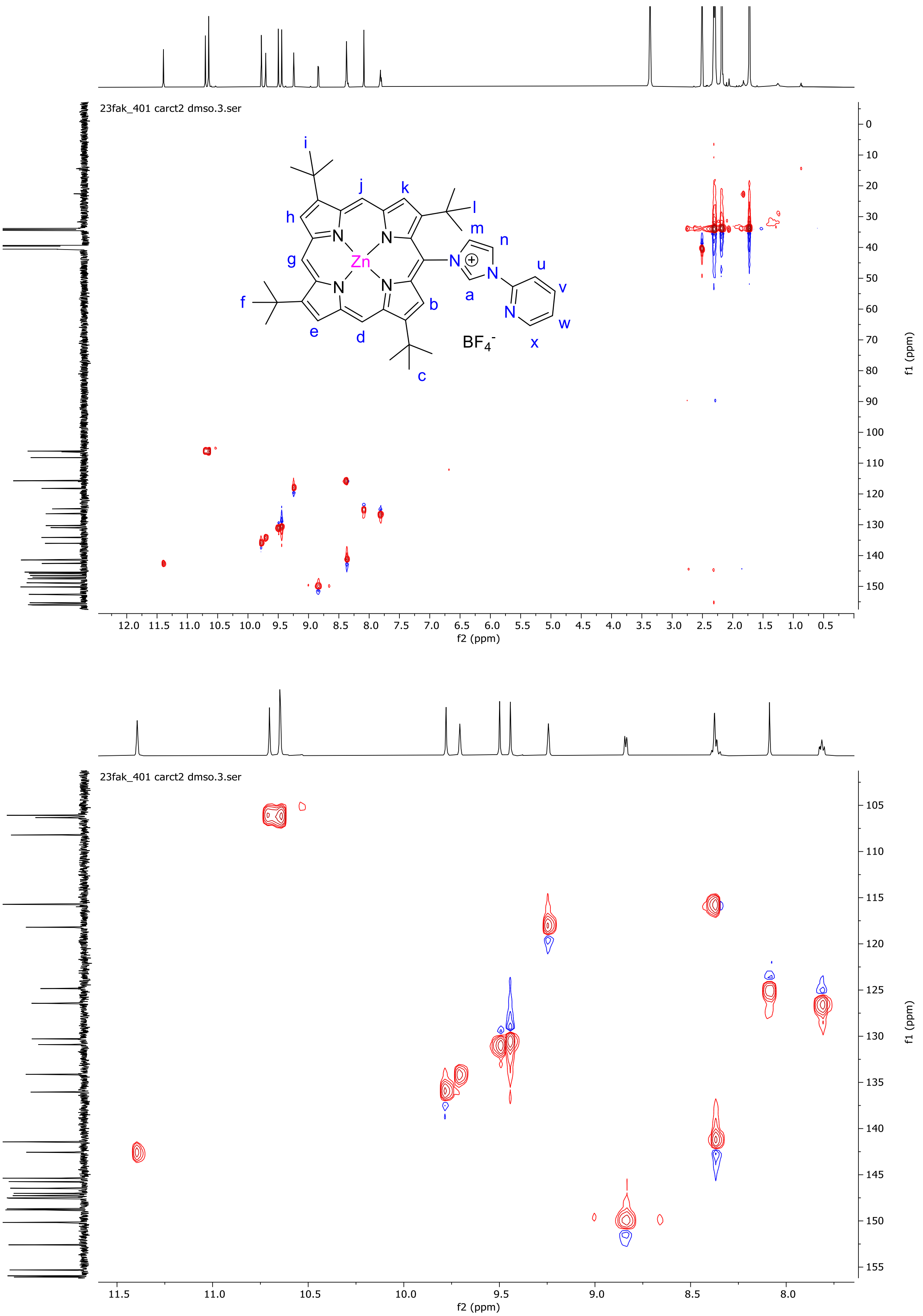

Figure S24. Full range (top) and partial (bottom) ^1H-^{13}C HSQC spectra of **Zn-4**$^+$ in $(CD_3)_2SO$, 500 MHz, 298 K

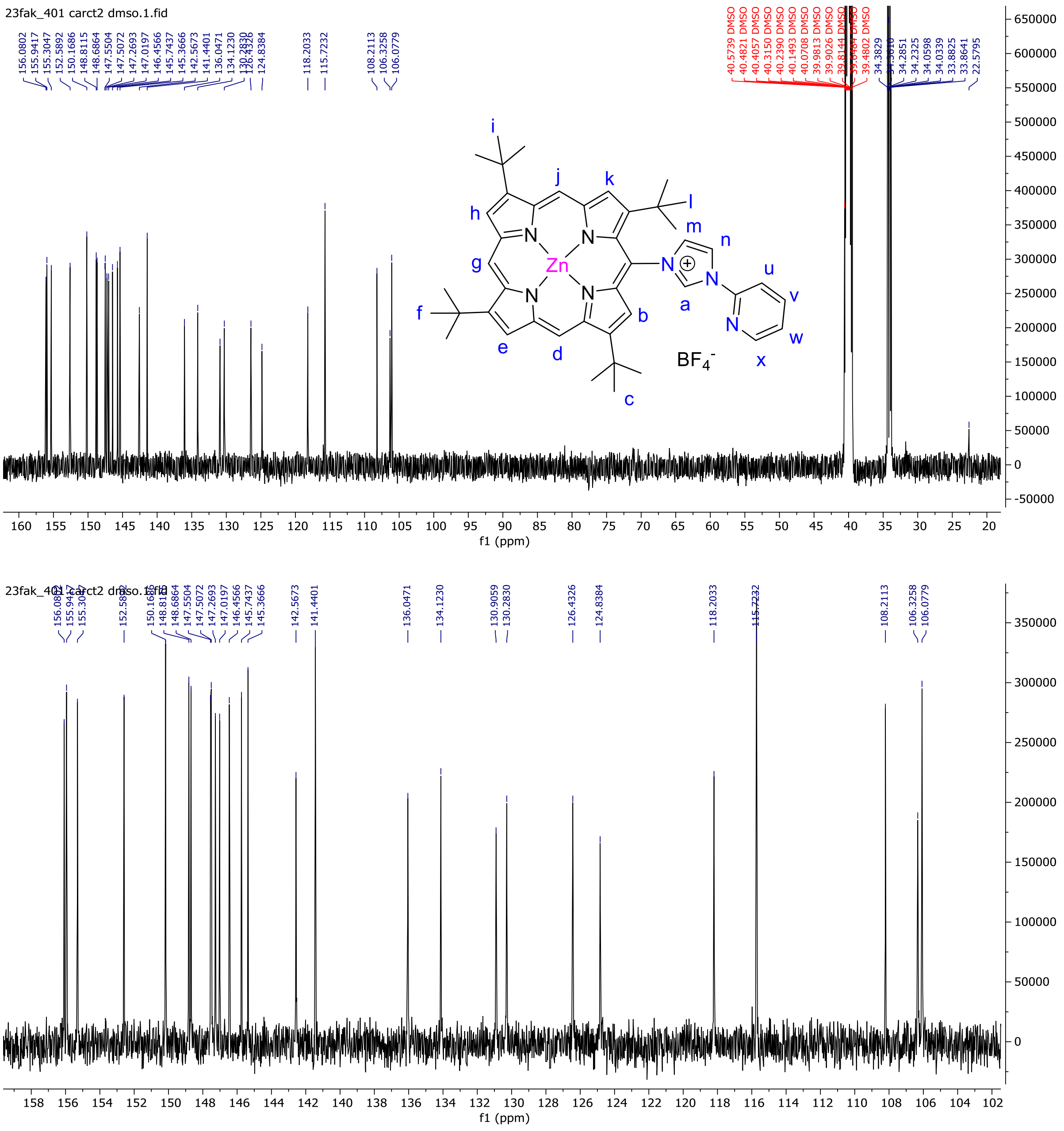

Figure S25. Full range (top) and partial (bottom) ^{13}C spectra of **Zn-4$^+$** in $(CD_3)_2SO$, 500 MHz, 298 K

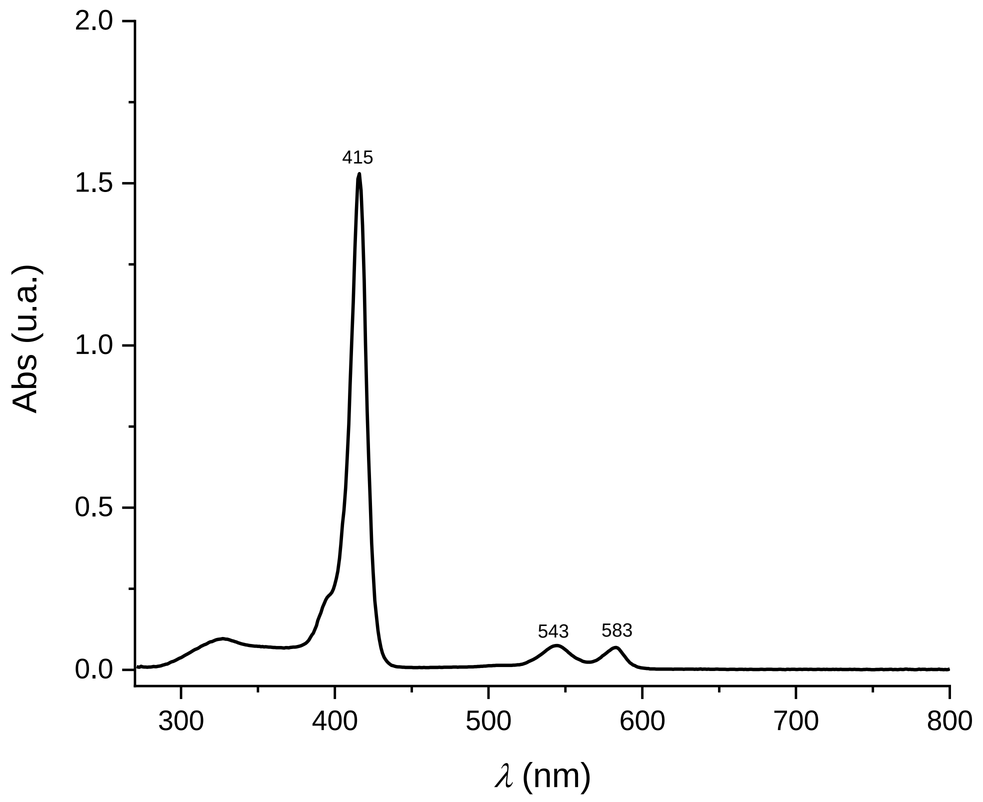

Figure S26. UV-Vis. absorption spectrum of **Zn-4**$^+$ in DMSO, room temperature

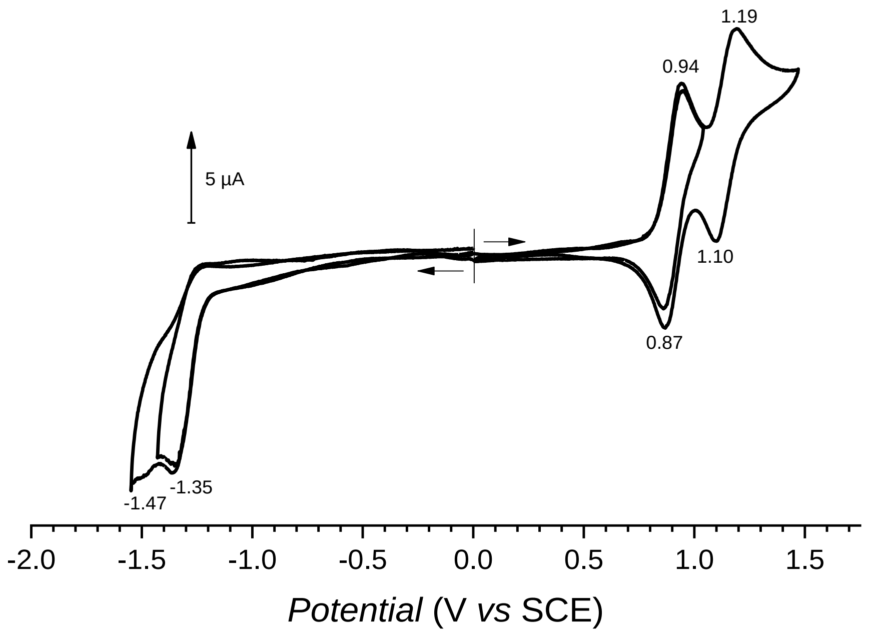

Figure S27. Cyclic voltammogram of compound **Zn-4**$^+$ ($C = 10^{-3}$ M in CH_3CN 0.1 M $TEABF_4$, v = 100 mV.s^{-1}, WE: Pt, Ø = 1.6 mm, CE: Pt, RE: SCE, IUPAC convention).

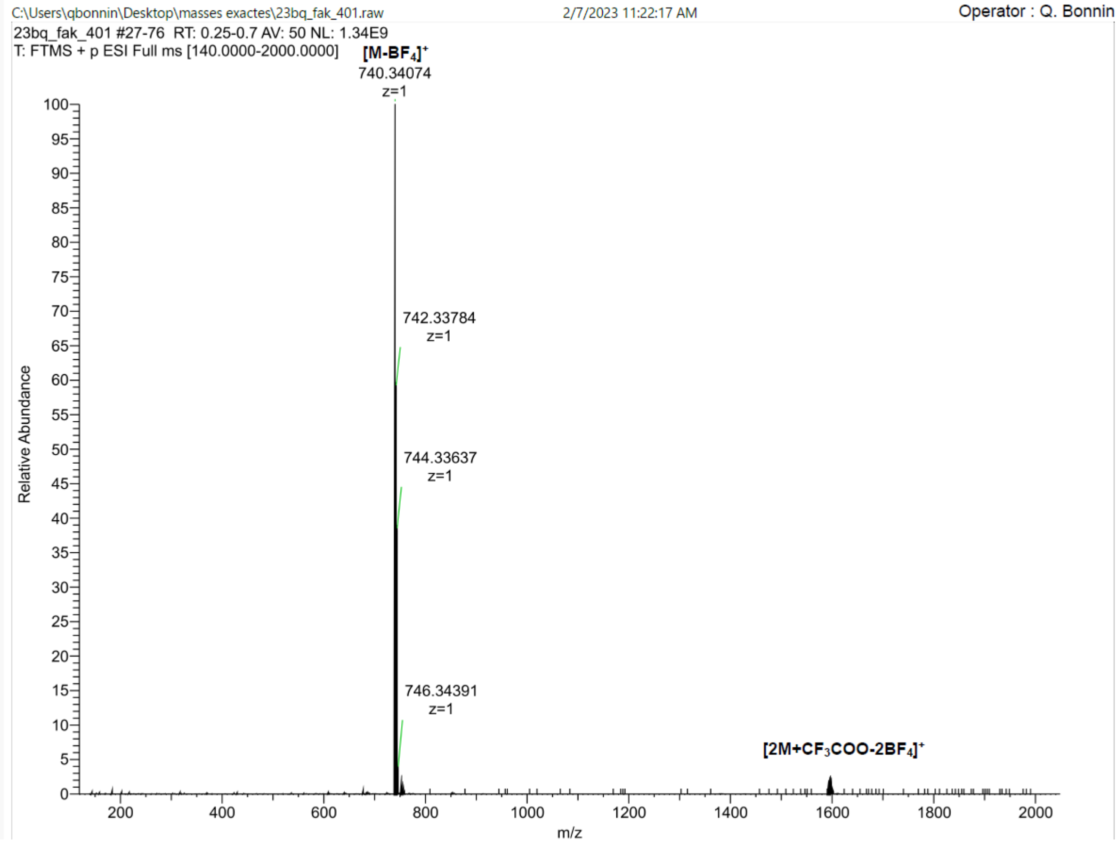

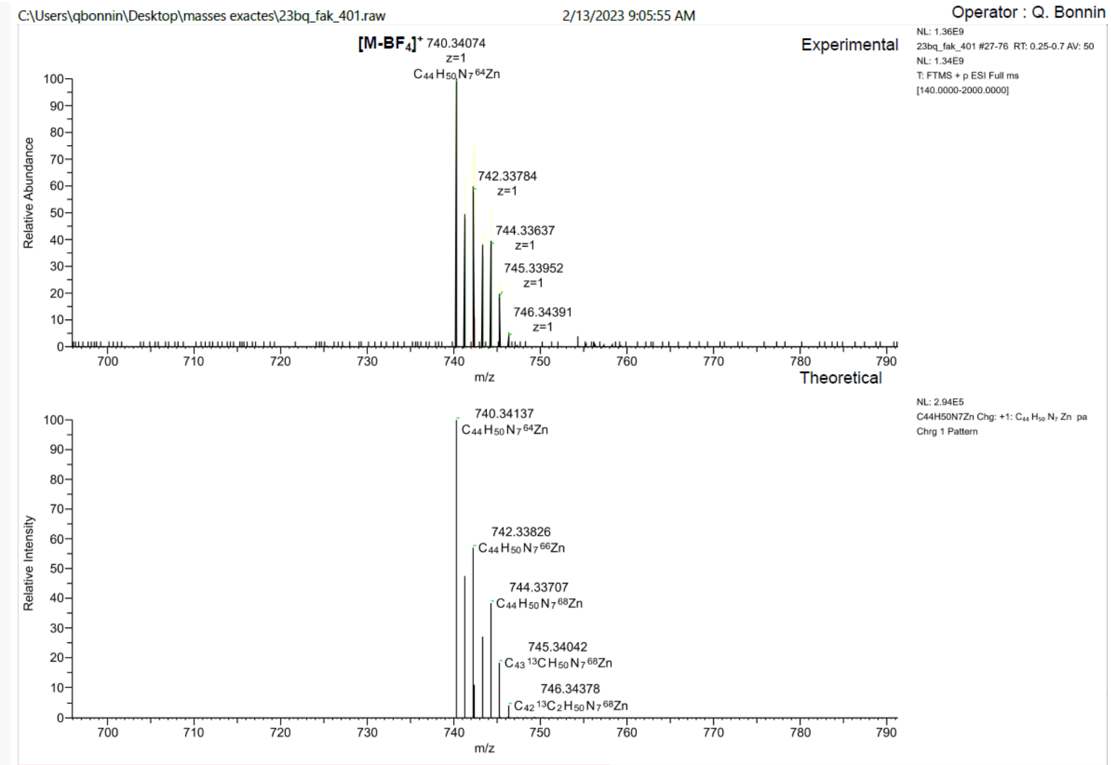

Display Formula	Peak Mass	Theo. mass	Delta [ppm]	RDB	Rank	MSMS Matched Fragments
$C_{44}H_{50}N_7{}^{64}Zn$	740.34074	740.34137	-0.85	23.50	1	(Collection)

Figure S28. High resolution ESI mass spectrum of **Zn-4**$^+$ and simulation of its isotopic pattern.

Electrosynthesis of Zn-5$^+$

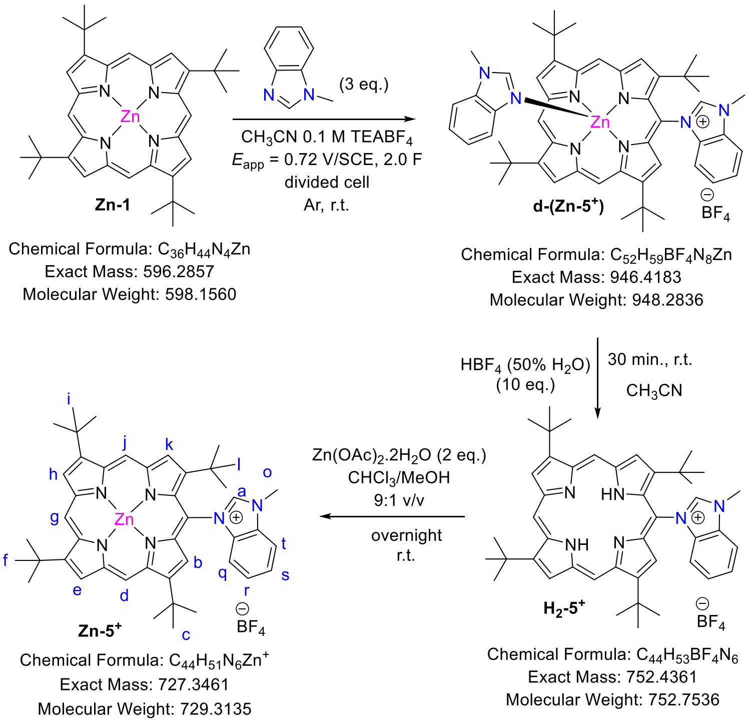

Zn-1 (199.1 mg, 3.33×10^{-1} mmol, 1 eq.) and methylbenzimidazole (133.3 mg, 1.00 mmol, 3 eq.) were dissolved in CH_3CN (200 mL, 0.1 M $TEABF_4$). Electrolysis was performed under an argon atmosphere under vigorous stirring at room temperature and at controlled potential (E_{app} = 0.74 V/SCE). Electrolysis was stopped after an uptake of 2.0 F *vs* **Zn-1** and the solvent was removed by rotary evaporation. The crude solid was dissolved in CH_2Cl_2 and washed three times (3×200 mL) with water to remove the supporting electrolyte. The product was purified by column chromatography (SiO_2, CH_2Cl_2 containing 5% CH_3OH). The fractions containing **d-(Zn-5$^+$)** were gathered and recrystallized in CH_2Cl_2/*n*-heptane. This porphyrin was dissolved in CH_3CN and HBF_4 (50% in H_2O, 423 µL, 3.37 mmol, 10.1 eq. *vs* **Zn-1**) was added to remove the zinc(II) metal (and the coordinated nucleophile). This mixture was stirred at room temperature for 30 min. and the solvents were evaporated. The crude solid was dissolved in CH_2Cl_2 and washed three times (3×200 mL) with water. The solvent was then removed and the crude solid was dissolved in 20 mL of $CHCl_3/CH_3OH$ (9:1 v/v) mixture and 2 eq. of $Zn(OAc)_2.2H_2O$ *vs* **Zn-1** (146.4 mg, 6.67×10^{-1} mmol) were added. The reaction was stirred overnight at room temperature and the solvent was evaporated. The crude product was dissolved in CH_2Cl_2 and washed 3 times with H_2O. The product was recrystallized in CH_2Cl_2/*n*-heptane and dried at 110 °C for 6 h to give **Zn-5$^+$** in 74% yield (186.0 mg, 2.27×10^{-1} mmol).

^{1}H NMR ($(CD_3)_2SO$, 500 MHz, 298 K): δ (ppm): 11.42 (s,1H, Ha), 10.69 (s, 1H, Hj), 10.63 (s, 1H, Hg), 10.58 (s, 1H, Hd), 9.73 (s, 1H, Hk), 9.49 (s, 1H, Hh), 9.41 (s, 1H, He), 8.50 (d, $^3J_{H\text{-}H}$ = 8.8 Hz, 1H, Ht), 7.88 (dd, $^3J_{H\text{-}H}$ =8.8 and 8.5 Hz, 1H, Hs), 7.67 (s, 1H, Hb), 7.53 (dd, $^3J_{H\text{-}H}$ =8.8 and 8.5 Hz, 1H, Hr), 6.70 (d, $^3J_{H\text{-}H}$ = 8.5, 1H, Hq), 4.68 (s, 3H, Ho), 2.29 (s, 9H, Hi), 2.26 (s, 1H, Hf), 2.04 (s, 9H, Hc), 1.46 (s, 9H, Hl).

^{13}C{1H} NMR ($(CD_3)_2SO$, 500 MHz, 298 K):156.3, 155.9, 155.3, 152.1, 148.8, 148.7, 147.8, 147.6, 147.4, 147.3, 146.3, 146.2, 145.8, 140.5, 136.1, 130.9, 130.7, 130.2, 128.8, 127.9, 124.4, 115.1, 106.4, 106.3, 105.9, 104.9, 34.7, 34.3, 34.3, 34.2, 34.1, 34.0, 34.0, 33.8, 33.7.

λ_{max} (DMSO)/ nm (log ε): 583 (4.20), 545 (4.22), 416 (5.54), 329 (4.34)

HRMS (ESI+): m/z cald for $C_{44}H_{51}N_6Zn^+$ [M-BF_4^-] 727.3461, found 727.3452.

Characterization of Zn-5⁺

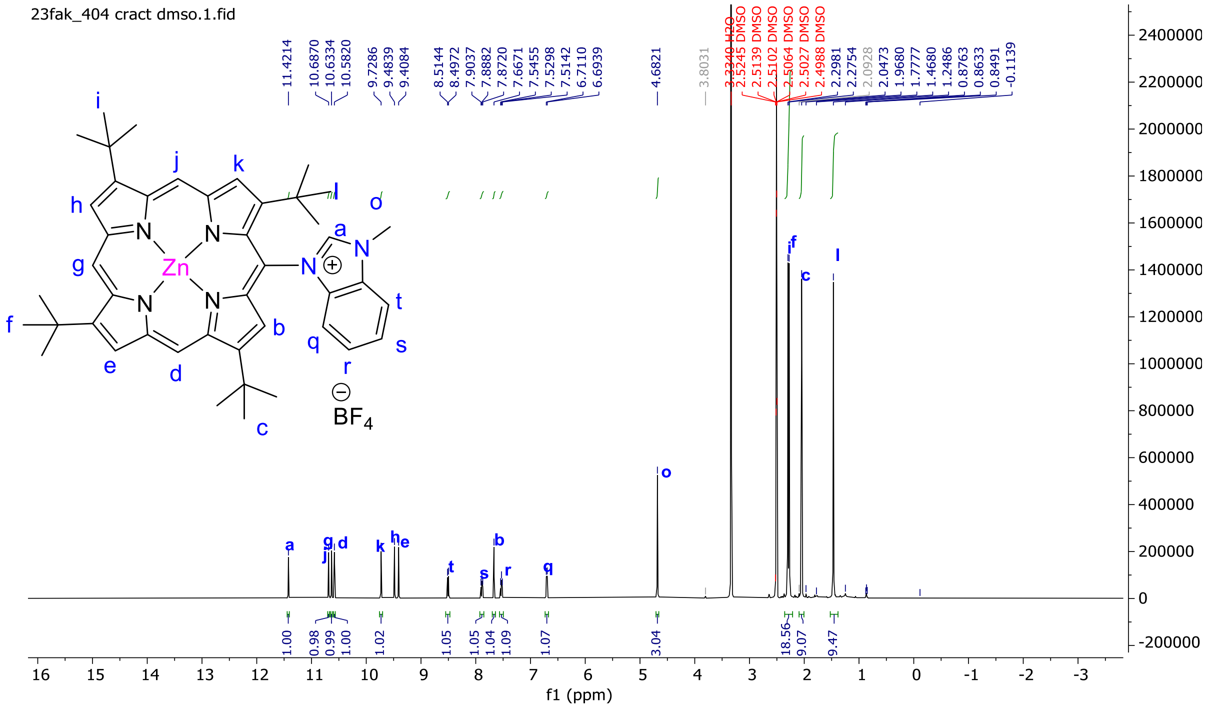

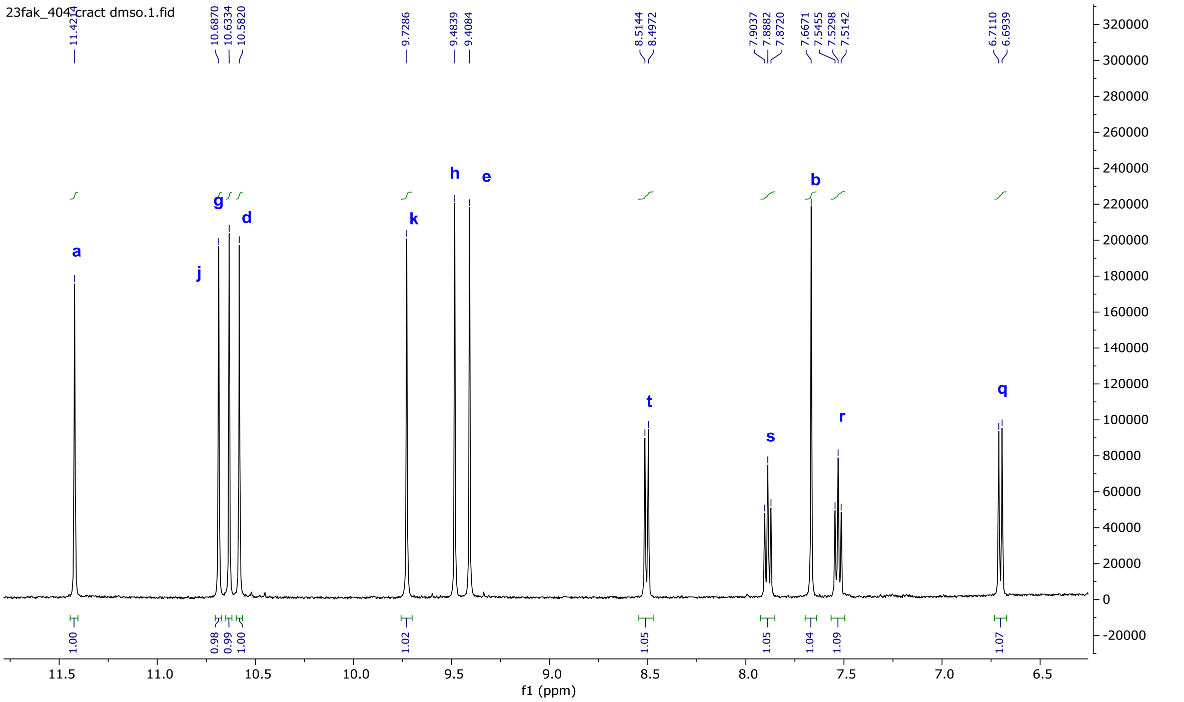

Figure S29. Full range (top) and partial (bottom) ^{1}H NMR spectra of **Zn-5⁺** in $(CD_3)_2SO$, 500 MHz, 298 K.

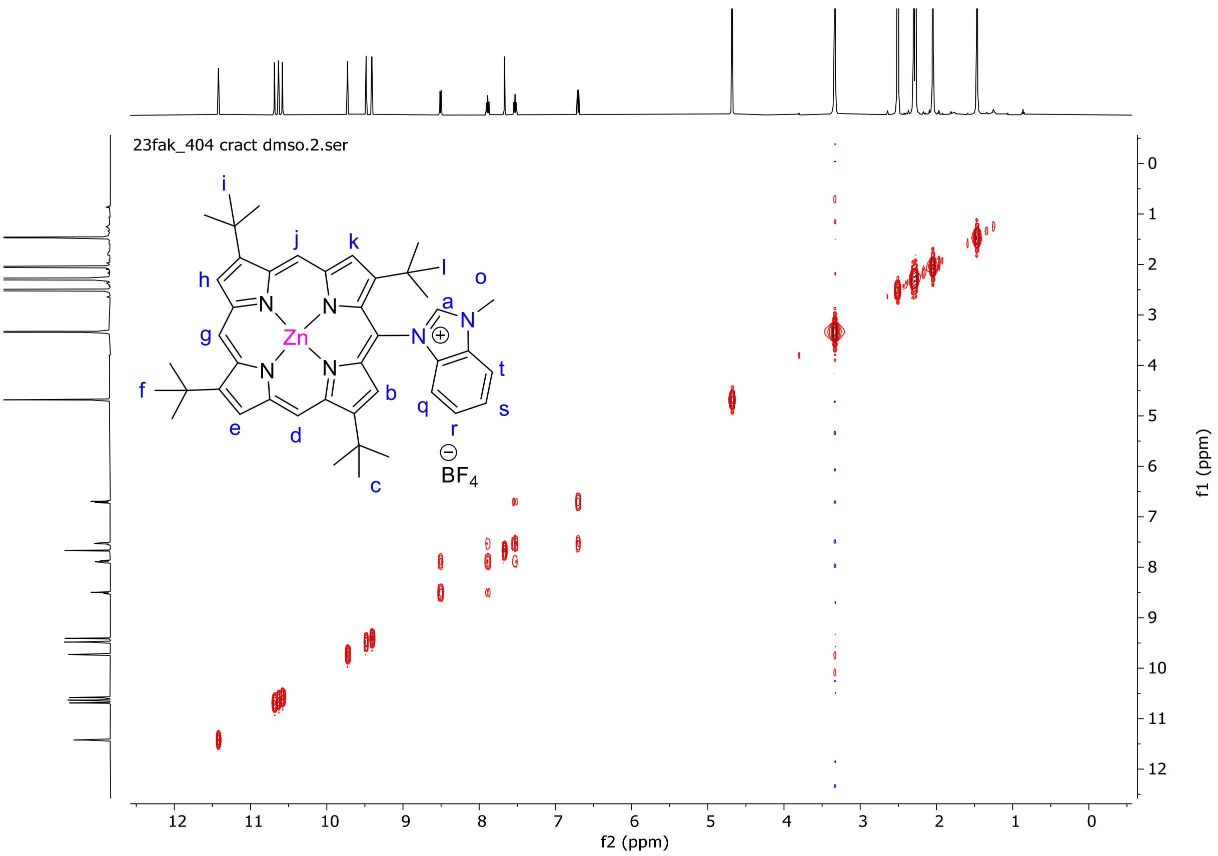

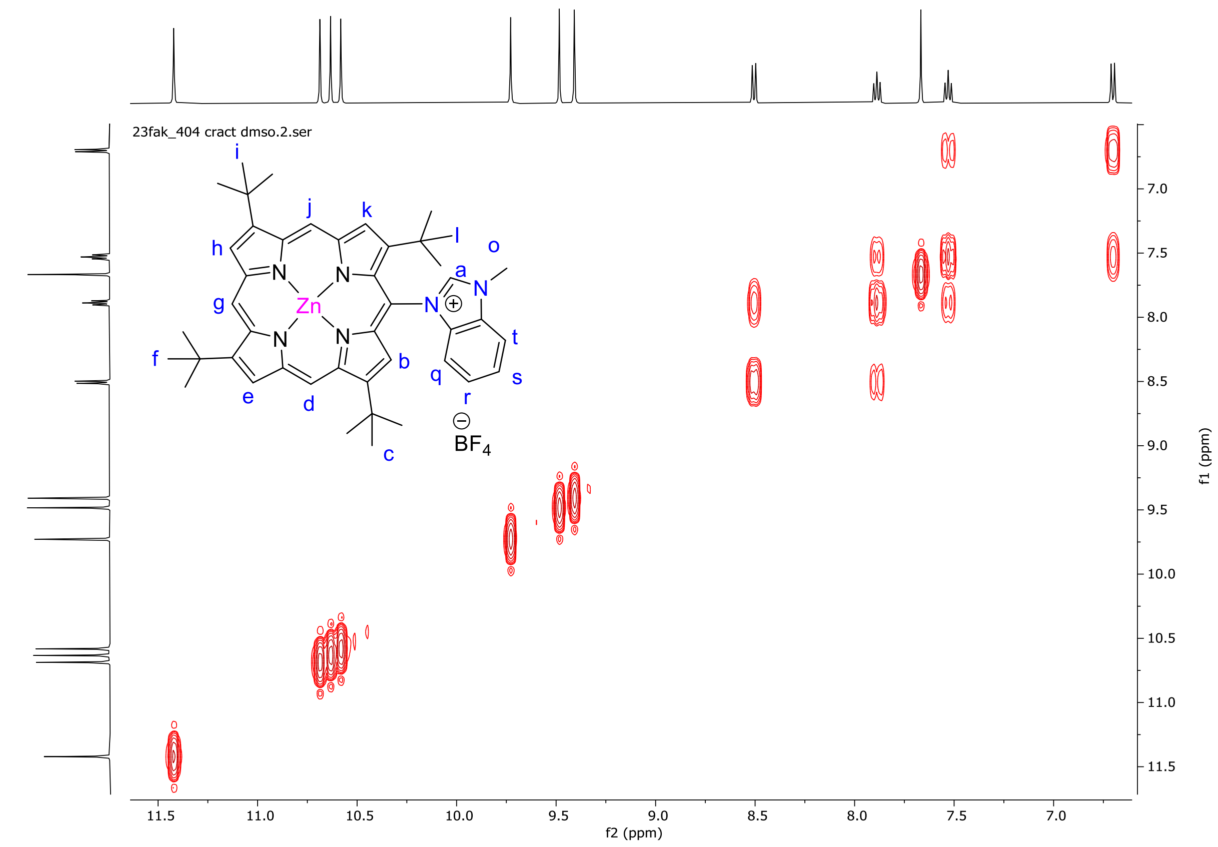

Figure S30. Full range (top) and partial (bottom) ^1H-^1H COSY spectra of **Zn-5$^+$** in $(CD_3)_2SO$, 500 MHz, 298 K.

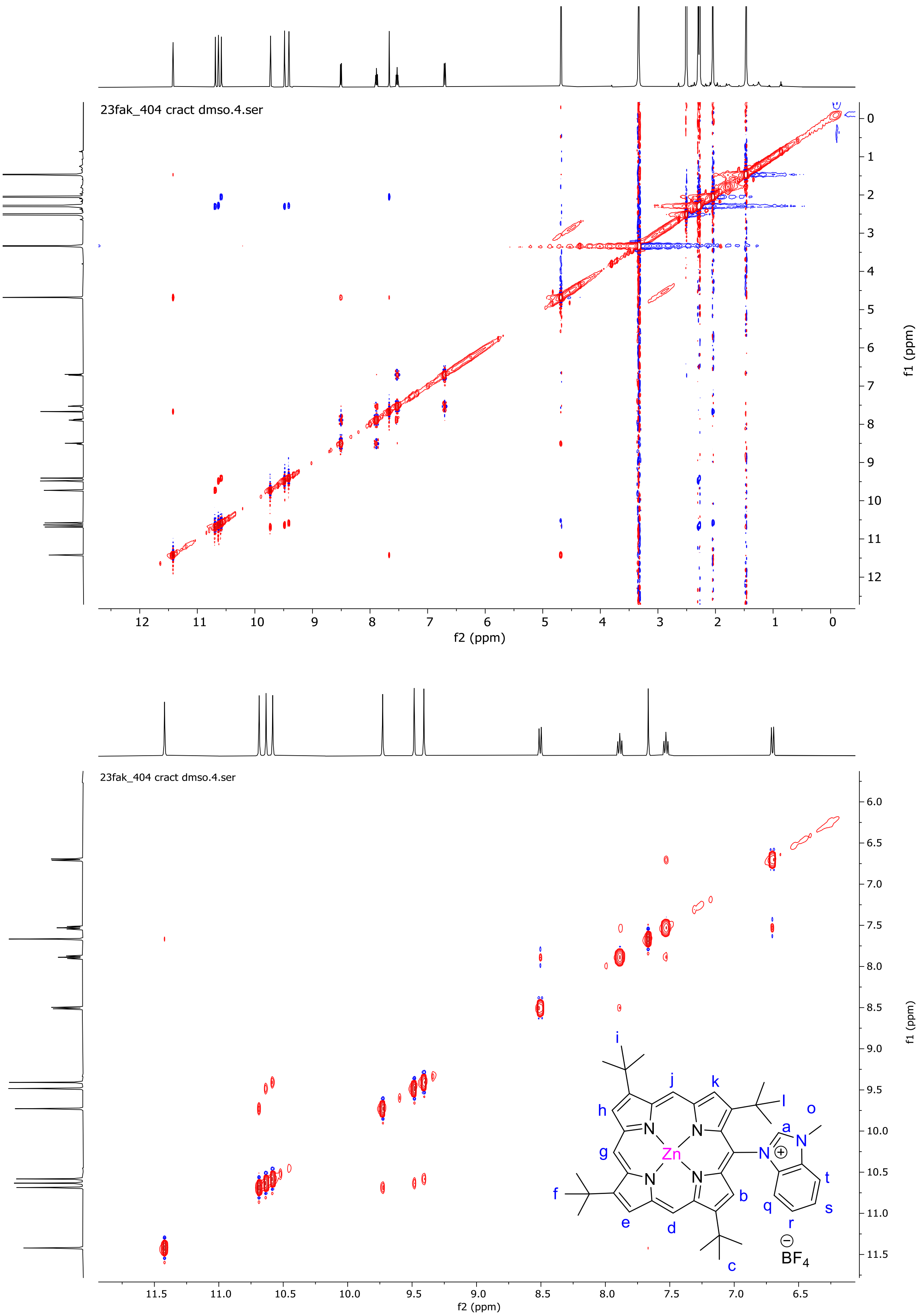

Figure S31. Full range (top) and partial (bottom) ^{1}H-^{1}H NOESY spectra of **Zn-5**$^+$ in $(CD_3)_2SO$, 500 MHz, 298 K.

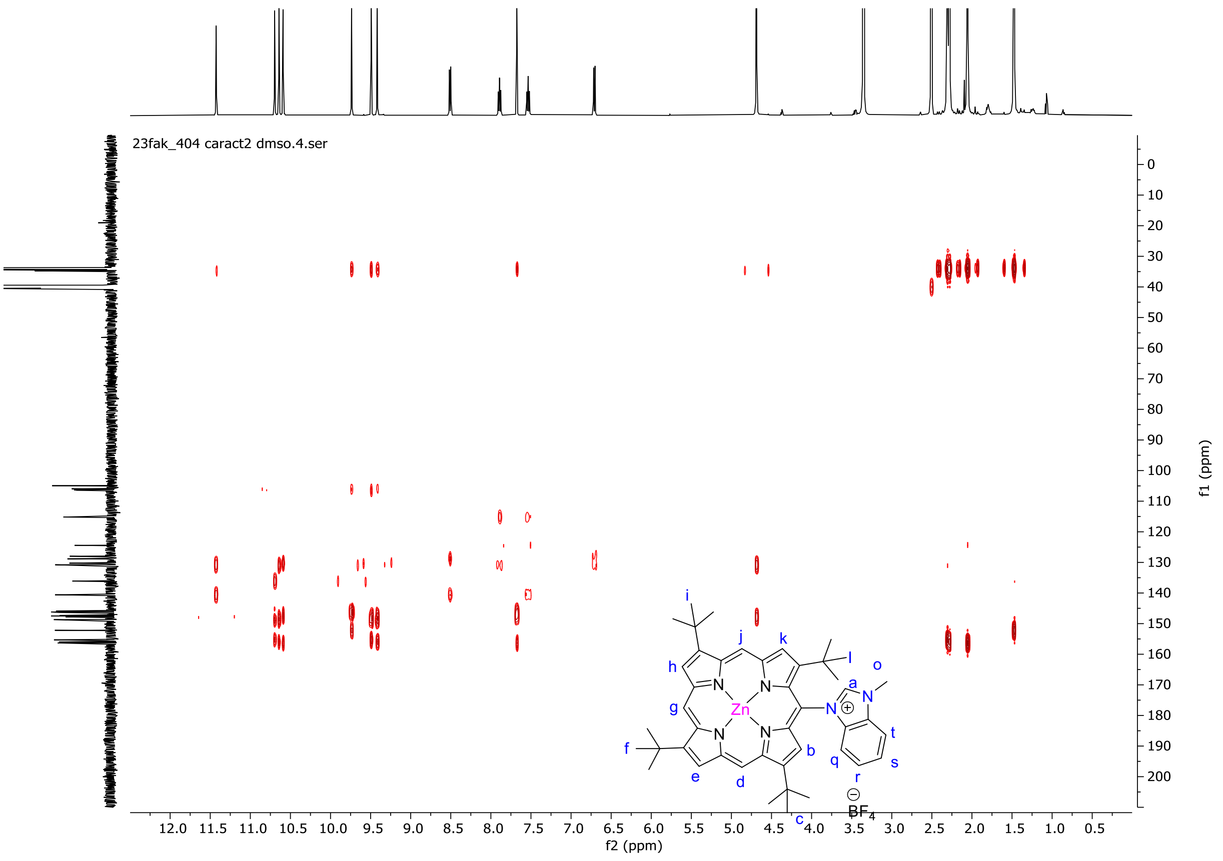

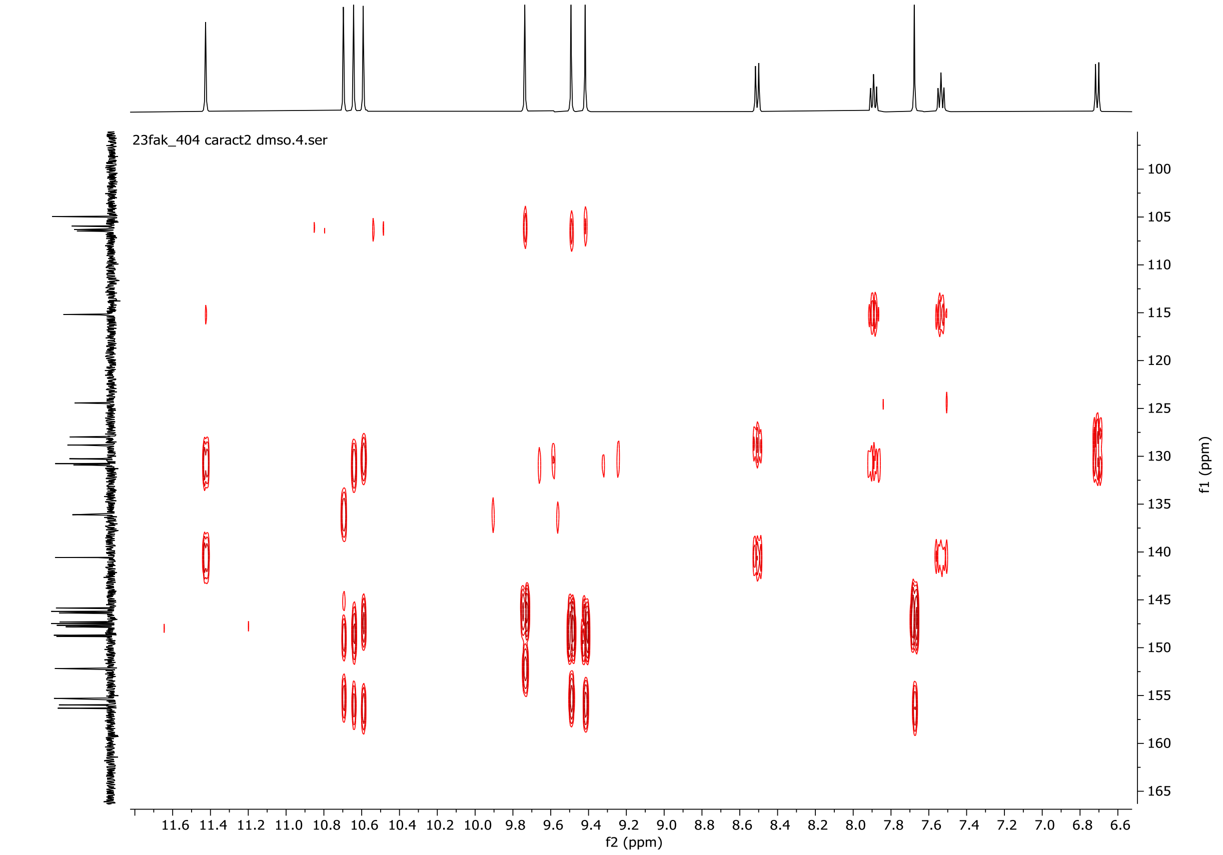

Figure S32 : Full range (top) and partial (bottom) ^{1}H-^{13}C HMBC spectra of **Zn-5**$^{+}$ in $(CD_3)_2SO$, 500 MHz, 298 K.

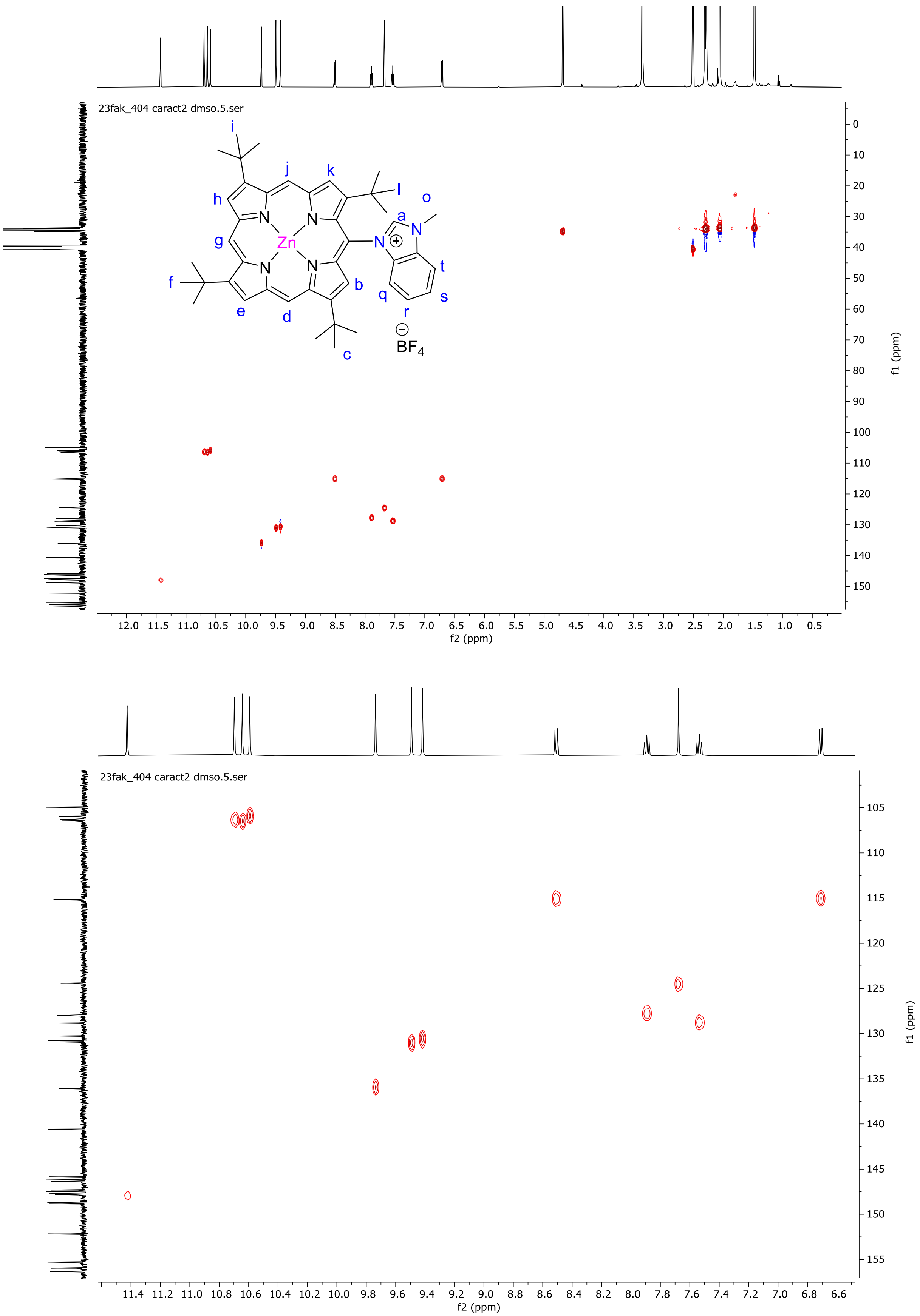

Figure S33 : Full range (top) and partial (bottom) ^{1}H-^{13}C HSQC spectra of **Zn-5**$^{+}$ in $(CD_3)_2SO$, 500 MHz, 298 K

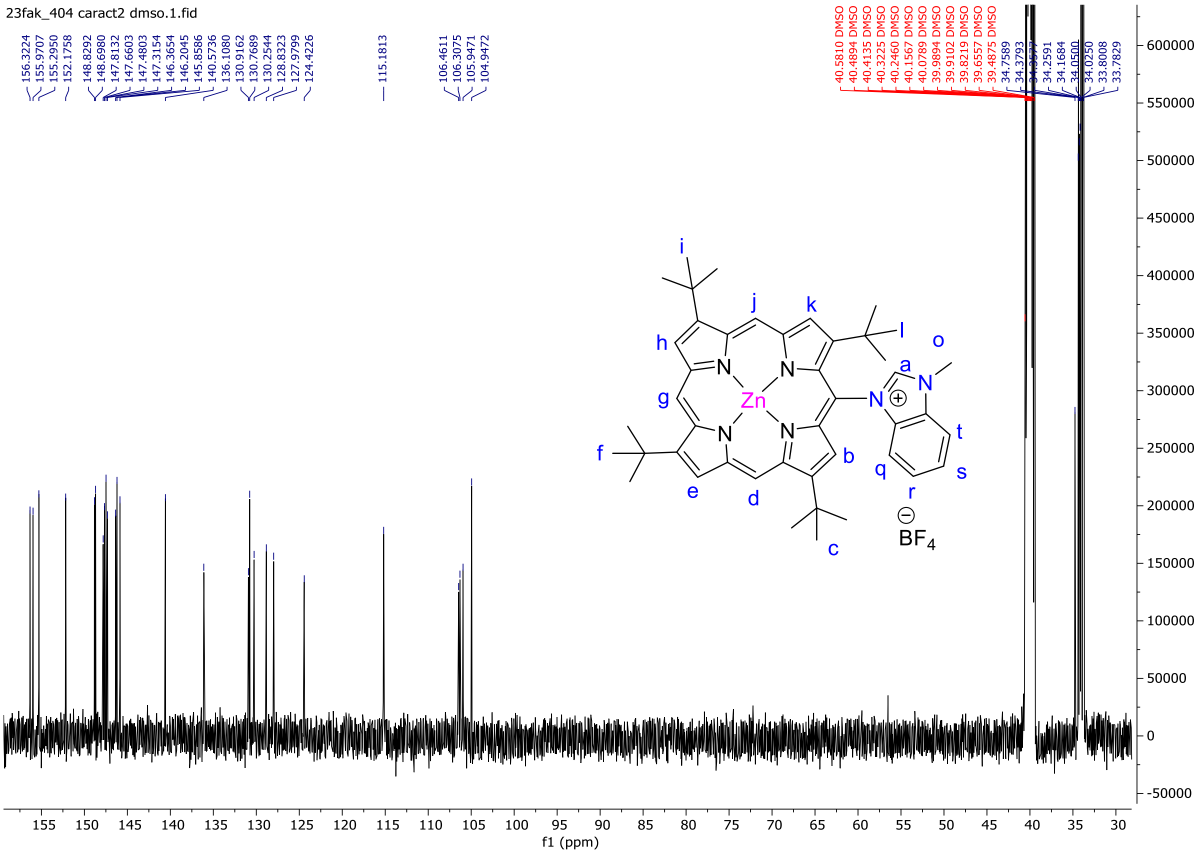

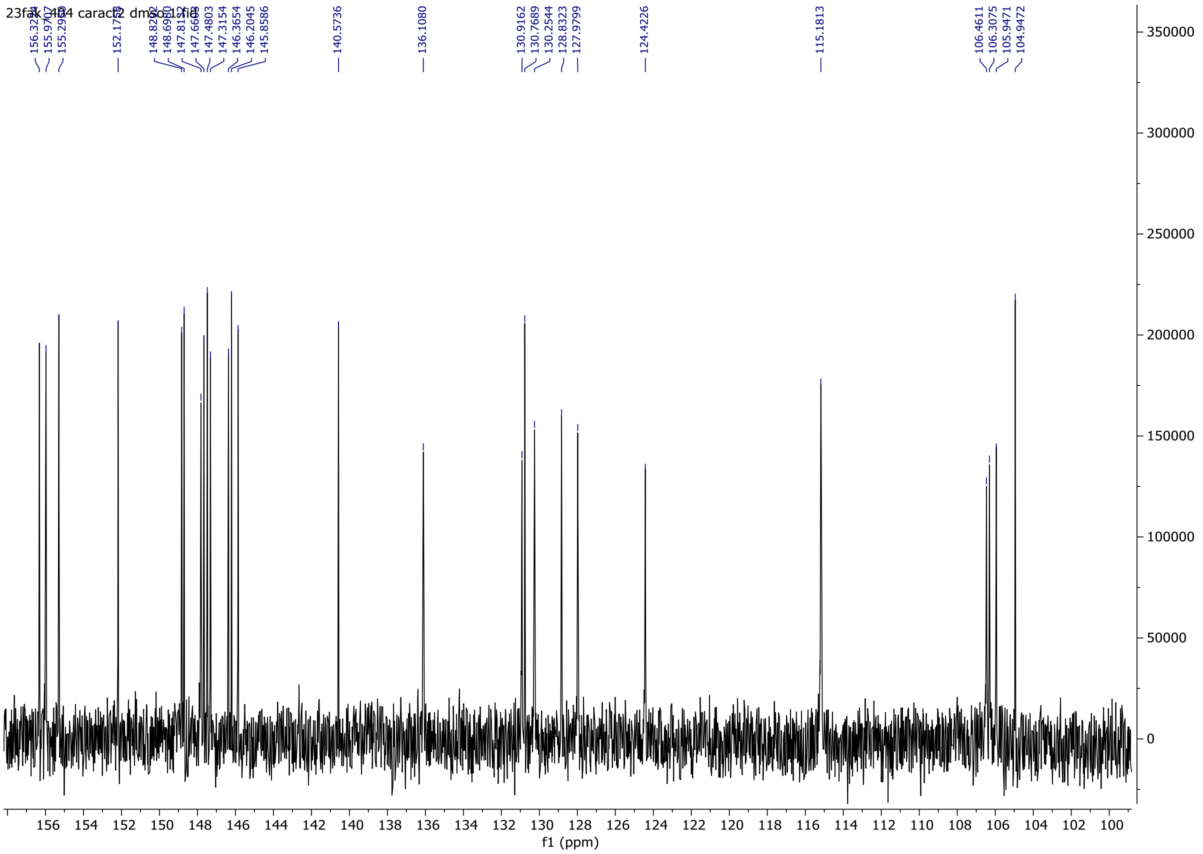

Figure S34 : Full range (top) and partial (bottom) ^{13}C spectra of **Zn-5**$^+$ in $(CD_3)_2SO$, 125 MHz, 298 K.

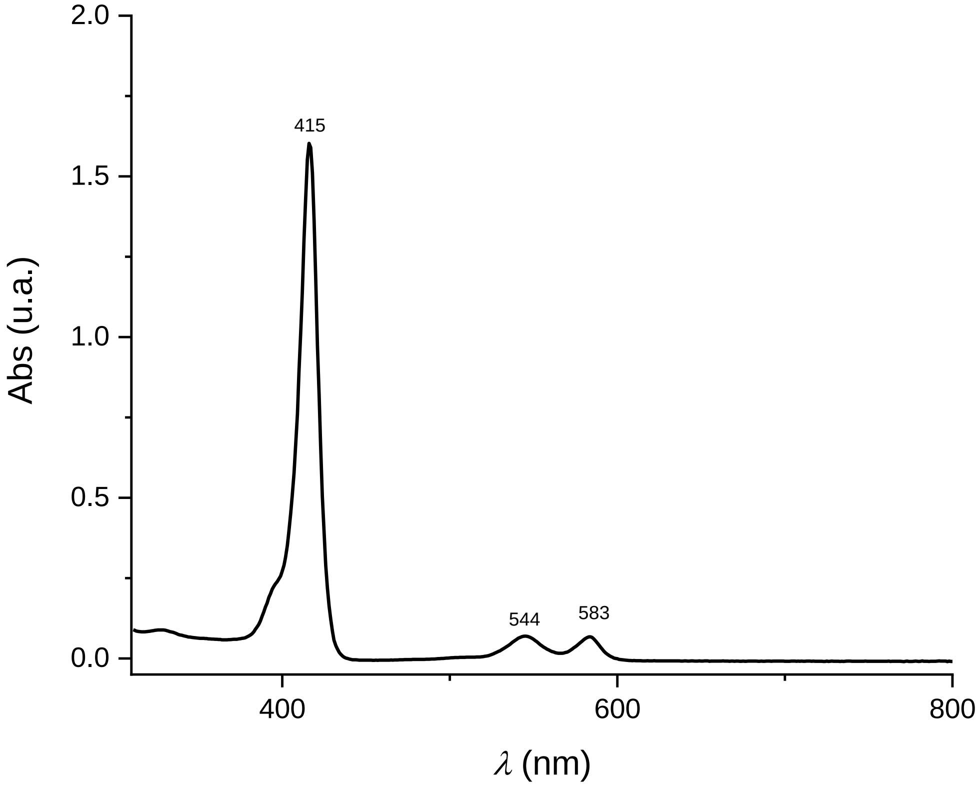

Figure S35. UV-Vis. absorption spectrum of **Zn-5**$^+$ in DMSO, room temperature

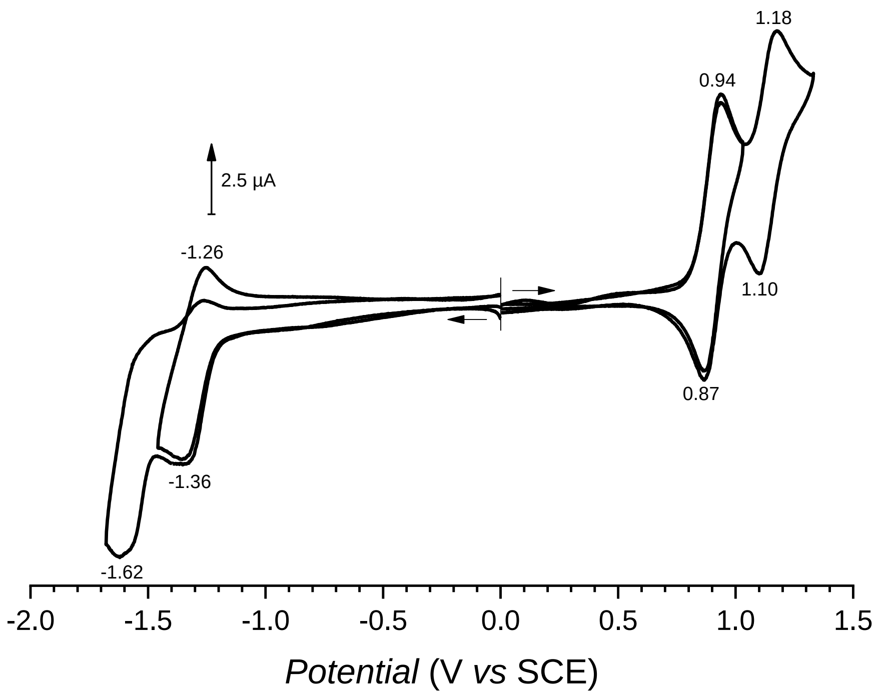

Figure S36. Cyclic voltammogram of compound **Zn-5**$^+$ (C = 10^{-3} M in CH_3CN 0.1 M $TEABF_4$, v = 100 mV.s^{-1}, WE: Pt, Ø = 1.6 mm, CE: Pt, RE: SCE, IUPAC convention).

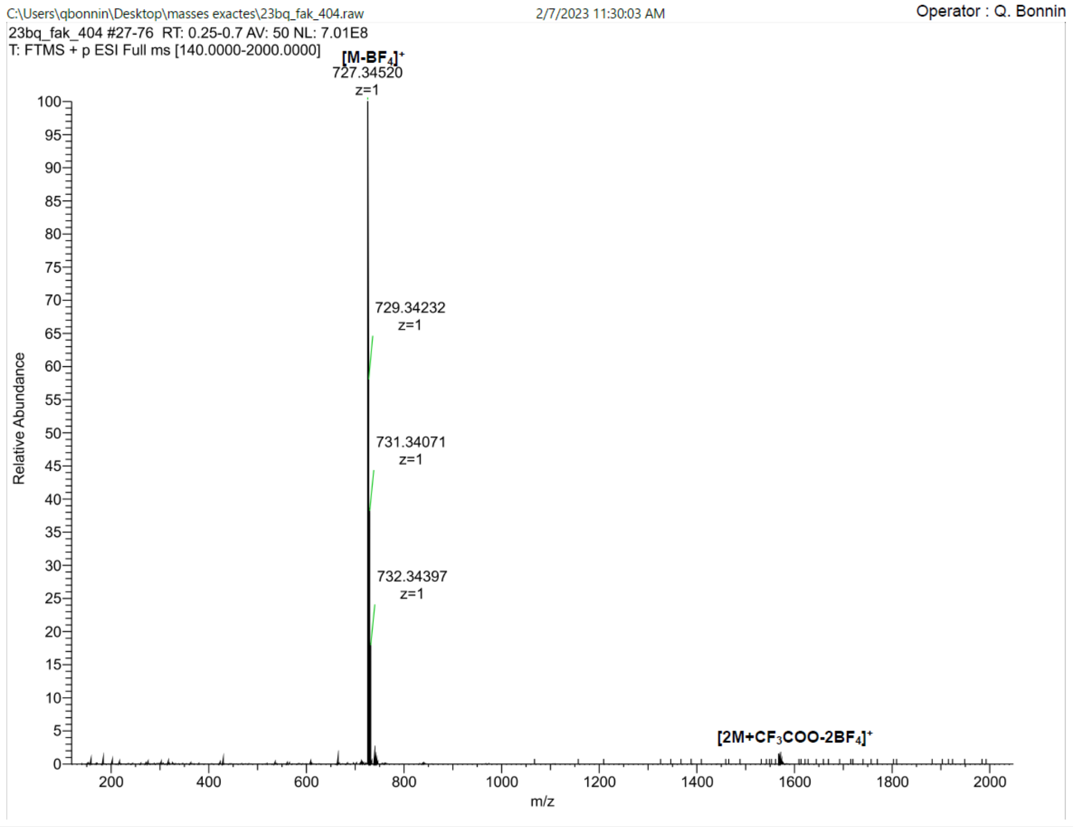

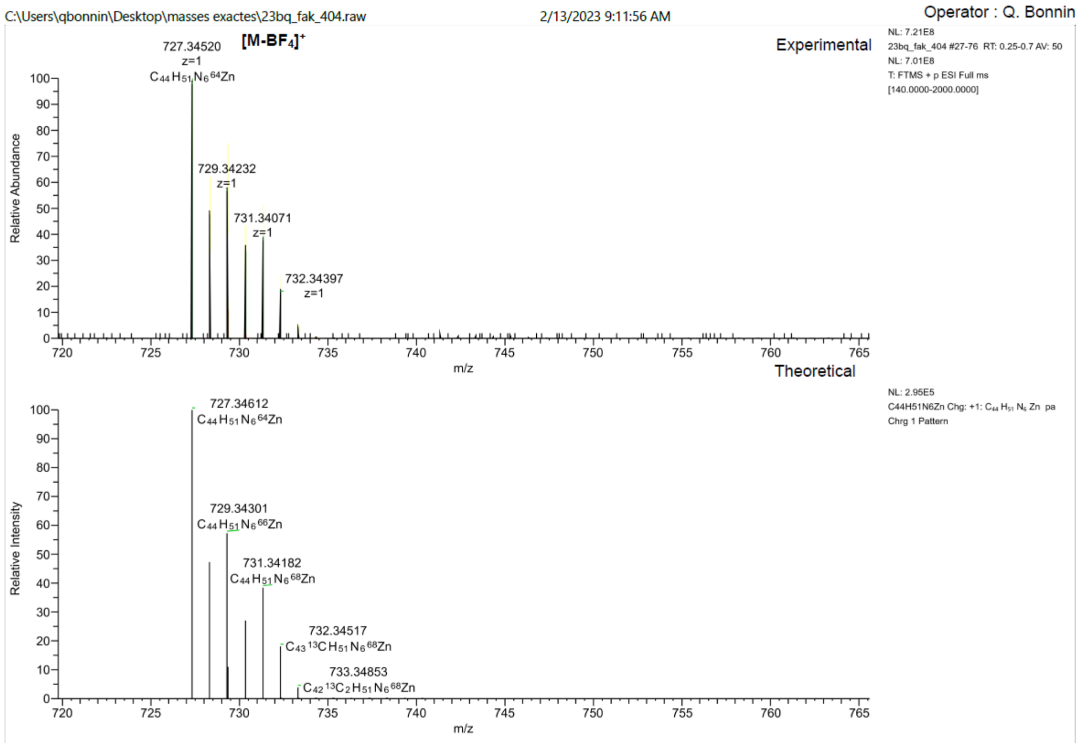

Display Formula	Peak Mass	Theo. mass	Delta [ppm]	RDB	Rank	MSMS Matched Fragments
$C_{44}H_{51}N_6{}^{64}Zn$	727.34520	727.34612	-1.26	22.50	1	(Collection)

Figure S37. High resolution ESI mass spectrum of **Zn-5**$^+$ and simulation of its isotopic pattern.

Electrosynthesis of $Zn\text{-}6^+$

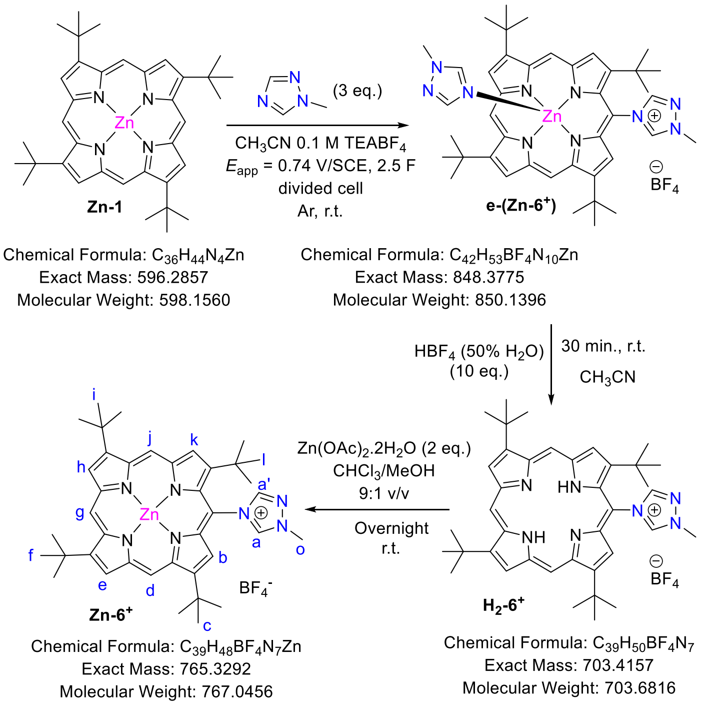

Zn-1 (200.0 mg, 3.34×10^{-1} mmol, 1 eq.) and 1-methyl-1*H*-1,2,4-triazole (57 µL, 1.00 mmol, 3 eq.) were dissolved in CH_3CN (200 mL, 0.1 M $TEABF_4$). The electrolysis was carried out under an argon atmosphere under vigorous stirring at room temperature and at controlled potential (E_{app} = 0.74 V/SCE). Electrolysis was stopped after an uptake of 2.5 F *vs* **Zn-1** and the solvent was removed by rotary evaporation. The crude solid was dissolved in CH_2Cl_2 and washed three times with water (3×200 mL) to remove the supporting electrolyte. The product was purified by column chromatography (SiO_2, CH_2Cl_2 containing 5% CH_3OH). After evaporation of the solvent and NMR analysis, we found that the target product was coordinated by the 1-methyl-1*H*-1,2,4-triazole nucleophile (**a-(Zn-6$^+$)**). This porphyrin was dissolved in CH_3CN and HBF_4 (50% in H_2O, 419 µL, 3.34 mmol, 10 eq. *vs* **Zn-1**) was added to remove the zinc(II) metal (and the coordinated nucleophile). This mixture was stirred at room temperature for 30 min. and the solvents were evaporated. The crude solid was dissolved in CH_2Cl_2 and washed three times with water (3×200 mL). The solvent was then removed and the crude solid was dissolved in 20 mL of $CHCl_3/CH_3OH$ (9:1 v/v) with 2 eq. of $Zn(OAc)_2.2H_2O$ *vs* **Zn-1** (146.6 mg, 6.68×10^{-1} mmol). The reaction was stirred overnight at room temperature, the solvent was evaporated and the crude product was dissolved in CH_2Cl_2 and washed 3 times with H_2O. The product was recrystallized in CH_2Cl_2/*n*-heptane and dried at 100 °C for 2 h to give **Zn-6$^+$** in 68% yield (155.0 mg, 2.27×10^{-1} mmol).

^{1}H NMR ($(CD_3)_2SO$, 500 MHz, 298 K): δ (ppm): 11.40 (s, 1H, Ha), 10.93 (s, 1H, Ha'), 10.69 (s, 1H, Hj), 10.65 (s, 2H, Hd/g), 9.76 (s, 1H, Hk), 9.48 (s, 1H, Hh), 9.43 (s, 1H, He), 7.85 (s, 1H, Hb), 4.58 (s, 1H, Ho), 2.28 (s, 9H, Hi), 2.27 (s, 9H, Hf), 2.18 (s, 9H, Hc), 1.66 (s, 9H, Hl).

$^{13}C\{^{1}H\}$ NMR (($(CD_3)_2SO$, 500 MHz, 298 K): δ (ppm): 156.4, 156.1, 155.4, 152.2, 151.0, 149.2, 148.9, 148.8, 147.7, 147.5, 147.4, 146.3, 145.2, 145.0, 136.0, 131.0, 130.4, 124.1, 106.6, 106.4, 106.2, 103.8, 34.37, 34.35, 34.33, 34.1, 34.04, 34.00, 33.8, 33.7.

λ_{max} (DMSO)/ nm (log ε): 415 (5.39), 545 (4.15), 583 (4.17).

HRMS (ESI+): m/z cald for $[M^+]C_{39}H_{48}N_7Zn^+$ 678.3257, found 678.3251.

Characterization of Zn-6+

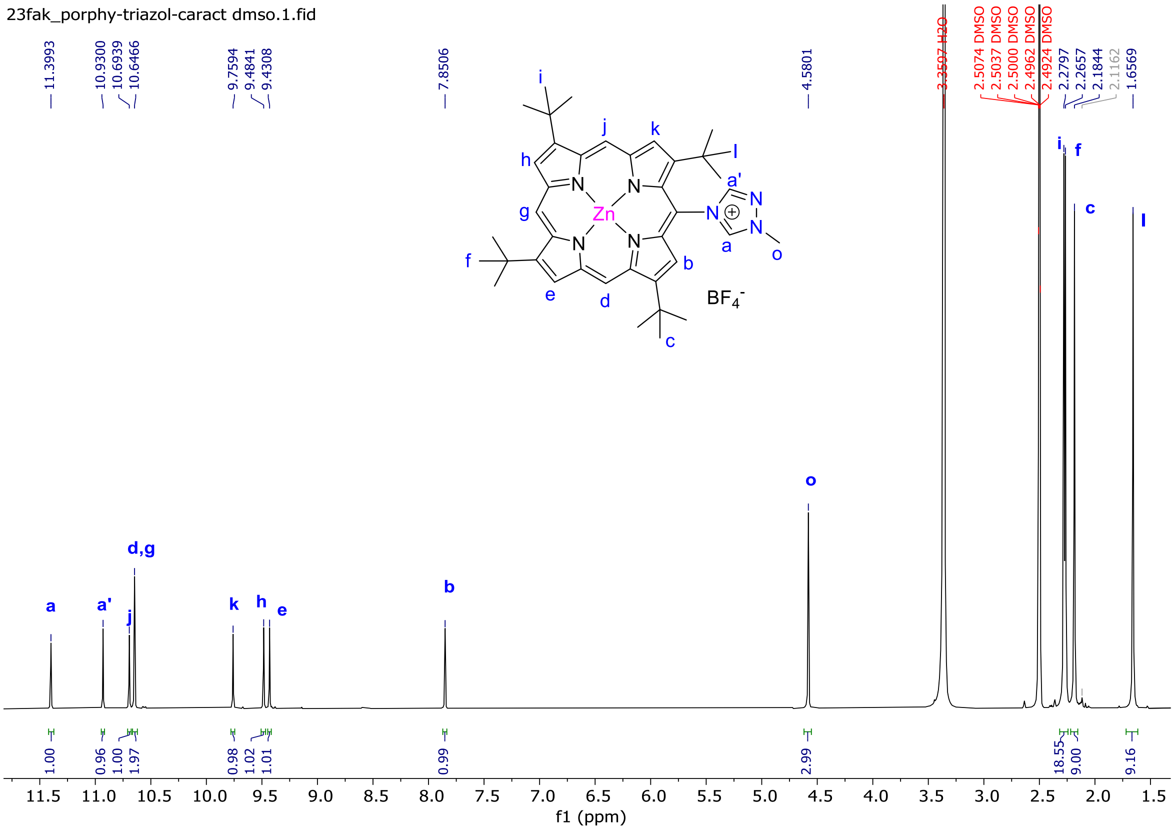

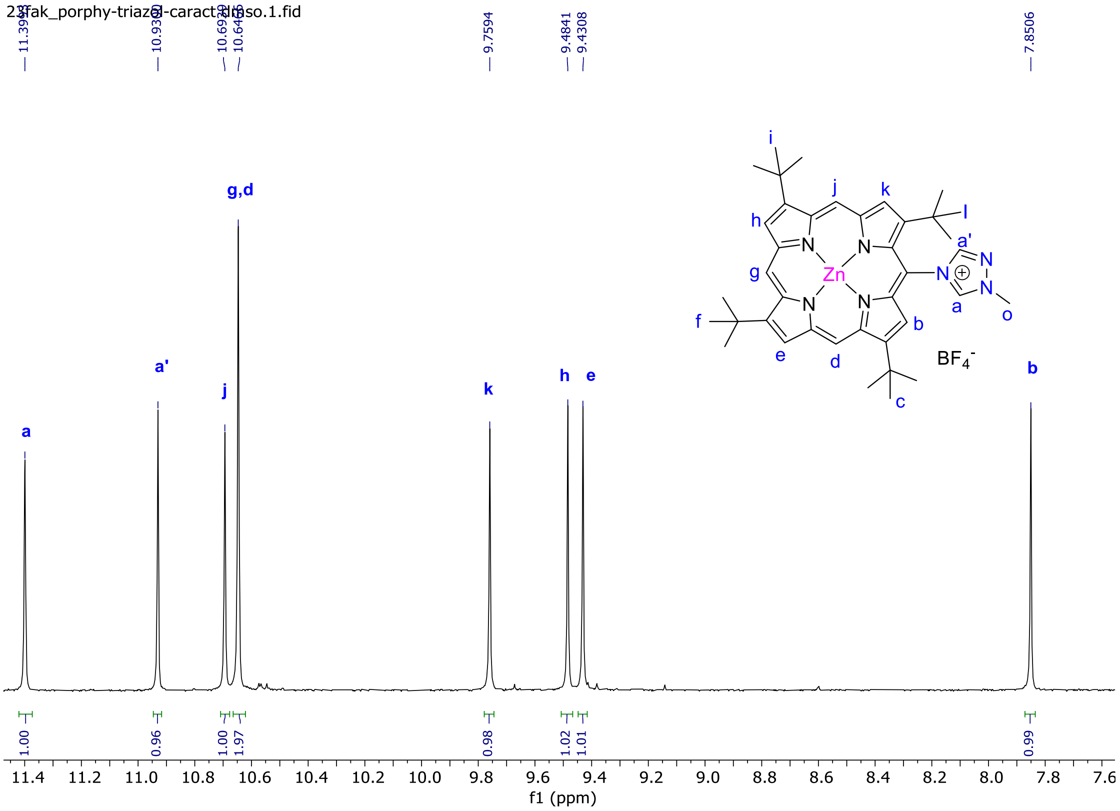

Figure S38. Full range (top) and partial (bottom) ^{1}H NMR spectra of **Zn-6**$^+$ in $(CD_3)_2SO$, 500 MHz, 298 K.

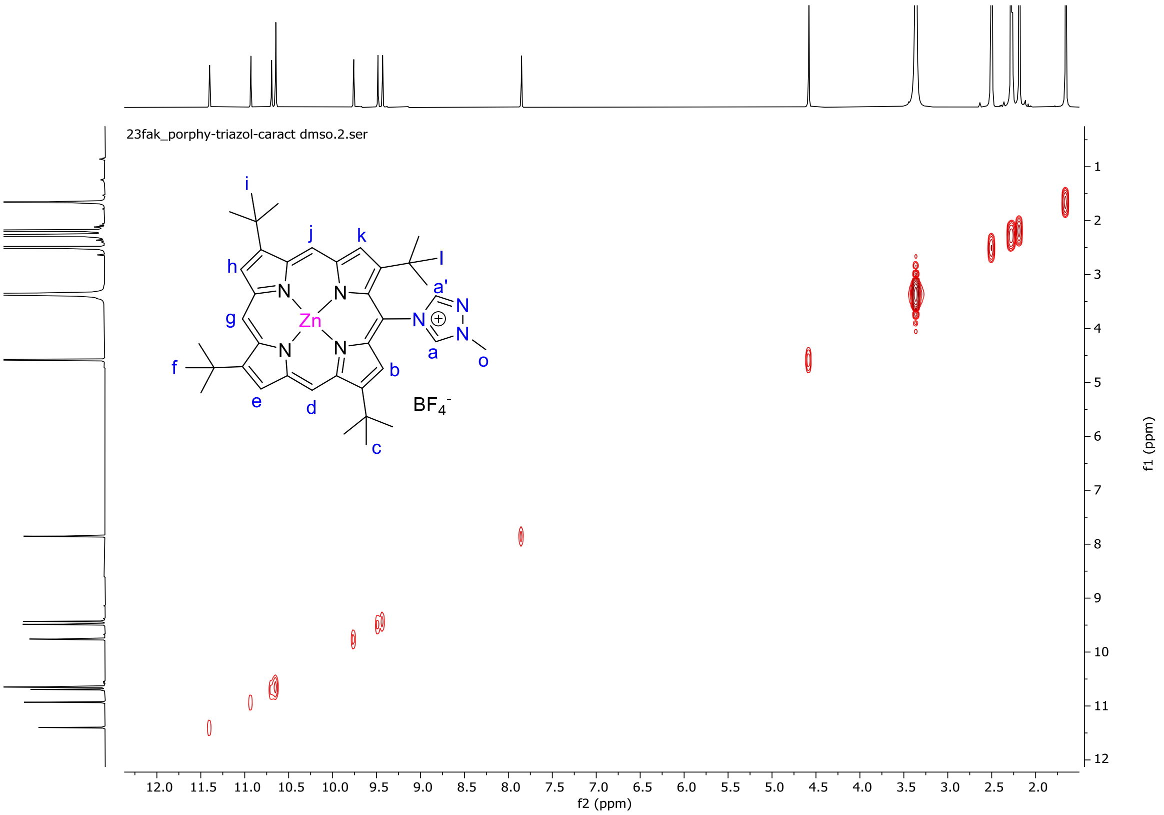

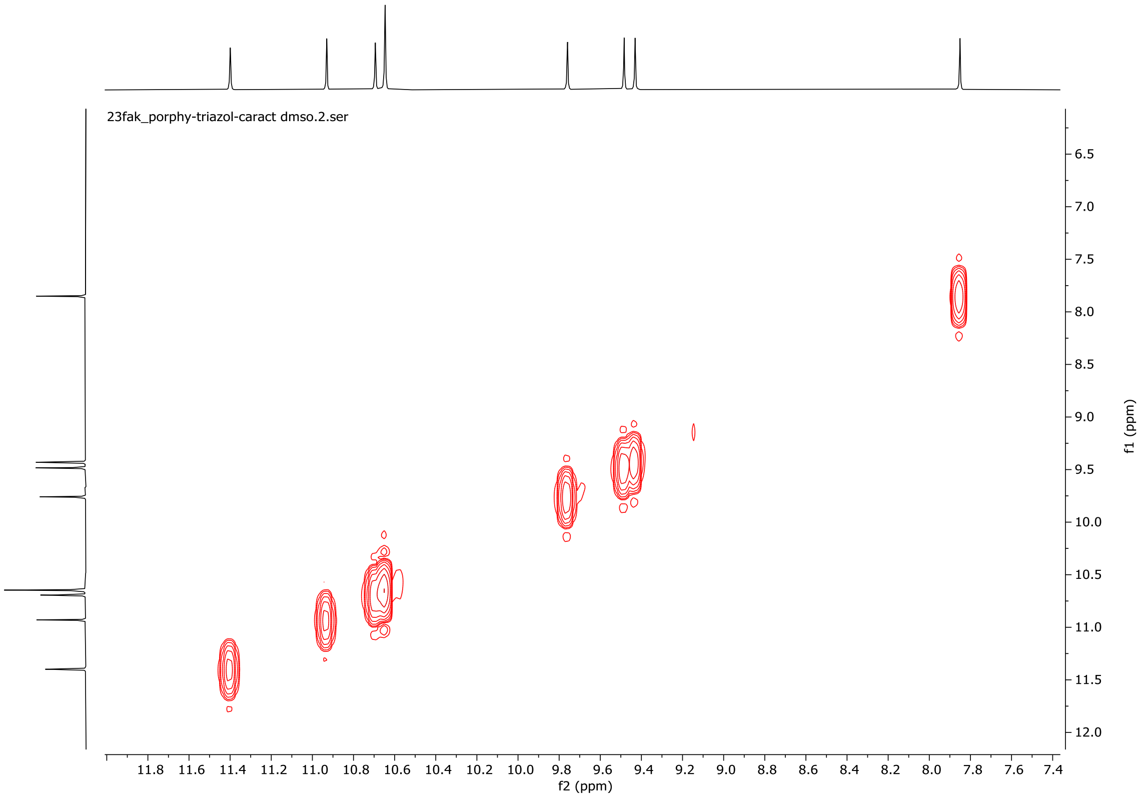

Figure S39. Full range (top) and partial (bottom) ^{1}H-^{1}H COSY spectra of **Zn-6**$^+$ in $(CD_3)_2SO$, 500 MHz, 298 K.

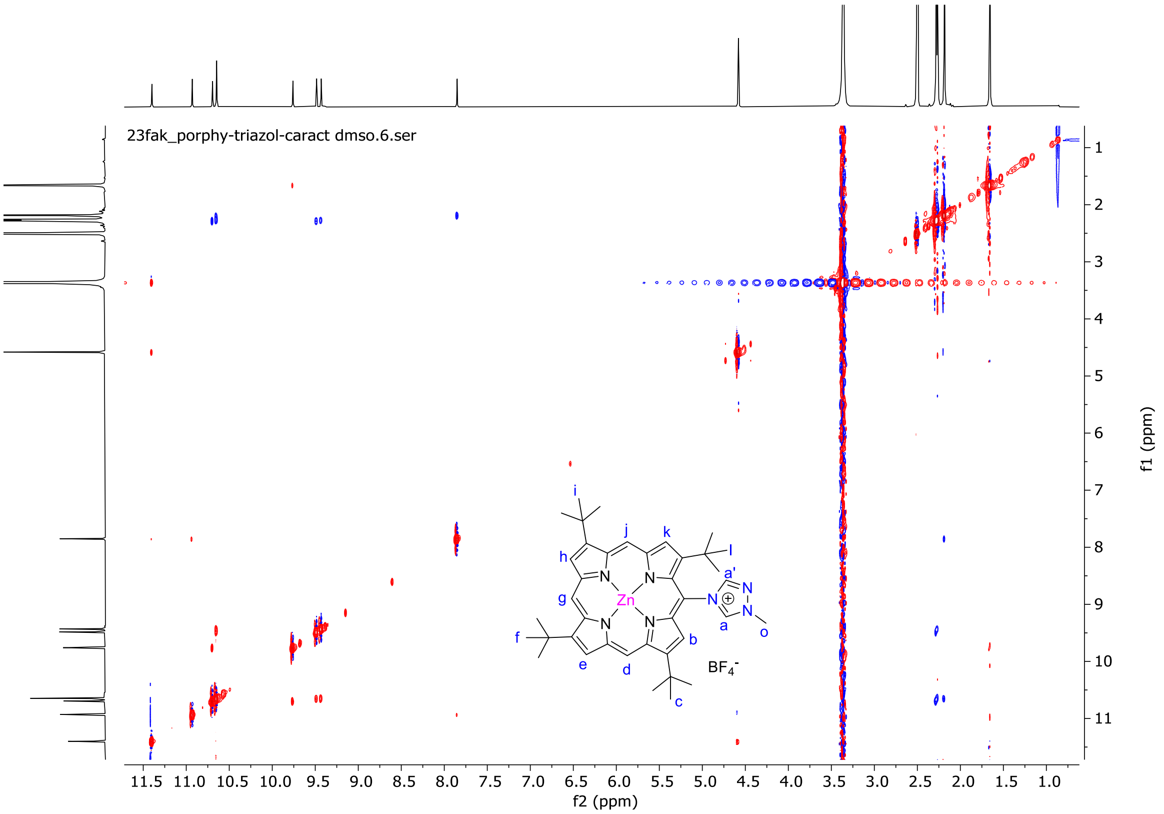

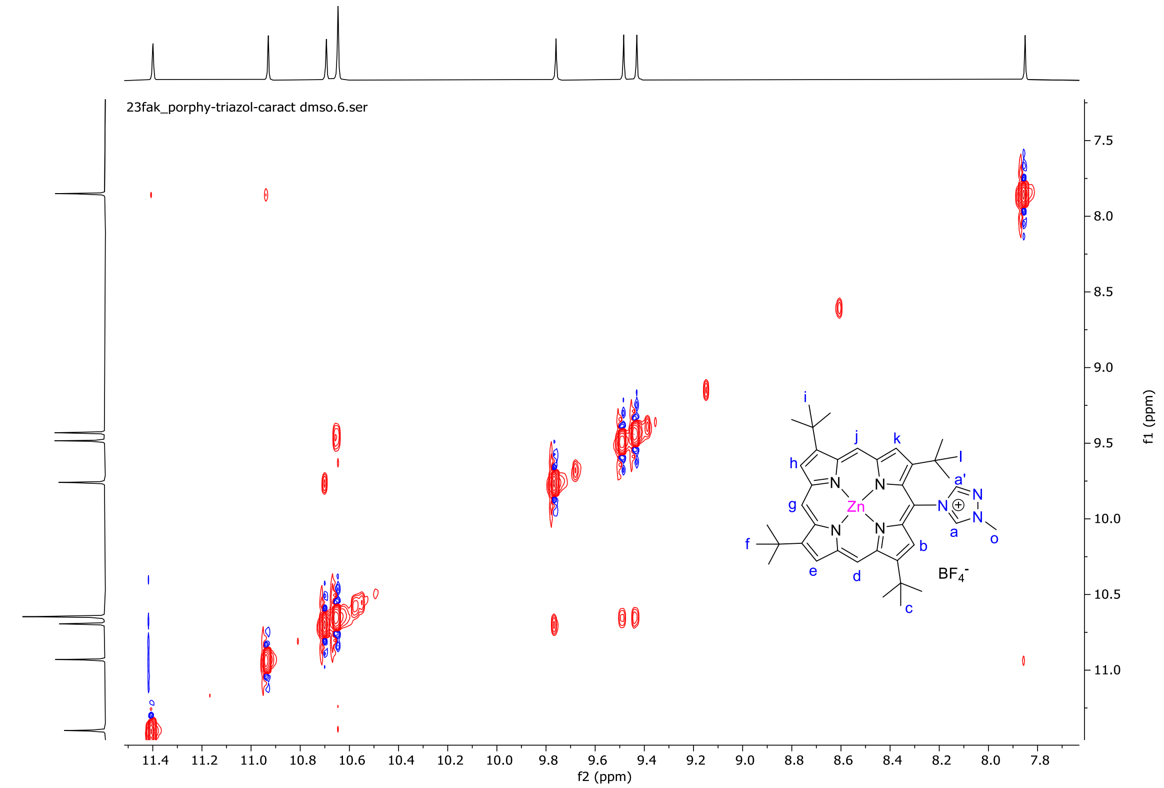

Figure S40. Full range (top) and partial (bottom) ^{1}H-^{1}H NOESY spectra of **Zn-6**$^{+}$ in $(CD_3)_2SO$, 500 MHz, 298 K

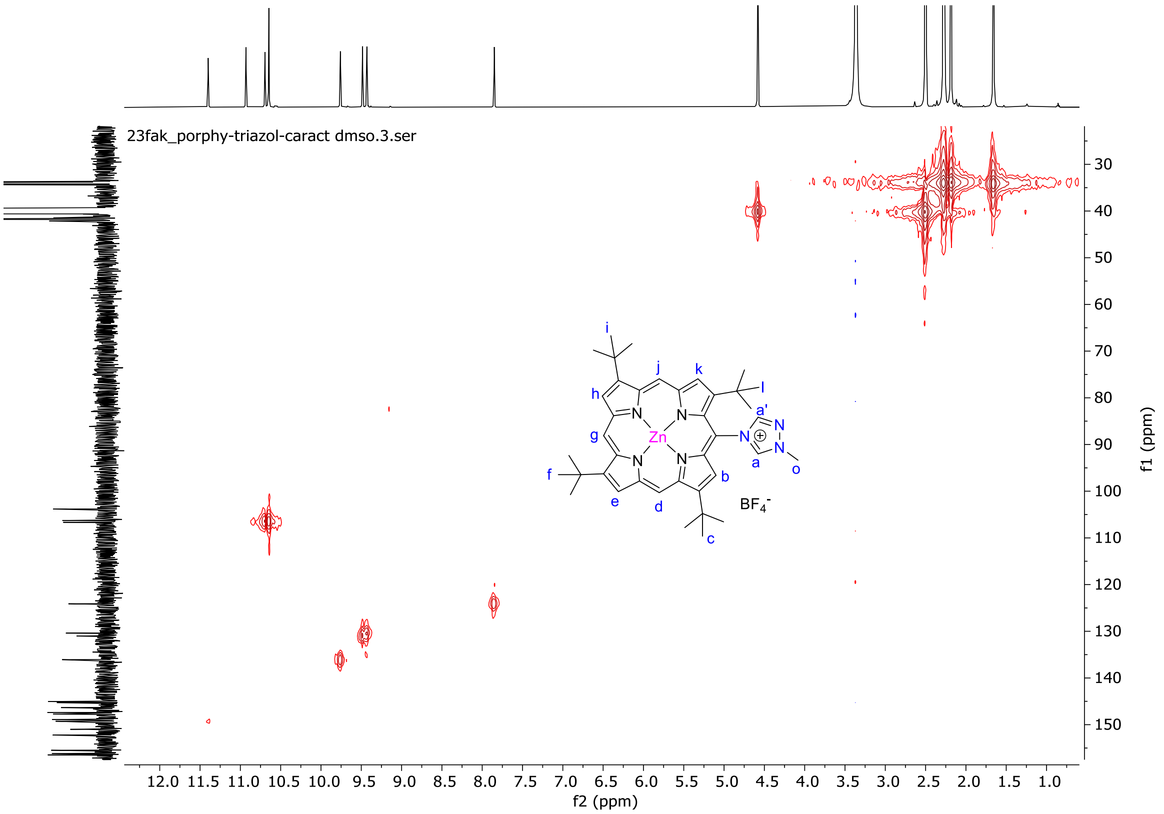

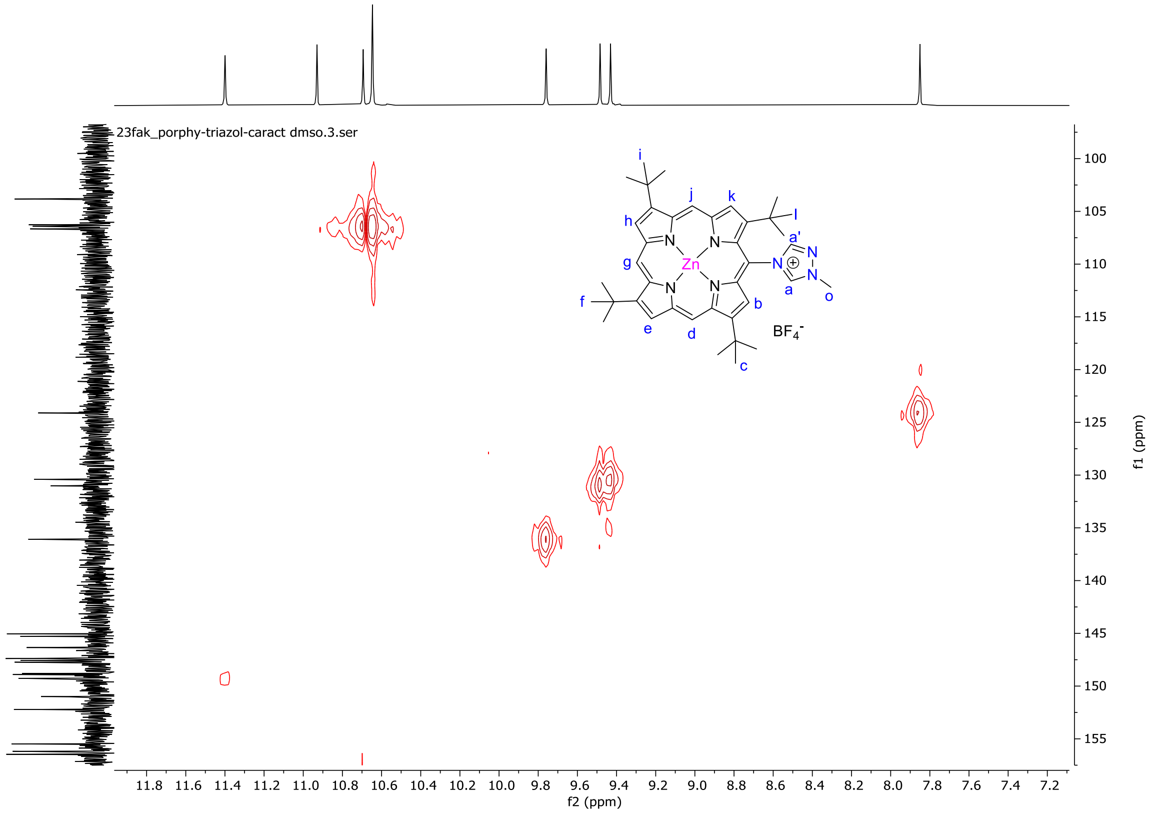

Figure S41. Full range (top) and partial (bottom) ^{1}H-^{13}C HSQC spectra of **Zn-6**$^{+}$ in $(CD_3)_2SO$, 500 MHz, 298 K.

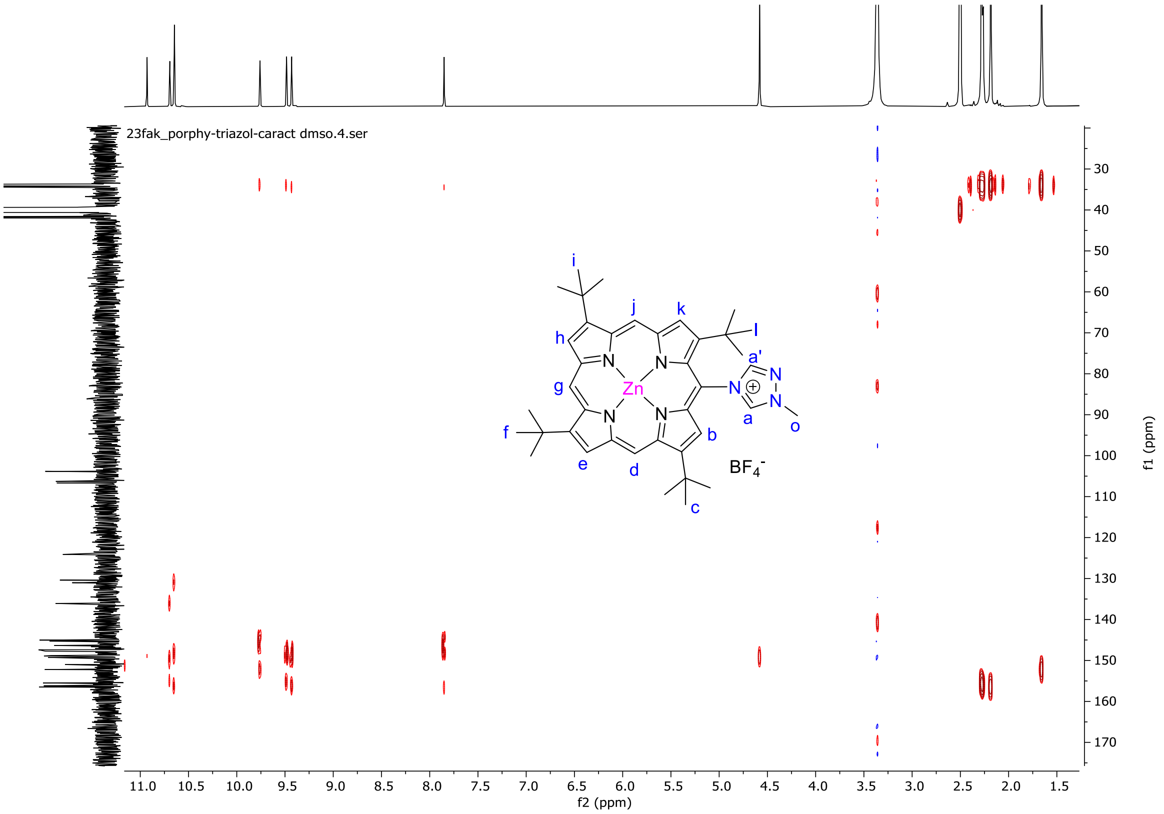

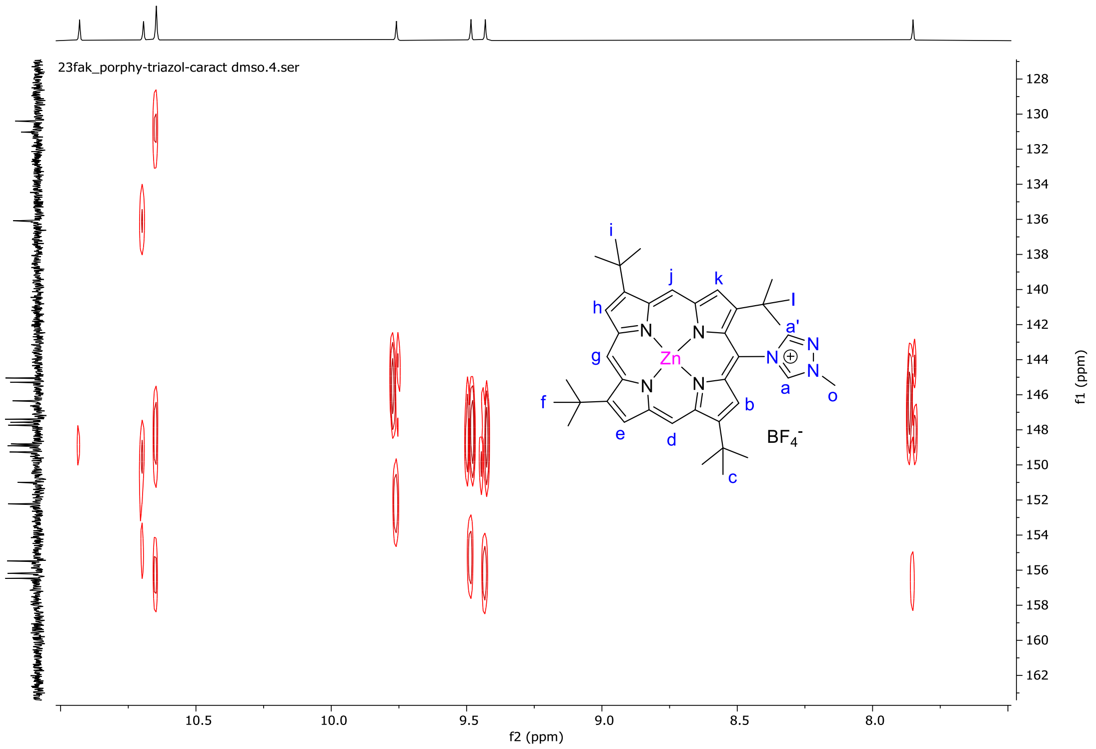

Figure S42. Full range (top) and partial (bottom) ^{1}H-^{13}C HMBC spectra of **Zn-6**$^{+}$ in $(CD_3)_2SO$, 500 MHz, 298 K.

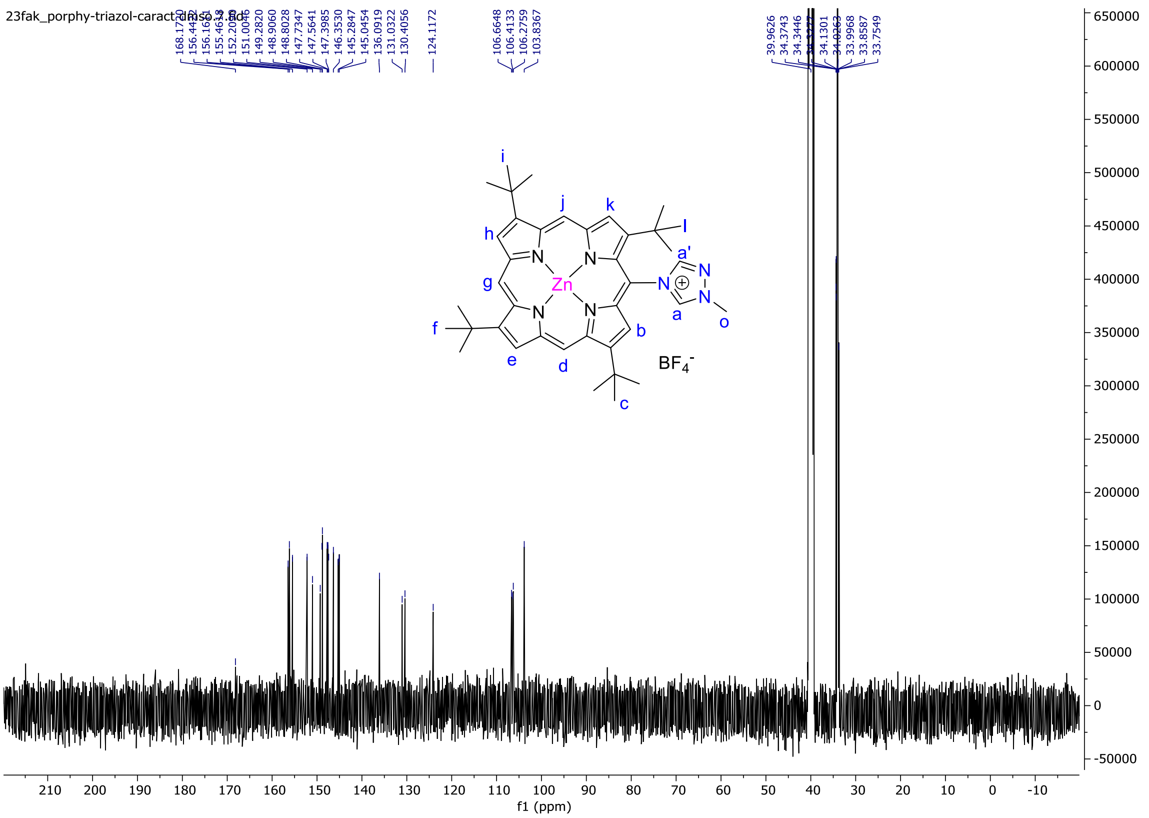

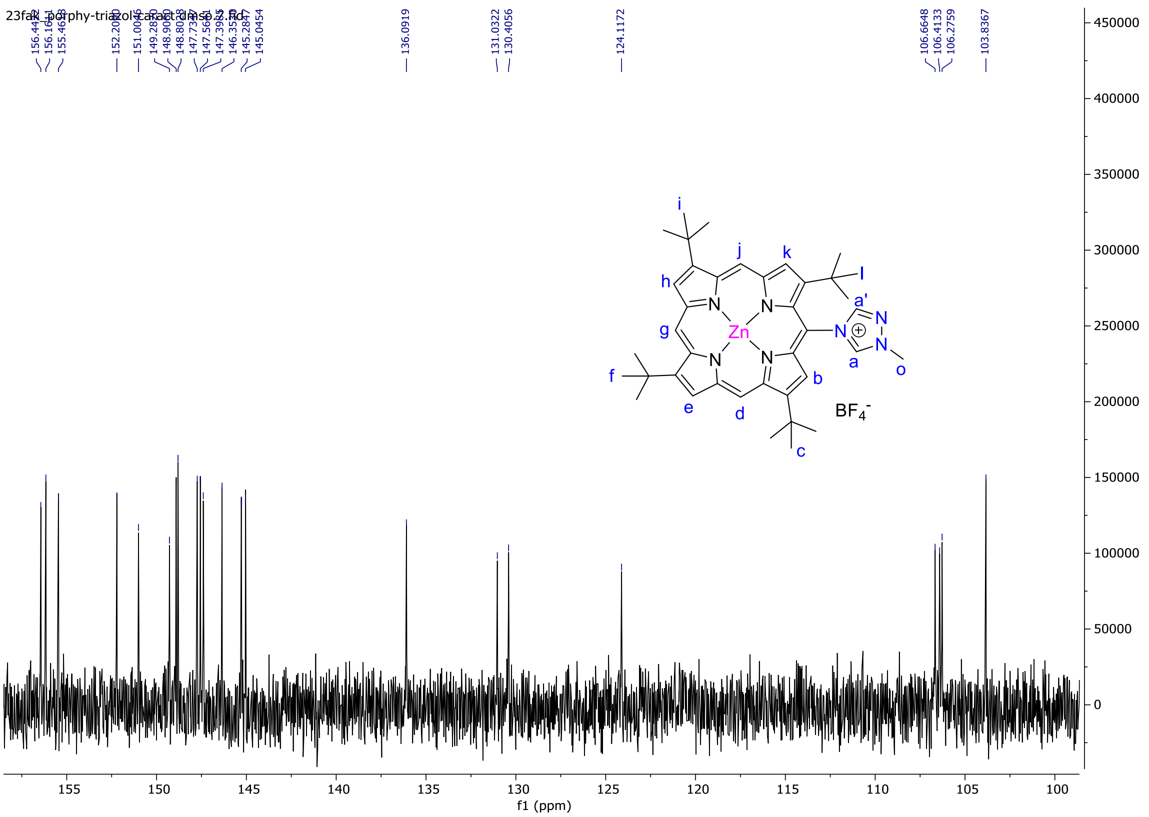

Figure S43. Full range (top) and partial (bottom) ^{13}C spectra of **Zn-6^{+}** in $(CD_3)_2SO$, 125 MHz, 298 K.

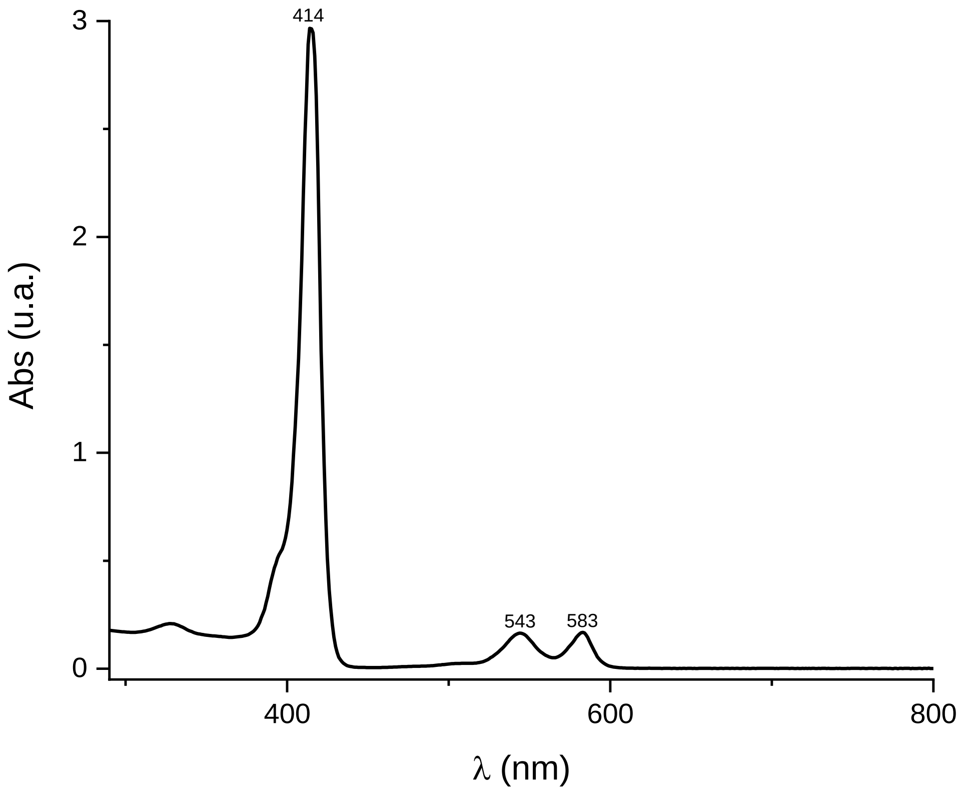

Figure 44. UV-Vis. absorption spectrum of **Zn-6**$^+$ in DMSO, room temperature.

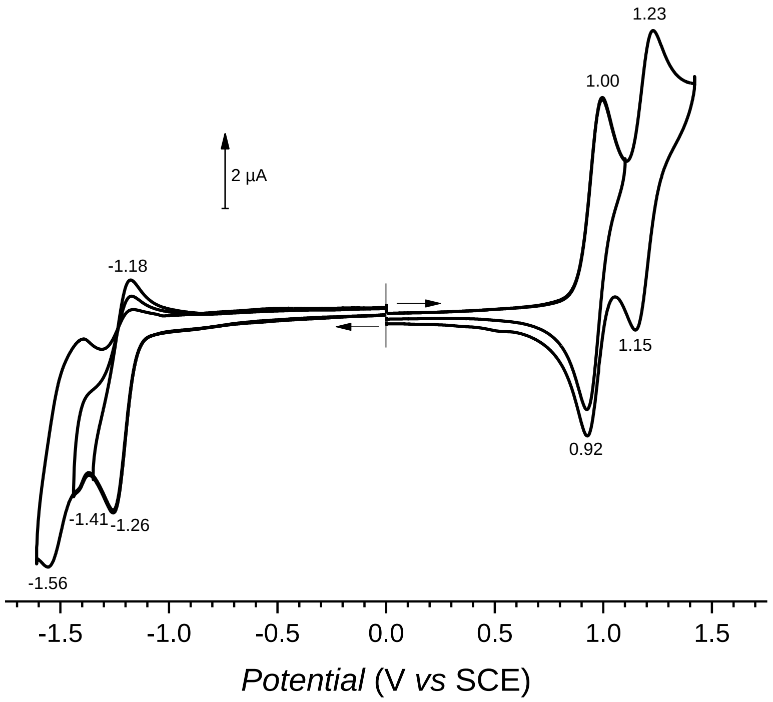

Figure S45. Cyclic voltammogram of compound **Zn-6**$^+$ ($C = 10^{-3}$ M in CH_3CN 0.1 M $TEABF_4$, v = 100 mV.s^{-1}, WE: Pt, Ø = 1.6 mm, CE: Pt, RE: SCE, IUPAC convention).

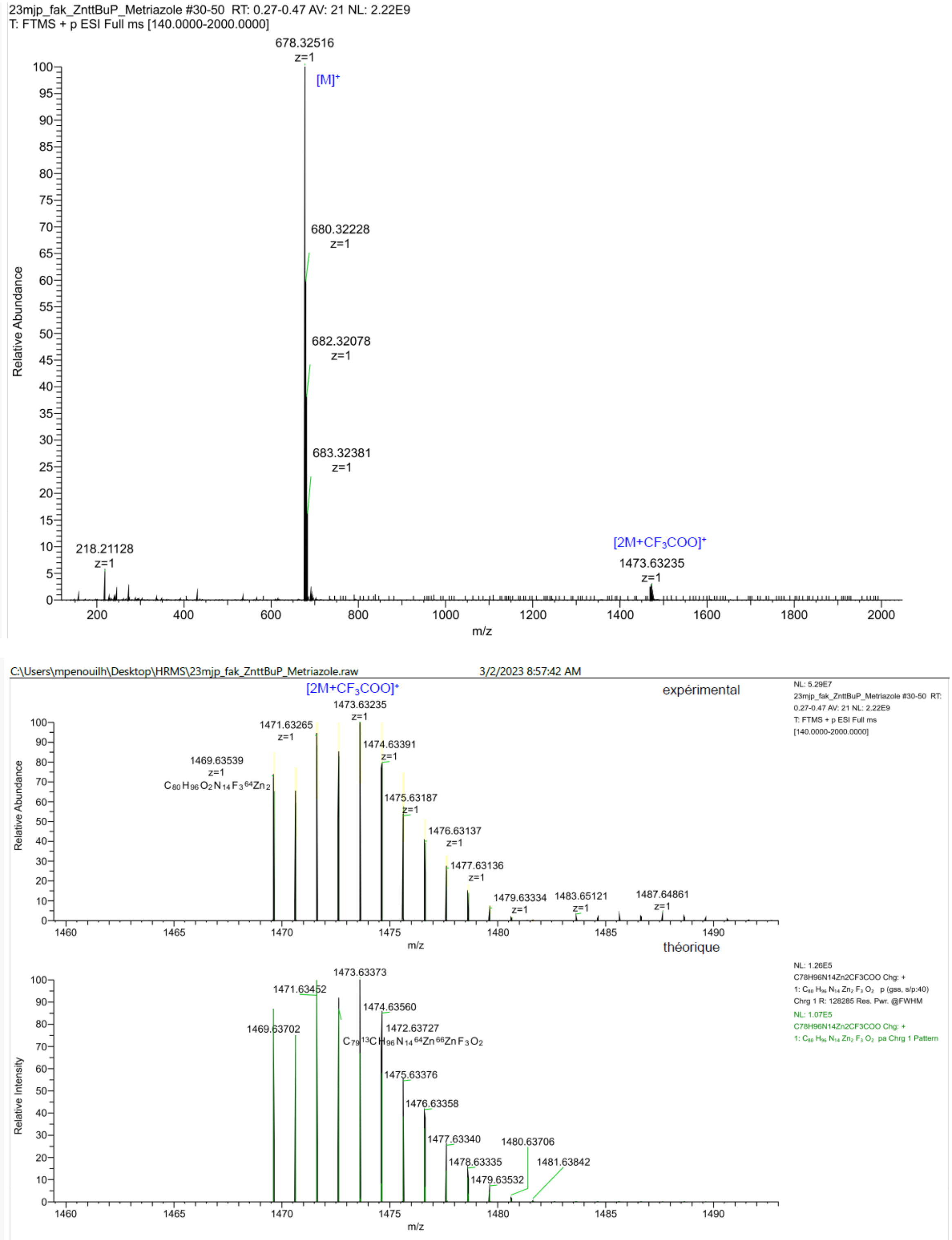

Peak Mass	Display Formula	RDB	Delta [ppm]	Theo. mass	Rank	Pattern Cov. [%]	MSMS Matched Fragm...
1469.63539	$C_{80}H_{96}O_2N_{14}F_3{}^{64}Zn_2$	38.50	-1.11	1469.63702	1	99.92	(Collection)

Figure S46. High resolution ESI mass spectrum of **Zn-6**$^+$ and simulation of its isotopic pattern.

Electrosynthesis of f-(Zn-7$^+$)

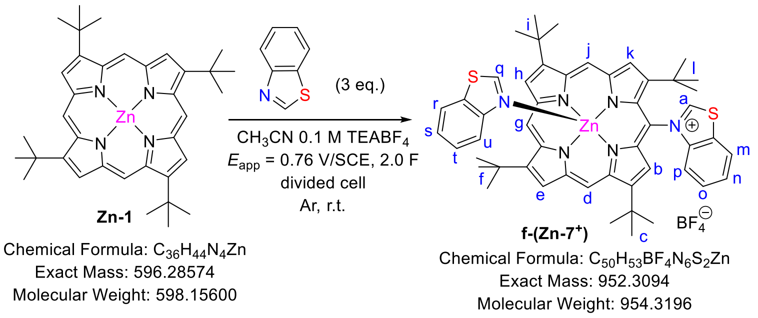

Zn-1 (100.0 mg, 1.67×10^{-2} mmol, 1 eq.), and 1-vinylimidazole (55 µL, 5.05×10^{-1} mmol, 3 eq.) was dissolved in CH_3CN (100 mL, 0.1 M $TEABF_4$). The electrolysis was carried out under an argon atmosphere under vigorous stirring at room temperature and at controlled potential (E_{app} = 0.76 V/SCE). Electrolysis was stopped after an uptake of 2.0 F *vs* **Zn-1** and the solvent was removed by rotary evaporation. The crude solid was dissolved in CH_2Cl_2 and washed three times (3×200 mL) with water to remove the supporting electrolyte. The product was recrystallized in CH_2Cl_2/*n*-heptane and dried at 40 °C for 12 h to give **f-(Zn-7$^+$)** in 97% yield (141.0 mg, 1.48×10^{-1} mmol, the molecular weight of **f-(Zn-7$^+$)** was calculated with 1 eq. of benzothiazole, in agreement with the ^{1}H NMR: MW = 954.3196 g mol^{-1}).

^{1}H NMR ($(CD_3)_2SO$, 500 MHz, 298 K): δ (ppm): 12.75 (s, 1H, Ha), 10.73 (s, 1H, Hj), 10.67 (s, 1H, Hg), 10.61 (s, 1H, Hd), 9.74 (s, 1H, Hk), 9.51 (s, 1H, Hh), 9.42 (s, 1H, He), 9.39 (s, 1H, Hq), 8.82 (d, $^3J_{H-H}$ = 8.4 Hz, 1H, Hm), 8.18 (d, $^3J_{H-H}$ = 8.0 Hz, 1H, Hu or Hr), 8.09 (d, $^3J_{H-H}$ = 9.0 Hz, 1H, Hr or Hu), 7.84 (dd, $^3J_{H-H}$ = 8.4 and 8.7 Hz, 1H, Hn), 7.55 (s+dd, the dd looks like a triplet, coupling constant difficult to measure precisely, 2H, Hb and Hs or Ht), 7.49 (dd looks like a triplet, coupling constant difficult to measure precisely, 1H, Ht or Hs), 7.40 (dd looks like a triplet, coupling constant difficult to measure precisely, 1H, Ho), 6.00 (d, $^3J_{H-H}$ = 8.7 Hz, 1H, Hp), 2.29 (s, 9H, Hi), 2.27 (s, 9H, Hf), 2.04 (s, 1H, Hc), 1.42 (s, 1H, Hl).

$^{13}C\{^1H\}$ NMR ($(CD_3)_2SO$, 125 MHz, 298 K): 170.04, 156.80, 156.52, 156.25, 155.53, 153.48, 151.79, 150.01, 149.02, 148.88, 147.95, 147.72, 147.53, 146.44, 145.14, 144.98, 136.46, 134.00, 131.36, 131.08, 130.43, 129.55, 129.20, 126.64, 126.32, 125.94, 123.68, 123.49, 122.97, 118.26, 107.91, 106.80, 106.25, 34.38, 34.37, 34.20, 34.01, 34.00, 33.68, 33.61, 31.72, 28.83, 28.83, 22.56, 14.42.

λ_{max} (DMSO)/ nm (log ε): 416 (5.35), 547 (4.23), 585 (4.21).

HRMS (ESI+): m/z cald for [M-BF_4^-] 730.2916, found 730.2913.

Characterization of f-(Zn-7+)

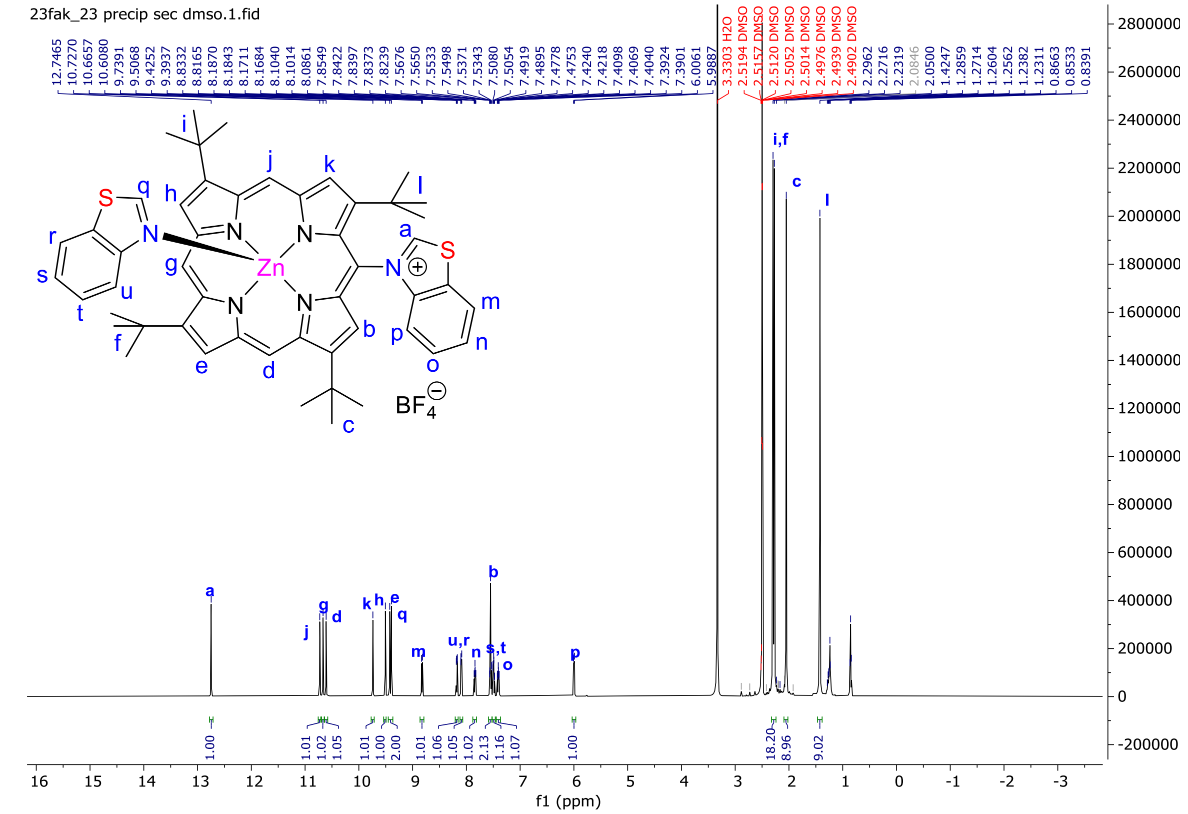

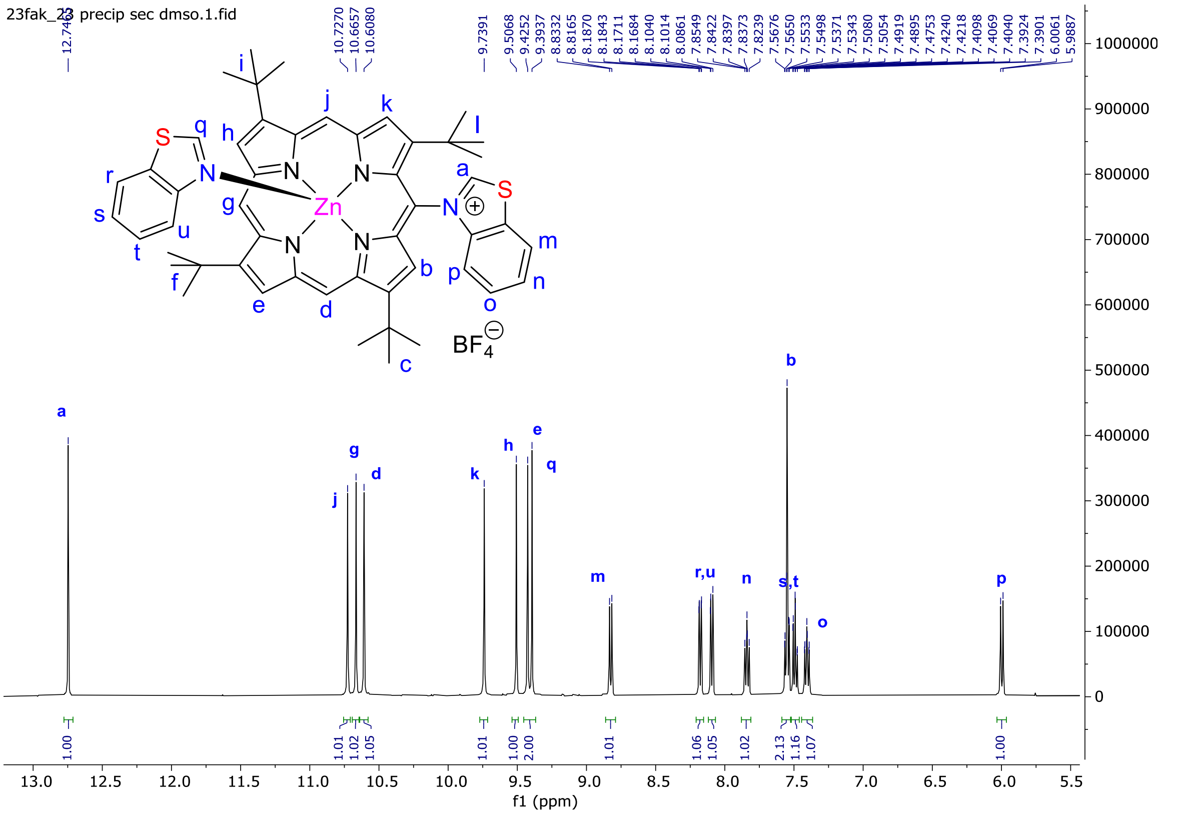

Figure S47 : Full range (top) and partial (bottom) ^{1}H NMR spectra of **f-(Zn-7$^+$)** in $(CD_3)_2SO$, 500 MHz, 298 K.

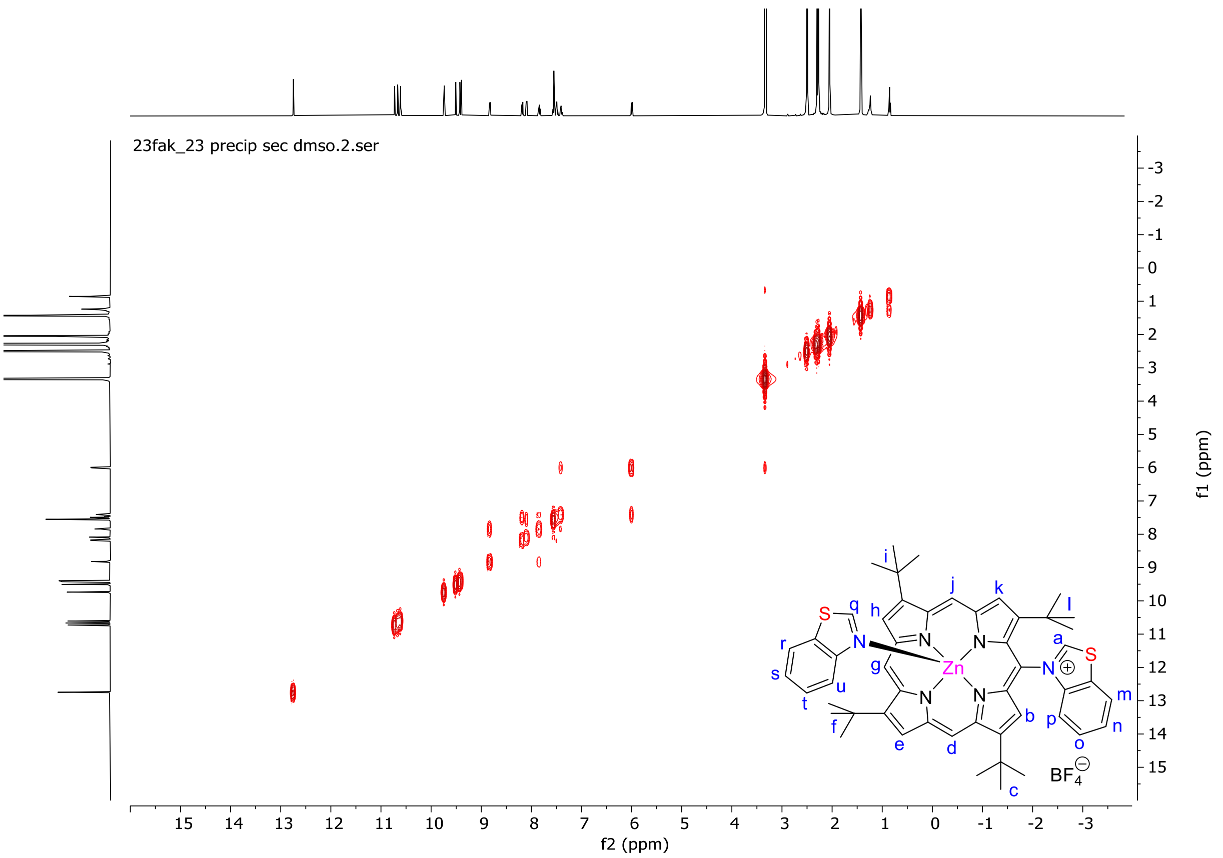

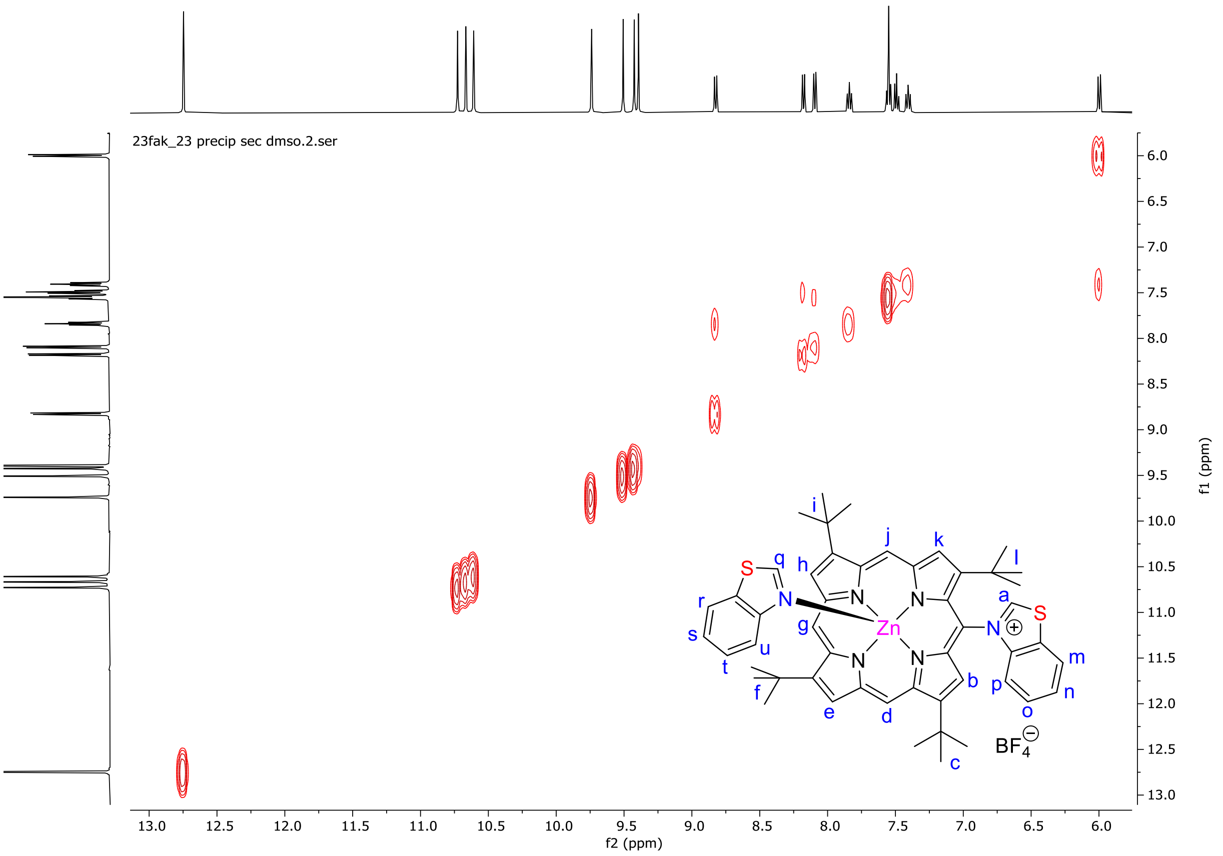

Figure S48 : Full range (top) and partial (bottom) ^{1}H-^{1}H COSY spectra of **f-(Zn-7$^+$)** in $(CD_3)_2SO$, 500 MHz, 298 K.

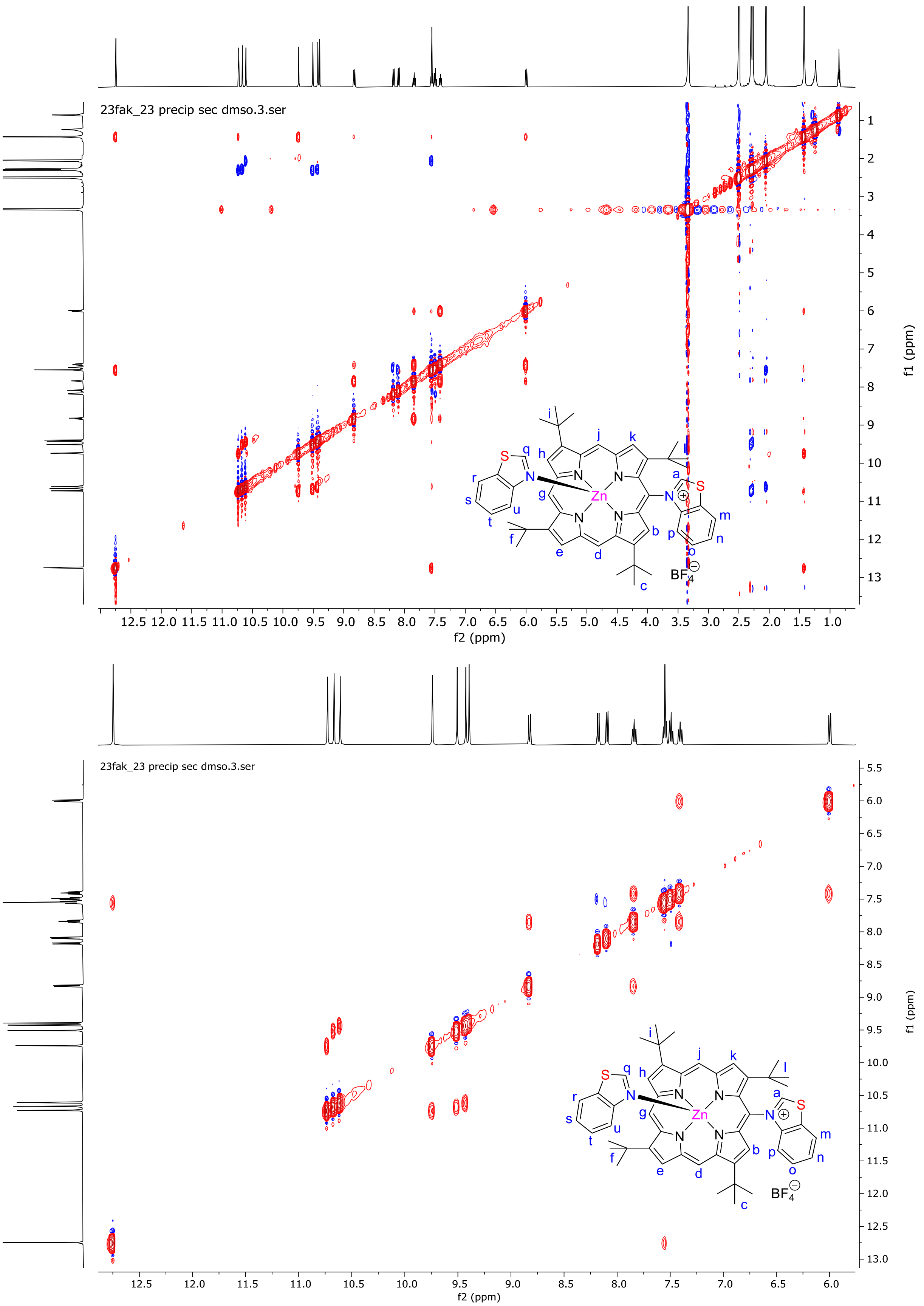

Figure S49 : Full range (top) and partial (bottom) ^{1}H-^{1}H NOESY spectra of **f-(Zn-7^{+})** in $(CD_3)_2SO$, 500 MHz, 298 K.

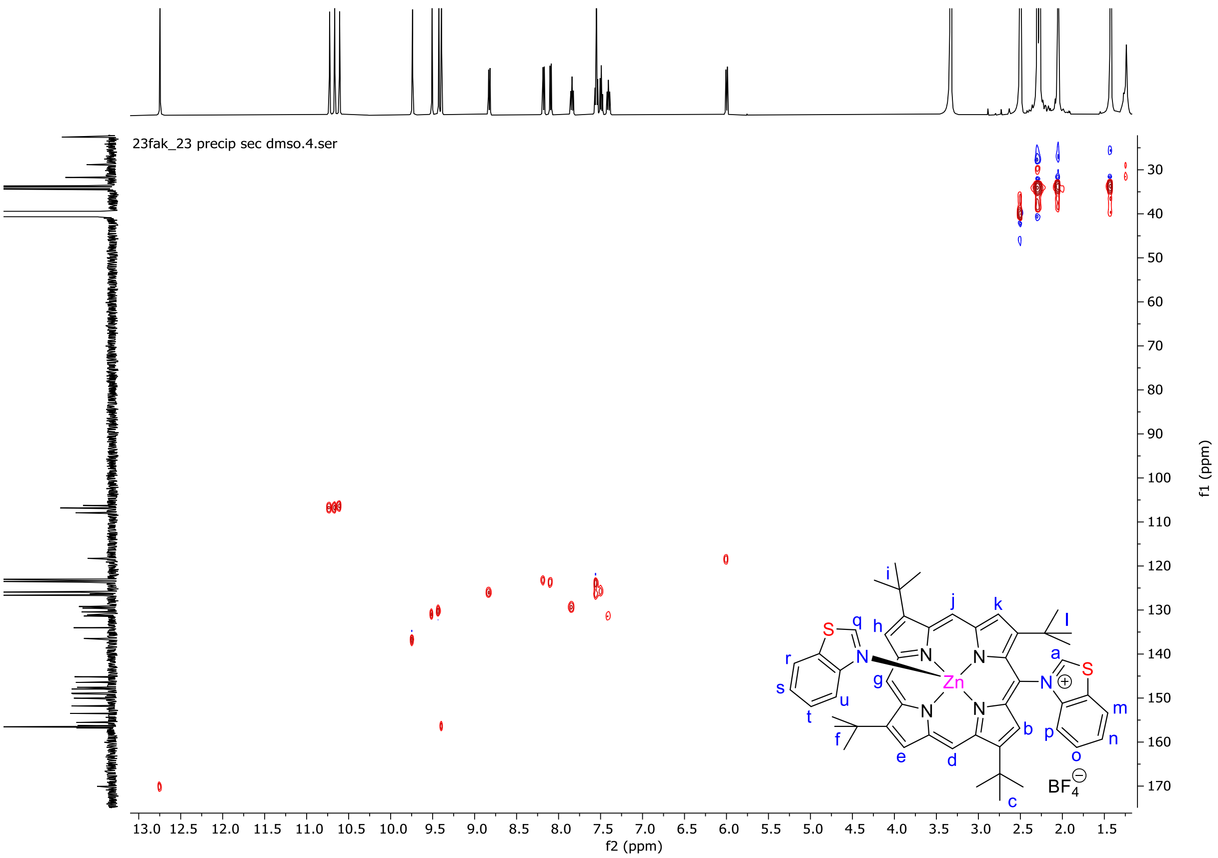

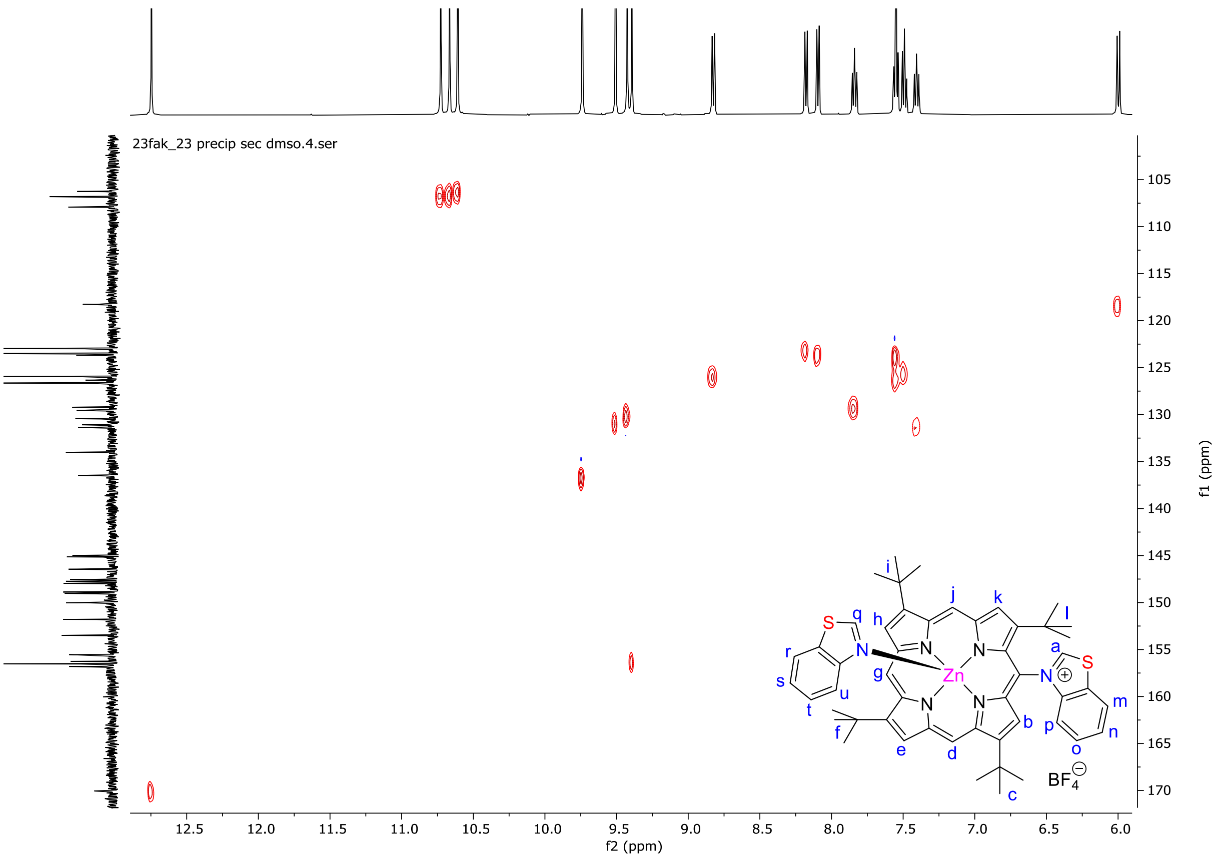

Figure S50 : Full range (top) and partial (bottom) ^{1}H-^{13}C HSQC spectra of **f-(Zn-7⁺)** in $(CD_3)_2SO$, 500 MHz, 298 K.

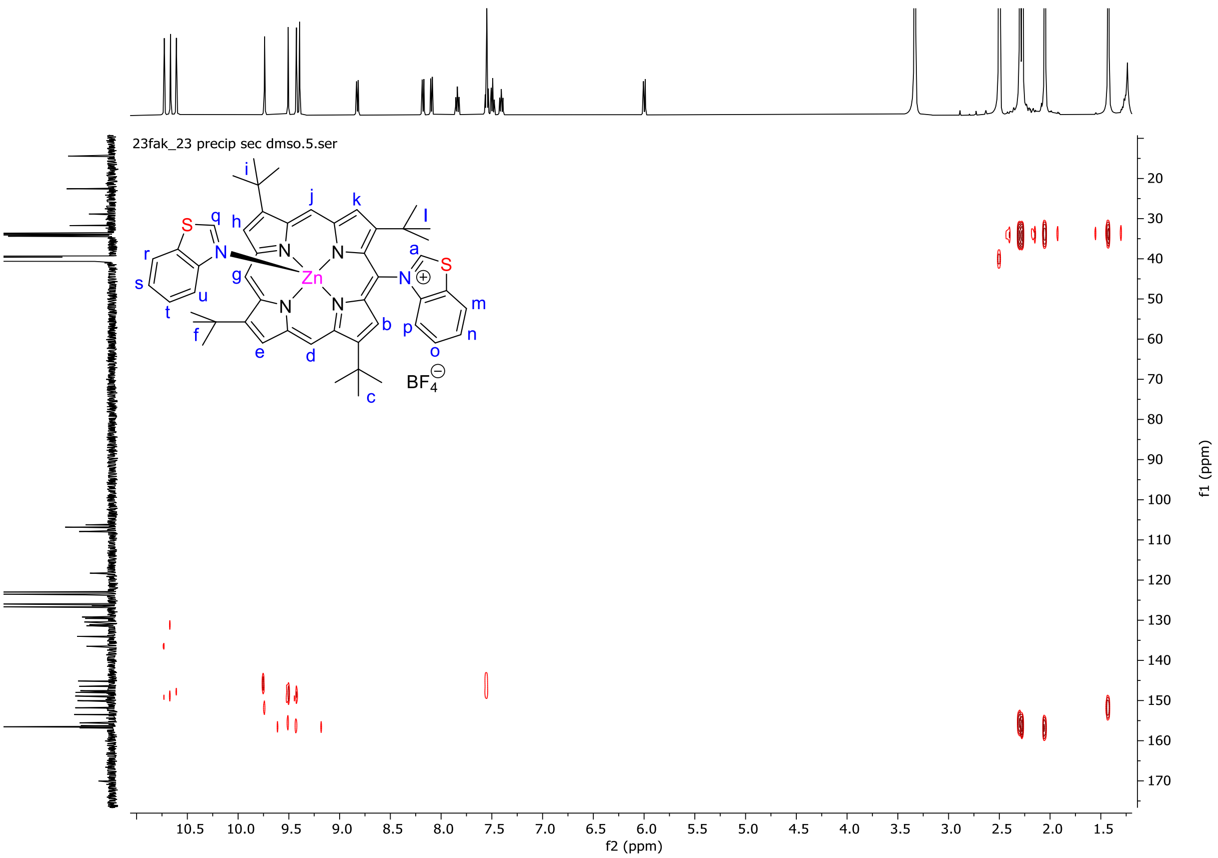

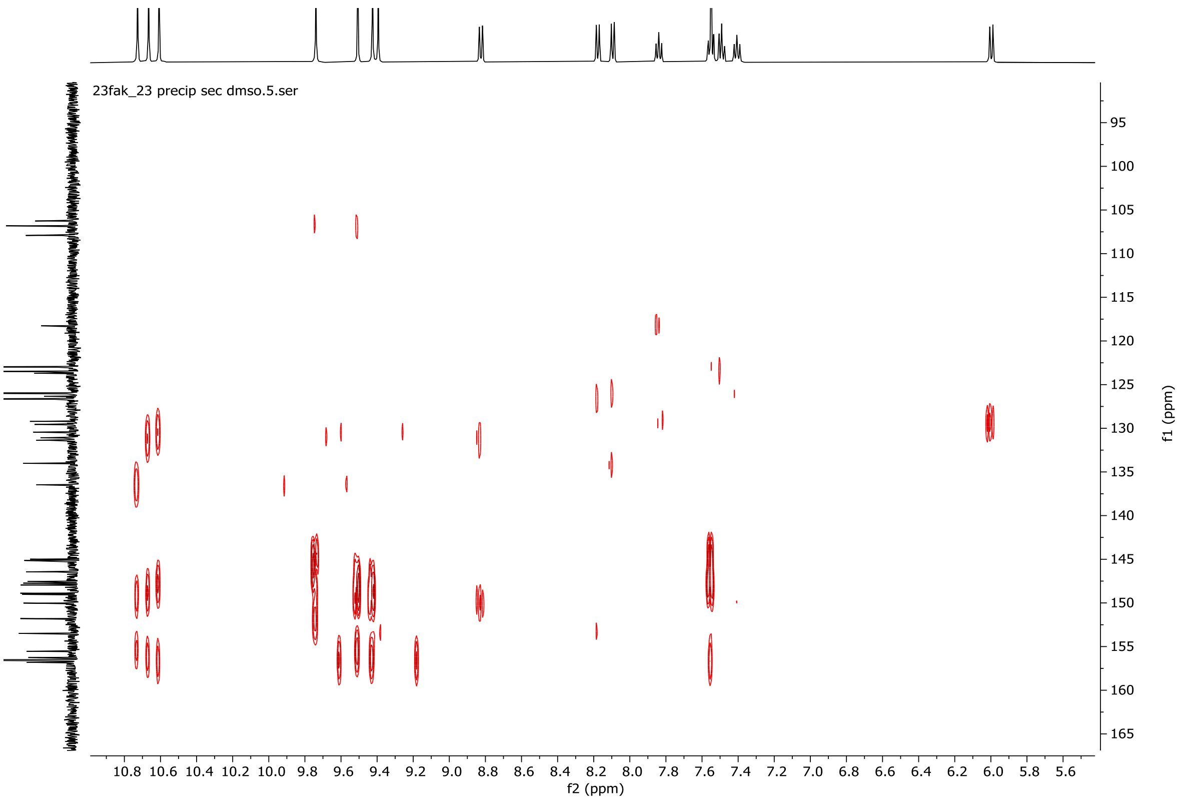

Figure S51 : Full range (top) and partial (bottom) ^{1}H-^{13}C HMBC spectra of **f-(Zn-7^{+})** in $(CD_3)_2SO$, 500 MHz, 298 K.

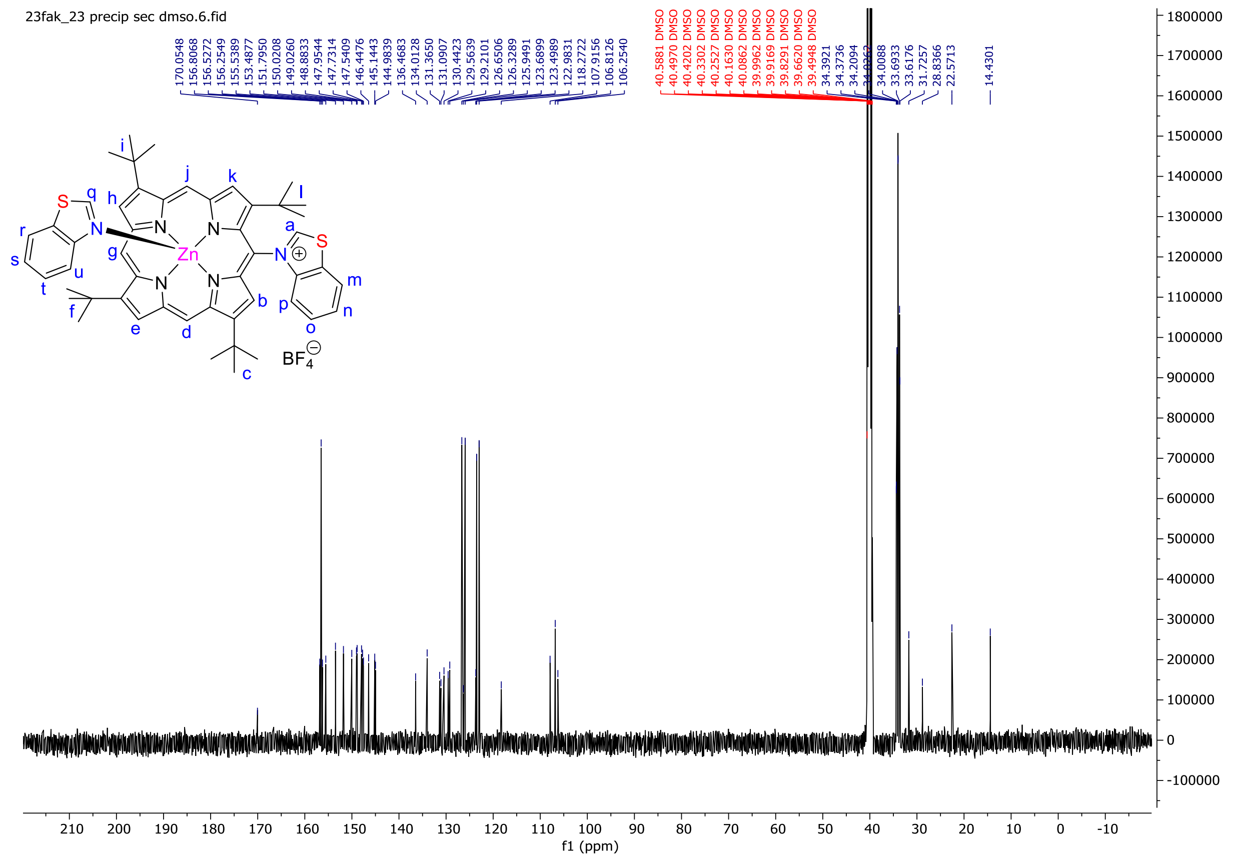

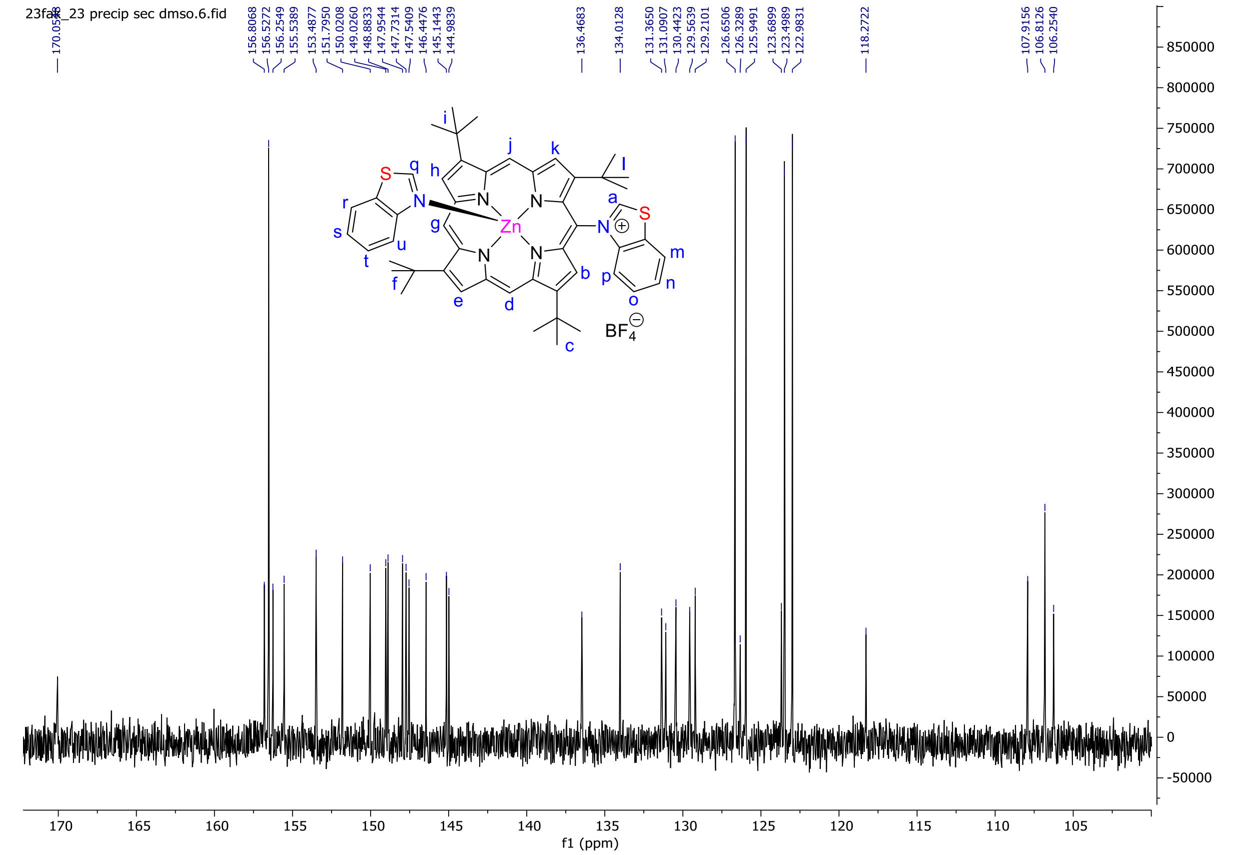

Figure S52. Full range (top) and partial (bottom) ^{13}C spectra of **f-(Zn-7$^+$)** in $(CD_3)_2SO$, 500 MHz, 298 K.

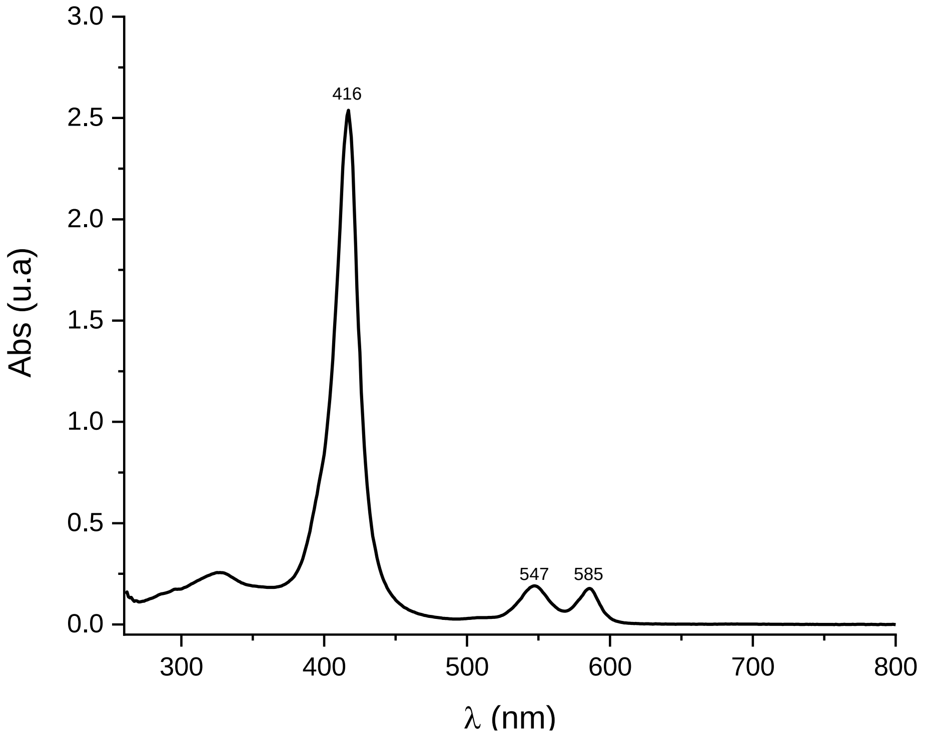

Figure S53. UV-Vis. absorption spectrum of **f-(Zn-7$^+$)** in DMSO, room temperature.

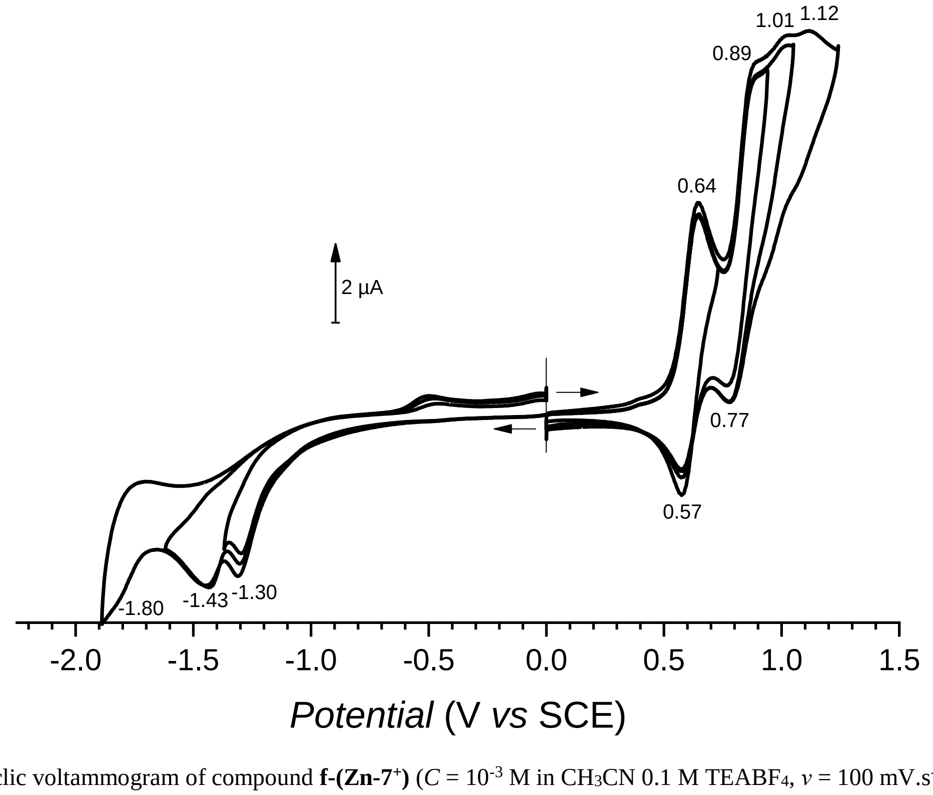

Figure S54. Cyclic voltammogram of compound **f-(Zn-7$^+$)** ($C = 10^{-3}$ M in CH_3CN 0.1 M $TEABF_4$, $v = 100$ mV.s^{-1}, WE: Pt, Ø = 1.6 mm, CE: Pt, RE: SCE, IUPAC convention).

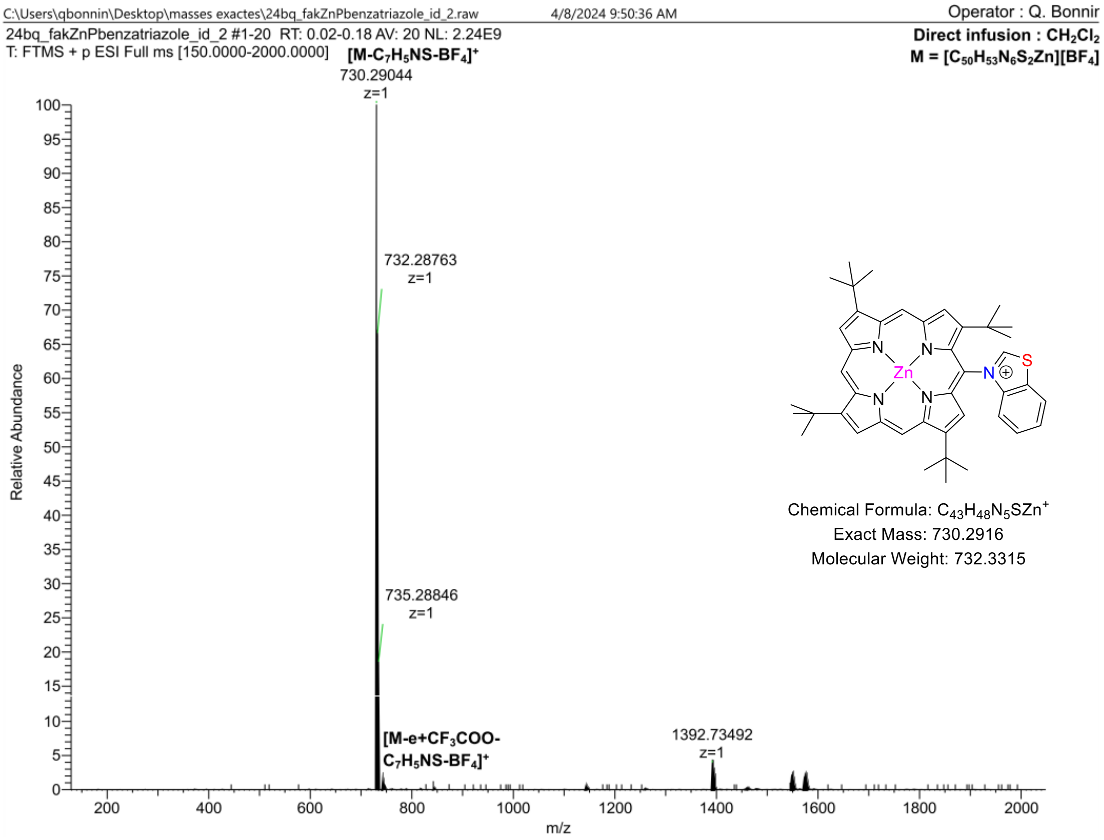

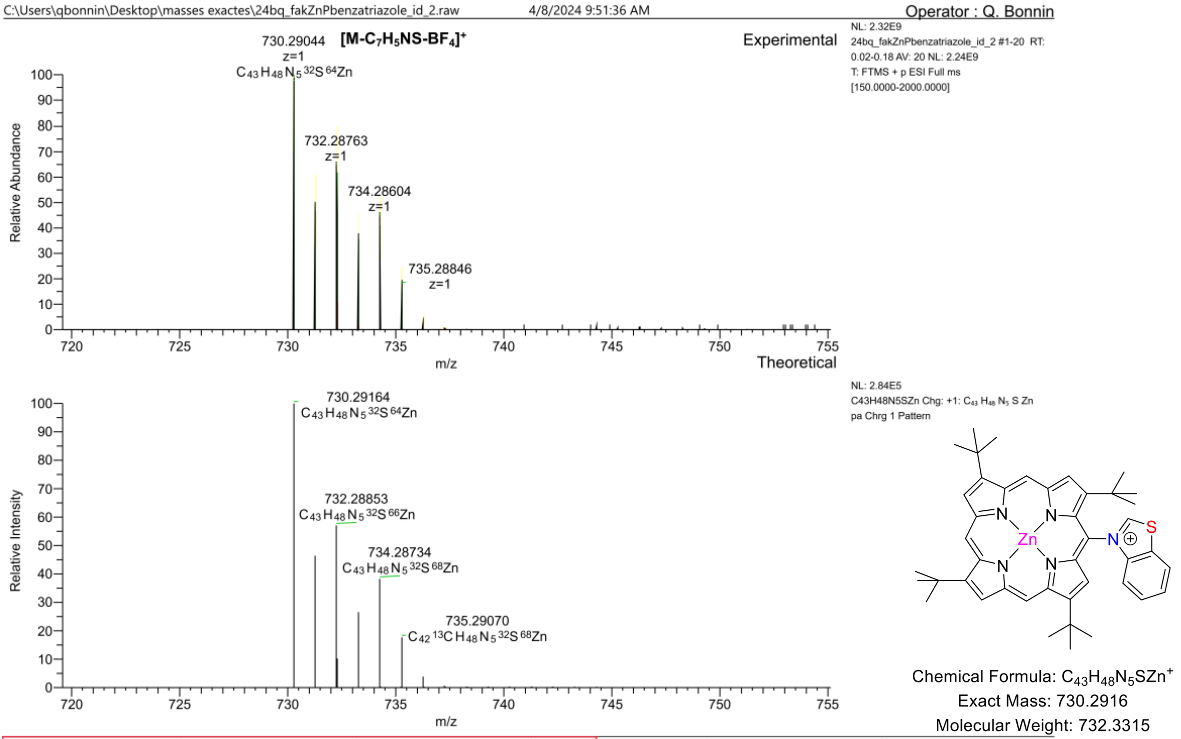

Display Formula	Peak Mass	Theo. mass	Delta [ppm]	RDB	MSMS Matched Fragments
$C_{43}H_{48}N_5{}^{32}S^{64}Zn$	730.29044	730.29164	-1.64	22.50	(Collection)

Figure S55. High resolution ESI mass spectrum of **f-(Zn-7⁺)** and simulation of its isotopic pattern.

Synthesis and characterization of Zn-1

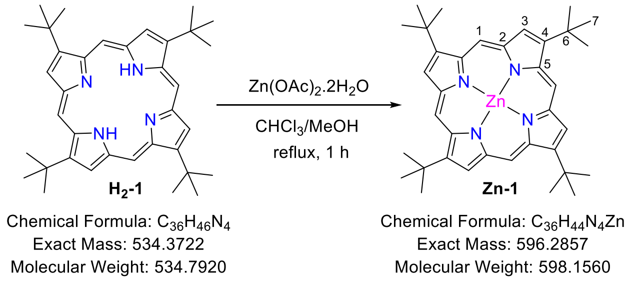

2,7,12,17-tetra-*tert*-butylporphyrin **(H_2-1)**[1–3] (3.001 g, 5.61 mmol) and Zn(AcO)$_2$·2H_2O (1.290 g, 5.877 mmol, 1.05 eq.) were dissolved in a mixture containing 80 mL of $CHCl_3$ and 20 mL of MeOH. This solution was stirred and heated under reflux during 1 h (oil bath temperature = 65 °C). The solvent was evaporated and the resulting solid was dissolved in 50 mL CH_2Cl_2 and washed 3 times with 25 mL of water. Then the organic phase was evaporated and the resulting solid was dried overnight under vacuum at 110 °C providing **Zn-1** in 75% yield (2.531 g, 4.23 mmol).

^{1}H NMR ^{1}H NMR (500 MHz, $CDCl_3$, 298 K): δ (ppm) 10.59 (s, 4H, H1), 9.32 (s, 4H, H3), 2.36 (s, 36H, H7).

^{13}C NMR (126 MHz, $CDCl_3$, 298 K): δ (ppm) 34.0 (C7), 34.4 (C6), 104.2 (C1), 129.0 (C3), 146.6 (C4), 148.3 (C5 or C2), 155.1 (C2 or C5).

λ_{max} (CH_2Cl_2) / nm (log ε): 400 (5.71), 529 (4.27), 564 (4.34).

HRMS(ESI+): m/z cald for $C_{36}H_{45}N_4Zn$ $[M+H]^+$: 597.29302, found 597.29200.

Characterization of Zn-1

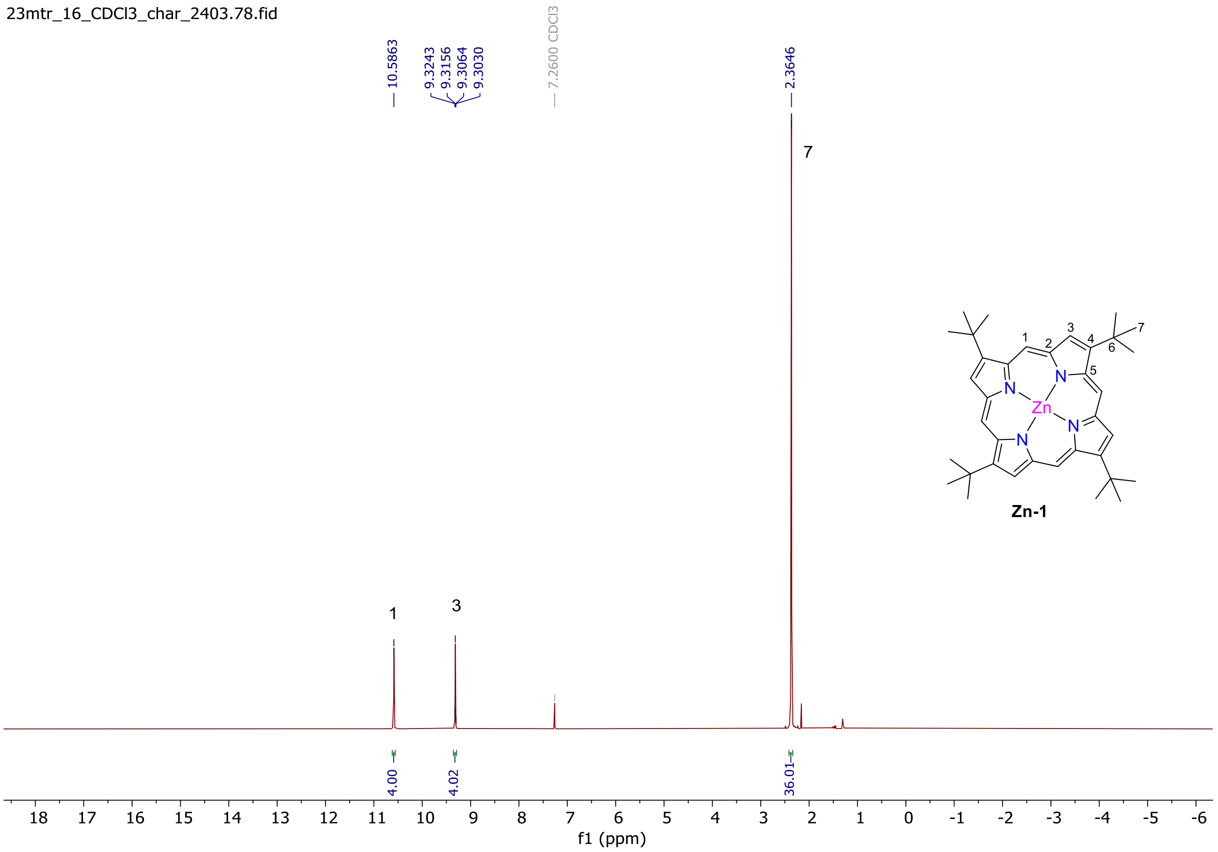

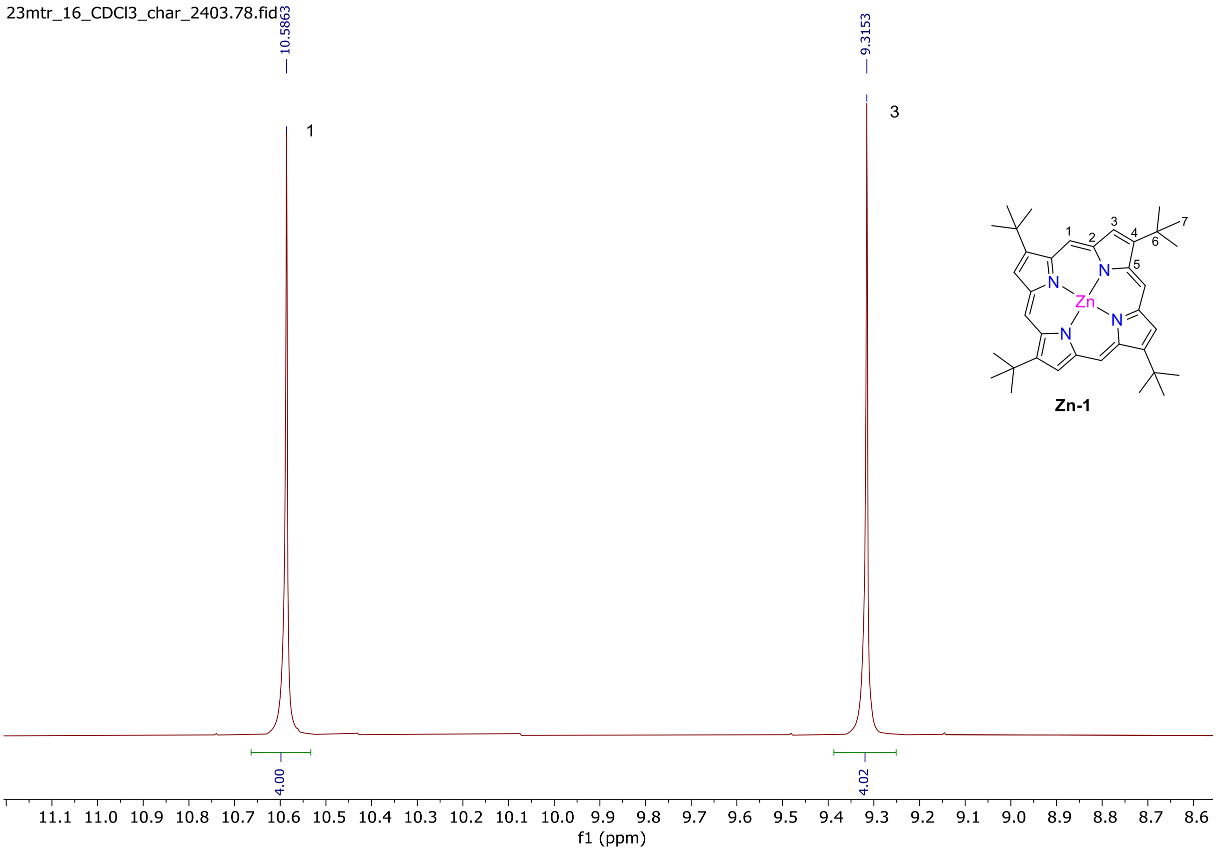

Figure S56. Full range (top) and partial (bottom) ^{1}H NMR spectra of **Zn-1** in $CDCl_3$, 500 MHz, 298 K.

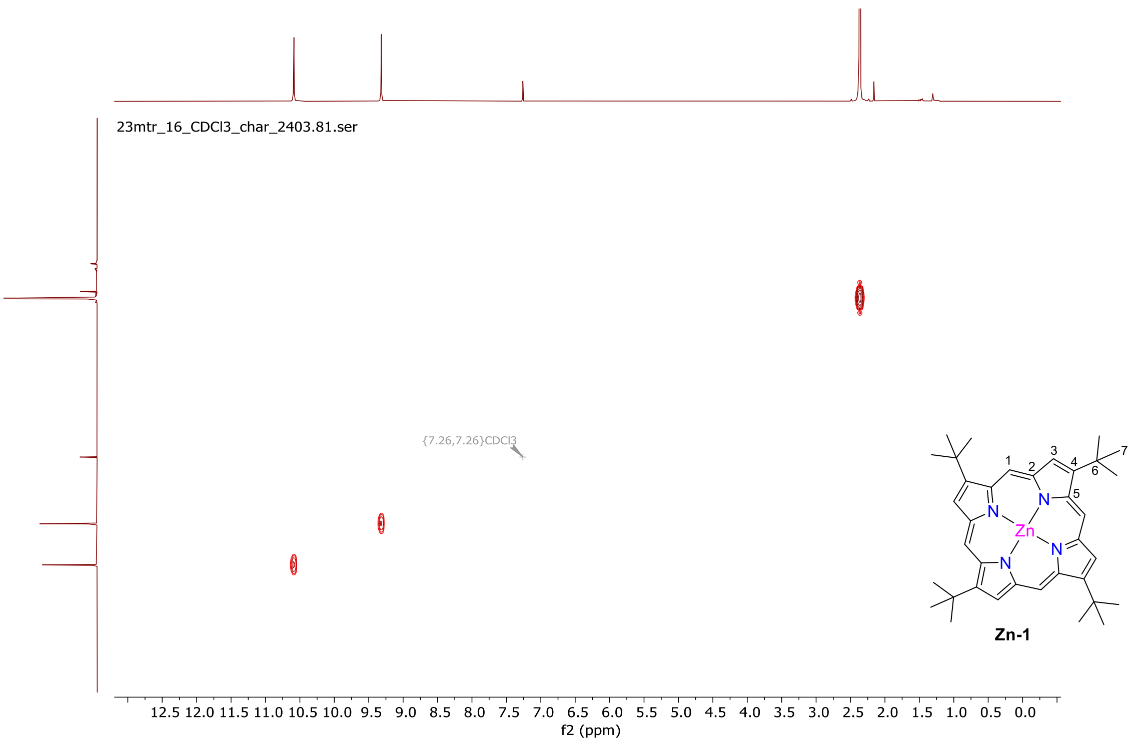

Figure S57. Full range ^{1}H-^{1}H COSY NMR spectrum of **Zn-1** in $CDCl_3$, 500 MHz, 298 K.

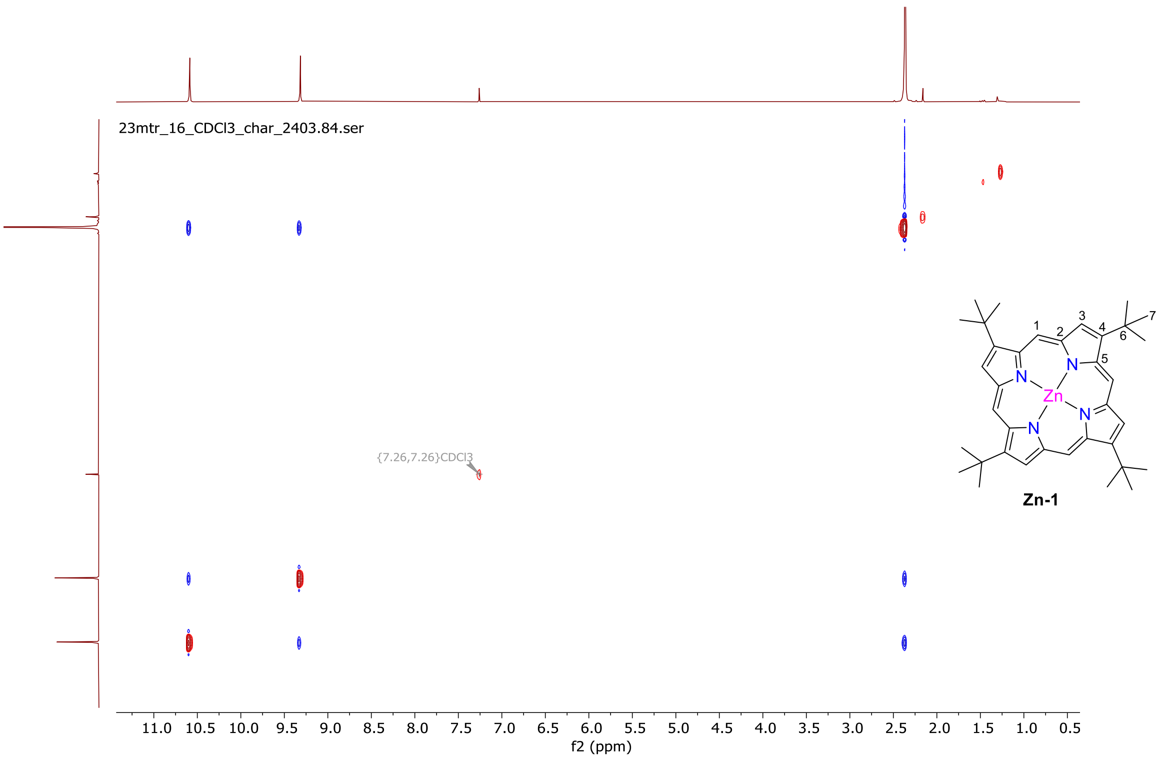

Figure S58. Full range ^{1}H-^{1}H NOESY NMR spectrum of **Zn-1** in $CDCl_3$, 500 MHz, 298 K.

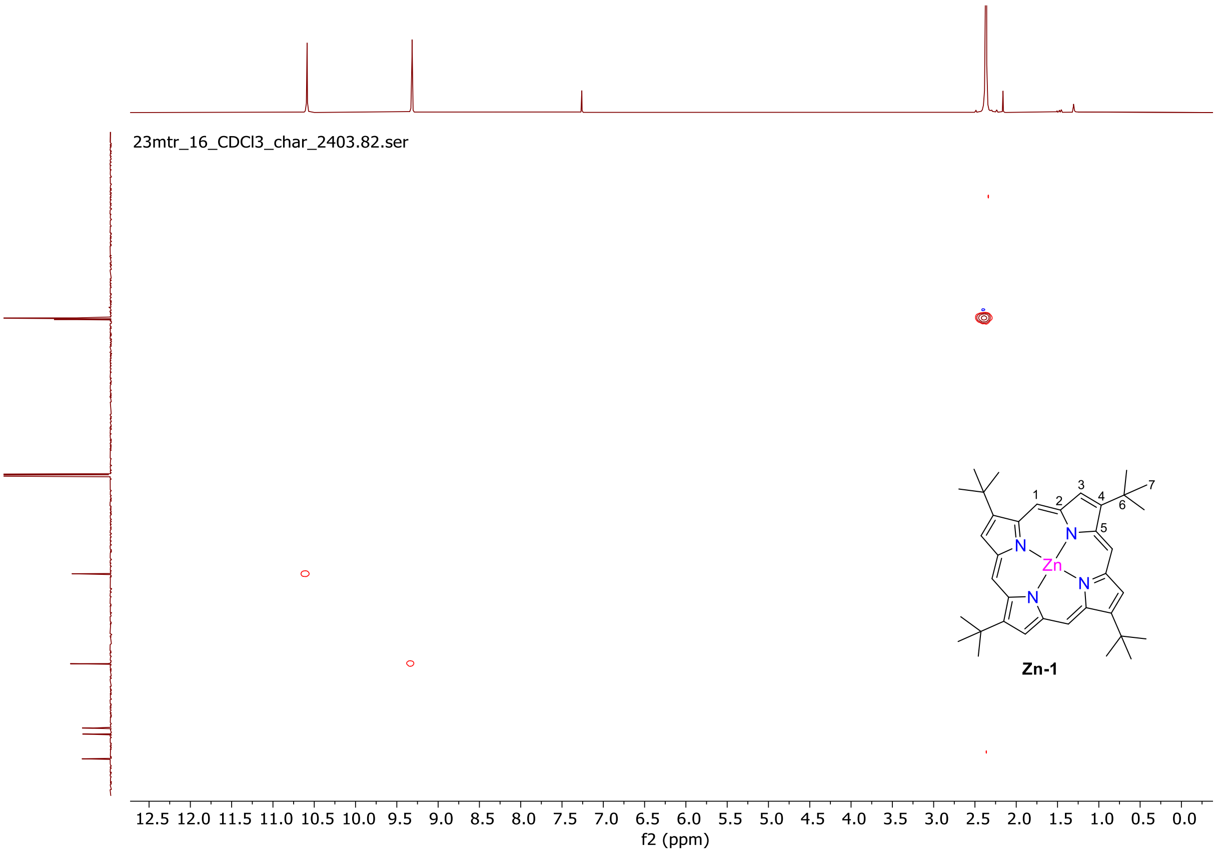

Figure S59. Full range ^{1}H-^{13}C HSQC spectra of **Zn-1** in $CDCl_3$, 500 MHz, 298 K.

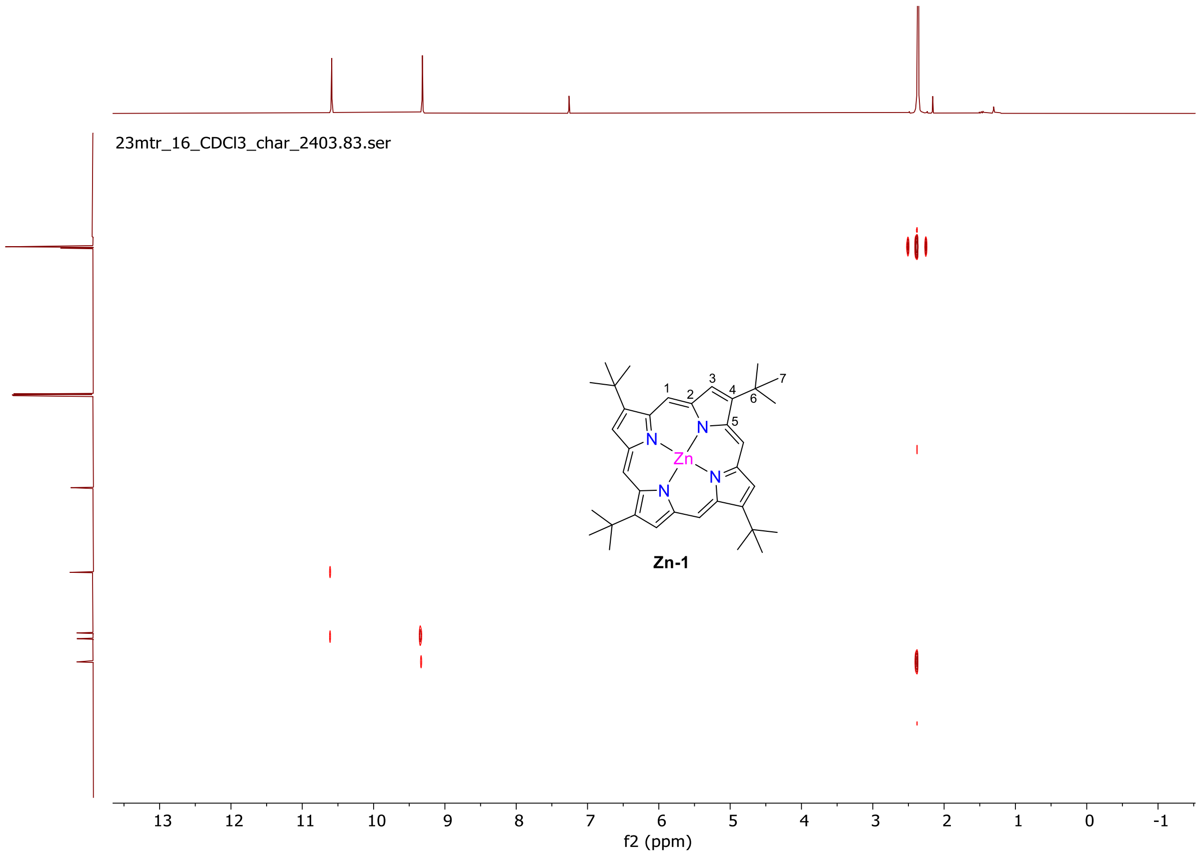

Figure S60. Full range ^{1}H-^{13}C HMBC spectra of **Zn-1** in $CDCl_3$, 500 MHz, 298 K.

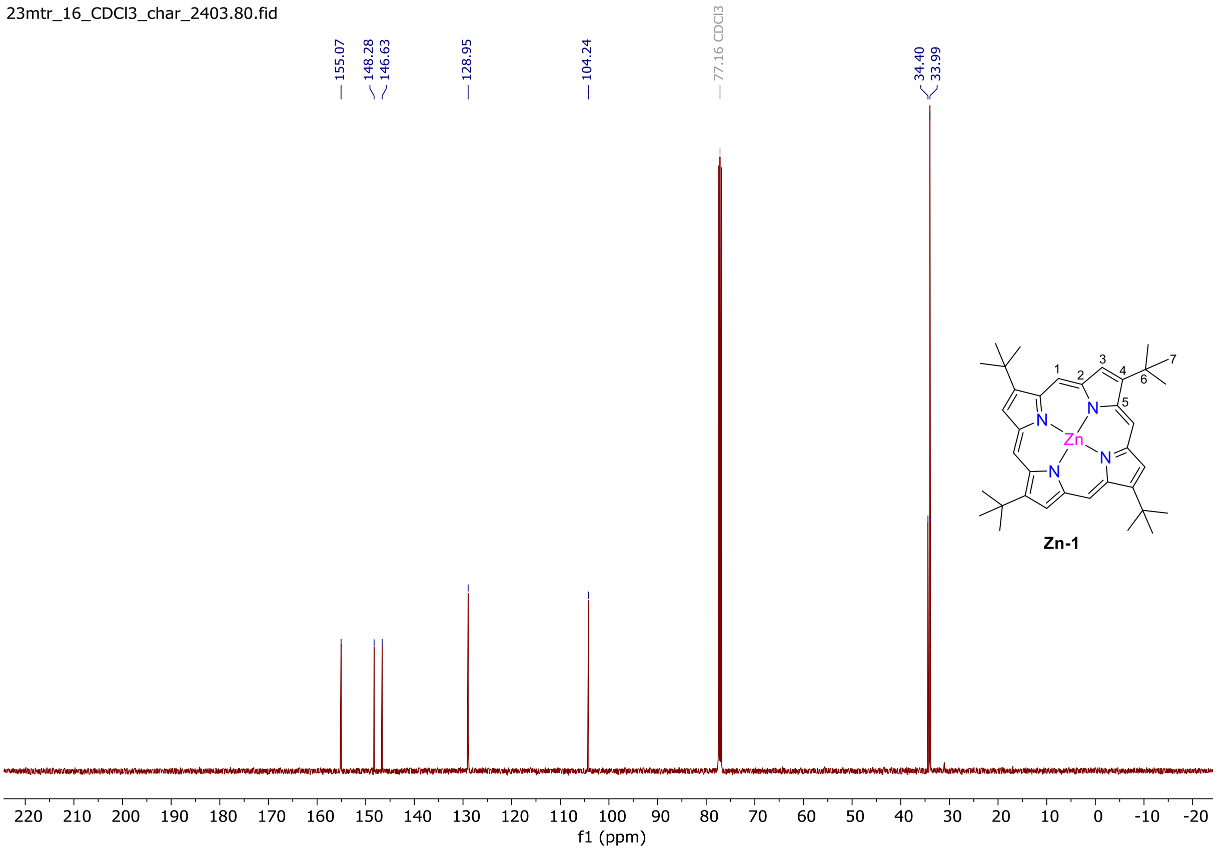

Figure S61. Full range ^{13}C spectra of **Zn-1** in $CDCl_3$, 500 MHz, 298 K.

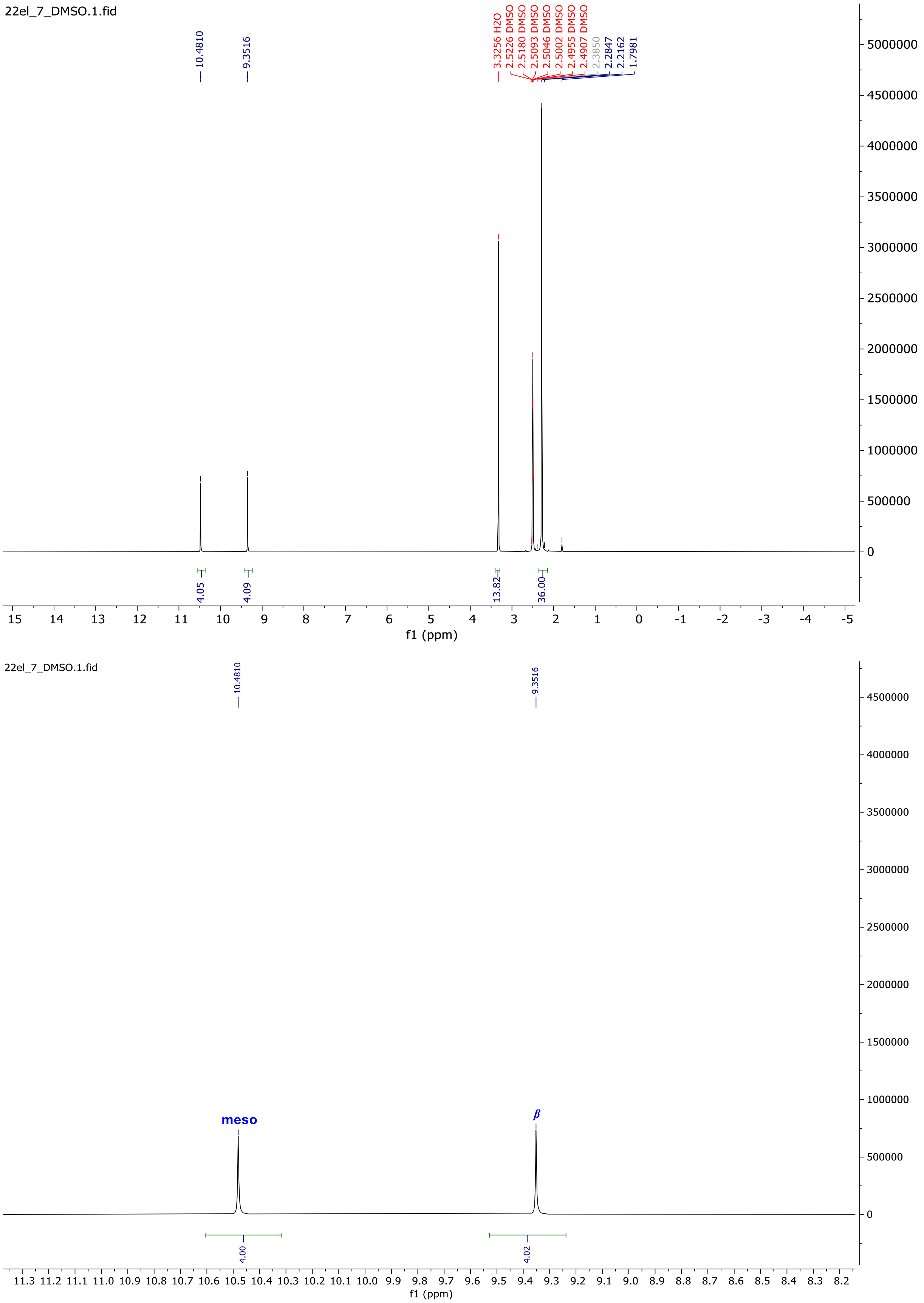

Figure S62. Full range (top) and partial (bottom) ^{1}H NMR spectra of **Zn-1** in $(CD_3)_2SO$, 500 MHz, 298 K.

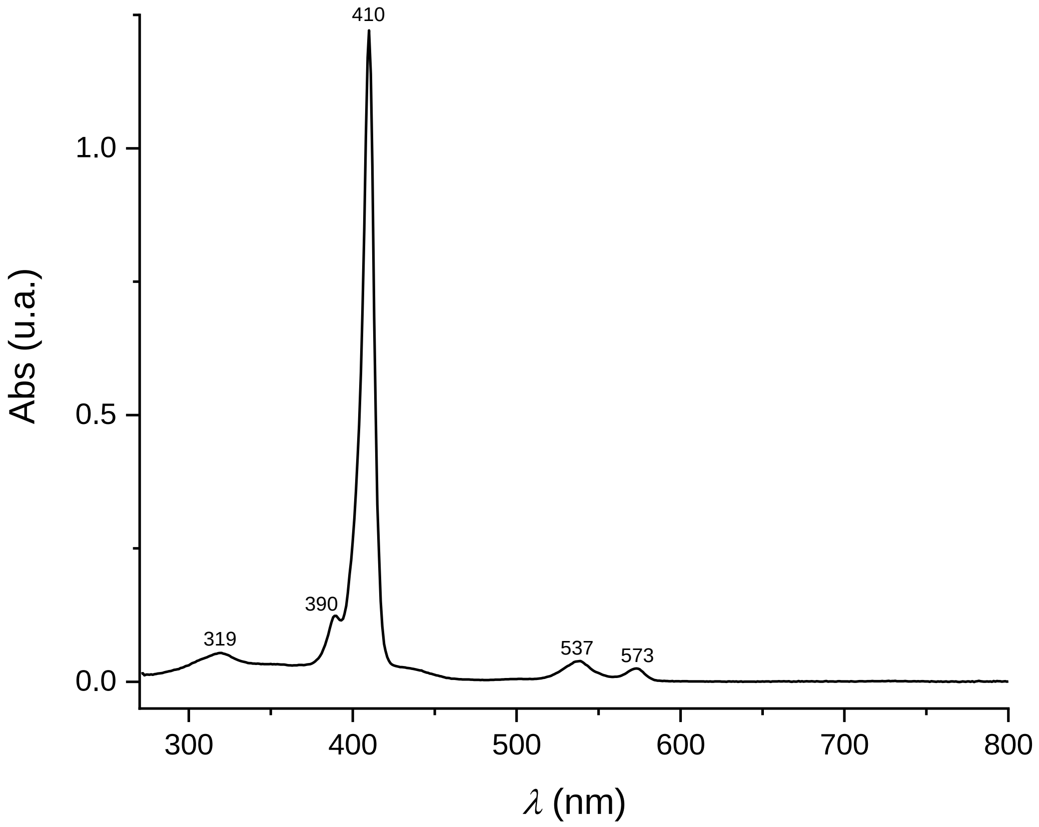

Figure S63. UV-Vis. absorption spectrum of **Zn-1** in DMSO, room temperature.

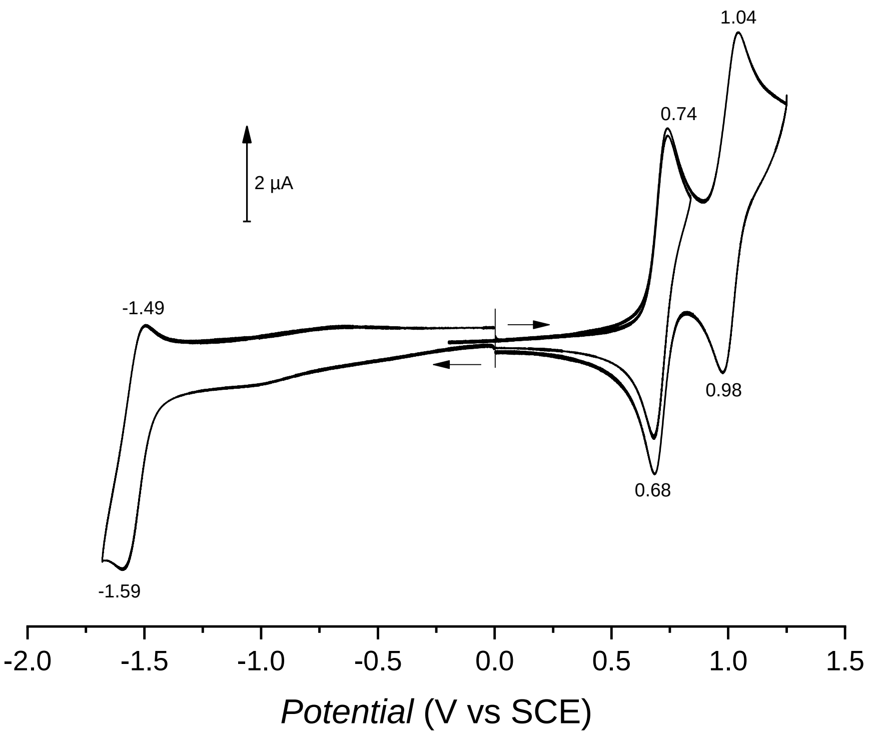

Figure S64. Cyclic voltammogram of **Zn-1** (C = 10^{-3} M in CH_3CN 0.1 M $TEABF_4$, ν = 100 mV s^{-1}, WE: Pt, Ø = 1.6 mm, CE: Pt, RE: SCE, IUPAC convention).

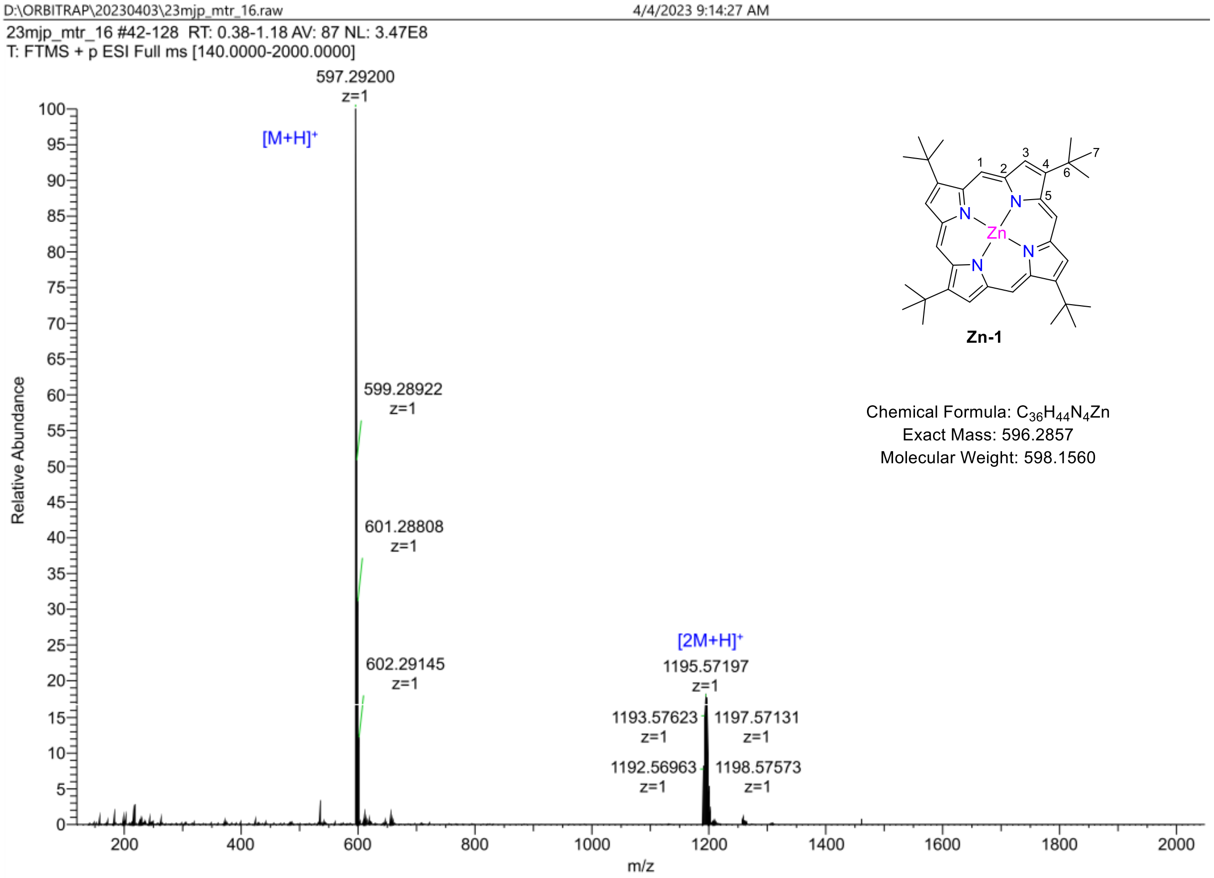

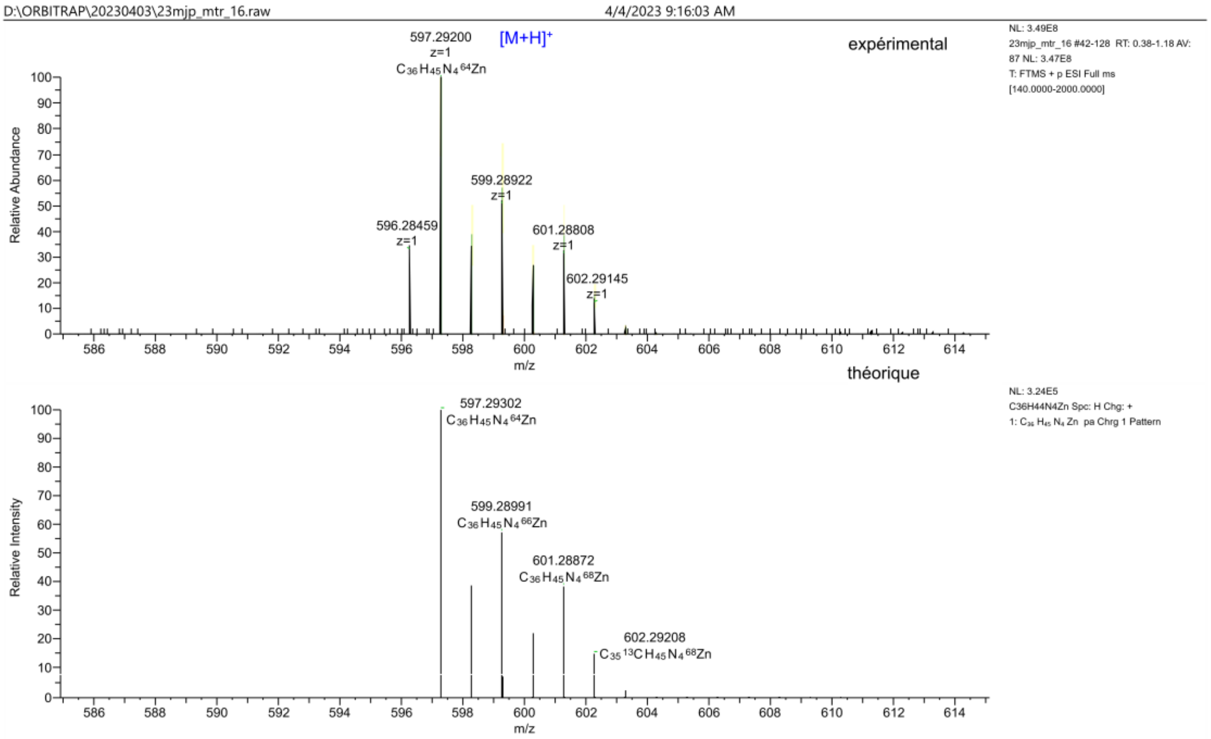

Peak Mass	Display Formula	RDB	Delta [ppm]	Theo. mass	Rank	Pattern Cov. [%]	MSMS Matched Fragm...
597.29200	$C_{36}H_{45}N_4{}^{64}Zn$	16.50	-1.71	597.29302	1	94.45	(Collection)

Figure S65. High resolution ESI mass spectrum of **Zn-1** and simulation of its isotopic pattern.

Oxidation of Zn-1 followed by UV-visible absorption spectroelectrochemistry.

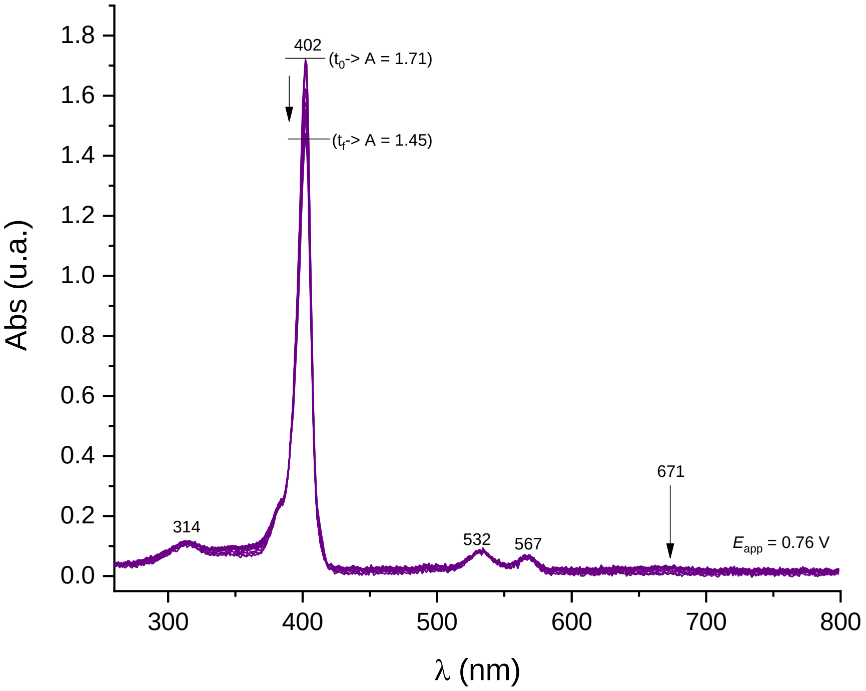

Figure S66. UV-Vis. absorption spectrum evolution during the electrochemical oxidation (E_{app} = 0.76 V/SCE, 1.0 F) of **Zn-1** (c = 3.3×10^{-5} M) in CH_3CN 0.1 M $TEABF_4$, room temperature. Divided cell, WE: Pt, CE: Pt, RE: SCE), Δt = 20 min.

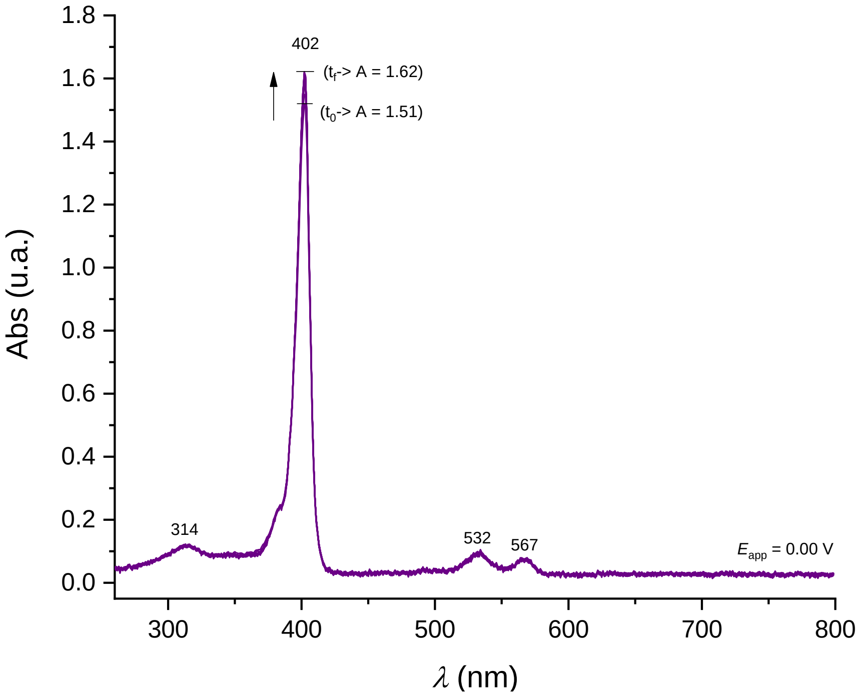

Figure S67. UV-Vis. absorption spectrum evolution during the electrochemical reduction of **Zn-1**$^{•+}$ (E_{app} = 0.00 V/SCE, 1.0 F, c = 3.3×10^{-5} M) in CH_3CN 0.1 M $TEABF_4$, room temperature. Divided cell, WE: Pt, CE: Pt, RE: SCE), Δt = 10 min.

X-ray crystallographic data

Zn-4^{+}

Olex2
Report created with ReportPlus

R_1=10.17%

Crystal Data and Experimental

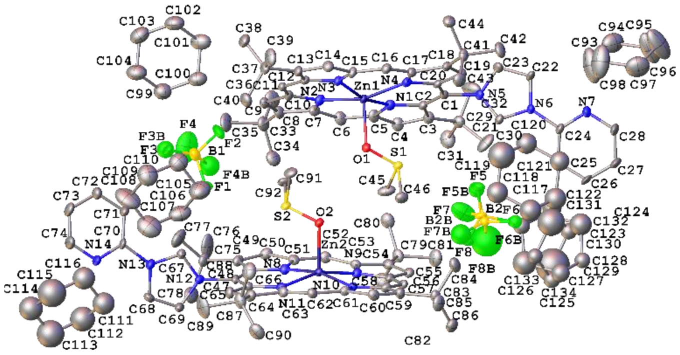

Experimental. Single clear light red prism-shaped crystals of **Zn-4^{+}** recrystallised from a mixture of dichloromethane and cyclohexane by slow evaporation. A suitable crystal with dimensions 0.30 × 0.25 × 0.20 mm^3 was selected and mounted on a mylar loop with oil on a Bruker APEX-II CCD diffractometer. The crystal was kept at a steady T = 110 K during data collection. The structure was solved with the ShelXT[6] 2018/2 solution program using dual methods and by using Olex2 1.5[7] as the graphical interface. The model was refined with ShelXL[8] 2019/3 using full matrix least squares minimisation on $\boldsymbol{F}^2$.

Crystal Data. $C_{64}H_{92}BF_4N_7OSZn$, M_r = 1159.68, monoclinic, $P2_1/c$ (No. 14), a = 18.5636(8) Å, b = 25.7945(11) Å, c = 26.9752(12) Å, β = 101.587(2)°, α = γ = 90°, V = 12653.5(10) Å^3, T = 110 K, Z = 8, Z' = 2, μ(Mo K$_{\alpha 1}$) = 0.478, 211176 reflections measured, 22272 unique (R_{int} = 0.0814) which were used in all calculations. The final wR_2 was 0.3006 (all data) and R_1 was 0.1017 (I≥2 σ(I)).

**Compound**	**Zn-4^{+}**
**CCDC**	**2363632**
Formula	$C_{64}H_{92}BF_4N_7OSZn$
$D_{calc.}$/ g cm^{-3}	1.217
μ/mm^{-1}	0.478
Formula Weight	1159.68
Colour	clear light red
Shape	prism-shaped
Size/mm^3	0.30×0.25×0.20
T/K	110
Crystal System	monoclinic
Space Group	$P2_1/c$
a/Å	18.5636(8)
b/Å	25.7945(11)
c/Å	26.9752(12)
α/°	90
β/°	101.587(2)
γ/°	90
V/Å^3	12653.5(10)
Z	8
Z'	2
Wavelength/Å	0.71073
Radiation type	Mo K$_{\alpha}$
Θ_{min}/°	1.103
Θ_{max}/°	25.000
Measured Refl's.	211176
Indep't Refl's	22272
Refl's I≥2 σ(I)	15178
R_{int}	0.0814
Parameters	1311
Restraints	0
Largest Peak	1.544
Deepest Hole	-1.713
GooF	1.108
wR_2 (all data)	0.3006
wR_2	0.2559
R_1 (all data)	0.1540
R_1	0.1017

A clear light red prism-shaped-shaped crystal with dimensions 0.30 × 0.25 × 0.20 mm^3 was mounted on a

mylar loop with oil. Data were collected using a Bruker APEX-II CCD diffractometer equipped with an Oxford Cryosystems low-temperature device operating at T = 110 K.

Data were measured using ϕ and ω scans with Mo K_α radiation. The diffraction pattern was indexed and the total number of runs and images was based on the strategy calculation from the program APEX3[9]. The maximum resolution that was achieved was Θ = 25.000° (0.84 Å).[9]

The unit cell was refined using SAINT V8.40B [9]on 9922 reflections, 5% of the observed reflections. Data reduction, scaling and absorption corrections were performed using SAINT V8.40B[10]. The final completeness is 100.00 % out to 25.000° in Θ. SADABS-2016/[10]was used for absorption correction. wR_2(int) was 0.0783 before and 0.0661 after correction. The Ratio of minimum to maximum transmission is 0.8100. The absorption coefficient μ of this material is 0.478 mm^{-1} at this wavelength (λ = 0.71073Å) and the minimum and maximum transmissions are 0.604 and 0.745.

The structure was solved and the space group $P2_1/c$ (# 14) determined by the ShelXT 2018/2 [8]structure solution program using using dual methods and refined by full matrix least squares minimisation on $\boldsymbol{F^2}$ using version 2019/3 of ShelXL 2019/3 [8]All non-hydrogen atoms were refined anisotropically. Hydrogen atom positions were calculated geometrically and refined using the riding model. The moiety formula is 2(C46 H56 N7 O S Zn), 2(B F4), 6(C6 H12). The two tetrafluoroborane molecules are disordered over several positions. Two fluorines atoms of the first tetrafluoroborane were found disordered over two positions with occupation factors converged to 0.73 :0.27, while for the second molecule, all the molecule was modeled with occupation factors converged also to 0.73 :0.27, the minor part being isotropically refined for the two tetrafluoroborane molecules. Six cyclohexane solvent molecules are present in the asymetric unit. Three are well defined and were anisotropically refined, two were isotropically refined and the last one was found disordered over several positions and was modeled isotropically over two positions with occupation factors converged to 0.61 :0.39. All the solvent and counter-ions molecules were modeled as rigid groups.

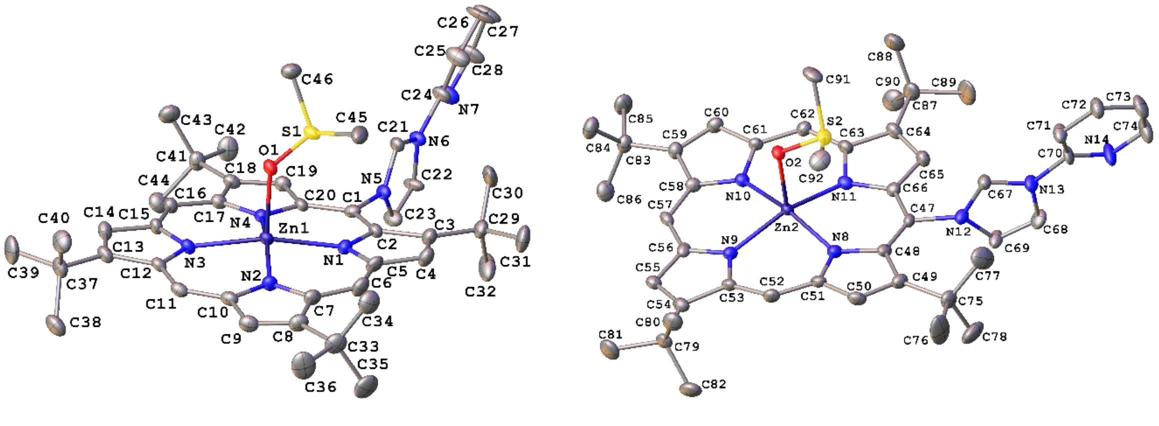

Figure : First and second molecules present in the asymetric unit.

Table 1: Fractional Atomic Coordinates (×10[4]) and Equivalent Isotropic Displacement Parameters (Å[2]×10[3]) for **Zn-4⁺**. U_{eq} is defined as 1/3 of the trace of the orthogonalised U_{ij}.

Atom	x	y	z	U_{eq}
N1	2256(3)	1941(2)	5811(2)	31.1(13)
Zn1	2910.8(4)	2603.6(3)	5761.6(3)	24.4(2)
O1	2424(2)	2802.5(18)	5018.6(17)	28.7(10)
C1	1282(4)	2321(3)	6128(3)	28.1(15)
S1	1600.6(9)	2683.0(7)	4864.4(7)	33.4(4)
N2	3754(3)	2150(2)	5626(2)	32.3(13)

Atom	x	y	z	U_{eq}
C2	1575(4)	1887(3)	5929(3)	28.0(15)
C3	1290(4)	1353(3)	5799(3)	36.6(17)
N3	3659(3)	3220(2)	5900(2)	27.6(12)
N4	2223(3)	3003(2)	6134(2)	27.5(12)
C4	1842(4)	1124(3)	5611(3)	43.6(19)
N5	551(3)	2288(2)	6251(2)	28.8(13)
C5	2437(4)	1480(3)	5626(3)	37.9(17)
C6	3085(5)	1340(3)	5483(3)	42.8(19)
N6	-613(3)	2432(2)	6202(2)	30.4(13)
N7	-1766(3)	2614(3)	6343(3)	45.9(17)
C7	3715(4)	1638(3)	5489(3)	34.7(17)
C8	4404(4)	1472(3)	5350(3)	40.4(18)
C9	4832(4)	1888(3)	5413(3)	38.3(18)
C10	4443(4)	2321(3)	5574(3)	32.7(16)
C11	4704(4)	2820(3)	5646(3)	30.4(15)
C12	4342(4)	3251(3)	5790(3)	28.5(15)
C13	4617(4)	3790(3)	5860(3)	35.1(17)
C14	4074(4)	4058(3)	6018(3)	37.0(17)
C15	3491(4)	3702(3)	6040(3)	29.7(15)
C16	2843(4)	3836(3)	6205(3)	31.2(16)
C17	2263(4)	3515(3)	6263(3)	29.3(15)
C18	1613(4)	3671(3)	6464(3)	31.2(16)
C19	1195(4)	3237(3)	6435(3)	31.3(15)
C20	1561(3)	2825(3)	6231(2)	26.5(14)
C21	-66(4)	2422(3)	5939(3)	29.9(15)
C22	-310(4)	2326(3)	6710(3)	40.0(19)
C23	420(4)	2229(3)	6735(3)	39.3(18)
C24	-1382(4)	2509(3)	6004(3)	37.8(18)
C25	-1658(5)	2446(4)	5491(3)	52(2)
C26	-2396(4)	2524(5)	5322(3)	66(3)
C27	-2828(5)	2647(4)	5667(3)	60(3)
C28	-2491(4)	2681(4)	6168(3)	57(3)
C29	607(4)	1042(3)	5866(3)	39.8(18)
C30	-112(5)	1281(4)	5621(5)	76(3)
C31	625(6)	507(3)	5606(4)	66(3)
C32	647(7)	940(4)	6418(4)	78(4)
C33	4570(5)	925(3)	5167(4)	52(2)
C34	4037(5)	774(3)	4688(4)	58(2)
C35	5350(5)	948(4)	5056(5)	72(3)
C36	4582(6)	536(3)	5592(4)	68(3)
C37	5342(4)	4003(3)	5782(3)	42.6(19)
C38	5993(4)	3736(4)	6139(4)	55(2)
C39	5385(5)	4589(4)	5914(4)	66(3)
C40	5429(5)	3942(4)	5235(3)	55(2)
C41	1428(4)	4198(3)	6649(3)	37.7(18)
C42	725(5)	4154(3)	6861(4)	55(2)
C43	1321(5)	4599(3)	6231(3)	51(2)
C44	2048(5)	4374(3)	7095(3)	48(2)
C45	1548(5)	2114(3)	4489(3)	56(2)
C46	1267(5)	3112(3)	4363(3)	45(2)
O2	2687(2)	2752.7(18)	3725.3(17)	28.3(10)
Zn2	2214.0(4)	2509.7(3)	2996.6(3)	22.63(19)
S2	3522.2(9)	2716.2(8)	3866.2(7)	35.4(4)
N8	2874(3)	1860(2)	2970(2)	31.4(13)
N9	1377(3)	2048(2)	3144(2)	28.5(12)
N10	1444(3)	3114(2)	2843(2)	27.4(12)
N11	2875(3)	2911(2)	2609(2)	28.1(12)
N12	4552(3)	2207(2)	2492(2)	31.5(13)
N13	5708(3)	2370(3)	2535(2)	37.2(15)
N14	6867(3)	2576(3)	2393(2)	49.4(19)

Atom	x	y	z	U_{eq}
C47	3823(3)	2239(3)	2618(2)	26.1(14)
C48	3557(4)	1801(3)	2841(3)	31.5(16)
C49	3853(4)	1282(3)	2980(3)	36.7(17)
C50	3324(4)	1057(3)	3199(3)	37.0(17)
C51	2736(4)	1399(3)	3182(3)	32.2(16)
C52	2101(4)	1266(3)	3339(3)	33.1(16)
C53	1439(4)	1549(2)	3314(3)	26.6(14)
C54	759(4)	1369(3)	3442(3)	35.5(17)
C55	301(4)	1788(3)	3356(3)	32.8(16)
C56	685(4)	2208(3)	3180(3)	30.7(15)
C57	399(4)	2706(3)	3091(3)	29.6(15)
C58	751(4)	3137(3)	2946(3)	29.2(15)
C59	467(4)	3668(3)	2873(3)	35.1(17)
C60	1018(4)	3947(3)	2734(3)	36.0(17)
C61	1612(4)	3597(2)	2718(3)	25.7(14)
C62	2266(4)	3741(3)	2559(3)	32.1(16)
C63	2846(4)	3426(3)	2499(3)	31.4(16)
C64	3496(4)	3592(3)	2318(3)	36.9(17)
C65	3908(4)	3156(3)	2325(3)	36.2(17)
C66	3546(4)	2739(3)	2518(3)	29.2(15)
C67	5176(4)	2356(3)	2803(3)	31.2(15)
C68	5416(4)	2233(3)	2040(3)	41.6(19)
C69	4700(4)	2143(3)	2008(3)	40.8(19)
C70	6478(4)	2464(3)	2742(3)	38.0(18)
C71	6741(4)	2430(4)	3245(3)	46(2)
C72	7475(4)	2516(4)	3413(3)	49(2)
C73	7901(4)	2626(4)	3064(4)	57(2)
C74	7580(5)	2651(4)	2561(4)	59(3)
C75	4529(4)	961(3)	2896(3)	45(2)
C76	4536(6)	444(4)	3155(6)	97(5)
C77	5270(5)	1217(4)	3117(5)	77(3)
C78	4449(6)	844(5)	2335(4)	80(4)
C79	593(4)	840(3)	3647(3)	39.1(18)
C80	1117(5)	721(3)	4149(4)	53(2)
C81	-195(5)	840(3)	3741(4)	59(3)
C82	644(5)	420(3)	3253(4)	55(2)
C83	-268(4)	3867(3)	2933(3)	37.9(18)
C84	-378(5)	3808(3)	3478(3)	53(2)
C85	-337(5)	4448(3)	2793(4)	55(2)
C86	-898(4)	3585(3)	2569(3)	50(2)
C87	3705(5)	4126(3)	2163(4)	49(2)
C88	3831(5)	4489(4)	2614(4)	65(3)
C89	4399(6)	4086(4)	1940(5)	83(4)
C90	3101(6)	4354(4)	1738(4)	62(3)
C91	3776(5)	3199(3)	4346(3)	47(2)
C92	3711(5)	2173(3)	4264(3)	50(2)
F3	6452(3)	2782(4)	4293(2)	57(2)
F4	6023(9)	2019(5)	4458(8)	172(7)
B1	5829(4)	2477(5)	4342.3(19)	69(4)
F1	5319(3)	2575(4)	3893.4(15)	132(4)
F2	5574(3)	2694(3)	4752.3(15)	75.1(18)
F3B	6467(16)	2359(13)	4326(11)	89(8)
F4B	5370(20)	1922(14)	4263(14)	122(11)
C123	2866(9)	5636(10)	225(7)	142(11)
C124	2058(9)	5478(7)	188(6)	104(8)
C125	1652(6)	5859(7)	475(6)	81(5)
C126	2053(9)	5924(8)	1028(5)	166(14)
C127	2862(9)	6082(8)	1065(6)	99(7)
C128	3228(10)	5702(7)	770(7)	72(5)
C114	7907(6)	4096(6)	1320(6)	168(8)

Atom	x	y	z	U_{eq}
C111	6470(6)	4112(5)	1649(5)	146(7)
C112	6712(8)	3638(5)	1377(6)	181(9)
C113	7241(9)	3790(6)	1029(5)	222(12)
C115	7664(7)	4570(5)	1592(6)	238(13)
C116	7135(8)	4416(5)	1939(4)	147(7)
C129	2706(18)	5292(8)	917(9)	350(60)
C130	2715(13)	5341(9)	350(9)	77(8)
C131	2121(14)	5718(11)	82(6)	105(12)
C132	2185(17)	6251(8)	344(8)	220(30)
C133	2175(12)	6202(8)	911(8)	68(7)
C134	2770(13)	5825(10)	1178(6)	79(9)
C120	718(6)	6240(5)	4432(3)	127(6)
C117	1382(6)	5846(5)	5458(3)	137(6)
C118	548(6)	5937(5)	5299(4)	132(6)
C119	284(5)	5884(5)	4724(5)	212(11)
C121	1551(6)	6148(4)	4592(4)	111(5)
C122	1816(5)	6203(5)	5168(4)	138(6)
C107	6233(7)	3921(4)	2985(3)	138(7)
C105	5550(4)	4102(4)	3696(4)	125(6)
C106	5704(5)	3703(3)	3305(4)	102(5)
C108	6949(5)	4121(4)	3319(4)	143(7)
C109	6796(5)	4519(3)	3709(4)	117(5)
C110	6266(5)	4302(4)	4029(3)	96(4)
C100	6751(3)	5446(3)	5265(3)	74(3)
C99	7336(4)	5258(3)	4973(2)	85(4)
C101	7030(4)	5408(3)	5840(2)	89(4)
C102	7764(4)	5696(3)	6010(2)	88(4)
C103	8347(3)	5507(4)	5719(3)	94(4)
C104	8069(4)	5546(3)	5144(3)	96(4)
C93	8681(6)	3877(4)	6963(5)	188(11)
C94	8437(6)	3588(3)	7401(5)	122(6)
C95	7648(7)	3735(4)	7443(5)	165(9)
C96	7560(7)	4326(4)	7487(5)	183(11)
C97	7803(7)	4613(3)	7050(5)	130(6)
C98	8593(7)	4466(4)	7008(6)	177(10)
B2	9416(2)	2355.6(18)	4426.3(13)	42(3)
F5	9766(3)	2667(2)	4829.7(15)	49.7(17)
F8	9466(3)	2593(2)	3967.0(14)	41.9(16)
F7	9757(5)	1868.6(19)	4456(3)	121(4)
F6	8673(3)	2294(4)	4451(2)	104(4)
B2B	9287(8)	2460(6)	4340(6)	26(8)
F5B	9744(10)	2339(9)	4806(8)	73(7)
F6B	8690(8)	2757(7)	4425(7)	51(5)
F8B	9686(13)	2743(12)	4043(9)	122(15)
F7B	9026(16)	2000(9)	4089(12)	290(30)

Table 2: Anisotropic Displacement Parameters (×10^4) for **Zn-4⁺**. The anisotropic displacement factor exponent takes the form: $-2\pi^2[h^2a^{*2} \times U_{11} + ... + 2hka^* \times b^* \times U_{12}]$

Atom	U_{11}	U_{22}	U_{33}	U_{23}	U_{13}	U_{12}
N1	32(3)	25(3)	35(3)	3(2)	5(3)	0(2)
Zn1	17.9(4)	29.0(4)	26.0(4)	-1.2(3)	3.5(3)	1.3(3)
O1	23(2)	34(3)	29(3)	-4(2)	6(2)	-5.0(19)
C1	25(3)	35(4)	24(4)	7(3)	6(3)	5(3)
S1	24.8(9)	46.7(11)	26.6(9)	1.5(8)	0.6(7)	-1.7(8)
N2	27(3)	28(3)	40(4)	-1(3)	4(3)	4(2)
C2	21(3)	33(4)	28(4)	0(3)	2(3)	-4(3)
C3	35(4)	29(4)	46(5)	6(3)	9(3)	-1(3)

Atom	U_{11}	U_{22}	U_{33}	U_{23}	U_{13}	U_{12}
N3	21(3)	30(3)	31(3)	-5(2)	4(2)	0(2)
N4	23(3)	27(3)	31(3)	3(2)	2(2)	0(2)
C4	48(5)	26(4)	60(5)	6(4)	16(4)	1(3)
N5	22(3)	36(3)	30(3)	6(2)	7(2)	-5(2)
C5	41(4)	23(3)	51(5)	6(3)	13(4)	11(3)
C6	52(5)	22(4)	58(5)	2(3)	19(4)	7(3)
N6	19(3)	45(3)	28(3)	5(3)	7(2)	0(2)
N7	27(3)	74(5)	39(4)	-7(3)	12(3)	7(3)
C7	37(4)	28(4)	38(4)	-4(3)	6(3)	9(3)
C8	41(4)	37(4)	45(5)	-1(3)	14(4)	12(3)
C9	32(4)	46(5)	38(4)	-2(3)	11(3)	10(3)
C10	23(4)	44(4)	31(4)	4(3)	6(3)	4(3)
C11	22(3)	45(4)	25(4)	-2(3)	5(3)	2(3)
C12	22(3)	36(4)	26(4)	-4(3)	0(3)	-2(3)
C13	23(4)	48(4)	34(4)	-5(3)	4(3)	-6(3)
C14	36(4)	35(4)	40(4)	-8(3)	7(3)	-9(3)
C15	21(3)	28(4)	39(4)	-4(3)	5(3)	-4(3)
C16	27(4)	30(4)	35(4)	-4(3)	3(3)	-3(3)
C17	27(4)	32(4)	28(4)	-5(3)	5(3)	2(3)
C18	24(4)	39(4)	29(4)	-4(3)	2(3)	6(3)
C19	26(4)	35(4)	34(4)	-1(3)	8(3)	4(3)
C20	19(3)	33(4)	24(3)	2(3)	-1(3)	-4(3)
C21	20(3)	35(4)	33(4)	3(3)	3(3)	-3(3)
C22	18(3)	68(5)	32(4)	17(4)	1(3)	5(3)
C23	32(4)	59(5)	29(4)	11(3)	10(3)	-4(4)
C24	17(3)	59(5)	36(4)	3(4)	2(3)	-1(3)
C25	34(4)	91(7)	34(5)	-2(4)	13(4)	1(4)
C26	20(4)	131(10)	39(5)	-4(5)	-14(4)	13(5)
C27	23(4)	111(8)	41(5)	-13(5)	-9(4)	13(5)
C28	20(4)	104(8)	46(5)	-9(5)	5(4)	14(4)
C29	43(5)	34(4)	43(5)	7(3)	11(4)	-10(3)
C30	41(5)	50(6)	122(10)	6(6)	-18(6)	-20(4)
C31	74(7)	34(5)	97(8)	-4(5)	31(6)	-21(4)
C32	101(9)	81(7)	51(6)	6(5)	15(6)	-54(7)
C33	51(5)	42(5)	66(6)	-3(4)	19(5)	19(4)
C34	70(6)	41(5)	67(6)	-14(4)	22(5)	7(4)
C35	59(6)	57(6)	109(9)	-26(6)	36(6)	19(5)
C36	79(7)	34(5)	94(8)	-1(5)	26(6)	26(5)
C37	30(4)	49(5)	50(5)	-5(4)	11(4)	-13(3)
C38	30(4)	77(6)	57(6)	-9(5)	5(4)	-10(4)
C39	49(6)	58(6)	99(8)	-14(5)	35(5)	-28(5)
C40	51(5)	67(6)	53(6)	3(5)	25(4)	-15(4)
C41	32(4)	33(4)	51(5)	-8(3)	14(4)	2(3)
C42	53(5)	50(5)	70(6)	-17(4)	33(5)	5(4)
C43	50(5)	42(5)	60(6)	2(4)	10(4)	16(4)
C44	49(5)	50(5)	44(5)	-14(4)	9(4)	-1(4)
C45	75(6)	39(5)	44(5)	1(4)	-15(5)	-15(4)
C46	49(5)	42(5)	40(5)	3(4)	0(4)	10(4)
O2	21(2)	35(3)	27(3)	-3(2)	0.8(19)	-1.6(19)
Zn2	20.7(4)	23.7(4)	23.1(4)	0.0(3)	3.2(3)	-3.6(3)
S2	23.2(9)	51.6(11)	28.6(10)	-5.1(8)	-1.1(7)	-1.8(8)
N8	30(3)	27(3)	36(3)	-5(2)	5(3)	-1(2)
N9	27(3)	25(3)	32(3)	-4(2)	3(2)	1(2)
N10	26(3)	26(3)	28(3)	0(2)	2(2)	-1(2)
N11	30(3)	29(3)	23(3)	2(2)	0(2)	-4(2)
N12	25(3)	41(3)	27(3)	-6(3)	1(2)	6(2)
N13	21(3)	52(4)	37(4)	-6(3)	2(3)	1(3)
N14	20(3)	95(6)	34(4)	-3(4)	6(3)	-11(3)
C47	18(3)	33(4)	28(4)	-8(3)	5(3)	2(3)
C48	22(3)	31(4)	39(4)	-11(3)	0(3)	0(3)

Atom	U_{11}	U_{22}	U_{33}	U_{23}	U_{13}	U_{12}
C49	31(4)	33(4)	43(5)	-8(3)	2(3)	8(3)
C50	41(4)	22(3)	45(5)	1(3)	2(3)	-4(3)
C51	39(4)	25(3)	33(4)	-7(3)	8(3)	-7(3)
C52	41(4)	18(3)	41(4)	1(3)	9(3)	0(3)
C53	33(4)	18(3)	29(4)	0(3)	6(3)	-4(3)
C54	38(4)	35(4)	34(4)	-3(3)	7(3)	-11(3)
C55	30(4)	33(4)	34(4)	-5(3)	3(3)	-6(3)
C56	23(3)	42(4)	26(4)	-9(3)	1(3)	1(3)
C57	24(3)	38(4)	27(4)	1(3)	4(3)	-10(3)
C58	30(4)	26(3)	29(4)	3(3)	-1(3)	3(3)
C59	33(4)	36(4)	38(4)	4(3)	11(3)	4(3)
C60	43(4)	27(4)	38(4)	3(3)	10(3)	5(3)
C61	28(4)	18(3)	30(4)	1(3)	4(3)	-1(3)
C62	35(4)	31(4)	31(4)	-1(3)	8(3)	2(3)
C63	32(4)	29(4)	34(4)	0(3)	8(3)	-9(3)
C64	34(4)	36(4)	41(4)	6(3)	6(3)	-10(3)
C65	30(4)	38(4)	43(4)	-1(3)	12(3)	-5(3)
C66	32(4)	30(4)	26(4)	-1(3)	7(3)	0(3)
C67	24(4)	39(4)	31(4)	-3(3)	7(3)	-1(3)
C68	43(5)	62(5)	22(4)	-2(3)	11(3)	6(4)
C69	26(4)	66(5)	30(4)	-13(4)	3(3)	5(3)
C70	13(3)	60(5)	41(4)	-3(4)	7(3)	-4(3)
C71	24(4)	80(6)	35(4)	-13(4)	5(3)	-5(4)
C72	30(4)	75(6)	44(5)	-1(4)	13(4)	-8(4)
C73	23(4)	84(7)	62(6)	8(5)	6(4)	-12(4)
C74	32(5)	101(8)	48(5)	11(5)	15(4)	-13(5)
C75	32(4)	41(4)	62(6)	-4(4)	8(4)	13(3)
C76	64(7)	54(6)	184(14)	25(8)	54(8)	39(6)
C77	41(5)	65(7)	118(10)	-9(6)	-6(6)	21(5)
C78	69(7)	84(8)	87(8)	-38(6)	16(6)	31(6)
C79	44(5)	25(4)	52(5)	1(3)	20(4)	-12(3)
C80	65(6)	33(4)	66(6)	8(4)	23(5)	-5(4)
C81	56(6)	37(5)	89(7)	14(5)	27(5)	-15(4)
C82	74(6)	29(4)	64(6)	-7(4)	16(5)	-20(4)
C83	37(4)	33(4)	46(5)	8(3)	14(4)	8(3)
C84	51(5)	53(5)	61(6)	6(4)	26(4)	16(4)
C85	47(5)	39(5)	82(7)	5(4)	18(5)	17(4)
C86	37(5)	51(5)	59(6)	9(4)	5(4)	11(4)
C87	52(5)	30(4)	71(6)	6(4)	30(5)	-8(4)
C88	58(6)	42(5)	97(8)	-9(5)	23(6)	-20(4)
C89	89(8)	48(6)	134(11)	19(6)	77(8)	-19(5)
C90	84(7)	45(5)	64(6)	17(5)	31(6)	-4(5)
C91	42(5)	55(5)	40(5)	-7(4)	-5(4)	-20(4)
C92	55(5)	60(5)	28(4)	7(4)	-7(4)	15(4)
F3	24(3)	111(7)	34(4)	6(4)	2(3)	-12(3)
F4	154(13)	58(7)	310(20)	6(10)	66(14)	26(8)
B1	49(7)	114(11)	46(6)	0(7)	20(5)	15(7)
F1	46(3)	313(13)	35(3)	-37(5)	3(3)	-36(5)
F2	62(4)	121(5)	52(3)	-31(3)	35(3)	-23(3)
C107	191(19)	86(11)	143(16)	-36(11)	43(15)	-22(12)
C105	119(13)	125(13)	122(13)	-41(11)	-1(10)	-8(10)
C106	126(12)	75(8)	91(10)	-14(7)	-15(9)	-9(8)
C108	220(20)	102(12)	118(14)	-54(10)	62(14)	-42(13)
C109	130(13)	84(10)	139(14)	-22(9)	37(11)	-8(9)
C110	102(10)	75(8)	108(11)	-13(7)	15(9)	-3(7)
C100	90(8)	60(6)	65(7)	2(5)	-2(6)	-14(6)
C99	91(9)	81(8)	82(9)	-7(7)	11(7)	-1(7)
C101	112(10)	72(8)	77(8)	8(6)	7(7)	-34(7)
C102	103(10)	89(9)	65(8)	-4(6)	-1(7)	-17(7)
C103	84(9)	88(9)	98(10)	1(7)	-8(7)	-12(7)

Atom	U_{11}	U_{22}	U_{33}	U_{23}	U_{13}	U_{12}
C104	114(11)	91(9)	76(9)	-10(7)	4(8)	-22(8)
C93	190(20)	90(12)	340(30)	-11(16)	180(20)	-26(12)
C94	131(14)	96(11)	129(14)	-11(10)	3(11)	36(10)
C95	220(20)	96(12)	220(20)	74(13)	153(19)	61(13)
C96	280(30)	104(13)	210(20)	51(13)	170(20)	94(16)
C97	160(17)	93(11)	128(14)	33(10)	8(12)	15(11)
C98	122(15)	86(12)	340(30)	12(15)	100(18)	-17(10)
B2	38(7)	63(9)	20(6)	1(6)	-6(6)	-5(6)
F5	52(4)	72(5)	25(3)	3(3)	4(3)	-17(3)
F8	45(4)	59(4)	20(3)	8(3)	3(3)	-5(3)
F7	210(13)	64(6)	94(8)	20(5)	46(8)	32(7)
F6	47(5)	231(13)	35(4)	-6(6)	12(3)	-51(6)

Table 3: Bond Lengths in Å for **Zn-4⁺**.

Atom	Atom	Length/Å
N1	Zn1	2.117(6)
N1	C2	1.372(9)
N1	C5	1.357(9)
Zn1	O1	2.090(5)
Zn1	N2	2.046(6)
Zn1	N3	2.095(5)
Zn1	N4	2.053(6)
O1	S1	1.533(5)
C1	C2	1.398(10)
C1	N5	1.464(8)
C1	C20	1.405(9)
S1	C45	1.774(9)
S1	C46	1.760(8)
N2	C7	1.370(9)
N2	C10	1.386(9)
C2	C3	1.491(10)
C3	C4	1.366(11)
C3	C29	1.541(10)
N3	C12	1.362(8)
N3	C15	1.353(9)
N4	C17	1.364(9)
N4	C20	1.385(8)
C4	C5	1.430(11)
N5	C21	1.324(9)
N5	C23	1.383(9)
C5	C6	1.383(11)
C6	C7	1.396(11)
N6	C21	1.349(9)
N6	C22	1.401(9)
N6	C24	1.434(9)
N7	C24	1.297(10)
N7	C28	1.345(10)
C7	C8	1.466(10)
C8	C9	1.327(11)
C8	C33	1.546(11)
C9	C10	1.444(10)
C10	C11	1.376(10)
C11	C12	1.393(10)
C12	C13	1.479(10)
C13	C14	1.360(10)
C13	C37	1.508(10)
C14	C15	1.430(9)
C15	C16	1.406(10)
C16	C17	1.392(10)
C17	C18	1.475(9)
C18	C19	1.355(10)
C18	C41	1.510(10)
C19	C20	1.430(9)
C22	C23	1.368(10)
C24	C25	1.383(11)
C25	C26	1.369(11)
C26	C27	1.382(13)
C27	C28	1.373(12)
C29	C30	1.500(12)
C29	C31	1.553(12)
C29	C32	1.497(12)
C33	C34	1.513(13)
C33	C35	1.538(12)
C33	C36	1.522(13)
C37	C38	1.549(12)
C37	C39	1.552(12)
C37	C40	1.524(12)
C41	C42	1.531(11)
C41	C43	1.514(11)
C41	C44	1.555(11)
O2	Zn2	2.082(4)
O2	S2	1.524(5)
Zn2	N8	2.087(6)
Zn2	N9	2.060(6)
Zn2	N10	2.099(5)
Zn2	N11	2.047(6)
S2	C91	1.790(8)
S2	C92	1.758(8)
N8	C48	1.388(9)
N8	C51	1.364(9)
N9	C53	1.362(8)
N9	C56	1.371(9)
N10	C58	1.370(9)
N10	C61	1.345(8)
N11	C63	1.359(9)
N11	C66	1.390(9)
N12	C47	1.463(8)
N12	C67	1.343(9)
N12	C69	1.397(9)

Atom	Atom	Length/Å
N13	C67	1.335(9)
N13	C68	1.383(10)
N13	C70	1.448(9)
N14	C70	1.327(10)
N14	C74	1.323(10)
C47	C48	1.414(10)
C47	C66	1.395(9)
C48	C49	1.467(10)
C49	C50	1.373(11)
C49	C75	1.559(10)
C50	C51	1.397(10)
C51	C52	1.375(10)
C52	C53	1.419(10)
C53	C54	1.451(10)
C54	C55	1.364(10)
C54	C79	1.527(10)
C55	C56	1.430(10)
C56	C57	1.391(10)
C57	C58	1.385(9)
C58	C59	1.467(9)
C59	C60	1.364(10)
C59	C83	1.496(10)
C60	C61	1.431(10)
C61	C62	1.417(10)
C62	C63	1.383(10)
C63	C64	1.454(10)
C64	C65	1.357(11)
C64	C87	1.512(10)
C65	C66	1.420(10)
C68	C69	1.335(11)
C70	C71	1.350(11)
C71	C72	1.365(11)
C72	C73	1.376(12)
C73	C74	1.369(13)
C75	C76	1.504(13)
C75	C77	1.533(13)
C75	C78	1.523(13)
C79	C80	1.532(12)
C79	C81	1.535(11)
C79	C82	1.532(11)
C83	C84	1.531(11)
C83	C85	1.545(10)
C83	C86	1.550(12)
C87	C88	1.518(13)
C87	C89	1.531(12)
C87	C90	1.549(13)
F3	B1	1.427(15)
F4	B1	1.256(15)
B1	F1	1.4027
B1	F2	1.4043
B1	F3B	1.23(3)

Atom	Atom	Length/Å
B1	F4B	1.65(4)
C123	C124	1.5377
C123	C128	1.50(2)
C124	C125	1.5378
C125	C126	1.5372
C126	C127	1.5391
C127	C128	1.51(2)
C114	C113	1.5410
C114	C115	1.5392
C111	C112	1.5391
C111	C116	1.5374
C112	C113	1.5394
C115	C116	1.5375
C129	C130	1.5376
C129	C134	1.5377
C130	C131	1.5378
C131	C132	1.5391
C132	C133	1.5377
C133	C134	1.5382
C120	C119	1.5395
C120	C121	1.5386
C117	C118	1.5383
C117	C122	1.5368
C118	C119	1.5368
C121	C122	1.5393
C107	C106	1.5376
C107	C108	1.5359
C105	C106	1.5409
C105	C110	1.5371
C108	C109	1.5387
C109	C110	1.5362
C100	C99	1.5413
C100	C101	1.5366
C99	C104	1.5394
C101	C102	1.5383
C102	C103	1.5394
C103	C104	1.5360
C93	C94	1.5395
C93	C98	1.5364
C94	C95	1.5372
C95	C96	1.5392
C96	C97	1.5362
C97	C98	1.5397
B2	F5	1.4023
B2	F8	1.4022
B2	F7	1.4023
B2	F6	1.4018
B2B	F5B	1.4022
B2B	F6B	1.4022
B2B	F8B	1.4024
B2B	F7B	1.4038

Table 4: Bond Angles in ° for **Zn-4**$^{+}$.

Atom	Atom	Atom	Angle/°
C2	N1	Zn1	131.6(5)
C5	N1	Zn1	119.8(5)
C5	N1	C2	107.7(6)

Atom	Atom	Atom	Angle/°
O1	Zn1	N1	96.6(2)
O1	Zn1	N3	97.3(2)
N2	Zn1	N1	91.1(2)

Atom	Atom	Atom	Angle/°
N2	Zn1	O1	99.7(2)
N2	Zn1	N3	87.8(2)
N2	Zn1	N4	161.5(2)
N3	Zn1	N1	166.0(2)
N4	Zn1	N1	87.3(2)
N4	Zn1	O1	98.9(2)
N4	Zn1	N3	89.3(2)
S1	O1	Zn1	115.2(3)
C2	C1	N5	119.3(6)
C2	C1	C20	131.3(6)
C20	C1	N5	109.3(6)
O1	S1	C45	105.0(4)
O1	S1	C46	105.1(4)
C46	S1	C45	96.3(4)
C7	N2	Zn1	126.8(5)
C7	N2	C10	106.2(6)
C10	N2	Zn1	126.2(5)
N1	C2	C1	117.1(6)
N1	C2	C3	109.8(6)
C1	C2	C3	133.1(6)
C2	C3	C29	135.6(7)
C4	C3	C2	103.2(6)
C4	C3	C29	121.0(7)
C12	N3	Zn1	128.2(4)
C15	N3	Zn1	124.4(4)
C15	N3	C12	106.5(5)
C17	N4	Zn1	127.1(5)
C17	N4	C20	106.1(6)
C20	N4	Zn1	126.1(4)
C3	C4	C5	110.2(7)
C21	N5	C1	124.3(6)
C21	N5	C23	109.6(6)
C23	N5	C1	124.6(6)
N1	C5	C4	109.0(7)
N1	C5	C6	128.6(7)
C6	C5	C4	122.3(7)
C5	C6	C7	128.8(7)
C21	N6	C22	108.3(6)
C21	N6	C24	127.2(6)
C22	N6	C24	124.4(6)
C24	N7	C28	115.6(7)
N2	C7	C6	122.1(6)
N2	C7	C8	110.4(6)
C6	C7	C8	127.4(7)
C7	C8	C33	126.1(7)
C9	C8	C7	105.2(6)
C9	C8	C33	128.7(7)
C8	C9	C10	109.9(7)
N2	C10	C9	108.2(6)
C11	C10	N2	126.1(6)
C11	C10	C9	125.7(7)
C10	C11	C12	127.7(6)
N3	C12	C11	122.4(6)
N3	C12	C13	109.9(6)
C11	C12	C13	127.7(6)
C12	C13	C37	128.3(7)
C14	C13	C12	105.1(6)
C14	C13	C37	126.6(7)
C13	C14	C15	107.5(6)
N3	C15	C14	111.1(6)

Atom	Atom	Atom	Angle/°
N3	C15	C16	125.0(6)
C16	C15	C14	123.9(6)
C17	C16	C15	128.3(6)
N4	C17	C16	123.4(6)
N4	C17	C18	110.6(6)
C16	C17	C18	126.0(6)
C17	C18	C41	128.6(6)
C19	C18	C17	104.7(6)
C19	C18	C41	126.7(6)
C18	C19	C20	109.1(6)
C1	C20	C19	125.4(6)
N4	C20	C1	125.1(6)
N4	C20	C19	109.5(6)
N5	C21	N6	108.5(6)
C23	C22	N6	106.3(6)
C22	C23	N5	107.1(6)
N7	C24	N6	114.4(7)
N7	C24	C25	125.7(7)
C25	C24	N6	119.8(7)
C26	C25	C24	117.5(8)
C25	C26	C27	119.0(8)
C28	C27	C26	117.9(8)
N7	C28	C27	124.3(8)
C3	C29	C31	108.6(7)
C30	C29	C3	114.5(7)
C30	C29	C31	105.3(8)
C32	C29	C3	109.3(7)
C32	C29	C30	111.9(9)
C32	C29	C31	106.9(8)
C34	C33	C8	111.7(7)
C34	C33	C35	108.9(8)
C34	C33	C36	112.5(8)
C35	C33	C8	106.5(7)
C36	C33	C8	109.5(7)
C36	C33	C35	107.5(8)
C13	C37	C38	111.0(7)
C13	C37	C39	109.3(7)
C13	C37	C40	111.6(7)
C38	C37	C39	107.1(7)
C40	C37	C38	109.6(7)
C40	C37	C39	108.2(7)
C18	C41	C42	108.9(6)
C18	C41	C43	111.9(7)
C18	C41	C44	109.7(6)
C42	C41	C44	106.7(7)
C43	C41	C42	109.5(7)
C43	C41	C44	110.0(7)
S2	O2	Zn2	115.6(3)
O2	Zn2	N8	97.8(2)
O2	Zn2	N10	96.0(2)
N8	Zn2	N10	166.2(2)
N9	Zn2	O2	100.5(2)
N9	Zn2	N8	90.7(2)
N9	Zn2	N10	87.5(2)
N11	Zn2	O2	98.5(2)
N11	Zn2	N8	88.4(2)
N11	Zn2	N9	160.9(2)
N11	Zn2	N10	88.7(2)
O2	S2	C91	104.1(3)
O2	S2	C92	105.6(4)

Atom	Atom	Atom	Angle/°
C92	S2	C91	97.0(4)
C48	N8	Zn2	131.9(5)
C51	N8	Zn2	121.6(5)
C51	N8	C48	105.6(6)
C53	N9	Zn2	126.4(4)
C53	N9	C56	106.3(6)
C56	N9	Zn2	126.3(5)
C58	N10	Zn2	128.5(4)
C61	N10	Zn2	123.7(4)
C61	N10	C58	106.5(5)
C63	N11	Zn2	127.2(5)
C63	N11	C66	105.8(6)
C66	N11	Zn2	125.4(4)
C67	N12	C47	124.2(6)
C67	N12	C69	108.1(6)
C69	N12	C47	126.0(6)
C67	N13	C68	109.1(6)
C67	N13	C70	125.2(6)
C68	N13	C70	125.4(6)
C74	N14	C70	116.0(7)
C48	C47	N12	118.2(6)
C66	C47	N12	109.2(6)
C66	C47	C48	132.4(6)
N8	C48	C47	115.7(6)
N8	C48	C49	110.4(6)
C47	C48	C49	133.8(6)
C48	C49	C75	136.1(7)
C50	C49	C48	103.4(6)
C50	C49	C75	120.3(7)
C49	C50	C51	109.9(6)
N8	C51	C50	110.7(6)
N8	C51	C52	126.4(7)
C52	C51	C50	122.9(7)
C51	C52	C53	130.0(6)
N9	C53	C52	121.2(6)
N9	C53	C54	111.2(6)
C52	C53	C54	127.6(6)
C53	C54	C79	128.3(7)
C55	C54	C53	104.4(6)
C55	C54	C79	127.2(7)
C54	C55	C56	108.8(6)
N9	C56	C55	109.3(6)
N9	C56	C57	126.7(6)
C57	C56	C55	124.0(6)
C58	C57	C56	127.1(7)
N10	C58	C57	122.5(6)
N10	C58	C59	109.9(6)
C57	C58	C59	127.6(7)
C58	C59	C83	128.1(6)
C60	C59	C58	105.1(6)
C60	C59	C83	126.8(7)
C59	C60	C61	107.4(6)
N10	C61	C60	111.1(6)
N10	C61	C62	125.1(6)
C62	C61	C60	123.8(6)
C63	C62	C61	128.0(6)
N11	C63	C62	123.1(6)
N11	C63	C64	111.1(6)
C62	C63	C64	125.8(7)
C63	C64	C87	129.5(7)

Atom	Atom	Atom	Angle/°
C65	C64	C63	104.8(6)
C65	C64	C87	125.7(7)
C64	C65	C66	109.0(6)
N11	C66	C47	124.8(6)
N11	C66	C65	109.2(6)
C47	C66	C65	126.0(6)
N13	C67	N12	108.0(6)
C69	C68	N13	107.4(6)
C68	C69	N12	107.4(6)
N14	C70	N13	113.5(7)
N14	C70	C71	126.1(7)
C71	C70	N13	120.4(7)
C70	C71	C72	117.2(8)
C71	C72	C73	118.7(8)
C74	C73	C72	119.5(8)
N14	C74	C73	122.6(8)
C76	C75	C49	109.6(7)
C76	C75	C77	106.2(8)
C76	C75	C78	106.1(9)
C77	C75	C49	113.6(7)
C78	C75	C49	109.1(7)
C78	C75	C77	111.9(9)
C54	C79	C80	111.2(6)
C54	C79	C81	109.1(6)
C54	C79	C82	109.6(6)
C80	C79	C81	108.0(7)
C80	C79	C82	110.8(7)
C82	C79	C81	108.0(7)
C59	C83	C84	111.8(6)
C59	C83	C85	109.8(6)
C59	C83	C86	111.0(7)
C84	C83	C85	108.0(7)
C84	C83	C86	109.5(7)
C85	C83	C86	106.6(7)
C64	C87	C88	110.3(7)
C64	C87	C89	109.4(7)
C64	C87	C90	111.3(7)
C88	C87	C89	110.5(8)
C88	C87	C90	109.2(8)
C89	C87	C90	106.1(8)
F4	B1	F3	110.3(9)
F4	B1	F1	119.6(13)
F4	B1	F2	107.7(11)
F1	B1	F3	103.8(8)
F1	B1	F2	109.4
F1	B1	F4B	78.8(14)
F2	B1	F3	105.2(8)
F2	B1	F4B	101.6(14)
F3B	B1	F1	120.1(14)
F3B	B1	F2	127.3(14)
F3B	B1	F4B	105(2)
C128	C123	C124	109.4(10)
C123	C124	C125	111.6
C126	C125	C124	111.5
C125	C126	C127	111.6
C128	C127	C126	109.2(10)
C123	C128	C127	115.5(15)
C115	C114	C113	111.6
C116	C111	C112	111.5
C111	C112	C113	111.7

Atom	Atom	Atom	Angle/°
C112	C113	C114	111.4
C116	C115	C114	111.7
C111	C116	C115	111.5
C130	C129	C134	111.5
C129	C130	C131	111.6
C130	C131	C132	111.5
C133	C132	C131	111.5
C132	C133	C134	111.6
C129	C134	C133	111.6
C121	C120	C119	111.3
C122	C117	C118	111.6
C119	C118	C117	111.6
C118	C119	C120	111.8
C120	C121	C122	111.6
C117	C122	C121	111.5
C108	C107	C106	111.6
C110	C105	C106	111.5
C107	C106	C105	111.6
C107	C108	C109	111.6
C110	C109	C108	111.6
C109	C110	C105	111.7
C101	C100	C99	111.5
C104	C99	C100	111.4

Atom	Atom	Atom	Angle/°
C100	C101	C102	111.6
C101	C102	C103	111.4
C104	C103	C102	111.5
C103	C104	C99	111.6
C98	C93	C94	111.4
C95	C94	C93	111.6
C94	C95	C96	111.6
C97	C96	C95	111.5
C96	C97	C98	111.6
C93	C98	C97	111.6
F5	B2	F7	109.5
F8	B2	F5	109.4
F8	B2	F7	109.5
F6	B2	F5	109.5
F6	B2	F8	109.4
F6	B2	F7	109.5
F5B	B2B	F8B	109.5
F5B	B2B	F7B	109.4
F6B	B2B	F5B	109.4
F6B	B2B	F8B	109.5
F6B	B2B	F7B	109.5
F8B	B2B	F7B	109.5

Table 5: Torsion Angles in ° for **Zn-4⁺**.

Atom	Atom	Atom	Atom	Angle/°
N1	C2	C3	C4	0.2(8)
N1	C2	C3	C29	175.2(8)
N1	C5	C6	C7	-0.4(15)
Zn1	N1	C2	C1	-11.4(9)
Zn1	N1	C2	C3	167.4(5)
Zn1	N1	C5	C4	-168.5(5)
Zn1	N1	C5	C6	13.4(11)
Zn1	O1	S1	C45	-103.1(4)
Zn1	O1	S1	C46	155.9(3)
Zn1	N2	C7	C6	-7.9(11)
Zn1	N2	C7	C8	171.2(5)
Zn1	N2	C10	C9	-172.2(5)
Zn1	N2	C10	C11	5.8(10)
Zn1	N3	C12	C11	-12.3(10)
Zn1	N3	C12	C13	168.9(5)
Zn1	N3	C15	C14	-169.7(5)
Zn1	N3	C15	C16	12.5(10)
Zn1	N4	C17	C16	-7.1(10)
Zn1	N4	C17	C18	172.3(4)
Zn1	N4	C20	C1	5.8(9)
Zn1	N4	C20	C19	-172.2(4)
C1	C2	C3	C4	178.7(8)
C1	C2	C3	C29	-6.3(14)
C1	N5	C21	N6	-169.1(6)
C1	N5	C23	C22	167.2(7)
N2	C7	C8	C9	0.6(9)
N2	C7	C8	C33	-179.0(7)

Atom	Atom	Atom	Atom	Angle/°
N2	C10	C11	C12	0.9(12)
C2	N1	C5	C4	1.8(8)
C2	N1	C5	C6	-176.3(8)
C2	C1	N5	C21	-93.4(8)
C2	C1	N5	C23	102.1(8)
C2	C1	C20	N4	2.0(12)
C2	C1	C20	C19	179.7(7)
C2	C3	C4	C5	0.9(9)
C2	C3	C29	C30	58.7(13)
C2	C3	C29	C31	176.0(9)
C2	C3	C29	C32	-67.7(12)
C3	C4	C5	N1	-1.7(10)
C3	C4	C5	C6	176.6(8)
N3	C12	C13	C14	0.9(8)
N3	C12	C13	C37	-179.9(7)
N3	C15	C16	C17	0.9(12)
N4	C17	C18	C19	-1.2(8)
N4	C17	C18	C41	-179.7(7)
C4	C3	C29	C30	-127.0(9)
C4	C3	C29	C31	-9.6(11)
C4	C3	C29	C32	106.7(10)
C4	C5	C6	C7	-178.2(8)
N5	C1	C2	N1	178.9(6)
N5	C1	C2	C3	0.5(11)
N5	C1	C20	N4	-176.4(6)
N5	C1	C20	C19	1.3(9)
C5	N1	C2	C1	180.0(6)
C5	N1	C2	C3	-1.2(8)
C5	C6	C7	N2	-3.4(14)
C5	C6	C7	C8	177.7(8)
C6	C7	C8	C9	179.5(8)
C6	C7	C8	C33	-0.1(13)
N6	C22	C23	N5	1.3(9)
N6	C24	C25	C26	179.9(9)
N7	C24	C25	C26	3.2(15)
C7	N2	C10	C9	-1.6(8)
C7	N2	C10	C11	176.3(7)
C7	C8	C9	C10	-1.6(9)
C7	C8	C33	C34	59.7(11)
C7	C8	C33	C35	178.5(8)
C7	C8	C33	C36	-65.5(11)
C8	C9	C10	N2	2.1(9)
C8	C9	C10	C11	-175.9(7)
C9	C8	C33	C34	-119.8(10)
C9	C8	C33	C35	-1.1(13)
C9	C8	C33	C36	114.9(10)
C9	C10	C11	C12	178.5(7)
C10	N2	C7	C6	-178.3(7)
C10	N2	C7	C8	0.7(8)
C10	C11	C12	N3	2.5(11)
C10	C11	C12	C13	-178.9(7)
C11	C12	C13	C14	-177.9(7)
C11	C12	C13	C37	1.4(12)
C12	N3	C15	C14	0.5(8)
C12	N3	C15	C16	-177.3(7)
C12	C13	C14	C15	-0.5(8)
C12	C13	C37	C38	-61.0(10)
C12	C13	C37	C39	-178.9(8)
C12	C13	C37	C40	61.5(11)
C13	C14	C15	N3	0.0(9)

Atom	Atom	Atom	Atom	Angle/˚
C13	C14	C15	C16	177.8(7)
C14	C13	C37	C38	118.1(9)
C14	C13	C37	C39	0.2(12)
C14	C13	C37	C40	-119.4(9)
C14	C15	C16	C17	-176.6(7)
C15	N3	C12	C11	178.0(6)
C15	N3	C12	C13	-0.8(8)
C15	C16	C17	N4	-4.1(12)
C15	C16	C17	C18	176.5(7)
C16	C17	C18	C19	178.3(7)
C16	C17	C18	C41	-0.3(12)
C17	N4	C20	C1	176.8(6)
C17	N4	C20	C19	-1.3(7)
C17	C18	C19	C20	0.3(8)
C17	C18	C41	C42	-175.3(7)
C17	C18	C41	C43	63.5(10)
C17	C18	C41	C44	-58.9(10)
C18	C19	C20	C1	-177.5(6)
C18	C19	C20	N4	0.6(8)
C19	C18	C41	C42	6.4(11)
C19	C18	C41	C43	-114.8(8)
C19	C18	C41	C44	122.9(8)
C20	C1	C2	N1	0.6(11)
C20	C1	C2	C3	-177.8(7)
C20	C1	N5	C21	85.2(8)
C20	C1	N5	C23	-79.2(8)
C20	N4	C17	C16	-177.9(6)
C20	N4	C17	C18	1.5(7)
C21	N5	C23	C22	0.8(9)
C21	N6	C22	C23	-2.9(9)
C21	N6	C24	N7	-165.5(7)
C21	N6	C24	C25	17.4(12)
C22	N6	C21	N5	3.5(8)
C22	N6	C24	N7	17.8(11)
C22	N6	C24	C25	-159.3(8)
C23	N5	C21	N6	-2.7(8)
C24	N6	C21	N5	-173.7(7)
C24	N6	C22	C23	174.3(7)
C24	N7	C28	C27	-0.3(15)
C24	C25	C26	C27	-1.6(16)
C25	C26	C27	C28	-0.6(17)
C26	C27	C28	N7	1.7(17)
C28	N7	C24	N6	-179.0(8)
C28	N7	C24	C25	-2.2(14)
C29	C3	C4	C5	-175.0(7)
C33	C8	C9	C10	178.0(8)
C37	C13	C14	C15	-179.8(7)
C41	C18	C19	C20	178.9(7)
Zn2	O2	S2	C91	154.1(3)
Zn2	O2	S2	C92	-104.3(4)
Zn2	N8	C48	C47	-10.6(9)
Zn2	N8	C48	C49	168.7(5)
Zn2	N8	C51	C50	-168.8(5)
Zn2	N8	C51	C52	14.2(10)
Zn2	N9	C53	C52	-9.3(9)
Zn2	N9	C53	C54	172.1(5)
Zn2	N9	C56	C55	-171.9(4)
Zn2	N9	C56	C57	4.6(10)
Zn2	N10	C58	C57	-12.7(10)
Zn2	N10	C58	C59	168.0(5)

Atom	Atom	Atom	Atom	Angle/°
Zn2	N10	C61	C60	-168.5(5)
Zn2	N10	C61	C62	15.5(9)
Zn2	N11	C63	C62	-12.1(10)
Zn2	N11	C63	C64	167.5(5)
Zn2	N11	C66	C47	9.0(9)
Zn2	N11	C66	C65	-169.0(5)
N8	C48	C49	C50	-1.3(8)
N8	C48	C49	C75	172.8(8)
N8	C51	C52	C53	1.7(13)
N9	C53	C54	C55	-1.9(8)
N9	C53	C54	C79	-179.7(7)
N9	C56	C57	C58	0.4(12)
N10	C58	C59	C60	-1.0(8)
N10	C58	C59	C83	178.7(7)
N10	C61	C62	C63	0.3(12)
N11	C63	C64	C65	0.1(8)
N11	C63	C64	C87	-178.8(8)
N12	C47	C48	N8	177.7(6)
N12	C47	C48	C49	-1.3(12)
N12	C47	C66	N11	-177.8(6)
N12	C47	C66	C65	-0.1(10)
N13	C68	C69	N12	-2.0(9)
N13	C70	C71	C72	179.2(8)
N14	C70	C71	C72	-0.2(14)
C47	N12	C67	N13	-168.0(6)
C47	N12	C69	C68	168.2(7)
C47	C48	C49	C50	177.7(8)
C47	C48	C49	C75	-8.2(15)
C48	N8	C51	C50	1.3(8)
C48	N8	C51	C52	-175.8(7)
C48	C47	C66	N11	-3.1(12)
C48	C47	C66	C65	174.6(7)
C48	C49	C50	C51	2.0(8)
C48	C49	C75	C76	178.3(10)
C48	C49	C75	C77	59.7(13)
C48	C49	C75	C78	-65.9(12)
C49	C50	C51	N8	-2.2(9)
C49	C50	C51	C52	175.0(7)
C50	C49	C75	C76	-8.4(12)
C50	C49	C75	C77	-127.0(9)
C50	C49	C75	C78	107.4(9)
C50	C51	C52	C53	-175.0(7)
C51	N8	C48	C47	-179.2(6)
C51	N8	C48	C49	0.0(8)
C51	C52	C53	N9	-4.9(12)
C51	C52	C53	C54	173.5(7)
C52	C53	C54	C55	179.5(7)
C52	C53	C54	C79	1.8(12)
C53	N9	C56	C55	-2.6(7)
C53	N9	C56	C57	173.9(7)
C53	C54	C55	C56	0.2(8)
C53	C54	C79	C80	58.9(10)
C53	C54	C79	C81	178.0(7)
C53	C54	C79	C82	-63.9(10)
C54	C55	C56	N9	1.5(8)
C54	C55	C56	C57	-175.1(7)
C55	C54	C79	C80	-118.3(9)
C55	C54	C79	C81	0.7(11)
C55	C54	C79	C82	118.8(9)
C55	C56	C57	C58	176.4(7)

Atom	Atom	Atom	Atom	Angle/°
C56	N9	C53	C52	-178.5(6)
C56	N9	C53	C54	2.8(8)
C56	C57	C58	N10	3.7(11)
C56	C57	C58	C59	-177.1(7)
C57	C58	C59	C60	179.8(7)
C57	C58	C59	C83	-0.5(13)
C58	N10	C61	C60	-0.6(8)
C58	N10	C61	C62	-176.7(6)
C58	C59	C60	C61	0.6(8)
C58	C59	C83	C84	62.6(10)
C58	C59	C83	C85	-177.5(8)
C58	C59	C83	C86	-59.9(10)
C59	C60	C61	N10	0.0(8)
C59	C60	C61	C62	176.1(7)
C60	C59	C83	C84	-117.7(9)
C60	C59	C83	C85	2.1(12)
C60	C59	C83	C86	119.7(9)
C60	C61	C62	C63	-175.3(7)
C61	N10	C58	C57	-179.7(6)
C61	N10	C58	C59	1.0(8)
C61	C62	C63	N11	-2.6(12)
C61	C62	C63	C64	177.8(7)
C62	C63	C64	C65	179.7(7)
C62	C63	C64	C87	0.9(13)
C63	N11	C66	C47	175.0(7)
C63	N11	C66	C65	-3.0(8)
C63	C64	C65	C66	-1.9(8)
C63	C64	C87	C88	65.1(11)
C63	C64	C87	C89	-173.2(9)
C63	C64	C87	C90	-56.3(11)
C64	C65	C66	N11	3.1(8)
C64	C65	C66	C47	-174.9(7)
C65	C64	C87	C88	-113.5(9)
C65	C64	C87	C89	8.2(13)
C65	C64	C87	C90	125.1(9)
C66	N11	C63	C62	-177.8(7)
C66	N11	C63	C64	1.8(8)
C66	C47	C48	N8	3.4(11)
C66	C47	C48	C49	-175.6(8)
C67	N12	C47	C48	-92.2(8)
C67	N12	C47	C66	83.4(8)
C67	N12	C69	C68	2.5(9)
C67	N13	C68	C69	0.9(9)
C67	N13	C70	N14	-165.5(7)
C67	N13	C70	C71	15.0(12)
C68	N13	C67	N12	0.7(8)
C68	N13	C70	N14	21.0(11)
C68	N13	C70	C71	-158.4(8)
C69	N12	C47	C48	104.3(8)
C69	N12	C47	C66	-80.1(8)
C69	N12	C67	N13	-2.0(8)
C70	N13	C67	N12	-173.7(7)
C70	N13	C68	C69	175.3(7)
C70	N14	C74	C73	-1.3(15)
C70	C71	C72	C73	-0.8(14)
C71	C72	C73	C74	0.7(15)
C72	C73	C74	N14	0.4(17)
C74	N14	C70	N13	-178.2(8)
C74	N14	C70	C71	1.2(14)
C75	C49	C50	C51	-173.2(7)

Atom	Atom	Atom	Atom	Angle/˚
C79	C54	C55	C56	178.1(7)
C83	C59	C60	C61	-179.1(7)
C87	C64	C65	C66	177.0(8)
C123	C124	C125	C126	-54.5
C124	C123	C128	C127	-56(2)
C124	C125	C126	C127	54.5
C125	C126	C127	C128	-53.5(12)
C126	C127	C128	C123	56(2)
C128	C123	C124	C125	53.6(12)
C114	C115	C116	C111	-54.6
C111	C112	C113	C114	54.4
C112	C111	C116	C115	54.5
C113	C114	C115	C116	54.5
C115	C114	C113	C112	-54.3
C116	C111	C112	C113	-54.6
C129	C130	C131	C132	54.5
C130	C129	C134	C133	54.5
C130	C131	C132	C133	-54.5
C131	C132	C133	C134	54.5
C132	C133	C134	C129	-54.6
C134	C129	C130	C131	-54.5
C120	C121	C122	C117	54.7
C117	C118	C119	C120	-54.3
C118	C117	C122	C121	-54.6
C119	C120	C121	C122	-54.5
C121	C120	C119	C118	54.3
C122	C117	C118	C119	54.4
C107	C108	C109	C110	54.3
C106	C107	C108	C109	-54.4
C106	C105	C110	C109	54.3
C108	C107	C106	C105	54.5
C108	C109	C110	C105	-54.3
C110	C105	C106	C107	-54.4
C100	C99	C104	C103	54.6
C100	C101	C102	C103	-54.7
C99	C100	C101	C102	54.6
C101	C100	C99	C104	-54.4
C101	C102	C103	C104	54.7
C102	C103	C104	C99	-54.8
C93	C94	C95	C96	-54.6
C94	C93	C98	C97	-54.5
C94	C95	C96	C97	54.5
C95	C96	C97	C98	-54.5
C96	C97	C98	C93	54.7
C98	C93	C94	C95	54.5

Table 6: Hydrogen Fractional Atomic Coordinates (×10[4]) and Equivalent Isotropic Displacement Parameters (Å^2×10^3) for **Zn-4⁺**. U_{eq} is defined as 1/3 of the trace of the orthogonalised U_{ij}.

Atom	x	y	z	U_{eq}
H4	1832.19	779.01	5487.73	52
H6	3103.7	995.13	5363.66	51
H9	5323.05	1899.42	5360.61	46
H11	5186.66	2878.07	5590.91	36
H14	4081.45	4416.56	6099.44	44
H16	2795.11	4190.88	6286.42	37
H19	734.29	3210.48	6534.79	38
H21	-115.39	2499.06	5589.68	36

Atom	x	y	z	U_{eq}
H22	-562.64	2322.26	6983.96	48
H23	772.25	2138.28	7029.17	47
H25	-1347.51	2352.19	5265.81	63
H26	-2608.67	2492.89	4971.81	79
H27	-3340.78	2706.31	5560.82	73
H28	-2790.03	2756.65	6406.15	69
H30A	-261.87	1533.58	5852.88	114
H30B	-487.37	1010.1	5541.91	114
H30C	-58.08	1456.91	5308.59	114
H31A	593.56	556.59	5242.23	99
H31B	206.64	297.33	5660.4	99
H31C	1084.43	327.71	5751.16	99
H32A	1106.9	758.59	6558.12	117
H32B	228.89	724.54	6460.04	117
H32C	632.04	1269.65	6595.74	117
H34A	4054.52	1031.84	4423.96	87
H34B	3537.93	757.81	4755.07	87
H34C	4173.19	433.22	4574.14	87
H35A	5356.88	1188.21	4774.8	108
H35B	5496.31	601.82	4963.51	108
H35C	5695.13	1069.28	5357.3	108
H36A	4934.63	650.2	5893.53	102
H36B	4729.42	195.45	5486.05	102
H36C	4091	511.96	5671.89	102
H38A	5932.85	3770.99	6490.26	83
H38B	6455.55	3899.44	6101.32	83
H38C	6003.9	3367.15	6052.26	83
H39A	4978.03	4771.85	5695.36	99
H39B	5853.64	4729.81	5859.92	99
H39C	5350.05	4635.86	6268.46	99
H40A	5413.84	3572.7	5146.41	82
H40B	5900.82	4089.14	5196.22	82
H40C	5027.46	4123.38	5010.44	82
H42A	308.28	4077.31	6584.46	82
H42B	636.06	4481.56	7021.82	82
H42C	779.73	3873.8	7111.23	82
H43A	1192.02	4932.59	6363.84	76
H43B	924.66	4487.98	5954.43	76
H43C	1777.8	4636.38	6104.54	76
H44A	2120.05	4109.02	7360.71	72
H44B	1909.02	4702.19	7231.58	72
H44C	2506.59	4420.35	6972.07	72
H45A	1810.1	2170.76	4212.11	85
H45B	1031.92	2033.48	4348.51	85
H45C	1774.56	1824.32	4698.76	85
H46A	1228.01	3460.99	4497.79	68
H46B	781.44	2995.77	4184.22	68
H46C	1606.68	3116.68	4127.76	68
H50	3353.45	719.4	3341.88	44
H52	2105.5	931.44	3487.89	40
H55	-191.01	1798.08	3405.24	39
H57	-91.6	2755.72	3134.51	35
H60	1009.87	4307.7	2660.65	43
H62	2313.2	4098.21	2484.72	38
H65	4364.07	3133.21	2218.11	43
H67	5230.24	2437	3151.74	37
H68	5677.51	2207.94	1771.95	50
H69	4354.77	2051.81	1710.5	49
H71	6427.78	2348.47	3473.24	56
H72	7687.09	2501.72	3763.79	59

Atom	x	y	z	U_{eq}
H73	8414.04	2683.61	3170.43	68
H74	7879.91	2724.36	2323.02	71
H76A	4570.59	497.2	3519.07	145
H76B	4959.03	241.33	3099.98	145
H76C	4080.98	256.33	3015.55	145
H77A	5384.39	1474.82	2877.73	116
H77B	5656.31	951.82	3176.55	116
H77C	5242.32	1386.61	3437.46	116
H78A	3993	650.13	2215.37	120
H78B	4868.9	637.67	2280.4	120
H78C	4431.7	1170.56	2146.27	120
H80A	1626.36	752	4105.63	80
H80B	1028.85	367.01	4255.79	80
H80C	1029.43	966.79	4407.61	80
H81A	-242.71	1109.09	3989.38	89
H81B	-305.14	500.86	3871.59	89
H81C	-541.24	910.94	3423.19	89
H82A	269.22	483.77	2947.07	83
H82B	562.36	79.31	3391.72	83
H82C	1132.91	429.88	3167.87	83
H84A	-363.9	3440.25	3568.58	79
H84B	-855.58	3954.77	3505.71	79
H84C	14.39	3993.04	3707.51	79
H85A	64.19	4641.05	3006.22	83
H85B	-809.87	4580.19	2848.54	83
H85C	-309.91	4492.13	2436.75	83
H86A	-852.8	3649.28	2217.95	74
H86B	-1372.76	3716.56	2620.37	74
H86C	-867.05	3211.93	2636.66	74
H88A	3911.33	4841.86	2501.53	97
H88B	4263.37	4375.37	2861.08	97
H88C	3398.37	4485.16	2771.26	97
H89A	4516.29	4427.83	1817.71	124
H89B	4316.17	3839.66	1658	124
H89C	4810.52	3966.5	2201.91	124
H90A	2645.38	4397.49	1865.09	93
H90B	3013.37	4118.13	1447.93	93
H90C	3261.92	4691.73	1634.03	93
H91A	3648.41	3543.15	4200.33	71
H91B	4306.48	3181.51	4479.86	71
H91C	3511.09	3137.04	4620.19	71
H92A	3462.11	2210.68	4550.09	75
H92B	4242.39	2145.32	4390.83	75
H92C	3532.71	1858.93	4073.48	75
H12A	3126.02	5365.45	68.1	170
H12B	2891.71	5965.05	40.62	170
H12C	2037.87	5126.1	330.55	125
H12D	1807.96	5467.51	-172.21	125
H12E	1614.5	6200.73	303.75	97
H12F	1147.62	5730.65	465.86	97
H12G	2033.08	5593.08	1210.69	199
H12H	1799.91	6191.59	1192.77	199
H12I	2886.93	6435.79	926.44	118
H12J	3119.82	6084.73	1423.91	118
H12K	3741.6	5815.17	786.27	86
H12L	3245.68	5359.4	937.87	86
H11A	8210.86	3865.07	1572.29	201
H11B	8213.27	4212.52	1080.62	201
H11C	6167.03	4343.02	1396.24	175
H11D	6162.87	3996.09	1888.04	175

Atom	x	y	z	U_{eq}
H11E	6272.73	3467.48	1173.49	217
H11F	6958.09	3385.36	1632.52	217
H11G	6975.19	4004.15	746.56	267
H11H	7416.22	3472.61	883.84	267
H11I	8103.06	4739.6	1796.52	285
H11J	7418.12	4822.23	1337.25	285
H11K	7401.09	4200.89	2220.9	176
H11L	6960.8	4732.94	2085.58	176
H12M	3119.89	5069.81	1081.46	415
H12N	2242.57	5123.69	957.97	415
H13A	2632.82	4995.51	189.24	92
H13B	3204.38	5465.84	309.38	92
H13C	1628.7	5570.06	82.81	127
H13D	2169.05	5761.41	-274.12	127
H13E	2648.77	6419.73	303.33	262
H13F	1771.46	6473.88	179.41	262
H13G	1686.1	6076.87	951.18	82
H13H	2257.1	6547.5	1071.89	82
H13I	3262.12	5973.19	1177.37	95
H13J	2722.54	5781.74	1534.79	95
H12O	561.57	6174.25	4064.09	152
H12P	606.96	6606.73	4495.36	152
H11M	1538.57	5911.3	5826.02	165
H11N	1493.92	5480.21	5393.67	165
H11O	285.68	5682.09	5472.46	158
H11P	428.17	6288.13	5406.25	158
H11Q	-244.92	5973.09	4632.7	255
H11R	341.56	5519.82	4623.44	255
H12Q	1815.52	6401.47	4417.86	133
H12R	1670.39	5796.15	4486.17	133
H12S	2345.13	6115.01	5259.85	165
H12T	1756.38	6567.22	5267.78	165
H10A	6350.72	3646.13	2757.77	166
H10B	5989.3	4208.34	2771.53	166
H10C	5272.14	4398.05	3516.8	150
H10D	5241.71	3939.88	3912.78	150
H10E	5920.85	3387.42	3483.14	123
H10F	5235.26	3604.87	3079.19	123
H10G	7257.17	4283.13	3101.82	172
H10H	7225.68	3824.72	3497.25	172
H10I	7265.02	4616.43	3935.26	140
H10J	6580.43	4835.54	3531.53	140
H11S	6149.01	4577.86	4255.48	115
H11T	6509.76	4015.39	4242.66	115
H10K	6621.84	5810.44	5172.81	89
H10L	6301.27	5233.53	5166.69	89
H99A	7416.3	4880.99	5029.39	103
H99B	7154.15	5314.35	4606.36	103
H10M	7097.22	5038.34	5938.45	107
H10N	6658.03	5556.85	6015.32	107
H10O	7945.09	5640.84	6377.38	106
H10P	7682.9	6072.88	5953.05	106
H10Q	8798.17	5718.59	5818.5	112
H10R	8474.95	5142.35	5811.44	112
H10S	8441.95	5397.73	4969.53	115
H10T	8002.49	5915.95	5046.94	115
H93A	9202.5	3794.42	6965.15	225
H93B	8382.92	3756.11	6637.58	225
H94A	8463.38	3209.95	7346.59	146
H94B	8777.02	3673.88	7722.99	146

Atom	x	y	z	U_{eq}
H95A	7524.96	3564.54	7743.72	198
H95B	7300.74	3607.6	7140.08	198
H96A	7038.5	4407.91	7485.17	219
H96B	7858.12	4446.66	7812.56	219
H97A	7776.55	4991.59	7104	156
H97B	7463.06	4527.29	6728.01	156
H98A	8716.73	4637.13	6707.45	212
H98B	8940.57	4593.3	7311.04	212

Table 7: Atomic Occupancies for all atoms that are not fully occupied in **Zn-4⁺**.

Atom	Occupancy
F3	0.73
F4	0.73
F3B	0.27
F4B	0.27
C123	0.61
H12A	0.61
H12B	0.61
C124	0.61
H12C	0.61
H12D	0.61
C125	0.61
H12E	0.61
H12F	0.61
C126	0.61
H12G	0.61
H12H	0.61
C127	0.61
H12I	0.61
H12J	0.61
C128	0.61
H12K	0.61
H12L	0.61
C129	0.39
H12M	0.39
H12N	0.39
C130	0.39
H13A	0.39
H13B	0.39
C131	0.39
H13C	0.39
H13D	0.39
C132	0.39
H13E	0.39
H13F	0.39
C133	0.39
H13G	0.39
H13H	0.39
C134	0.39
H13I	0.39
H13J	0.39
B2	0.73
F5	0.73
F8	0.73
F7	0.73
F6	0.73
B2B	0.27

Atom	Occupancy
F5B	0.27
F6B	0.27
F8B	0.27
F7B	0.27

R_1=6.58%

Crystal Data and Experimental

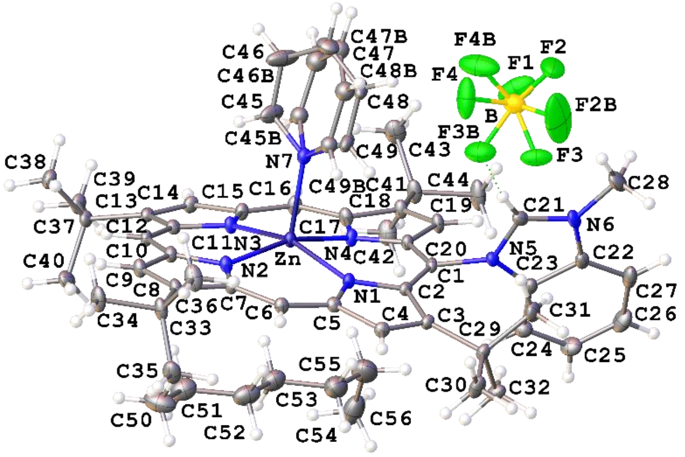

Experimental. Single clear light red prism-shaped crystals of **Zn-5+** recrystallised from a mixture of toluene and pyridine by slow evaporation. A suitable crystal with dimensions 0.40 × 0.20 × 0.20 mm^3 was selected and mounted on a mylar loop with oil on a Bruker APEX-II CCD diffractometer. The crystal was kept at a steady T = 110 K during data collection. The structure was solved with the ShelXT 2018/[6] solution program using dual methods and by using Olex2 1.5[11] as the graphical interface. The model was refined with **XL**[8] using full matrix least squares minimisation on F^2.

Crystal Data. $C_{56}H_{72}BF_4N_7Zn$, M_r = 995.38, monoclinic, $C2/c$ (No. 15), a = 20.5490(14) Å, b = 12.2656(8) Å, c = 43.860(3) Å, β = 103.259(2)°, α = γ = 90°, V = 10760.1(12) Å^3, T = 110 K, Z = 8, Z' = 1, μ(Mo K$_\alpha$) = 0.513, 126297 reflections measured, 12358 unique (R_{int} = 0.0526) which were used in all calculations. The final wR_2 was 0.1389 (all data) and R_1 was 0.0658 (I≥2 σ(I)).

**Compound**	**Zn-5+**
**CCDC**	**2363633**
Formula	$C_{56}H_{72}BF_4N_7Zn$
$D_{calc.}$/ g cm^{-3}	1.229
μ/mm^{-1}	0.513
Formula Weight	995.38
Colour	clear light red
Shape	prism-shaped
Size/mm^3	0.40×0.20×0.20
T/K	110
Crystal System	monoclinic
Space Group	$C2/c$
a/Å	20.5490(14)
b/Å	12.2656(8)
c/Å	43.860(3)
α/°	90
β/°	103.259(2)
γ/°	90
V/Å^3	10760.1(12)
Z	8
Z'	1
Wavelength/Å	0.71073
Radiation type	Mo K$_\alpha$
Θ_{min}/°	2.064
Θ_{max}/°	27.505
Measured Refl's.	126297
Indep't Refl's	12358
Refl's I≥2 σ(I)	10695
R_{int}	0.0526
Parameters	684
Restraints	0
Largest Peak	1.046
Deepest Hole	-0.578
GooF	1.223
wR_2 (all data)	0.1389
wR_2	0.1352
R_1 (all data)	0.0766
R_1	0.0658

A clear light red prism-shaped-shaped crystal with dimensions 0.40 × 0.20 × 0.20 mm^3 was mounted on a mylar loop with oil. Data were collected using a Bruker APEX-II CCD diffractometer equipped with an Oxford Cryosystems low-temperature device operating at T = 110 K.

Data were measured using ϕ and ω scans with Mo K_{α} radiation. The maximum resolution that was achieved was Θ = 27.505° (0.77 Å). The unit cell was refined using SAINT V8.40B[10] on 9921 reflections, 8% of the observed reflections.

Data reduction, scaling and absorption corrections were performed using SAINT V8.[10]. The final completeness is 99.80 % out to 27.505° in Θ. SADABS-2016/2 [10] was used for absorption correction. wR_2(int) was 0.0569 before and 0.0518 after correction. The Ratio of minimum to maximum transmission is 0.9406. The absorption coefficient μ of this material is 0.513 mm^{-1} at this wavelength (λ = 0.71073Å) and the minimum and maximum transmissions are 0.701 and 0.746.

The structure was solved and the space group $C2/c$ (# 15) determined by the ShelXT 2018/[6] structure solution program using using dual methods and refined by full matrix least squares minimisation on $\boldsymbol{F^2}$ using version 2019/3 of ShelXL 2019/[8]. All non-hydrogen atoms were refined anisotropically. Hydrogen atom positions were calculated geometrically and refined using the riding model. The tetraflouoroborate was found disordered over several postions and three fluor atom were modelize over two positions with occupation factors converged to 0.60 :0.40. The pyridin coordinated to the Zn was also found disordered over two positions with occupation factors converged to 0.76 :0.24, the minor part being isotropically refined.

Table 8: Fractional Atomic Coordinates (×10^4) and Equivalent Isotropic Displacement Parameters (Å^2×10^3) for **Zn-5$^+$**. U_{eq} is defined as 1/3 of the trace of the orthogonalised U_{ij}.

**Atom**	**x**	**y**	**z**	$\boldsymbol{U_{eq}}$
Zn	3708.3(2)	3967.0(3)	6160.4(2)	19.20(9)
B	1744(2)	1829(4)	7128.5(10)	41.6(10)
N1	3137.4(12)	2537(2)	6056.6(6)	19.6(5)
F1	2361.1(13)	1993(3)	7311.7(8)	86.4(11)
C1	3671.6(14)	1431(2)	6488.2(7)	19.2(6)
F2B	1435(10)	956(12)	7146(3)	189(8)
N2	3188.4(12)	4672(2)	5750.0(6)	21.4(5)
C2	3180.4(14)	1547(2)	6209.7(7)	18.5(6)
F2	1338(3)	1609(5)	7338.5(14)	85.9(19)
C3	2658.1(14)	794(2)	6036.1(7)	20.1(6)
F3	1736.2(19)	876(5)	6955.5(11)	55.3(13)
N3	4457.5(12)	5121(2)	6170.0(6)	21.4(5)
F3B	1923(4)	1840(7)	6836.3(16)	68(2)
C21	3211.4(16)	529(3)	6897.2(7)	26.3(7)
F4B	1397(5)	2805(11)	7126(2)	95(4)
N4	4376.7(12)	3052(2)	6483.2(6)	21.9(5)
C4	2316.6(14)	1412(2)	5793.9(7)	22.4(6)
F4	1457(3)	2646(5)	6948.8(17)	87(2)
N5	3620.4(12)	534(2)	6699.3(6)	20.9(5)
C5	2604.1(14)	2472(2)	5805.7(7)	21.3(6)
N6	3351.6(14)	-326(2)	7090.3(6)	29.5(6)
C6	2345.8(14)	3277(2)	5588.1(7)	22.5(6)
C7	2597.6(14)	4319(2)	5561.0(7)	20.0(6)
N7	3209.5(13)	4663(2)	6488.6(6)	24.7(5)
C8	2327.5(15)	5160(3)	5329.0(7)	23.6(6)
C9	2778.9(14)	5977(3)	5384.3(7)	25.8(6)
C10	3317.0(15)	5672(2)	5642.4(7)	22.3(6)
C11	3896.9(14)	6281(2)	5742.6(7)	22.2(6)
C12	4450.6(14)	6005(3)	5978.9(7)	21.2(6)

Atom	x	y	z	U_{eq}
C13	5086.3(14)	6595(2)	6066.3(7)	20.4(6)
C14	5451.8(14)	6043(3)	6316.5(7)	24.4(6)
C15	5065.2(14)	5128(2)	6377.0(7)	22.7(6)
C16	5286.3(15)	4372(3)	6613.0(7)	27.0(7)
C17	4972.8(15)	3412(3)	6666.7(7)	25.1(7)
C18	5215.1(15)	2644(3)	6921.0(7)	25.9(7)
C19	4736.6(15)	1865(3)	6888.7(7)	25.9(7)
C20	4222.5(14)	2106(2)	6612.6(7)	20.4(6)
C22	3876.8(16)	-891(3)	7025.1(7)	27.4(7)
C23	4057.2(16)	-353(2)	6778.1(7)	24.3(6)
C24	4604.7(17)	-690(3)	6661.9(8)	32.6(7)
C25	4933.1(18)	-1627(3)	6793.3(9)	39.3(9)
C26	4752(2)	-2159(3)	7039.3(10)	45.9(10)
C27	4221(2)	-1815(3)	7163.1(8)	39.8(9)
C28	2989(2)	-609(3)	7333.6(9)	45.8(10)
C29	2530.6(15)	-434(2)	6054.6(7)	24.5(6)
C30	1967.0(18)	-771(3)	5774.3(9)	37.4(8)
C31	2306.0(19)	-785(3)	6352.6(8)	35.8(8)
C32	3156.0(17)	-1061(3)	6026.0(8)	33.3(7)
C33	1689.7(16)	5113(3)	5069.7(8)	32.0(8)
C34	1595.5(18)	6216(3)	4898.4(9)	41.3(9)
C35	1755(2)	4229(3)	4832.2(8)	43.3(9)
C36	1075.1(17)	4917(4)	5203.3(9)	44.2(10)
C37	5323.9(15)	7533(2)	5895.0(7)	24.3(6)
C38	4878.3(17)	8540(3)	5877.3(8)	31.5(7)
C39	6033.6(15)	7867(3)	6068.3(8)	30.9(7)
C40	5340.2(18)	7151(3)	5563.1(8)	35.9(8)
C41	5877.0(16)	2663(3)	7162.6(8)	34.0(8)
C42	6462.4(17)	2661(3)	6997.5(9)	43.1(9)
C43	5932(2)	3646(3)	7380.9(9)	47.3(10)
C44	5941.9(19)	1622(4)	7361.3(10)	50.8(12)
C45B	3612(8)	5222(13)	6733(4)	31(3)
C45	3345(3)	5657(5)	6602.0(15)	48.3(15)
C46	3076(3)	6100(6)	6837.4(17)	69(2)
C46B	3386(9)	5549(15)	6988(4)	43(4)
C47	2629(4)	5479(8)	6953.3(19)	44.9(18)
C47B	2782(12)	5320(20)	7013(6)	33(6)
C48B	2343(12)	4677(19)	6745(5)	36(6)
C48	2476(4)	4494(5)	6840.5(18)	44.9(17)
C49B	2646(9)	4381(15)	6510(4)	29(4)
C49	2753(3)	4104(4)	6603.7(15)	36.8(13)
C50	3946(2)	4212(5)	5056.0(11)	71.2(16)
C51	4581(2)	4017(4)	5303.7(11)	59.0(12)
C52	4569(2)	2948(4)	5476.1(11)	55.5(12)
C53	5171(2)	2722(4)	5736.5(11)	55.9(11)
C54	5144(3)	1657(4)	5900.2(12)	63.9(13)
C55	5732(3)	1419(5)	6170.1(12)	79.1(17)
C56	6382(3)	1190(4)	6077.4(14)	76.2(16)

Table 9: Anisotropic Displacement Parameters (×10[4]) for **Zn-5⁺**. The anisotropic displacement factor exponent takes the form: $-2\pi^2[h^2a^{*2} \times U_{11} + \ldots + 2hka^* \times b^* \times U_{12}]$

Atom	U_{11}	U_{22}	U_{33}	U_{23}	U_{13}	U_{12}
Zn	15.61(15)	19.43(16)	21.16(16)	3.80(14)	1.33(12)	-3.37(14)
B	35(2)	52(3)	34(2)	-3(2)	0.6(18)	2(2)
N1	17.5(11)	18.1(12)	22.5(12)	3.2(10)	3.0(9)	-3.3(9)
F1	49.7(16)	80(2)	104(2)	-40.3(18)	-35.4(16)	17.7(15)
C1	19.3(13)	16.6(13)	22.5(14)	5.2(11)	6.3(11)	1.0(11)

Atom	U_{11}	U_{22}	U_{33}	U_{23}	U_{13}	U_{12}
F2B	269(19)	136(12)	134(12)	27(10)	-12(12)	-153(13)
N2	16.7(11)	24.0(13)	22.5(12)	5.1(10)	2.3(10)	-3.8(10)
C2	16.2(13)	16.8(13)	23.5(14)	-0.2(11)	6.5(11)	-3.5(11)
F2	134(5)	66(4)	83(4)	4(3)	79(4)	17(3)
C3	17.2(13)	17.1(14)	26.2(15)	-0.5(11)	5.5(11)	-3.0(11)
F3	30.3(19)	78(4)	54(3)	-35(3)	3.0(18)	3(2)
N3	17.4(12)	23.3(13)	22.3(12)	5.3(10)	2.1(10)	-5.9(10)
F3B	58(4)	84(5)	62(4)	-19(4)	12(3)	16(4)
C21	26.9(16)	27.7(16)	26.5(16)	-5.6(13)	10.9(13)	-10.4(13)
F4B	63(5)	153(10)	63(6)	-32(6)	0(5)	50(6)
N4	17.3(12)	19.2(12)	26.8(13)	6.1(10)	0.1(10)	-4.8(10)
C4	16.2(13)	24.6(15)	25.0(15)	-1.2(12)	2.0(11)	-3.4(11)
F4	49(3)	79(4)	114(5)	60(4)	-19(4)	-14(3)
N5	21.8(12)	21.4(12)	20.2(12)	0.7(10)	5.9(10)	-4.7(10)
C5	14.6(13)	25.1(15)	23.9(15)	0.5(12)	3.9(11)	-2.4(11)
N6	35.5(15)	29.5(14)	24.2(13)	3.4(11)	8.2(12)	-15.1(12)
C6	16.9(13)	24.8(15)	24.4(15)	1.7(12)	1.9(11)	-4.7(11)
C7	14.9(13)	22.1(14)	22.1(14)	4.9(11)	2.3(11)	-2.4(11)
N7	25.4(13)	21.9(13)	26.8(13)	1.7(10)	6.0(11)	-4.2(10)
C8	19.9(14)	27.3(16)	22.8(15)	8.9(12)	3.2(12)	-1.7(12)
C9	22.0(14)	27.9(16)	25.6(15)	8.4(13)	1.8(12)	-2.2(13)
C10	20.7(14)	22.4(14)	23.3(15)	5.0(12)	3.9(12)	-2.0(11)
C11	22.9(14)	19.0(14)	24.9(15)	5.4(11)	5.7(12)	-3.1(11)
C12	17.7(13)	23.6(14)	23.3(14)	1.4(12)	6.9(11)	-2.0(12)
C13	19.2(14)	19.3(14)	24.3(15)	0.1(11)	8.2(11)	-1.8(11)
C14	17.9(13)	22.3(14)	31.9(16)	0.0(13)	3.2(12)	-5.2(12)
C15	17.8(14)	21.6(15)	27.3(15)	4.2(12)	2.4(12)	-3.8(11)
C16	19.5(14)	27.1(16)	29.7(16)	5.7(13)	-3.8(12)	-6.8(12)
C17	19.0(14)	30.1(17)	22.8(15)	7.8(13)	-2.3(12)	-3.2(12)
C18	20.6(14)	27.9(16)	25.4(15)	8.1(13)	-2.5(12)	-4.7(12)
C19	22.4(15)	28.3(16)	24.2(15)	10.1(13)	-0.1(12)	-5.3(13)
C20	19.4(14)	18.5(14)	22.8(14)	4.2(11)	3.9(11)	-2.5(11)
C22	33.8(17)	23.1(16)	23.6(15)	0.0(12)	2.8(13)	-9.8(13)
C23	29.1(16)	20.3(14)	21.1(15)	1.5(12)	1.3(12)	-3.8(12)
C24	29.8(17)	34.8(18)	33.3(18)	-0.5(14)	7.4(14)	2.5(14)
C25	31.3(18)	44(2)	41(2)	-1.5(17)	3.7(16)	13.0(16)
C26	44(2)	33(2)	52(2)	5.3(18)	-8.2(18)	8.4(17)
C27	52(2)	28.9(18)	31.0(18)	8.6(15)	-6.2(16)	-7.8(17)
C28	57(2)	51(2)	37(2)	-1.1(18)	25.4(18)	-23(2)
C29	24.3(15)	17.2(14)	30.2(16)	0.0(12)	2.6(13)	-3.2(12)
C30	37.4(19)	22.4(17)	44(2)	-0.3(14)	-7.9(16)	-8.9(14)
C31	42(2)	25.4(17)	42(2)	3.6(14)	12.5(16)	-13.5(15)
C32	35.0(18)	28.1(17)	32.9(17)	-5.3(15)	0.0(14)	6.4(15)
C33	24.5(16)	37.4(19)	29.3(17)	15.3(14)	-3.8(13)	-7.7(14)
C34	34.9(19)	44(2)	36(2)	19.5(17)	-9.8(15)	-7.0(16)
C35	48(2)	46(2)	29.6(18)	3.5(16)	-4.3(16)	-14.1(18)
C36	20.0(16)	59(3)	49(2)	22(2)	-2.1(15)	-3.2(16)
C37	23.2(15)	21.0(15)	30.3(16)	3.4(12)	9.6(13)	-6.6(12)
C38	32.0(17)	22.6(16)	39.3(19)	4.7(14)	6.9(15)	-1.9(14)
C39	23.3(16)	25.3(16)	45(2)	4.9(14)	10.5(14)	-7.7(13)
C40	41(2)	37.3(19)	35.2(19)	0.6(15)	19.9(16)	-11.2(16)
C41	25.4(16)	38.9(19)	29.7(17)	12.9(15)	-10.0(13)	-11.3(14)
C42	23.0(17)	53(2)	47(2)	15.5(19)	-6.0(15)	-2.5(16)
C43	41(2)	56(3)	36(2)	0.9(18)	-8.8(17)	-15.6(19)
C44	37(2)	55(3)	48(2)	29(2)	-16.9(18)	-16.5(19)
C45	45(3)	38(3)	71(4)	-22(3)	32(3)	-23(3)
C46	57(4)	70(4)	91(5)	-54(4)	39(4)	-30(4)
C47	38(4)	65(5)	35(4)	-2(3)	14(3)	10(4)
C48	57(4)	34(3)	56(4)	16(3)	39(4)	11(3)
C49	47(3)	18(2)	54(4)	4(3)	29(3)	-3(2)

Atom	U_{11}	U_{22}	U_{33}	U_{23}	U_{13}	U_{12}
C50	56(3)	107(5)	50(3)	-14(3)	13(2)	18(3)
C51	51(3)	59(3)	66(3)	-11(2)	10(2)	1(2)
C52	45(2)	54(3)	68(3)	-23(2)	13(2)	-9(2)
C53	60(3)	46(3)	60(3)	-12(2)	11(2)	-7(2)
C54	80(4)	56(3)	61(3)	-6(2)	26(3)	-9(3)
C55	112(5)	72(4)	54(3)	-11(3)	19(3)	-12(3)
C56	90(4)	44(3)	82(4)	4(3)	-8(3)	-13(3)

Table 10: Bond Lengths in Å for **Zn-5⁺**.

Atom	Atom	Length/Å
Zn	N1	2.101(2)
Zn	N2	2.061(2)
Zn	N3	2.084(2)
Zn	N4	2.064(2)
Zn	N7	2.128(3)
B	F1	1.351(5)
B	F2B	1.257(11)
B	F2	1.403(7)
B	F3	1.391(6)
B	F3B	1.412(8)
B	F4B	1.392(12)
B	F4	1.327(7)
N1	C2	1.381(4)
N1	C5	1.366(4)
C1	C2	1.402(4)
C1	N5	1.458(4)
C1	C20	1.407(4)
N2	C7	1.374(4)
N2	C10	1.361(4)
C2	C3	1.486(4)
C3	C4	1.361(4)
C3	C29	1.534(4)
N3	C12	1.369(4)
N3	C15	1.365(4)
C21	N5	1.339(4)
C21	N6	1.337(4)
N4	C17	1.375(4)
N4	C20	1.361(4)
C4	C5	1.424(4)
N5	C23	1.402(4)
C5	C6	1.391(4)
N6	C22	1.367(4)
N6	C28	1.476(4)
C6	C7	1.393(4)
C7	C8	1.466(4)
N7	C45B	1.378(15)
N7	C45	1.323(6)
N7	C49B	1.233(19)
N7	C49	1.349(6)
C8	C9	1.349(4)
C8	C33	1.527(4)
C9	C10	1.439(4)
C10	C11	1.390(4)

Atom	Atom	Length/Å
C11	C12	1.394(4)
C12	C13	1.465(4)
C13	C14	1.359(4)
C13	C37	1.515(4)
C14	C15	1.435(4)
C15	C16	1.386(4)
C16	C17	1.388(4)
C17	C18	1.457(4)
C18	C19	1.355(4)
C18	C41	1.521(4)
C19	C20	1.443(4)
C22	C23	1.389(4)
C22	C27	1.399(5)
C23	C24	1.400(5)
C24	C25	1.389(5)
C25	C26	1.383(6)
C26	C27	1.392(6)
C29	C30	1.540(4)
C29	C31	1.544(5)
C29	C32	1.527(4)
C33	C34	1.538(5)
C33	C35	1.531(5)
C33	C36	1.528(5)
C37	C38	1.528(4)
C37	C39	1.537(4)
C37	C40	1.537(4)
C41	C42	1.540(5)
C41	C43	1.528(6)
C41	C44	1.534(5)
C45B	C46B	1.36(2)
C45	C46	1.389(7)
C46	C47	1.378(11)
C46B	C47B	1.30(3)
C47	C48	1.316(11)
C47B	C48B	1.53(3)
C48B	C49B	1.37(3)
C48	C49	1.380(8)
C50	C51	1.512(6)
C51	C52	1.516(7)
C52	C53	1.505(6)
C53	C54	1.497(7)
C54	C55	1.513(7)
C55	C56	1.509(8)

Table 11: Bond Angles in ° for **Zn-5⁺**.

Atom	Atom	Atom	Angle/°
N1	Zn	N7	98.62(10)
N2	Zn	N1	90.54(10)
N2	Zn	N3	88.00(9)
N2	Zn	N4	163.26(10)
N2	Zn	N7	100.88(10)
N3	Zn	N1	160.76(10)
N3	Zn	N7	100.49(10)
N4	Zn	N1	86.86(9)
N4	Zn	N3	89.04(9)
N4	Zn	N7	95.87(10)
F1	B	F2	104.8(5)
F1	B	F3	109.9(4)
F1	B	F3B	97.7(4)
F1	B	F4B	106.6(6)
F2B	B	F1	120.5(9)
F2B	B	F3B	107.8(10)
F2B	B	F4B	117.9(12)
F3	B	F2	105.2(5)
F4B	B	F3B	102.9(6)
F4	B	F1	118.1(5)
F4	B	F2	106.6(5)
F4	B	F3	111.2(5)
C2	N1	Zn	131.19(19)
C5	N1	Zn	121.9(2)
C5	N1	C2	106.9(2)
C2	C1	N5	119.2(2)
C2	C1	C20	129.3(3)
C20	C1	N5	111.5(2)
C7	N2	Zn	127.13(19)
C10	N2	Zn	125.3(2)
C10	N2	C7	106.9(2)
N1	C2	C1	117.8(2)
N1	C2	C3	109.8(2)
C1	C2	C3	132.4(3)
C2	C3	C29	134.0(3)
C4	C3	C2	103.7(2)
C4	C3	C29	121.6(3)
C12	N3	Zn	128.45(19)
C15	N3	Zn	125.21(19)
C15	N3	C12	106.3(2)
N6	C21	N5	109.8(3)
C17	N4	Zn	126.2(2)
C20	N4	Zn	124.61(19)
C20	N4	C17	106.9(2)
C3	C4	C5	110.2(3)
C21	N5	C1	124.2(3)
C21	N5	C23	107.4(3)
C23	N5	C1	127.3(2)
N1	C5	C4	109.4(3)
N1	C5	C6	128.4(3)
C6	C5	C4	122.2(3)
C21	N6	C22	109.3(3)
C21	N6	C28	124.9(3)
C22	N6	C28	125.8(3)
C5	C6	C7	128.2(3)
N2	C7	C6	122.1(3)
N2	C7	C8	109.8(2)
C6	C7	C8	128.0(3)
C45B	N7	Zn	115.4(7)
C45	N7	Zn	122.1(3)
C45	N7	C49	115.6(4)
C49B	N7	Zn	123.1(9)
C49B	N7	C45B	119.1(11)
C49	N7	Zn	122.2(3)
C7	C8	C33	127.9(3)
C9	C8	C7	105.2(3)
C9	C8	C33	126.8(3)
C8	C9	C10	108.7(3)
N2	C10	C9	109.4(3)
N2	C10	C11	126.5(3)
C11	C10	C9	123.9(3)
C10	C11	C12	126.8(3)
N3	C12	C11	122.7(3)
N3	C12	C13	110.3(2)
C11	C12	C13	127.0(3)
C12	C13	C37	127.9(3)
C14	C13	C12	105.1(3)
C14	C13	C37	126.7(3)
C13	C14	C15	108.2(3)
N3	C15	C14	110.1(3)
N3	C15	C16	125.8(3)
C16	C15	C14	124.1(3)
C15	C16	C17	127.5(3)
N4	C17	C16	124.0(3)
N4	C17	C18	110.0(3)
C16	C17	C18	126.0(3)
C17	C18	C41	128.1(3)
C19	C18	C17	105.3(3)
C19	C18	C41	126.5(3)
C18	C19	C20	108.5(3)
C1	C20	C19	125.0(3)
N4	C20	C1	125.7(3)
N4	C20	C19	109.2(2)
N6	C22	C23	106.7(3)
N6	C22	C27	131.7(3)
C23	C22	C27	121.6(3)
C22	C23	N5	106.9(3)
C22	C23	C24	121.6(3)
C24	C23	N5	131.5(3)
C25	C24	C23	116.6(3)
C26	C25	C24	121.5(3)
C25	C26	C27	122.4(3)
C26	C27	C22	116.2(3)
C3	C29	C30	109.1(3)
C3	C29	C31	114.0(3)
C30	C29	C31	106.6(3)
C32	C29	C3	109.4(3)
C32	C29	C30	107.0(3)
C32	C29	C31	110.5(3)
C8	C33	C34	108.7(3)
C8	C33	C35	109.9(3)
C8	C33	C36	111.4(3)
C35	C33	C34	108.1(3)
C36	C33	C34	107.6(3)
C36	C33	C35	111.0(3)
C13	C37	C38	112.4(2)

Atom	Atom	Atom	Angle/°
C13	C37	C39	109.5(3)
C13	C37	C40	108.5(3)
C38	C37	C39	107.4(3)
C38	C37	C40	109.9(3)
C40	C37	C39	109.0(3)
C18	C41	C42	110.0(3)
C18	C41	C43	112.0(3)
C18	C41	C44	109.3(3)
C43	C41	C42	110.0(3)
C43	C41	C44	108.5(3)
C44	C41	C42	106.9(3)
C46B	C45B	N7	121.8(14)
N7	C45	C46	123.7(5)
C47	C46	C45	118.1(6)
C47B	C46B	C45B	121(2)
C48	C47	C46	119.4(7)
C46B	C47B	C48B	117(2)
C49B	C48B	C47B	115(2)
C47	C48	C49	119.8(6)
N7	C49B	C48B	125.5(18)
N7	C49	C48	123.2(5)
C50	C51	C52	112.7(4)
C53	C52	C51	115.7(4)
C54	C53	C52	114.2(4)
C53	C54	C55	115.6(5)
C56	C55	C54	115.0(5)

Table 12: Torsion Angles in ° for **Zn-5⁺**.

Atom	Atom	Atom	Atom	Angle/°
Zn	N1	C2	C1	1.6(4)
Zn	N1	C2	C3	-179.36(19)
Zn	N1	C5	C4	179.91(19)
Zn	N1	C5	C6	-0.9(4)
Zn	N2	C7	C6	-14.9(4)
Zn	N2	C7	C8	168.6(2)
Zn	N2	C10	C9	-168.9(2)
Zn	N2	C10	C11	16.2(4)
Zn	N3	C12	C11	1.8(4)
Zn	N3	C12	C13	-179.5(2)
Zn	N3	C15	C14	-179.6(2)
Zn	N3	C15	C16	0.3(5)
Zn	N4	C17	C16	-15.2(5)
Zn	N4	C17	C18	164.6(2)
Zn	N4	C20	C1	20.3(4)
Zn	N4	C20	C19	-163.2(2)
Zn	N7	C45B	C46B	-168.6(13)
Zn	N7	C45	C46	-173.8(6)
Zn	N7	C49B	C48B	169.7(14)
Zn	N7	C49	C48	172.9(5)
N1	C2	C3	C4	-2.3(3)
N1	C2	C3	C29	167.7(3)
N1	C5	C6	C7	5.0(5)
C1	C2	C3	C4	176.5(3)
C1	C2	C3	C29	-13.4(6)
C1	N5	C23	C22	169.9(3)
C1	N5	C23	C24	-7.0(5)
N2	C7	C8	C9	1.4(4)
N2	C7	C8	C33	178.9(3)
N2	C10	C11	C12	-2.2(5)
C2	N1	C5	C4	-1.8(3)
C2	N1	C5	C6	177.4(3)
C2	C1	N5	C21	-78.6(4)
C2	C1	N5	C23	114.7(3)
C2	C1	C20	N4	1.8(5)
C2	C1	C20	C19	-174.1(3)
C2	C3	C4	C5	1.2(3)
C2	C3	C29	C30	-170.8(3)
C2	C3	C29	C31	70.3(4)
C2	C3	C29	C32	-54.0(4)
C3	C4	C5	N1	0.3(4)

Atom	Atom	Atom	Atom	Angle/°
C3	C4	C5	C6	-179.0(3)
N3	C12	C13	C14	-1.4(3)
N3	C12	C13	C37	172.1(3)
N3	C15	C16	C17	7.1(6)
C21	N5	C23	C22	1.4(3)
C21	N5	C23	C24	-175.6(3)
C21	N6	C22	C23	-0.6(3)
C21	N6	C22	C27	178.1(3)
N4	C17	C18	C19	-2.6(4)
N4	C17	C18	C41	174.5(3)
C4	C3	C29	C30	-2.1(4)
C4	C3	C29	C31	-121.1(3)
C4	C3	C29	C32	114.6(3)
C4	C5	C6	C7	-175.9(3)
N5	C1	C2	N1	163.3(2)
N5	C1	C2	C3	-15.5(5)
N5	C1	C20	N4	-174.9(3)
N5	C1	C20	C19	9.1(4)
N5	C21	N6	C22	1.5(4)
N5	C21	N6	C28	-178.2(3)
N5	C23	C24	C25	-179.7(3)
C5	N1	C2	C1	-176.5(3)
C5	N1	C2	C3	2.6(3)
C5	C6	C7	N2	3.5(5)
C5	C6	C7	C8	179.2(3)
N6	C21	N5	C1	-170.7(3)
N6	C21	N5	C23	-1.7(3)
N6	C22	C23	N5	-0.5(3)
N6	C22	C23	C24	176.8(3)
N6	C22	C27	C26	-178.4(3)
C6	C7	C8	C9	-174.7(3)
C6	C7	C8	C33	2.7(5)
C7	N2	C10	C9	2.1(3)
C7	N2	C10	C11	-172.7(3)
C7	C8	C9	C10	-0.1(4)
C7	C8	C33	C34	176.8(3)
C7	C8	C33	C35	-65.1(4)
C7	C8	C33	C36	58.4(5)
N7	C45B	C46B	C47B	2(3)
N7	C45	C46	C47	-1.8(12)
C8	C9	C10	N2	-1.2(4)
C8	C9	C10	C11	173.8(3)
C9	C8	C33	C34	-6.3(5)
C9	C8	C33	C35	111.9(4)
C9	C8	C33	C36	-124.7(4)
C9	C10	C11	C12	-176.3(3)
C10	N2	C7	C6	174.3(3)
C10	N2	C7	C8	-2.2(3)
C10	C11	C12	N3	-7.5(5)
C10	C11	C12	C13	174.0(3)
C11	C12	C13	C14	177.2(3)
C11	C12	C13	C37	-9.3(5)
C12	N3	C15	C14	0.3(3)
C12	N3	C15	C16	-179.8(3)
C12	C13	C14	C15	1.5(3)
C12	C13	C37	C38	62.5(4)
C12	C13	C37	C39	-178.2(3)
C12	C13	C37	C40	-59.3(4)
C13	C14	C15	N3	-1.2(4)
C13	C14	C15	C16	178.9(3)

Atom	Atom	Atom	Atom	Angle/°
C14	C13	C37	C38	-125.4(3)
C14	C13	C37	C39	-6.1(4)
C14	C13	C37	C40	112.8(4)
C14	C15	C16	C17	-173.0(3)
C15	N3	C12	C11	-178.0(3)
C15	N3	C12	C13	0.6(3)
C15	C16	C17	N4	0.9(6)
C15	C16	C17	C18	-178.9(3)
C16	C17	C18	C19	177.3(3)
C16	C17	C18	C41	-5.7(6)
C17	N4	C20	C1	-176.1(3)
C17	N4	C20	C19	0.4(3)
C17	C18	C19	C20	2.7(4)
C17	C18	C41	C42	-56.9(5)
C17	C18	C41	C43	65.8(5)
C17	C18	C41	C44	-174.0(4)
C18	C19	C20	C1	174.4(3)
C18	C19	C20	N4	-2.1(4)
C19	C18	C41	C42	119.5(4)
C19	C18	C41	C43	-117.8(4)
C19	C18	C41	C44	2.5(5)
C20	C1	C2	N1	-13.2(5)
C20	C1	C2	C3	168.0(3)
C20	C1	N5	C21	98.5(3)
C20	C1	N5	C23	-68.2(4)
C20	N4	C17	C16	-178.6(3)
C20	N4	C17	C18	1.3(4)
C22	C23	C24	C25	3.8(5)
C23	C22	C27	C26	0.1(5)
C23	C24	C25	C26	-3.8(5)
C24	C25	C26	C27	2.1(6)
C25	C26	C27	C22	-0.2(6)
C27	C22	C23	N5	-179.3(3)
C27	C22	C23	C24	-2.0(5)
C28	N6	C22	C23	179.1(3)
C28	N6	C22	C27	-2.3(6)
C29	C3	C4	C5	-170.4(3)
C33	C8	C9	C10	-177.7(3)
C37	C13	C14	C15	-172.1(3)
C41	C18	C19	C20	-174.4(3)
C45B	N7	C49B	C48B	8(2)
C45B	C46B	C47B	C48B	0(3)
C45	N7	C49	C48	-4.6(9)
C45	C46	C47	C48	0.5(13)
C46	C47	C48	C49	-1.4(13)
C46B	C47B	C48B	C49B	2(3)
C47	C48	C49	N7	3.6(12)
C47B	C48B	C49B	N7	-6(3)
C49B	N7	C45B	C46B	-5(2)
C49	N7	C45	C46	3.7(9)
C50	C51	C52	C53	-177.3(4)
C51	C52	C53	C54	-179.7(4)
C52	C53	C54	C55	-178.2(4)
C53	C54	C55	C56	-69.8(6)

Table 13: Hydrogen Fractional Atomic Coordinates (×10[4]) and Equivalent Isotropic Displacement Parameters (Å^2×10^3) for **Zn-5⁺**. U_{eq} is defined as 1/3 of the trace of the orthogonalised U_{ij}.

Atom	x	y	z	U_{eq}
H21	2872.73	1053.7	6899.91	32
H4	1941.01	1170.73	5639.38	27
H6	1947.34	3093.89	5439.11	27
H9	2746.72	6643.07	5271.49	31
H11	3918.15	6956.44	5639.2	27
H14	5887.77	6231.08	6431.3	29
H16	5700.15	4529.48	6754.28	32
H19	4739.16	1261.74	7024.84	31
H24	4744.58	-298.12	6501.39	39
H25	5290.06	-1908.34	6712.4	47
H26	4999.68	-2783.41	7126.96	55
H27	4099.13	-2186.57	7331.69	48
H28A	2600.13	-130.64	7314.41	69
H28B	3286.88	-516.53	7541.21	69
H28C	2840.14	-1369.56	7306.86	69
H30A	1550.59	-407.59	5789.08	56
H30B	1905.53	-1562.77	5776.52	56
H30C	2086.29	-556.42	5578.84	56
H31A	2696.57	-827.76	6528.98	54
H31B	2089.67	-1500.61	6318.41	54
H31C	1989.09	-248.84	6399.72	54
H32A	3273.2	-868.08	5828.42	50
H32B	3069.02	-1845.56	6029.5	50
H32C	3527.21	-870.53	6201.71	50
H34A	1592.24	6801.66	5050.01	62
H34B	1170.39	6214.5	4741.69	62
H34C	1963.95	6334.05	4794.49	62
H35A	2165.93	4347.37	4759.38	65
H35B	1368.74	4262.44	4653.54	65
H35C	1771.8	3510.75	4931.71	65
H36A	1130.81	4235.13	5322.71	66
H36B	676.38	4868.17	5031.3	66
H36C	1023.95	5523.65	5341.42	66
H38A	4414.8	8341.65	5780.85	47
H38B	5029.68	9102.37	5750.75	47
H38C	4904.77	8820.42	6089	47
H39A	6028.91	8098.67	6281.64	46
H39B	6187.99	8470.95	5956.63	46
H39C	6336.27	7244.53	6077.11	46
H40A	5628.99	6508.78	5576.38	54
H40B	5515.43	7737.56	5452.97	54
H40C	4886.46	6963.18	5448.37	54
H42A	6419.89	2033.8	6855.99	65
H42B	6885.97	2612.74	7154.56	65
H42C	6453.97	3335.88	6877	65
H43A	5876.71	4318.34	7256.76	71
H43B	6372.56	3647.06	7526.28	71
H43C	5583.14	3604.34	7499.69	71
H44A	5585.21	1603.47	7476.09	76
H44B	6377.49	1615.07	7510.39	76
H44C	5904.38	982.83	7224.34	76
H45B	4059.74	5384.41	6724.28	37
H45	3643.99	6092.92	6517.71	58
H46	3197.3	6811.27	6916.21	83
H46B	3674.71	5948.65	7149.41	52
H47	2432.25	5759.86	7113.09	54
H47B	2621	5546.91	7189.49	39
H48B	1891.94	4495.29	6741.14	43
H48	2175.89	4052.71	6921.97	54
H49B	2401.4	3919.11	6350.67	34

Atom	x	y	z	U_{eq}
H49	2616.27	3408.2	6517.5	44
H50A	3884.07	3620.4	4901.76	107
H50B	3979.53	4908.25	4951.12	107
H50C	3563.92	4232.94	5154.54	107
H51A	4644.42	4623.59	5456.6	71
H51B	4965.91	4016.58	5203.45	71
H52A	4167.23	2939.68	5564.74	67
H52B	4521.82	2346.42	5322.23	67
H53A	5574.8	2729.76	5649.25	67
H53B	5217.93	3317.18	5892.45	67
H54A	5110.25	1063.41	5744.46	77
H54B	4731.05	1641.9	5980.05	77
H55A	5798.15	2050.08	6314.65	95
H55B	5619.74	781.36	6286.64	95
H56A	6745.06	1142.11	6265.88	114
H56B	6476.54	1781.2	5943.44	114
H56C	6346.85	499.19	5962.65	114

Table 14: Atomic Occupancies for all atoms that are not fully occupied in **Zn-5⁺**.

Atom	Occupancy
F2B	0.4
F2	0.6
F3	0.6
F3B	0.4
F4B	0.4
F4	0.6
C45B	0.24
H45B	0.24
C45	0.76
H45	0.76
C46	0.76
H46	0.76
C46B	0.24
H46B	0.24
C47	0.76
H47	0.76
C47B	0.24
H47B	0.24
C48B	0.24
H48B	0.24
C48	0.76
H48	0.76
C49B	0.24
H49B	0.24
C49	0.76
H49	0.76

Report created with ReportPlus

R_1=4.35%

Crystal Data and Experimental

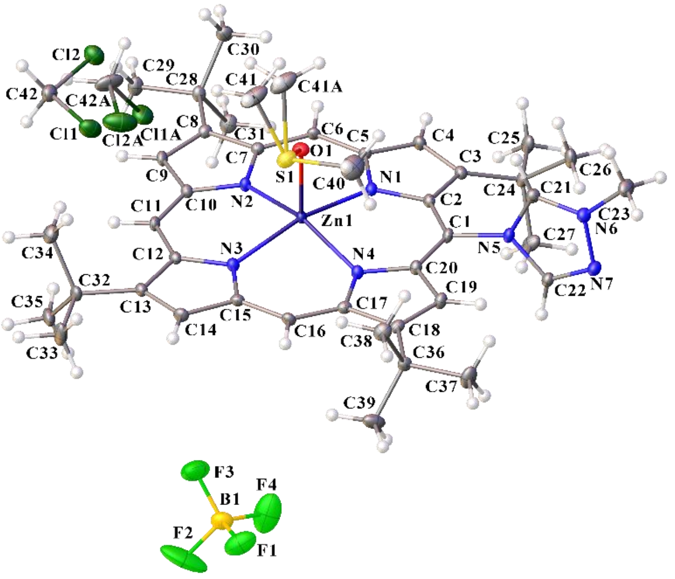

Experimental. Single clear light red prism-shaped crystals of **Zn-6+** recrystallised from a mixture of dichloromethane and heptane by slow evaporation. A suitable crystal with dimensions 0.20 × 0.15 × 0.10 mm^3 was selected and mounted on a mylar loop oil on a Bruker APEX-II CCD diffractometer. The crystal was kept at a steady T = 110 K during data collection. The structure was solved with the **ShelXT** 2018/ [6] solution program using dual methods and by using Olex2 1.5[11] as the graphical interface. The model was refined with **Shel XL** using full matrix least squares minimisation on $\boldsymbol{F}^2$.

Crystal Data. $C_{42}H_{56}BCl_2F_4N_7OSZn$, M_r = 930.07, triclinic, P-1 (No. 2), a = 12.5177(3) Å, b = 14.2011(4) Å, c = 14.7676(4) Å, α = 64.768(2)°, β = 78.945(2)°, γ = 70.887(2)°, V = 2239.61(11) Å^3, T = 110 K, Z = 2, Z' = 1, μ(Mo K$_{\alpha 1}$) = 0.772, 68242 reflections measured, 7954 unique (R_{int} = 0.0794) which were used in all calculations. The final wR_2 was 0.1012 (all data) and R_1 was 0.0435 (I≥2 σ(I)).

**Compound**	**Zn-6+**
**CCDC**	**2363634**
Formula	$C_{42}H_{56}BCl_2F_4N_7OSZn$
$D_{calc.}$/ g cm^{-3}	1.379
μ/mm^{-1}	0.772
Formula Weight	930.07
Colour	clear light red
Shape	prism-shaped
Size/mm^3	0.20×0.15×0.10
T/K	110
Crystal System	triclinic
Space Group	P-1
a/Å	12.5177(3)
b/Å	14.2011(4)
c/Å	14.7676(4)
α/°	64.768(2)
β/°	78.945(2)
γ/°	70.887(2)
V/Å^3	2239.61(11)
Z	2
Z'	1
Wavelength/Å	0.71073
Radiation type	Mo K$_\alpha$
Θ_{min}/°	2.652
Θ_{max}/°	25.109
Measured Refl's.	68242
Indep't Refl's	7954
Refl's I≥2 σ(I)	6176
R_{int}	0.0794
Parameters	579
Restraints	6
Largest Peak	0.768
Deepest Hole	-0.659
GooF	1.026
wR_2 (all data)	0.1012
wR_2	0.0913
R_1 (all data)	0.0669
R_1	0.0435

A clear light red prism-shaped-shaped crystal with dimensions 0.20 × 0.15 × 0.10 mm^3 was mounted on a mylar loop oil. Data were collected using a Bruker APEX-II CCD diffractometer operating at T = 110 K.

Data were measured using ϕ and ω scans with Mo K_α radiation. The diffraction pattern was indexed and the total number of runs and images was based on the strategy calculation from the program APEX3[10]The maximum resolution that was achieved was Θ = 25.109° (0.84 Å).

The unit cell was refined using SAINT V8.40B[10] on 9326 reflections, 14% of the observed reflections. Data reduction, scaling and absorption corrections were performed using SAINT V8.40B[10]. The final completeness is 99.60 % out to 25.109° in Θ. SADABS-2016/2 (Bruker[(10)], 2016/2) was used for absorption correction. wR_2(int) was 0.0632 before and 0.0587 after correction. The Ratio of minimum to maximum transmission is 0.9194. The absorption coefficient μ of this material is 0.772 mm^{-1} at this wavelength (λ = 0.71073Å) and the minimum and maximum transmissions are 0.450 and 0.490.

The structure was solved and the space group P-1 (# 2) determined by the ShelXT 2018/[6]structure solution program using using dual methods and refined by full matrix least squares minimisation on $\boldsymbol{F^2}$ using version 2019/1 of **XL**[8]. All non-hydrogen atoms were refined anisotropically. Hydrogen atom positions were calculated geometrically and refined using the riding model. One dichloromethane was found disordered over two positions with occupations factors converged to 0.54:0.46.

Table 15: Fractional Atomic Coordinates (×10^4) and Equivalent Isotropic Displacement Parameters (Å^2×10^3) for **Zn-6⁺**. U_{eq} is defined as 1/3 of the trace of the orthogonalised U_{ij}.

Atom	x	y	z	U_{eq}
Zn1	5575.3(3)	4441.5(3)	2841.7(2)	13.59(10)
S1	3639.6(7)	5763.5(7)	1288.2(6)	29.2(2)
O1	4900.7(17)	5312.3(17)	1418.5(15)	22.0(5)
N1	7001(2)	5023.6(19)	2514.0(17)	16.1(5)
N2	6558.6(19)	3044.1(19)	2676.8(17)	15.4(5)
N3	4314(2)	3653.9(19)	3565.6(18)	17.4(5)
N4	4772(2)	5571.6(19)	3464.0(18)	16.6(5)
N5	6268(2)	7643.7(19)	2759.9(18)	17.6(5)
N6	5902(2)	9366(2)	2069.4(18)	21.3(6)
N7	6047(2)	9099(2)	3053.5(19)	22.7(6)
C1	6216(2)	6572(2)	2925(2)	17.3(6)
C2	7151(2)	5894(2)	2601(2)	16.5(6)
C3	8345(2)	5927(2)	2320(2)	17.3(6)
C4	8829(3)	5084(2)	2033(2)	19.6(7)
C5	8022(2)	4522(2)	2163(2)	16.1(6)
C6	8303(2)	3556(2)	2027(2)	16.3(6)
C7	7657(2)	2838(2)	2285(2)	16.4(6)
C8	8027(2)	1779(2)	2224(2)	17.5(6)
C9	7115(2)	1376(2)	2594(2)	18.3(6)
C10	6214(2)	2154(2)	2873(2)	17.0(6)
C11	5150(2)	2007(2)	3268(2)	17.6(6)
C12	4241(2)	2705(2)	3584(2)	16.3(6)
C13	3107(2)	2568(2)	3956(2)	18.2(6)
C14	2536(3)	3468(2)	4143(2)	20.7(7)
C15	3278(2)	4131(2)	3913(2)	16.9(6)
C16	2993(2)	5105(2)	4035(2)	18.1(6)
C17	3665(2)	5782(2)	3839(2)	16.8(6)
C18	3315(2)	6822(2)	3937(2)	17.2(6)
C19	4227(2)	7219(2)	3594(2)	20.5(7)
C20	5132(2)	6439(2)	3306(2)	17.1(6)
C21	6024(2)	8504(2)	1895(2)	20.2(7)
C22	6255(2)	8052(2)	3453(2)	19.3(7)
C23	5609(3)	10499(3)	1368(2)	31.4(8)

Atom	x	y	z	U_{eq}
C24	9052(3)	6550(2)	2464(2)	20.6(7)
C25	10303(3)	6124(3)	2148(3)	26.1(7)
C26	8715(3)	7782(3)	1839(3)	29.3(8)
C27	8987(3)	6322(3)	3583(2)	28.9(8)
C28	9182(2)	1213(2)	1875(2)	19.8(7)
C29	9159(3)	131(3)	1913(3)	26.6(7)
C30	9523(3)	1881(3)	791(2)	26.6(7)
C31	10071(3)	994(3)	2579(3)	27.6(8)
C32	2648(3)	1643(3)	4092(2)	21.3(7)
C33	1437(3)	1830(3)	4547(3)	40.6(10)
C34	2637(3)	1599(3)	3075(3)	30.9(8)
C35	3358(3)	552(3)	4796(2)	29.9(8)
C36	2166(2)	7404(2)	4289(2)	18.8(7)
C37	2245(3)	8420(3)	4368(3)	33.5(8)
C38	1299(3)	7759(3)	3526(2)	27.1(8)
C39	1783(3)	6684(3)	5326(2)	34.5(9)
C40	3285(4)	7125(3)	1152(3)	51.3(11)
C41	3391(13)	5761(13)	173(11)	48(3)
C41A	3624(13)	6243(13)	-100(11)	48(3)
F1	5935.1(18)	873(2)	9087.8(19)	61.9(7)
F2	6920(3)	-862(3)	9803(2)	114.8(15)
F3	6898(2)	-37(2)	8151.2(19)	71.1(8)
F4	7842(2)	395(3)	8978(3)	91.6(11)
B1	6915(3)	92(4)	8994(3)	36.7(10)
Cl1	1227(2)	3908(2)	639(2)	41.3(6)
Cl2	3521.5(15)	3069.7(14)	-85.7(13)	38.4(4)
C42	2200(6)	2815(5)	375(5)	31.9(15)
Cl1A	1601(3)	4262(3)	539(3)	65.9(11)
Cl2A	3972.7(19)	3259.7(17)	991.0(15)	44.1(5)
C42A	2895(8)	3537(8)	205(6)	47(2)

Table 16: Anisotropic Displacement Parameters (×10[4]) for **Zn-6⁺**. The anisotropic displacement factor exponent takes the form: $-2\pi^2[h^2a^{*2} \times U_{11} + \ldots + 2hka^* \times b^* \times U_{12}]$

Atom	U_{11}	U_{22}	U_{33}	U_{23}	U_{13}	U_{12}
Zn1	12.42(17)	11.69(18)	17.06(18)	-6.07(14)	-0.01(13)	-3.75(13)
S1	21.4(4)	34.2(5)	32.6(5)	-17.4(4)	-5.9(4)	0.0(4)
O1	18.1(11)	26.0(12)	19.2(11)	-7.1(9)	-3.5(9)	-3.8(9)
N1	17.7(13)	13.9(13)	17.0(12)	-5.3(10)	-2.8(10)	-4.8(10)
N2	13.1(12)	13.2(13)	20.4(13)	-7.5(10)	0.0(10)	-3.6(10)
N3	16.8(13)	15.8(13)	19.6(13)	-7.0(11)	0.6(10)	-5.3(10)
N4	14.5(13)	15.5(13)	20.6(13)	-7.8(11)	-1.1(10)	-4.1(10)
N5	16.0(13)	17.4(13)	23.6(14)	-11.5(11)	-0.2(10)	-5.7(10)
N6	23.8(14)	17.3(14)	21.9(14)	-7.3(11)	-2.9(11)	-4.3(11)
N7	28.0(15)	20.8(15)	22.6(14)	-10.4(12)	-2.7(11)	-7.6(12)
C1	18.0(15)	15.5(15)	22.4(16)	-10.0(13)	-3.0(12)	-5.1(12)
C2	16.3(15)	16.8(16)	17.7(15)	-6.1(13)	-2.3(12)	-6.5(12)
C3	17.1(15)	16.9(16)	17.3(15)	-4.8(12)	-1.4(12)	-6.3(12)
C4	16.8(16)	20.3(16)	23.0(16)	-9.3(13)	2.1(12)	-7.3(13)
C5	16.0(15)	17.1(15)	15.4(14)	-6.7(12)	0.4(12)	-5.0(12)
C6	13.6(15)	17.4(15)	17.3(15)	-8.6(12)	3.0(12)	-3.4(12)
C7	15.5(15)	17.1(15)	16.1(14)	-6.7(12)	-0.9(12)	-3.8(12)
C8	17.5(15)	15.7(15)	17.9(15)	-6.2(12)	-3.3(12)	-2.0(12)
C9	20.8(16)	12.1(15)	22.1(16)	-5.3(13)	-3.0(13)	-5.7(13)
C10	17.6(15)	15.0(15)	18.0(15)	-5.2(12)	-2.0(12)	-5.3(12)
C11	21.3(16)	11.9(15)	21.1(15)	-6.9(13)	-2.3(13)	-5.3(12)
C12	16.3(15)	17.9(16)	15.4(14)	-5.0(12)	-1.5(12)	-7.3(12)
C13	18.9(16)	18.9(16)	18.3(15)	-6.9(13)	0.1(12)	-8.5(13)

Atom	U_{11}	U_{22}	U_{33}	U_{23}	U_{13}	U_{12}
C14	15.6(15)	25.1(17)	21.8(16)	-10.5(14)	4.1(12)	-7.3(13)
C15	15.3(15)	19.0(16)	14.8(14)	-6.3(12)	1.1(12)	-4.2(12)
C16	14.7(15)	19.1(16)	18.7(15)	-7.6(13)	3.9(12)	-5.0(13)
C17	15.8(15)	16.2(15)	16.0(15)	-6.4(12)	-2.4(12)	-0.5(12)
C18	16.0(15)	17.7(16)	17.8(15)	-8.6(13)	-5.0(12)	0.1(12)
C19	18.1(16)	17.8(16)	28.7(17)	-12.9(14)	-2.9(13)	-2.9(13)
C20	14.7(15)	16.4(16)	21.9(15)	-9.0(13)	-1.4(12)	-4.4(12)
C21	19.9(16)	19.4(17)	21.7(16)	-9.6(14)	-0.2(13)	-4.6(13)
C22	20.0(16)	19.6(17)	22.6(16)	-11.2(14)	-0.9(13)	-6.9(13)
C23	44(2)	18.4(17)	29.4(18)	-6.9(15)	-8.4(16)	-5.8(16)
C24	19.2(16)	23.2(17)	25.8(16)	-13.2(14)	1.8(13)	-10.7(13)
C25	18.7(17)	31.3(19)	39.0(19)	-21.6(16)	4.2(14)	-12.9(14)
C26	25.8(18)	25.5(18)	44(2)	-18.7(16)	4.8(15)	-13.9(15)
C27	19.3(17)	46(2)	32.3(19)	-23.9(17)	0.5(14)	-11.8(15)
C28	14.5(15)	16.6(16)	27.4(17)	-9.6(13)	-1.0(13)	-1.9(12)
C29	18.8(17)	22.2(18)	39(2)	-16.0(15)	-1.1(14)	-1.6(14)
C30	24.8(18)	25.8(18)	28.5(18)	-14.6(15)	5.1(14)	-4.4(14)
C31	17.6(17)	27.0(18)	37.2(19)	-14.9(16)	-4.8(14)	0.1(14)
C32	19.1(16)	23.3(17)	27.0(17)	-12.5(14)	4.0(13)	-12.3(13)
C33	28(2)	42(2)	68(3)	-37(2)	19.9(18)	-23.8(18)
C34	31.0(19)	33(2)	36(2)	-17.0(16)	-3.7(15)	-12.1(16)
C35	40(2)	24.2(18)	29.6(18)	-5.7(15)	-3.3(15)	-20.2(16)
C36	15.7(15)	17.1(16)	23.0(16)	-10.4(13)	0.3(12)	-1.2(12)
C37	22.4(18)	33(2)	55(2)	-31.4(18)	3.0(16)	-3.1(15)
C38	17.1(16)	30.7(19)	32.8(19)	-15.0(16)	-4.3(14)	-0.8(14)
C39	35(2)	28.7(19)	23.9(18)	-7.8(15)	7.7(15)	1.8(16)
C40	56(3)	32(2)	55(3)	-14(2)	-21(2)	8(2)
C41	45(5)	52(8)	45(7)	-32(6)	-25(4)	17(5)
C41A	45(5)	52(8)	45(7)	-32(6)	-25(4)	17(5)
F1	32.6(13)	91(2)	64.9(16)	-49.9(15)	-11.9(11)	9.9(13)
F2	70(2)	104(3)	58.3(18)	38.4(17)	15.0(15)	10.0(18)
F3	97(2)	66.4(18)	48.5(15)	-32.4(14)	8.7(14)	-14.4(16)
F4	33.8(15)	95(2)	170(3)	-84(2)	-14.8(17)	2.0(15)
B1	26(2)	44(3)	35(2)	-17(2)	2.8(18)	-3.4(19)
Cl1	37.6(15)	36.7(14)	39.1(12)	-15.2(10)	9.5(10)	-3.2(9)
Cl2	31.4(9)	36.5(10)	41.0(10)	-11.2(8)	2.0(8)	-9.3(8)
C42	42(4)	23(3)	31(3)	-11(3)	0(3)	-10(3)
Cl1A	49(2)	75(3)	40.4(16)	-15.6(18)	2.7(15)	12.7(16)
Cl2A	55.7(13)	35.4(11)	32.3(10)	-2.7(9)	-7.5(9)	-12.9(10)
C42A	43(5)	52(6)	35(5)	-29(5)	-1(4)	15(5)

Table 17: Bond Lengths in Å for **Zn-6⁺**.

Atom	Atom	Length/Å
Zn1	O1	2.107(2)
Zn1	N1	2.096(2)
Zn1	N2	2.052(2)
Zn1	N3	2.092(2)
Zn1	N4	2.068(2)
S1	O1	1.513(2)
S1	C40	1.765(4)
S1	C41	1.736(14)
S1	C41A	1.866(14)
N1	C2	1.369(4)
N1	C5	1.370(4)
N2	C7	1.373(4)
N2	C10	1.365(4)
N3	C12	1.369(4)
N3	C15	1.368(4)
N4	C17	1.376(4)
N4	C20	1.362(4)
N5	C1	1.457(4)
N5	C21	1.338(4)
N5	C22	1.370(4)
N6	N7	1.369(3)
N6	C21	1.312(4)
N6	C23	1.461(4)
N7	C22	1.298(4)
C1	C2	1.403(4)
C1	C20	1.405(4)
C2	C3	1.483(4)
C3	C4	1.356(4)

Atom	Atom	Length/Å
C3	C24	1.536(4)
C4	C5	1.419(4)
C5	C6	1.392(4)
C6	C7	1.389(4)
C7	C8	1.460(4)
C8	C9	1.360(4)
C8	C28	1.519(4)
C9	C10	1.435(4)
C10	C11	1.392(4)
C11	C12	1.396(4)
C12	C13	1.464(4)
C13	C14	1.359(4)
C13	C32	1.523(4)
C14	C15	1.430(4)
C15	C16	1.392(4)
C16	C17	1.384(4)
C17	C18	1.458(4)
C18	C19	1.354(4)
C18	C36	1.524(4)
C19	C20	1.445(4)

Atom	Atom	Length/Å
C24	C25	1.542(4)
C24	C26	1.542(4)
C24	C27	1.534(4)
C28	C29	1.524(4)
C28	C30	1.534(4)
C28	C31	1.534(4)
C32	C33	1.523(4)
C32	C34	1.532(4)
C32	C35	1.533(4)
C36	C37	1.532(4)
C36	C38	1.524(4)
C36	C39	1.525(4)
F1	B1	1.389(5)
F2	B1	1.372(5)
F3	B1	1.338(5)
F4	B1	1.356(5)
Cl1	C42	1.772(7)
Cl2	C42	1.755(7)
Cl1A	C42A	1.721(9)
Cl2A	C42A	1.784(9)

Table 18: Bond Angles in ° for **Zn-6⁺**.

Atom	Atom	Atom	Angle/°
N1	Zn1	O1	97.93(8)
N2	Zn1	O1	99.88(9)
N2	Zn1	N1	90.36(9)
N2	Zn1	N3	88.28(9)
N2	Zn1	N4	161.58(9)
N3	Zn1	O1	97.79(9)
N3	Zn1	N1	164.22(9)
N4	Zn1	O1	98.52(9)
N4	Zn1	N1	87.96(9)
N4	Zn1	N3	88.38(9)
O1	S1	C40	106.31(17)
O1	S1	C41	107.8(5)
O1	S1	C41A	100.4(5)
C40	S1	C41A	89.1(5)
C41	S1	C40	107.9(5)
S1	O1	Zn1	122.34(12)
C2	N1	Zn1	131.05(19)
C2	N1	C5	106.4(2)
C5	N1	Zn1	122.49(19)
C7	N2	Zn1	127.65(19)
C10	N2	Zn1	126.13(19)
C10	N2	C7	106.1(2)
C12	N3	Zn1	127.83(19)
C15	N3	Zn1	124.16(19)
C15	N3	C12	106.5(2)
C17	N4	Zn1	126.70(19)
C20	N4	Zn1	124.34(19)
C20	N4	C17	106.4(2)
C21	N5	C1	123.2(2)
C21	N5	C22	105.7(2)
C22	N5	C1	128.9(2)
N7	N6	C23	120.5(2)
C21	N6	N7	111.5(2)
C21	N6	C23	128.0(3)

Atom	Atom	Atom	Angle/°
C22	N7	N6	103.8(2)
C2	C1	N5	118.7(3)
C2	C1	C20	129.8(3)
C20	C1	N5	111.1(2)
N1	C2	C1	118.3(3)
N1	C2	C3	110.2(2)
C1	C2	C3	131.6(3)
C2	C3	C24	133.3(3)
C4	C3	C2	103.9(3)
C4	C3	C24	121.9(3)
C3	C4	C5	109.8(3)
N1	C5	C4	109.7(2)
N1	C5	C6	127.3(3)
C6	C5	C4	122.8(3)
C7	C6	C5	128.5(3)
N2	C7	C6	122.4(3)
N2	C7	C8	110.9(2)
C6	C7	C8	126.7(3)
C7	C8	C28	128.5(3)
C9	C8	C7	104.5(2)
C9	C8	C28	126.9(3)
C8	C9	C10	108.8(3)
N2	C10	C9	109.8(3)
N2	C10	C11	126.4(3)
C11	C10	C9	123.9(3)
C10	C11	C12	127.6(3)
N3	C12	C11	122.2(3)
N3	C12	C13	110.5(2)
C11	C12	C13	127.3(3)
C12	C13	C32	128.5(3)
C14	C13	C12	104.4(3)
C14	C13	C32	127.1(3)
C13	C14	C15	109.1(3)
N3	C15	C14	109.4(3)

Atom	Atom	Atom	Angle/°
N3	C15	C16	125.3(3)
C16	C15	C14	125.2(3)
C17	C16	C15	128.5(3)
N4	C17	C16	123.0(3)
N4	C17	C18	110.3(3)
C16	C17	C18	126.6(3)
C17	C18	C36	129.2(3)
C19	C18	C17	105.4(3)
C19	C18	C36	125.4(3)
C18	C19	C20	108.2(3)
N4	C20	C1	126.0(3)
N4	C20	C19	109.7(2)
C1	C20	C19	124.3(3)
N6	C21	N5	107.2(3)
N7	C22	N5	111.8(3)
C3	C24	C25	109.3(2)
C3	C24	C26	115.0(3)
C25	C24	C26	106.0(3)
C27	C24	C3	108.5(2)
C27	C24	C25	107.5(3)
C27	C24	C26	110.3(3)
C8	C28	C29	109.7(2)
C8	C28	C30	111.9(2)
C8	C28	C31	109.5(2)

Atom	Atom	Atom	Angle/°
C29	C28	C30	107.3(3)
C29	C28	C31	108.3(3)
C31	C28	C30	110.0(3)
C13	C32	C34	110.2(3)
C13	C32	C35	111.3(3)
C33	C32	C13	109.5(3)
C33	C32	C34	108.2(3)
C33	C32	C35	108.1(3)
C34	C32	C35	109.5(3)
C18	C36	C37	109.9(3)
C18	C36	C38	109.6(2)
C18	C36	C39	111.1(2)
C38	C36	C37	107.9(3)
C38	C36	C39	111.0(3)
C39	C36	C37	107.2(3)
F2	B1	F1	108.2(3)
F3	B1	F1	108.5(3)
F3	B1	F2	109.4(4)
F3	B1	F4	111.0(3)
F4	B1	F1	110.2(4)
F4	B1	F2	109.5(4)
Cl2	C42	Cl1	111.3(4)
Cl1A	C42A	Cl2A	112.2(5)

Table 19: Torsion Angles in ° for **Zn-6⁺**.

Atom	Atom	Atom	Atom	Angle/°
Zn1	N1	C2	C1	-2.7(4)
Zn1	N1	C2	C3	176.97(18)
Zn1	N1	C5	C4	-178.63(18)
Zn1	N1	C5	C6	-4.1(4)
Zn1	N2	C7	C6	6.6(4)
Zn1	N2	C7	C8	-175.41(18)
Zn1	N2	C10	C9	175.59(18)
Zn1	N2	C10	C11	-3.4(4)
Zn1	N3	C12	C11	13.3(4)
Zn1	N3	C12	C13	-166.06(19)
Zn1	N3	C15	C14	166.19(19)
Zn1	N3	C15	C16	-14.4(4)
Zn1	N4	C17	C16	13.6(4)
Zn1	N4	C17	C18	-163.30(18)
Zn1	N4	C20	C1	-17.0(4)
Zn1	N4	C20	C19	163.21(19)
N1	C2	C3	C4	2.7(3)
N1	C2	C3	C24	-165.8(3)
N1	C5	C6	C7	-5.6(5)
N2	C7	C8	C9	-0.3(3)
N2	C7	C8	C28	-177.5(3)
N2	C10	C11	C12	-3.1(5)
N3	C12	C13	C14	0.4(3)
N3	C12	C13	C32	179.4(3)
N3	C15	C16	C17	0.1(5)
N4	C17	C18	C19	1.2(3)
N4	C17	C18	C36	178.2(3)
N5	C1	C2	N1	-163.2(2)
N5	C1	C2	C3	17.2(5)
N5	C1	C20	N4	174.2(3)
N5	C1	C20	C19	-6.1(4)

Atom	Atom	Atom	Atom	Angle/˚
N6	N7	C22	N5	1.1(3)
N7	N6	C21	N5	-0.8(3)
C1	N5	C21	N6	165.9(3)
C1	N5	C22	N7	-164.9(3)
C1	C2	C3	C4	-177.6(3)
C1	C2	C3	C24	13.8(5)
C2	N1	C5	C4	0.4(3)
C2	N1	C5	C6	174.9(3)
C2	C1	C20	N4	1.8(5)
C2	C1	C20	C19	-178.5(3)
C2	C3	C4	C5	-2.4(3)
C2	C3	C24	C25	171.7(3)
C2	C3	C24	C26	-69.2(4)
C2	C3	C24	C27	54.7(4)
C3	C4	C5	N1	1.5(3)
C3	C4	C5	C6	-173.4(3)
C4	C3	C24	C25	4.8(4)
C4	C3	C24	C26	123.9(3)
C4	C3	C24	C27	-112.1(3)
C4	C5	C6	C7	168.3(3)
C5	N1	C2	C1	178.4(2)
C5	N1	C2	C3	-1.9(3)
C5	C6	C7	N2	4.4(5)
C5	C6	C7	C8	-173.2(3)
C6	C7	C8	C9	177.6(3)
C6	C7	C8	C28	0.4(5)
C7	N2	C10	C9	-0.3(3)
C7	N2	C10	C11	-179.3(3)
C7	C8	C9	C10	0.1(3)
C7	C8	C28	C29	-178.4(3)
C7	C8	C28	C30	-59.4(4)
C7	C8	C28	C31	62.9(4)
C8	C9	C10	N2	0.1(3)
C8	C9	C10	C11	179.1(3)
C9	C8	C28	C29	5.0(4)
C9	C8	C28	C30	124.0(3)
C9	C8	C28	C31	-113.7(3)
C9	C10	C11	C12	178.1(3)
C10	N2	C7	C6	-177.6(3)
C10	N2	C7	C8	0.3(3)
C10	C11	C12	N3	-2.2(5)
C10	C11	C12	C13	177.0(3)
C11	C12	C13	C14	-178.9(3)
C11	C12	C13	C32	0.1(5)
C12	N3	C15	C14	-0.7(3)
C12	N3	C15	C16	178.7(3)
C12	C13	C14	C15	-0.8(3)
C12	C13	C32	C33	177.4(3)
C12	C13	C32	C34	-63.7(4)
C12	C13	C32	C35	58.0(4)
C13	C14	C15	N3	1.0(3)
C13	C14	C15	C16	-178.4(3)
C14	C13	C32	C33	-3.8(4)
C14	C13	C32	C34	115.1(3)
C14	C13	C32	C35	-123.3(3)
C14	C15	C16	C17	179.4(3)
C15	N3	C12	C11	179.5(3)
C15	N3	C12	C13	0.2(3)
C15	C16	C17	N4	0.7(5)
C15	C16	C17	C18	177.1(3)

Atom	Atom	Atom	Atom	Angle/°
C16	C17	C18	C19	-175.6(3)
C16	C17	C18	C36	1.4(5)
C17	N4	C20	C1	-180.0(3)
C17	N4	C20	C19	0.3(3)
C17	C18	C19	C20	-1.0(3)
C17	C18	C36	C37	174.1(3)
C17	C18	C36	C38	-67.4(4)
C17	C18	C36	C39	55.6(4)
C18	C19	C20	N4	0.5(3)
C18	C19	C20	C1	-179.3(3)
C19	C18	C36	C37	-9.5(4)
C19	C18	C36	C38	109.0(3)
C19	C18	C36	C39	-128.0(3)
C20	N4	C17	C16	176.0(3)
C20	N4	C17	C18	-0.9(3)
C20	C1	C2	N1	8.7(5)
C20	C1	C2	C3	-170.9(3)
C21	N5	C1	C2	83.6(3)
C21	N5	C1	C20	-89.7(3)
C21	N5	C22	N7	-1.6(3)
C21	N6	N7	C22	-0.2(3)
C22	N5	C1	C2	-115.7(3)
C22	N5	C1	C20	71.0(4)
C22	N5	C21	N6	1.4(3)
C23	N6	N7	C22	177.9(3)
C23	N6	C21	N5	-178.6(3)
C24	C3	C4	C5	167.8(3)
C28	C8	C9	C10	177.4(3)
C32	C13	C14	C15	-179.8(3)
C36	C18	C19	C20	-178.1(3)
C40	S1	O1	Zn1	95.5(2)
C41	S1	O1	Zn1	-149.1(6)
C41A	S1	O1	Zn1	-172.4(5)

Table 20: Hydrogen Fractional Atomic Coordinates (×10[4]) and Equivalent Isotropic Displacement Parameters (Å^2×10^3) for **Zn-6⁺**. U_{eq} is defined as 1/3 of the trace of the orthogonalised U_{ij}.

Atom	x	y	z	U_{eq}
H4	9592.19	4896.89	1783.22	24
H6	9033.05	3362.11	1717.38	20
H9	7081.07	691.44	2658.19	22
H11	5029.29	1354.96	3329.9	21
H14	1766.32	3630.09	4386.87	25
H16	2238.07	5334.36	4286	22
H19	4264.7	7894.62	3551.17	25
H21	5952.53	8491.62	1271.27	24
H22	6382.91	7621.24	4141.85	23
H23A	6252.43	10793.21	1254.65	47
H23B	4950.64	10915.72	1648.46	47
H23C	5428.06	10547.47	729.64	47
H25A	10374.31	6267.48	1431.81	39
H25B	10567.89	5342.59	2536.34	39
H25C	10762.25	6492.35	2275.07	39
H26A	8067.62	8136.56	2182.13	44
H26B	8507.9	7923.05	1176.64	44
H26C	9356.04	8067.84	1761.39	44
H27A	9379.99	5560.93	3953.51	43
H27B	8191.65	6470.82	3833.93	43

Atom	x	y	z	U_{eq}
H27C	9347.62	6788.58	3677.11	43
H29A	8598.04	255.83	1464.38	40
H29B	8955.42	-321.68	2600.25	40
H29C	9908.96	-236.72	1698.27	40
H30A	9555.62	2581.7	741.93	40
H30B	8962.15	1999.72	344.87	40
H30C	10268.54	1489.09	593.06	40
H31A	9846.07	569.54	3269.25	41
H31B	10127.6	1684.51	2536.05	41
H31C	10807.78	589.44	2379.33	41
H33A	961.93	2512.2	4096.83	61
H33B	1425.1	1865.63	5197.36	61
H33C	1144.57	1230.72	4640.96	61
H34A	3410.83	1475.5	2770.49	46
H34B	2160.4	2287.4	2633.23	46
H34C	2332.42	1007.07	3171.03	46
H35A	3435.07	605.19	5418.96	45
H35B	4110.18	363.63	4470.12	45
H35C	2982.28	-9.55	4947.67	45
H37A	2436.68	8925.91	3702.01	50
H37B	2833.11	8216.92	4821.41	50
H37C	1514.52	8767.27	4629.8	50
H38A	1240.82	7122.01	3456.05	41
H38B	1540.33	8255.32	2876.46	41
H38C	559.79	8127.76	3756.35	41
H39A	2379.78	6419.11	5789.63	52
H39B	1633.78	6067.86	5286.18	52
H39C	1090.37	7104.07	5568.63	52
H40A	3802.48	7491.57	640.32	77
H40B	3349.26	7147.7	1793.19	77
H40C	2505.14	7491.27	948.08	77
H41A	3590.54	5012.76	225.86	71
H41B	3854.63	6160.32	-377.96	71
H41C	2588.33	6108.85	39.93	71
H41D	3895.73	5617.93	-290.79	71
H41E	4119.41	6720.56	-431.36	71
H41F	2849.76	6640.55	-306.98	71
H42A	2296.04	2146.38	994.85	38
H42B	1893.35	2698.47	-126.56	38
H42C	3117.43	3951.47	-498.86	56
H42D	2827.32	2844.01	246.72	56

Table 21: Atomic Occupancies for all atoms that are not fully occupied in **Zn-6⁺**.

Atom	Occupancy
C41	0.486(15)
H41A	0.486(15)
H41B	0.486(15)
H41C	0.486(15)
C41A	0.514(15)
H41D	0.514(15)
H41E	0.514(15)
H41F	0.514(15)
Cl1	0.54
Cl2	0.54

References

(1) Liebeskind, L. S.; Liu, W. Synthesis of 4-Substituted Pyrrole-2-Carbaldehyde Compounds. WO2001028997A2, 2001.
(2) Nickel, E. G.; Liebeskind, L. S. A Method for Synthesizing Porphyrin Compounds. WO2000052012A2, 2000.
(3) Whitlock, B. J.; Whitlock, H. W.; Alles, H. Cyclotetramerization of 2-Dimethylamino-4-Tert-Butylpyrrole. Tetra-Tert-Butylporphyrins. *J. Am. Chem. Soc.* **1974**, *96* (12), 3959–3965. https://doi.org/10.1021/ja00819a038.
(4) Adler, A. D.; Longo, F. R.; Kampas, F.; Kim, J. On the Preparation of Metalloporphyrins. *J. Inorg. Nucl. Chem.* **1970**, *32*, 2443.
(5) Fulmer, G. R.; Miller, A. J. M.; Sherden, N. H.; Gottlieb, H. E.; Nudelman, A.; Stoltz, B. M.; Bercaw, J. E.; Goldberg, K. I. NMR Chemical Shifts of Trace Impurities: Common Laboratory Solvents, Organics, and Gases in Deuterated Solvents Relevant to the Organometallic Chemist. *Organometallics* **2010**, *29* (9), 2176–2179. https://doi.org/10.1021/om100106e.
(6) Sheldrick, G.M. ShelXT-Integrated Space-Group and Crystal-Structure Determination. *Acta Cryst* **2015**, *A71*, 3–8.
(7) Sheldrick, G. M. A Short History of SHELX. *Acta Cryst A* **2008**, *64*, 112–122. https://doi.org/10.1107/S0108767307043930.
(8) Sheldrick, G. M. Crystal Structure Refinement with ShelXL. *Acta Cryst* **2015**, *C27*, 3–8.
(9) Bruker V8.34A : SADABS, SAINT, APEX2. Bruker AXS Inc., Madison, Wisconsin, USA. (2014).
(10) Dolomanov, O. V.; Bourhis, L. J.; Gildea, R. J.; Howard, J. a. K.; Puschmann, H. Bruker SAINT (V8.40B, 2016), APEX3 (Bruker, 2013, 2015, 2020) & SADABS (2016). Bruker AXS Inc., Madison, Wisconsin, USA. *J. Appl. Crystallogr.* **2009**, *42* (2), 339–341. https://doi.org/10.1107/S0021889808042726.
(11) Dolomanov, O. V.; Bourhis, L. J.; Gildea, R. J.; Howard, J. A. K.; Puschmann, H. OLEX2: A Complete Structure Solution, Refinement and Analysis Program. *J. Appl. Crystallogr.* **2009**, *42* (2), 339–341. https://doi.org/10.1107/S0021889808042726.
